# Transcriptomic Analysis Based on RNA-Seq Technology Reveals the Molecular Mechanisms of Sunflower (*Helianthus annuus* L.) Response to Salt Stress

**DOI:** 10.3390/genes17060629

**Published:** 2026-05-30

**Authors:** Yanfang Zhang, Jiaxin Xie, Shuchun Guo, Mengjie Liu, Haijun Chen, Min Xie, Ruifen Sun, Xiuwen Huo

**Affiliations:** 1 Horticulture Department, College of Horticulture and Plant Protection, Inner Mongolia Agricultural University, Hohhot 010019, China; 2 Inner Mongolia Academy of Agricultural and Animal Husbandry Sciences, Hohhot 010031, China; 3 Wuyuan County Bureau of Agricultural, Animal Husbandry and Science and Technology, Bayannur 015100, China

**Keywords:** differentially expressed genes, RNA sequencing, salt stress, sunflower, transcription factors

## Abstract

**Background/Objectives**: Sunflower (*Helianthus annuus* L.) is one of the four major oil crops worldwide and possesses strong stress tolerance. However, salt stress remains limiting in the improvement of sunflower yield and quality. **Methods**: In this study, the salt-tolerant cultivar P50 and salt-sensitive cultivar P29 were used as experimental materials to conduct transcriptome sequencing on root and leaf samples treated with NaCl. Subsequently, the molecular mechanisms underlying salt tolerance in sunflower were revealed through assembly and splicing, functional annotation, differential expression analysis, enrichment analysis, and transcription factors (TFs) prediction. **Results**: Results showed that 54,860,184 and 60,601,572 high-quality clean reads were obtained from the two cultivars, respectively. A total of 110,751 all-unigenes were generated after assembly and clustering, of which 77,536 were functionally annotated. A total of 21,332 differentially expressed genes (DEGs) were identified, including 10,306 upregulated and 11,026 downregulated genes. Quantitative real-time PCR validation of 15 DEGs showed a 93.33% consistency rate with the sequencing data. GO enrichment analysis indicated that DEGs were significantly enriched in pathways related to antioxidant enzyme activities. KEGG enrichment analysis demonstrated that DEGs were primarily involved in 15 carbohydrate metabolism pathways, especially starch and sucrose metabolism. In addition, 67 differentially expressed TF families containing 528 DEGs were identified, including bHLH, AP2/ERF-ERF, MYB, C3H, WRKY, EREBP, B3-ARF, and NAC. **Conclusions**: Our study constructed a comprehensive transcription map of the sunflower response to salt stress and systematically elucidated the molecular mechanisms underlying salt tolerance. The salt-tolerant sunflower cultivar P50 exhibits an efficient salt stress defense system via three core strategies: (i) activating the antioxidant system to rapidly scavenge excess reactive oxygen species and mitigate oxidative damage; (ii) regulating carbohydrate metabolism through starch and sucrose redistribution to provide energy and osmotic protection against physiological drought; and (iii) mobilizing multiple TF families to establish a complex regulatory network for the precise control of downstream functional genes.

## 1. Introduction

Sunflower (*Helianthus annuus* L.) is one of the four major oil crops worldwide, making substantial contributions to edible oil supply and agricultural economic development [1]. In addition to high economic value, sunflower also holds important implications for environmental protection and ecological restoration. Owing to its remarkable salt tolerance, drought resistance, and adaptability to barren soils, sunflower has emerged as a key candidate for rehabilitating marginal lands [2,3]. Consequently, it is widely recognized as a preferred crop for the development and utilization of saline–alkali soils in arid and semi-arid regions of China, such as Inner Mongolia. Notably, although sunflower has developed intrinsic regulatory mechanisms to cope with salt stress during long-term evolutionary adaptation, the molecular mechanisms underlying its salt tolerance remain largely unelucidated. This knowledge gap severely limits the expansion of salt-tolerant gene resources in sunflower and impedes the effective enhancement of its salt tolerance through molecular breeding. Therefore, a comprehensive exploration of the mechanisms of sunflower response to salt stress at the molecular level is urgently required.

Salt tolerance in plants is a complex trait governed by numerous genes. The response of genes to salt stress involves multiple processes including osmotic adjustment, ion balance, signal transduction, and antioxidant stress [4]. Transcriptomics focuses on the dynamic expression profiles of transcribed RNAs in a given biological system under specific conditions, constituting an important link between genomics and proteomics. Transcriptome research can capture the dynamic patterns of differentially expressed genes (DEGs) in response to environmental stress [5,6,7]. This allows researchers to identify stress-responsive genes and reconstruct associated regulatory pathways, laying the foundation for understanding complex stress-tolerance traits. RNA sequencing (RNA-seq) technology is a powerful transcriptomic approach in the field of gene expression analysis. Compared with traditional Sanger sequencing, RNA-seq technology offers the advantages of high throughput and cost-effectiveness. In addition, RNA-seq technology exhibits superior accuracy and sensitivity in detecting both low-abundance and high-abundance gene expression [8,9].

To date, RNA-seq technology has been successfully applied to dissect mechanisms of response to salt stress at the transcriptomic level in numerous plant species, such as rice [10], soybean [11], Alfalfa [12], barley [13]. As a result, numerous genes associated with salt tolerance have been identified. The genes are mainly involved in secondary metabolism, signal transduction, transcription, transport, and disease resistance. It is worth noting that RNA-seq technology has also been employed to explore the molecular mechanism of *Verticillium dahliae* resistance in sunflower [14], which proves its feasibility in sunflower transcriptome research.

Given the lack of systematic research on mechanisms of response salt stress in sunflower, the present study aimed to clarify the molecular mechanisms of sunflower salt tolerance using RNA-seq technology. Our study performed a comparative transcriptome analysis of salt-tolerant and salt-sensitive sunflower varieties under NaCl treatment. Subsequently, we identified DEGs, and further conducted Gene Ontology (GO) and Kyoto Encyclopedia of Genes and Genomes (KEGG) functional enrichment analyses on these genes. Furthermore, candidate transcription factors (TFs) involved in the regulation of salt stress were also predicted. These findings not only reveal the molecular mechanism of sunflower response to salt stress, but also provide valuable candidate genes for genetic engineering and molecular breeding of salt-tolerant sunflower varieties. This study can provide a theoretical basis for the stable production of oil crops and the sustainable utilization of the saline–alkali land.

## 2. Materials and Methods

### 2.1. Plant Materials and Treatment Details

The salt-tolerant (P50) and salt-sensitive (P29) sunflower varieties were provided by the Institute of Crop Sciences, Inner Mongolia Academy of Agricultural and Animal Husbandry Sciences. Healthy, plump and uniformly sized P50 and P29 seeds were soaked in water at room temperature for 24 h, and then sown separately in nutrient pots filled with vermiculite. The pots were placed in a tissue culture chamber at 24 ± 2 °C with 16 h light/8 h dark photoperiod. After the seeds germinated and developed the first pair of true leaves, the seedlings were carefully uprooted. The vermiculite adhering to the roots was rinsed off with water. Then, the seedlings were transferred to 1/2 Murashige and Skoog nutrient solution and cultivated for 4–5 d to acclimatize to the growth environment. Next, 20 seedlings were transferred to 1/2 Murashige and Skoog nutrient solution supplemented with 120 mmol/L NaCl for salt stress treatment, whereas an equal number of control seedlings were maintained in 1/2 Murashige and Skoog nutrient solution (based on our preliminary experiments, this concentration effectively induces salt stress responses in sunflower without causing lethality [15]). Samples of roots, hypocotyls, and young leaves were collected from five NaCl-treated seedlings at 1, 2, and 3 days post-treatment, while corresponding tissues were collected from five control seedlings at 0 and 3 days post-treatment. Three independent biological replicates were performed.

Samples were immediately frozen in liquid nitrogen and submitted to Shenzhen Huada Gene Co., Ltd. (Shenzhen, China) for RNA extraction, quality control, library construction (RNA extracted from P50 and P29 under different treatment were mixed in equal amounts). Sequencing was performed on the Illumina HiSeq^TM^2000 platform (Illumina, Inc., San Diego, CA, USA).

### 2.2. Transcriptome Assembly

After the sequencing data was off, the raw reads were filtered to remove the dirty raw reads containing repetitive bases, adaptors, and low-quality bases. The remaining high-quality clean reads were used for de novo assembly with Trinity software V2.0.3 (a short-read assembling program) to obtain unigenes of P50 and P29, respectively. The obtained unigenes were spliced, and the redundancy and homologous clustered transcripts were eliminated using the Tgicl software v2.1.

### 2.3. Prediction of Coding Sequences

First, all unigene sequences were aligned via BLASTx (https://blast.ncbi.nlm.nih.gov/Blast.cgi) (accessed on 28 May 2026) E-value < 0.00001 against public protein databases, following the priority order of the NCBI Non-Redundant Protein Sequence Database (NR), Swiss-Prot, KEGG, and Clusters of Orthologous Groups (COG). Subsequently, coding sequences (CDSs) were determined based on the BLASTx results, and the coding region was then translated into an amino acid sequence using the standard codon table. Next, the nucleotide sequences (5′ → 3′) and amino acid sequences of the unigene coding region were obtained. For unigenes with no database hits, ESTScan was performed to derive the nucleotide sequence (5′ → 3′) orientation and amino acid sequence of the predicted coding region.

### 2.4. Identification of DEGs Between P50 and P29 in Response to Salt Stress

The DEGs between P50 and P29 were identified using the FPKM method, with cutoff values of |log_2_ Fold-change| ≥ 1 and FDR < 0.001. and their expression fold changes were calculated from the FPKM values. Then, the differential expression fold of the genes was calculated based on their FPKM-quantified expression levels. Greater significance in differential expression is associated with a larger fold change and a smaller FDR. The raw data have been submitted to the NCBI under the accession numbers PRJNA343571 and PRJNA343345.

### 2.5. Validation by the Real-Time Fluorescence Quantitative PCR

The expression patterns of 15 DEGs were validated using the real-time fluorescence quantitative PCR (RT-qPCR) analysis. Briefly, total RNA was extracted from samples using an RNA extraction kit (RNAiso Plus, Takara, Dalian, China). Subsequently, cDNA was synthesized from 1.5 µg of total RNA using PrimeSript RT reagent Kit with gDNA Eraser (Perfect Real Time) (Takara, Japan) following the manufacturer’s instructions. The 2 × SYBR-Green I RT-qPCR Master Mix (Takara, Japan) was used as a labeling agent, while *18S rRNA* (accession number: HM638219) of sunflower served as the internal reference gene (verification confirmed that the expression level of this gene remained relatively stable under salt stress [16]). The reaction mixture (20 µL) contained 10 µL 2 × Master Mix, 1.0 µL each of forward and reverse primers (10 µmol/L), 2 µL template cDNA (diluted to 200 ng/µL with deionized water), and 6 µL RNase-free deionized water. The thermal cycling protocol was as follows: an initial denaturation at 95 °C for 30 s, followed by 40 cycles of 95 °C for 5 s and 60 °C for 30 s, and a final cycle of 95 °C for 5 s and 60 °C for 1 min. All reactions were performed in triplicate for each sample on a LightCycler 480 PCR system (Roche, Basel, Switzerland). The relative mRNA expression levels of the selected unigenes were normalized to *18S rRNA* and calculated using the relative 2^−ΔΔCT^ method. Results are expressed as the mean standard deviations of three replicates. Primer sequences of RT-qPCR are shown in Table 1.

### 2.6. GO and KEGG Enrichment Analysis of DEGs Between P50 and P29 in Response to Salt Stress

DEGs were mapped to each term of the GO database (http://www.geneontology.org/) (accessed on 15 July 2022) based on the NR annotation, and then the number of genes per term were calculated. Next, the hypergeometric test was applied to compare DEGs with the sunflower transcriptome, aiming to identify significantly enriched GO terms. GO functional enrichment analysis revealed the main biological functions of the DEGs.

We used the KEGG database (https://www.kegg.jp/) (accessed on 25 July 2022) to screen for the main pathways associated with the DEGs. By comparing the DEGs against the sunflower transcriptome, pathways with q-value ≤ 0.05 were defined as significantly enriched in DEGs.

### 2.7. Prediction of TFs Between P50 and P29 in Response to Salt Stress

**Figure 1:**
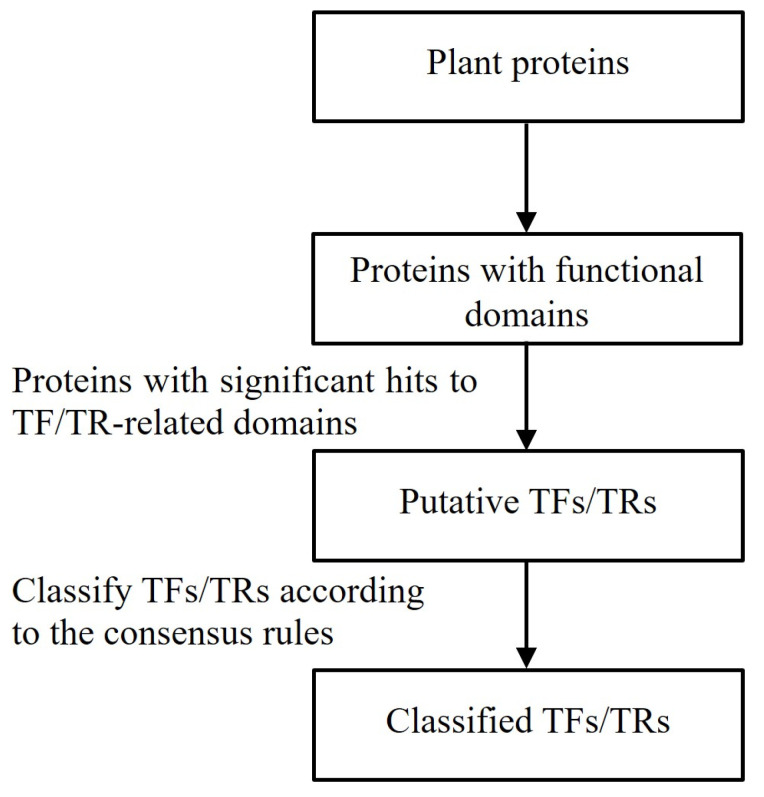
The workflow of the iTAK program to identify transcription factors (TFs).

The DEGs (P29-vs-P50.diff annot.Xls) were compared using iTAK V2.0.1 software (http://itak.feilab.net/cgi-bin/itak/index.cgi, accessed on 7 September 2025) (https://github.com/kentnf/iTAK, accessed on 21 December 2025). The comparison was performed following the method described by Zheng et al. [17]. The output file of iTAK (TFs_classification.txt) contains the classification statistics. The detail of workflow is illustrated in Figure 1.

## 3. Results

### 3.1. Transcriptome Sequencing and De Novo Assembly

**Figure 2:**
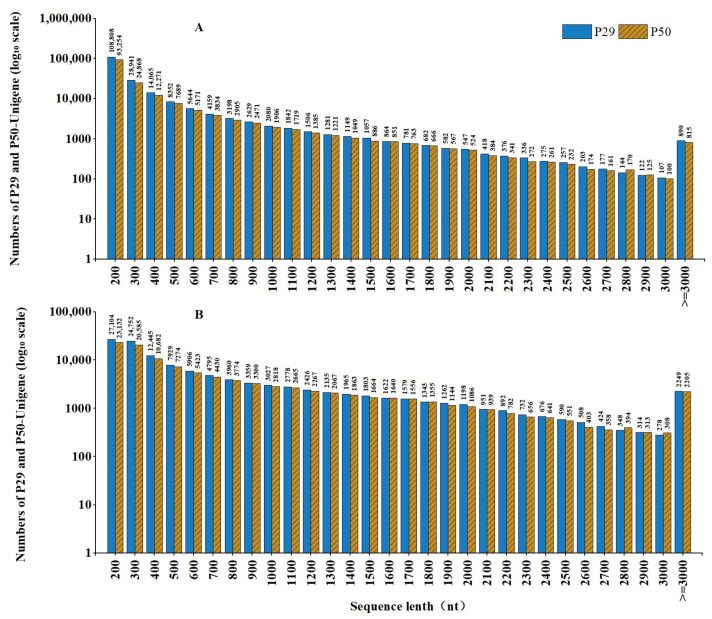
The length distribution of contigs (**A**) and unigenes (**B**) for P29 and P50.

cDNA libraries from P50 and P29 were constructed and sequenced on an Illumina HiSeq 2000^TM^ platform. Results indicated that the P50 and P29 yielded 57,232,364 and 63,276,298 raw reads, respectively. After filtering, 54,860,184 (P50) and 60,601,572 (P29) clean reads were obtained, corresponding to total nucleotides of 4,937,416,560 nt and 5,454,141,480 nt, respectively (Table 2). De novo assembly of these short reads yielded 106,275 and 119,350 unigenes for P50 and P29 individually, which had average lengths of 716 nt and 685 nt as well as N50 lengths of 1301 nt and 1263 nt, respectively. A combined assembly of both samples generated 110,751 all-unigenes, with a total length of 96,970,017 nt, an average length of 876 nt, and an N50 length of 1409 nt. In addition, there are 1494 unigenes (1.35% of all unigenes) longer than 2000 nt (Table 3). The size distributions of contigs and unigenes are shown in Figure 2, respectively.

### 3.2. Prediction of CDS

**Figure 3:**
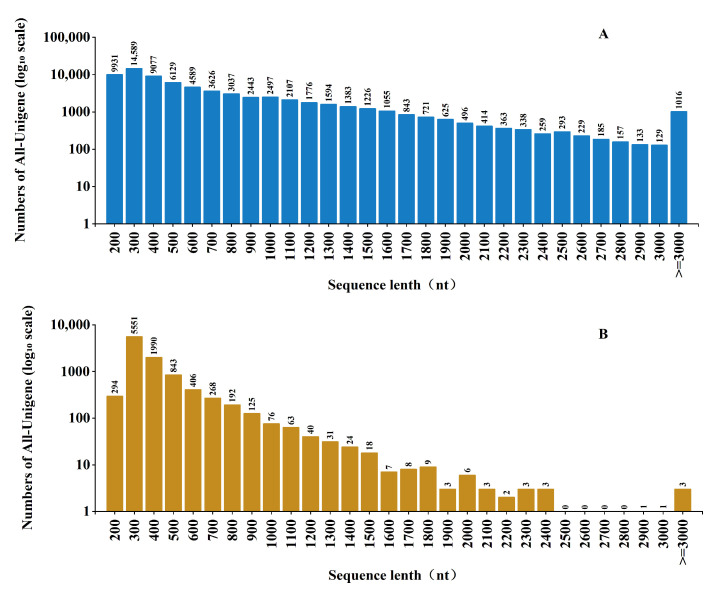
The length distribution of coding sequences. (**A**) The result of length distribution by BLAST to analyze coding sequences. (**B**) The result of length distribution by EST scan to analyze coding sequences.

All-unigene sequences were aligned against protein databases using BLASTx (E-value < 0.00001), following the priority order of NR, Swiss-Prot, KEGG, and COG. A total of 71,260 CDS were extracted from the matched unigenes and further translated into peptide sequences. For unigenes with no blast hits, 9970 CDS were predicted using ESTScan and translated into peptide sequences. A total of 81,230 CDS were obtained, and their distribution is shown in Figure 3.

### 3.3. Identification of DEGs

**Figure 4:**
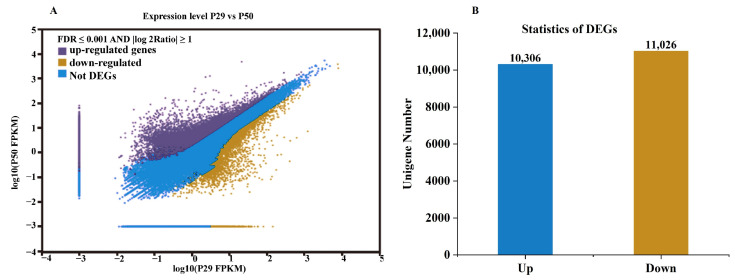
Screening of differentially expressed genes for P29 and P50. (**A**) Expression levels of all-unigenes; (**B**) numbers of differentially expressed genes.

**Figure 5:**
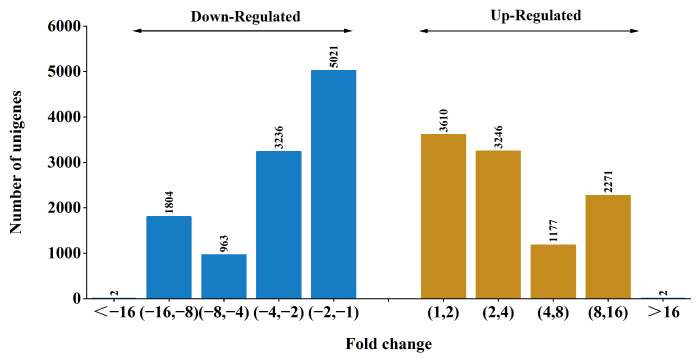
The distributions of transcript changes for P29 and P50.

All genes were categorized into three groups based on their expression patterns: red denotes genes upregulated in P50 relative to P29, green indicates those downregulated in P50 relative to P29, and blue represents genes with no differential expression. The X-axis and Y-axis represent the expression levels of P29 and P50, respectively (Figure 4A). A total of 21,332 DEGs were identified (|Log_2_Ratio| ≥ 1 and FDR ≤ 0.001), comprising 10,306 upregulated (red bars) and 11,026 downregulated (green bars) genes (Figure 4B). Transcripts ranged from −17 to 16, and the distribution of changes is shown in Figure 5.

### 3.4. RT-qPCR Validation

**Figure 6:**
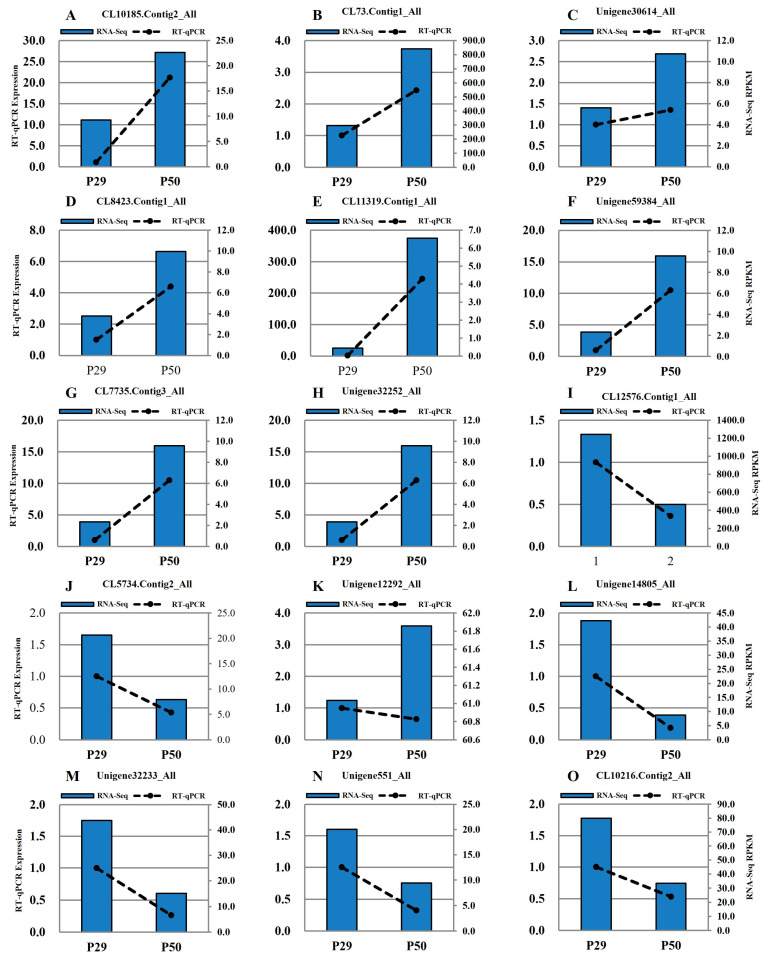
Comparison of the real-time fluorescence quantitative PCR and transcriptome results of differentially expressed genes from sunflower. Panels (**A**–**H**) represent up-regulated genes, and Panels (**I**–**O**) represent down-regulated genes.

To validate the RNA-Seq expression data, we selected 15 responsive DEGs (including CL10185.Contig2_All, CL12576.Contig1_All, CL5734.Contig2_All and CL73.Contig1_All) of salt stress for RT-qPCR analysis (Table 4). The results demonstrated that the expression levels of these unigenes were highly consistent with the transcript level estimated from the sequencing data, with the exception of Unigene12292_All. And the concordance rate reached 93.33% (Figure 6).

### 3.5. GO and KEGG Enrichment Analysis of DEGs

**Figure 7:**
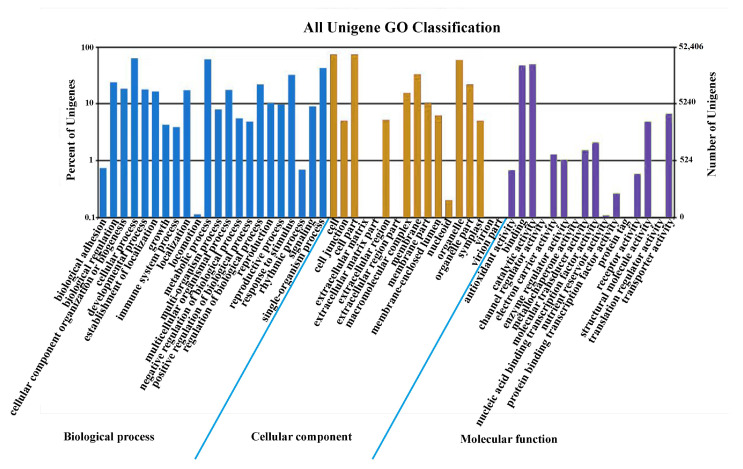
GO enrichment classification.

**Figure 8:**
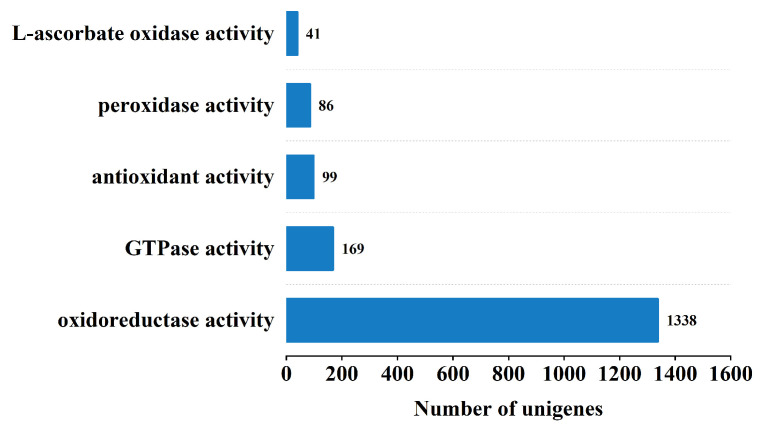
Molecular functions significantly associated with salt stress responses. The number and names of genes enriched in each molecular function are listed in the Table S1.

Results showed that the DEGs were significantly enriched in 55 GO terms. These included 17 terms in the cellular component category, 16 terms in molecular function category, and 22 terms in biological process category (Figure 7). Within the molecular function category, 1733 DEGs (23.19%) were specifically enriched in including oxidoreductase, GTPase, antioxidant, peroxidase, and L-ascorbate oxidase activities (Figure 8).

**Figure 9:**
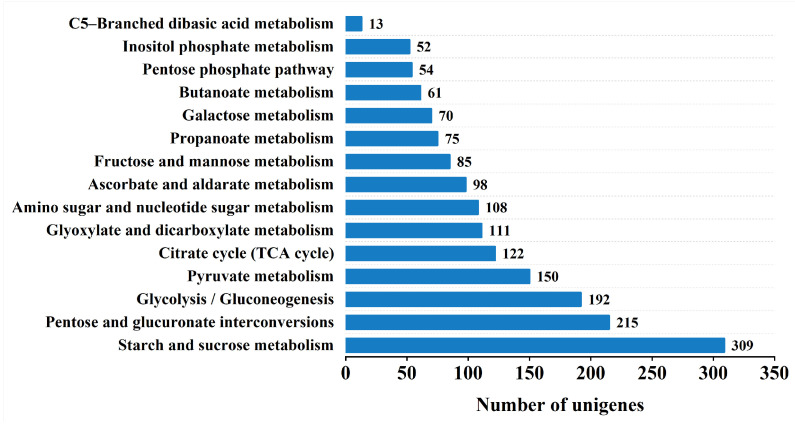
Carbohydrate metabolism pathway in differentially expressed genes of KEGG enrichment analysis.

KEGG pathway enrichment analysis indicated that DEGs were significantly enriched in 128 metabolic pathways, which spanned 20 functional categories such as amino acid metabolism, lipid metabolism, carbohydrate metabolism, biosynthesis of other secondary metabolites, other amino acid metabolism, energy metabolism, metabolism of terpenoids and polyketides, glycan biosynthesis, and metabolism and signal transduction (Figure S1). Among them, carbohydrate metabolism pathways were the most significant pathway (15 pathways), with the highest abundance of DEGs regulating the metabolism of starch and sucrose, followed by pentose and glucuronate interconversions and glycolysis/gluconeogenesis (Figure 9).

### 3.6. Prediction of TFs

**Figure 10:**
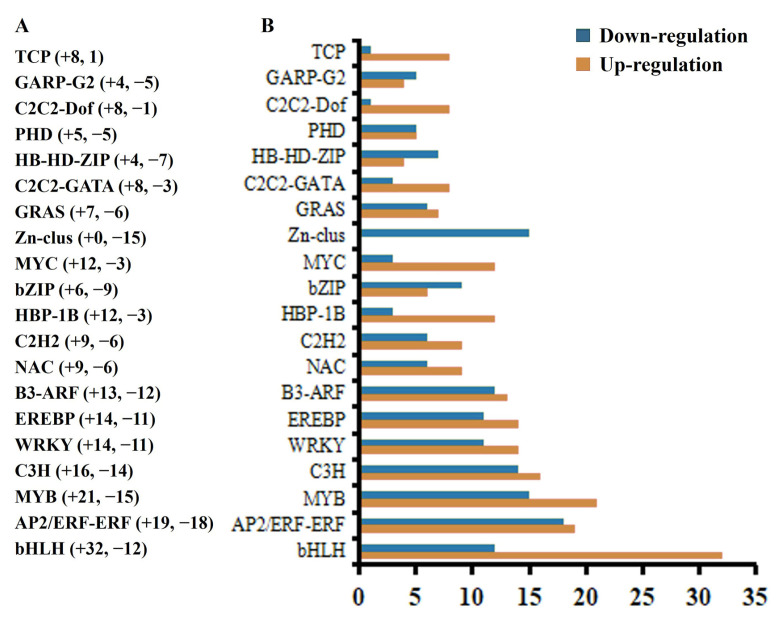
The distribution of transcription factors in gene families (top 20 most abundant families). (**A**) The distribution of transcription factors according to the gene family information; (**B**) differentially expressed genes from every gene family involved in transcription.

In this study, 67 TF factor families were identified from the 21,332 DEGs. These families comprised 528 DEGs (302 upregulated, 226 downregulated) (Figure S2). The largest gene family was the bHLH family (32 upregulated, 12 downregulated), followed by the AP2/ERF-ERF family (19 upregulated, 18 downregulated), MYB family (21 upregulated, 15 downregulated), C3H family (16 upregulated, 14 downregulated), WRKY family (14 upregulated, 11 downregulated), EREBP family (14 upregulated, 11 downregulated), B3-ARF family (13 upregulated, 12 downregulated), and NAC family (9 upregulated, 6 downregulated) (Figure 10).

## 4. Discussion and Conclusions

High-quality transcriptome data serves as the foundation for differential expression analysis and functional annotation. In this study, the salt-tolerant sunflower cultivar P50 and the salt-sensitive cultivar P29 were sequenced using the Illumina HiSeq^TM^ 2000 platform, generating a sufficient number of nucleotide sequences. De novo assembly results revealed that the N50 length of unigenes exceeded 1200 nt, indicating high assembly quality, as a higher N50 value generally reflects better assembly completeness. Furthermore, RT-qPCR validation of 15 DEGs achieved a 93.33% concordance rate with the sequencing data. These results demonstrate that the transcriptome dataset generated in this study possesses high coverage and accuracy and can reliably support subsequent analysis.

Salt stress disrupts the balance between the production and scavenging of reactive oxygen species (ROS) in plant cells [18]. The excessive accumulation of ROS attacks biological macromolecules such as proteins, lipids, and nucleic acids, causing oxidative damage and further impairing physiological metabolism [19,20,21]. In this study, more than one thousand DEGs were significantly enriched in antioxidant-related GO terms, including oxidoreductase activity, GTPase activity, antioxidant activity, peroxidase activity, and L-ascorbate oxidase activity. This finding indicates that the antioxidant defense system is transcriptionally activated under salt stress and may contribute to mitigating salt-induced oxidative damage in sunflower. It is worth noting that the upregulation levels of multiple antioxidant-related genes in P50 were significantly higher than those in P29 under salt stress. This discrepancy aligns with our previous physiological observations. Studies have shown that the superoxide dismutase activity (SOD) and peroxidase activity (POD) in P50 showed markedly greater increases compared to P29 under salt stress [15]. The transcriptomic data obtained in this study further clarify the molecular basis of this phenomenon. Under salt stress, P50 more efficiently activates genes encoding key antioxidant enzymes such as peroxidase and ascorbate oxidase, thereby quickly scavenging excess ROS within cells and reducing oxidative damage. These results further support that enhancing the antioxidant defense system is a crucial strategy for improving salt tolerance in sunflower. In addition, the significant enrichment of GTPase-related genes in this study suggests their potential function as important signaling molecules involved in the antioxidant defense system and related regulatory pathways. Similar findings have been reported in studies on *Piriformospora indica*. Nivedita et al. [22] demonstrated that Rho-type GTPase expression was upregulated in *Piriformospora indica* under salt stress, subsequently activating pathways associated with osmotic adaptation, salt stress response, and cell growth, and thus alleviating the adverse effects. These insights provide the valuable foundation for further exploration of the salt stress signal transduction mechanism in sunflower.

In addition to direct oxidative damage, salt stress disrupts intracellular and extracellular osmotic balance, leading to cellular dehydration and physiological drought, which poses another major challenge for plants [23,24]. KEGG pathway enrichment analysis revealed that DEGs were primarily enriched in carbohydrate metabolism pathways, particularly starch and sucrose metabolism. This finding indicates that salt-stressed sunflowers achieve carbohydrate redistribution by regulating the expression of genes associated with starch and sucrose metabolism. Such regulation may involve accelerating starch degradation to convert starch into more soluble sugars. On the one hand, this process provides sufficient energy for plants to cope with salt stress. On the other hand, soluble sugars act as osmoprotectants to maintain cellular pressure balance, preserve the integrity of the membrane system and the stability of the protein structure, thereby effectively alleviating salt-induced physiological drought [25,26,27]. This response pattern is consistent with observations in Prunellae Spica [28] and pigeonpea [29], suggesting that the regulation of carbohydrate metabolism is an effective strategy for plants to battle salt stress. Furthermore, sugar molecules may also serve as signaling molecules to coordinately regulate the expression of stress-responsive genes. In this study, we elucidated the expression profiles of key DEGs involved in the starch and sucrose metabolic pathways of the sunflower under salt stress. These results lay the foundation for the further identification of core regulatory genes involved in sugar transport and synthesis, as well as for the dissection of the osmotic adjustment mechanism in the sunflower.

As molecular switches governing gene expression, TFs can specifically bind to the promoter regions of target genes to regulate the transcription initiation and expression levels of downstream functional genes [30,31]. They play a pivotal role in integrating salt stress signals, activating the expression of defense genes, and establishing the regulatory network under salt stress [32,33,34]. In this study, we identified 67 TF families involving 528 DEGs, with 302 genes showing upregulation and 226 genes exhibiting downregulation. These findings highlight the complexity and diversity of the regulatory network under salt stress in sunflower. Among all TFs, the bHLH family exhibited the largest number of 44 DEGs, with 32 upregulated and 12 downregulated genes. Previous studies have confirmed that bHLH family members can enhance plant salt tolerance by maintaining cellular osmotic homeostasis, promoting ROS scavenging, and regulating the accumulations of resistance-related secondary metabolites [35,36]. In the present study, the extensive upregulation of bHLH genes in the salt-tolerant sunflower cultivar P50 implies that these genes may positively contribute to salt tolerance regulation and represent one of the core TF families responsible for salt stress defense. Likewise, other TF families including AP2/ERF-ERF, MYB, C3H, WRKY, EREBP, B3-ARF, and NAC also showed significant differential expression. These TFs have been reported to be widely distributed across plant species and involved in abiotic stress responses [37,38,39,40]. It is suggested that the above TF families may coordinately participate in the specific regulation of response to salt stress in sunflower.

Interestingly, complex regulatory patterns were observed within these TF families. The same family contains both upregulated and downregulated members (e.g., 19 upregulated and 18 downregulated members in the AP2/ERF-ERF family). This indicates that the response to salt stress in sunflower does not simply involve activating or repressing an entire TF family. Instead, precise regulation of downstream target genes is achieved through the specific expression of distinct family members, thus building an efficient and orderly stress defense network. This complex expression pattern is consistent with the findings of Guo et al. [41] in cotton, who reported that salt-tolerant and salt-sensitive cultivars activate distinct TF subgroups to form specific cultivar differences in response to stress. The differentially expressed TFs identified in this study provide candidate gene resources for investigating the biological functions of specific TF subtypes and the regulatory network under salt stress in sunflower. Our findings suggest a complex transcriptional regulatory network associated with abiotic stress response in sunflower and identify the bHLH, AP2/ERF-ERF, MYB, C3H, WRKY, EREBP, B3-ARF, and NAC families as the most salt-responsive TF families in our dataset.

In this study, we performed a systematic transcriptome analysis to characterize gene expression differences between the salt-tolerant sunflower cultivar P50 and the salt-sensitive cultivar P29 under salt stress, and we identified a large number of candidate genes involved in response to salt stress. Nevertheless, the research has certain limitations. First of all, only mixed samples of root and leaf were used for transcriptome profiling. However, the response mechanisms of plant root and leaf to salt stress may be different. Therefore, tissue-specific transcriptome analyses should be conducted in future investigations. Secondly, our current analysis was confined to the transcriptional level. Future work should integrate proteomic and metabolomic approaches to achieve a comprehensive multi-omics analysis. Additionally, the functional validation of key candidate genes through transgenic or gene-editing techniques is also required to further clarify their precise regulatory mechanisms.

In conclusion, this study constructed a comprehensive transcription map of sunflower response to salt stress and systematically elucidated the molecular mechanisms underlying salt tolerance. The salt-tolerant sunflower cultivar P50 exhibits an efficient salt stress defense system via three core strategies: (i) activating the antioxidant system to rapidly scavenge excess ROS and mitigate oxidative damage; (ii) regulating carbohydrate metabolism through starch and sucrose redistribution to provide energy and osmotic protection against physiological drought; and (iii) mobilizing multiple TF families to establish a complex regulatory network for the precise control of downstream functional genes.

## Figures and Tables

**Table 1:** Primer sequences of the real-time fluorescence quantitative PCR.

Primer Name	Forward Primer (5′ → 3′)	Reverse Primer (5′ → 3′)
CL10185.Contig2_All	TCGCTGTTGCTACTCGCAATC	CGTACAGTGAGATAGTCCGA
CL73.Contig1_All	GCAGGCGTTGGAGTGCTACA	AATGGGCCAGCAGCCAAGAT
Unigene30614_All	ATCTATCCATCACCAATCTTG	TGCACACAAGGTGTTCGATG
CL8423.Contig1_All	ACTACAGACGACAAAGATGAG	GGCAGAAAAGTAAATACCCAC
CL11319.Contig1_All	TGTGATGGTTTTTTTTAGTGT	TCTTGTTTTTGTGTGTTTTGT
Unigene59384_All	ATGAACACAACAATAAGCAGG	ATACCAAGAAAATATCCGAGG
CL7735.Contig3_All	CCTCGCTATGGCTGCCTCGTT	CTTGGCGGCCTGGTACTCCTG
Unigene32252_All	TTTGTCCCCAAATTCACACAA	CTATCGGAAAACCCTAACGCC
CL12576.Contig1_All	TGAGAAGATCAAGGAGAAGC	AGGAGATCACTGGTGACTTC
CL5734.Contig2_All	ACAGGAAGTGATCTTGTATGC	TGGAATGCTCCACATGCTAG
Unigene12292_All	CAATGTGAGTGGTTAGCTCA	TAGCTTCTCAGATGCATATG
Unigene14805_All	GCAACAGGACCACTCCTTCT	TCATGGGTTTGTCACCTGCTC
Unigene32233_All	TGCAACGATTGGAGTGTTAC	ACCTTGCCGCTTAGAACATG
Unigene551_All	TAAGCGAGCTCGATGGCGATC	TTCACGTAGCTTATTCACCTG
CL10216.Contig2_All	CTCTCTCTGCTCTCTTCTTCA	TACCACAGTCGTCTCCGGCTT
The internal reference gene	GATCGGAGTAATGATTAACAG	TTATGGTTGAGACTAGGACG

**Table 2:** Output statistics of sequencing.

Item	P50	P29
Total raw reads	57,232,364	63,276,298
Total clean reads	54,860,184	60,601,572
Total clean nucleotides (nt)	4,937,416,560	5,454,141,480
Q20 percentage (%)	98.51	98.48
GC percentage (%)	44.92	45.21

**Table 3:** Statistics of assembly quality.

Item	Contig		Unigene		
	P50	P29	P50	P29	All-Unigene
Total number	167,035	191,472	106,275	119,350	110,751
Total length (nt)	59,610,658	66,198,098	76,123,994	81,773,140	96,970,017
Average length (nt)	357	346	716	685	876
N50 (nt)	624	590	1301	1263	1409
Contig number (>600)	5644	5171	—	—	—
Unigene number (>2000)	—	—	1086	1198	1494

**Table 4:** Comparison of the real-time fluorescence quantitative PCR and transcriptome results of differentially expressed genes from sunflower.

Code	Gene ID	Transcriptome Data		RT-qPCR Validation	
		P29_FPKM	P50_FPKM	P29	P50
A	CL10185.Contig2_All	9.23	22.63	1	21.26
B	CL73.Contig1_All	296.60	842.25	1	2.44
C	Unigene30614_All	5.59	10.72	1	1.35
D	CL8423.Contig1_All	3.79	9.94	1	4.40
E	CL11319.Contig1_All	0.44	6.55	1	245.00
F	Unigene59384_All	2.32	9.57	1	10.48
G	CL7735.Contig3_All	2.71	0.15	1	0.17
H	Unigene32252_All	10.25	13.30	1	1.15
I	CL12576.Contig1_All	1243.58	464.27	1	0.36
J	CL5734.Contig2_All	20.66	7.92	1	0.43
K	Unigene12292_All	61.03	61.86	1	0.65
L	Unigene14805_All	42.24	8.71	1	0.19
M	Unigene32233_All	43.73	15.13	1	0.26
N	Unigene551_All	20.03	9.41	1	0.32
O	CL10216.Contig2_All	79.96	33.47	1	0.53

## Data Availability

The original contributions presented in this study are included in the article. Further inquiries can be directed to the corresponding author.

## Supplementary Materials

The following supporting information can be downloaded at: https://www.mdpi.com/article/10.3390/genes17060629/s1. Table S1. The number and names of genes enriched in each molecular function for P29-vs-P50; Figure S1: KEGG pathway enrichment. Figure S2: The distribution of transcription factors in gene families. (A) The distribution of transcription factors according to the gene family information; (B) differentially expressed genes from every gene family involved in transcription.

## Abbreviations

The following abbreviations are used in this manuscript:

DEGs	Differentially expressed genes
RNA-seq	RNA sequencing
GO	Gene Ontology
KEGG	Kyoto Encyclopedia of Genes and Genomes
TFs	Transcription factors
NR	Non-Redundant Protein Sequence Database
COG	Clusters of Orthologous Groups
CDS	Coding sequences
ROS	Reactive oxygen species
