# Supplementary material for: Transcriptomic Analysis Based on RNA-Seq Technology Reveals the Molecular Mechanisms of Sunflower (Helianthus annuus L.) Response to Salt Stress
Source: Genes (Basel). 2026 May 30;17(6):629. doi: 10.3390/genes17060629 (PMC13298483; doi:10.3390/genes17060629)
Supplement: Supplementary file 1 [file genes-17-00629-s001.zip › Table S1..html]

Terms for P29-vs-P50\_F


## Terms for P29-vs-P50\_F

---


### Result Table

|  |
| --- |
| **Terms from the Function Ontology with p-value as good or better than 1** |

| Gene Ontology term | Cluster frequency | Genome frequency of use | Corrected P-value | Expression Profile |
| --- | --- | --- | --- | --- |
| structural molecule activity | 662 out of 7474 genes, 8.9% | 2566 out of 40099 genes, 6.4% | 1.76e-17 | View Result |
| structural constituent of ribosome | 579 out of 7474 genes, 7.7% | 2232 out of 40099 genes, 5.6% | 1.69e-15 | View Result |
| L-ascorbate oxidase activity | 41 out of 7474 genes, 0.5% | 76 out of 40099 genes, 0.2% | 7.52e-09 | View Result |
| oxidoreductase activity, acting on diphenols and related substances as donors, oxygen as acceptor | 44 out of 7474 genes, 0.6% | 88 out of 40099 genes, 0.2% | 3.80e-08 | View Result |
| oxidoreductase activity | 1140 out of 7474 genes, 15.3% | 5240 out of 40099 genes, 13.1% | 7.32e-07 | View Result |
| protein heterodimerization activity | 103 out of 7474 genes, 1.4% | 324 out of 40099 genes, 0.8% | 1.14e-05 | View Result |
| structural constituent of cytoskeleton | 60 out of 7474 genes, 0.8% | 163 out of 40099 genes, 0.4% | 4.82e-05 | View Result |
| hydroquinone:oxygen oxidoreductase activity | 21 out of 7474 genes, 0.3% | 35 out of 40099 genes, 0.1% | 9.38e-05 | View Result |
| oxidoreductase activity, acting on diphenols and related substances as donors | 47 out of 7474 genes, 0.6% | 128 out of 40099 genes, 0.3% | 0.00149 | View Result |
| peroxidase activity | 86 out of 7474 genes, 1.2% | 284 out of 40099 genes, 0.7% | 0.00182 | View Result |
| oxidoreductase activity, acting on peroxide as acceptor | 86 out of 7474 genes, 1.2% | 284 out of 40099 genes, 0.7% | 0.00182 | View Result |
| hydrogen-translocating pyrophosphatase activity | 22 out of 7474 genes, 0.3% | 45 out of 40099 genes, 0.1% | 0.00536 | View Result |
| GTPase activity | 162 out of 7474 genes, 2.2% | 628 out of 40099 genes, 1.6% | 0.00677 | View Result |
| antioxidant activity | 99 out of 7474 genes, 1.3% | 353 out of 40099 genes, 0.9% | 0.01291 | View Result |
| aspartyl esterase activity | 20 out of 7474 genes, 0.3% | 42 out of 40099 genes, 0.1% | 0.02407 | View Result |
| protein dimerization activity | 208 out of 7474 genes, 2.8% | 860 out of 40099 genes, 2.1% | 0.03384 | View Result |
| beta-glucosidase activity | 40 out of 7474 genes, 0.5% | 118 out of 40099 genes, 0.3% | 0.07684 | View Result |
| cellulase activity | 23 out of 7474 genes, 0.3% | 56 out of 40099 genes, 0.1% | 0.10960 | View Result |
| pectinesterase activity | 25 out of 7474 genes, 0.3% | 64 out of 40099 genes, 0.2% | 0.14550 | View Result |
| tetrapyrrole binding | 147 out of 7474 genes, 2.0% | 601 out of 40099 genes, 1.5% | 0.28084 | View Result |
| FMN reductase activity | 5 out of 7474 genes, 0.1% | 5 out of 40099 genes, 0.0% | 0.29796 | View Result |
| glucan endo-1,3-beta-D-glucosidase activity | 21 out of 7474 genes, 0.3% | 53 out of 40099 genes, 0.1% | 0.39984 | View Result |
| nutrient reservoir activity | 22 out of 7474 genes, 0.3% | 57 out of 40099 genes, 0.1% | 0.45009 | View Result |
| glucosidase activity | 45 out of 7474 genes, 0.6% | 149 out of 40099 genes, 0.4% | 0.57290 | View Result |
| heme binding | 136 out of 7474 genes, 1.8% | 560 out of 40099 genes, 1.4% | 0.64456 | View Result |
| glutamate synthase activity, NAD(P)H as acceptor | 7 out of 7474 genes, 0.1% | 10 out of 40099 genes, 0.0% | 0.72892 | View Result |
| NADP+ binding | 7 out of 7474 genes, 0.1% | 10 out of 40099 genes, 0.0% | 0.72892 | View Result |
| L-tyrosine aminotransferase activity | 7 out of 7474 genes, 0.1% | 10 out of 40099 genes, 0.0% | 0.72892 | View Result |
| GTP binding | 213 out of 7474 genes, 2.8% | 932 out of 40099 genes, 2.3% | 0.83432 | View Result |
| guanyl ribonucleotide binding | 213 out of 7474 genes, 2.8% | 932 out of 40099 genes, 2.3% | 0.83432 | View Result |
| enzyme inhibitor activity | 35 out of 7474 genes, 0.5% | 110 out of 40099 genes, 0.3% | 0.83657 | View Result |
| oxidoreductase activity, acting on the CH-OH group of donors, NAD or NADP as acceptor | 160 out of 7474 genes, 2.1% | 679 out of 40099 genes, 1.7% | 0.95127 | View Result |
| nicotianamine synthase activity | 6 out of 7474 genes, 0.1% | 8 out of 40099 genes, 0.0% | 1 | View Result |
| ribonucleoside-diphosphate reductase activity, thioredoxin disulfide as acceptor | 10 out of 7474 genes, 0.1% | 19 out of 40099 genes, 0.0% | 1 | View Result |
| oxidoreductase activity, acting on CH or CH2 groups, disulfide as acceptor | 10 out of 7474 genes, 0.1% | 19 out of 40099 genes, 0.0% | 1 | View Result |
| guanyl nucleotide binding | 213 out of 7474 genes, 2.8% | 939 out of 40099 genes, 2.3% | 1 | View Result |
| hydrolase activity, hydrolyzing O-glycosyl compounds | 185 out of 7474 genes, 2.5% | 804 out of 40099 genes, 2.0% | 1 | View Result |
| inositol 3-alpha-galactosyltransferase activity | 5 out of 7474 genes, 0.1% | 6 out of 40099 genes, 0.0% | 1 | View Result |
| integrase activity | 4 out of 7474 genes, 0.1% | 4 out of 40099 genes, 0.0% | 1 | View Result |
| glutamate synthase activity | 7 out of 7474 genes, 0.1% | 11 out of 40099 genes, 0.0% | 1 | View Result |
| alcohol dehydrogenase (NADP+) activity | 11 out of 7474 genes, 0.1% | 23 out of 40099 genes, 0.1% | 1 | View Result |
| aldo-keto reductase (NADP) activity | 20 out of 7474 genes, 0.3% | 55 out of 40099 genes, 0.1% | 1 | View Result |
| L-phenylalanine aminotransferase activity | 17 out of 7474 genes, 0.2% | 45 out of 40099 genes, 0.1% | 1 | View Result |
| L-tyrosine:2-oxoglutarate aminotransferase activity | 6 out of 7474 genes, 0.1% | 9 out of 40099 genes, 0.0% | 1 | View Result |
| protein C-terminus binding | 6 out of 7474 genes, 0.1% | 9 out of 40099 genes, 0.0% | 1 | View Result |
| glutamate synthase (NADH) activity | 6 out of 7474 genes, 0.1% | 9 out of 40099 genes, 0.0% | 1 | View Result |
| nicotianamine aminotransferase activity | 6 out of 7474 genes, 0.1% | 9 out of 40099 genes, 0.0% | 1 | View Result |
| inositol oxygenase activity | 6 out of 7474 genes, 0.1% | 9 out of 40099 genes, 0.0% | 1 | View Result |
| copper ion binding | 191 out of 7474 genes, 2.6% | 847 out of 40099 genes, 2.1% | 1 | View Result |
| primary amine oxidase activity | 10 out of 7474 genes, 0.1% | 21 out of 40099 genes, 0.1% | 1 | View Result |
| mannan synthase activity | 10 out of 7474 genes, 0.1% | 21 out of 40099 genes, 0.1% | 1 | View Result |
| oxygen binding | 28 out of 7474 genes, 0.4% | 89 out of 40099 genes, 0.2% | 1 | View Result |
| intramolecular oxidoreductase activity, transposing C=C bonds | 7 out of 7474 genes, 0.1% | 12 out of 40099 genes, 0.0% | 1 | View Result |
| glutamate-ammonia ligase activity | 12 out of 7474 genes, 0.2% | 28 out of 40099 genes, 0.1% | 1 | View Result |
| beta-1,4-mannosyltransferase activity | 10 out of 7474 genes, 0.1% | 22 out of 40099 genes, 0.1% | 1 | View Result |
| L-phenylalanine:2-oxoglutarate aminotransferase activity | 16 out of 7474 genes, 0.2% | 44 out of 40099 genes, 0.1% | 1 | View Result |
| transaminase activity | 48 out of 7474 genes, 0.6% | 179 out of 40099 genes, 0.4% | 1 | View Result |
| transferase activity, transferring nitrogenous groups | 48 out of 7474 genes, 0.6% | 179 out of 40099 genes, 0.4% | 1 | View Result |
| drug transmembrane transporter activity | 49 out of 7474 genes, 0.7% | 184 out of 40099 genes, 0.5% | 1 | View Result |
| oxidoreductase activity, acting on paired donors, with incorporation or reduction of molecular oxygen, NAD(P)H as one donor, and incorporation of one atom of oxygen | 53 out of 7474 genes, 0.7% | 202 out of 40099 genes, 0.5% | 1 | View Result |
| dodecenoyl-CoA delta-isomerase activity | 4 out of 7474 genes, 0.1% | 5 out of 40099 genes, 0.0% | 1 | View Result |
| intramolecular transferase activity, transferring amino groups | 4 out of 7474 genes, 0.1% | 5 out of 40099 genes, 0.0% | 1 | View Result |
| histone serine kinase activity | 4 out of 7474 genes, 0.1% | 5 out of 40099 genes, 0.0% | 1 | View Result |
| histone kinase activity (H3-S10 specific) | 4 out of 7474 genes, 0.1% | 5 out of 40099 genes, 0.0% | 1 | View Result |
| glutamate-1-semialdehyde 2,1-aminomutase activity | 4 out of 7474 genes, 0.1% | 5 out of 40099 genes, 0.0% | 1 | View Result |
| geranylgeranyl reductase activity | 4 out of 7474 genes, 0.1% | 5 out of 40099 genes, 0.0% | 1 | View Result |
| oxidoreductase activity, acting on CH-OH group of donors | 187 out of 7474 genes, 2.5% | 844 out of 40099 genes, 2.1% | 1 | View Result |
| isocitrate dehydrogenase (NADP+) activity | 10 out of 7474 genes, 0.1% | 23 out of 40099 genes, 0.1% | 1 | View Result |
| ammonia ligase activity | 13 out of 7474 genes, 0.2% | 34 out of 40099 genes, 0.1% | 1 | View Result |
| acid-ammonia (or amide) ligase activity | 13 out of 7474 genes, 0.2% | 34 out of 40099 genes, 0.1% | 1 | View Result |
| (1->3)-beta-D-glucan binding | 3 out of 7474 genes, 0.0% | 3 out of 40099 genes, 0.0% | 1 |  |
| methylglutaconyl-CoA hydratase activity | 3 out of 7474 genes, 0.0% | 3 out of 40099 genes, 0.0% | 1 | View Result |
| stearoyl-CoA 9-desaturase activity | 3 out of 7474 genes, 0.0% | 3 out of 40099 genes, 0.0% | 1 | View Result |
| CMP-N-acetylneuraminate transmembrane transporter activity | 3 out of 7474 genes, 0.0% | 3 out of 40099 genes, 0.0% | 1 | View Result |
| sialic acid transmembrane transporter activity | 3 out of 7474 genes, 0.0% | 3 out of 40099 genes, 0.0% | 1 | View Result |
| acyl-CoA desaturase activity | 3 out of 7474 genes, 0.0% | 3 out of 40099 genes, 0.0% | 1 | View Result |
| indoleacetamide hydrolase activity | 3 out of 7474 genes, 0.0% | 3 out of 40099 genes, 0.0% | 1 | View Result |
| 3-oxoacyl-[acyl-carrier-protein] reductase (NADPH) activity | 8 out of 7474 genes, 0.1% | 17 out of 40099 genes, 0.0% | 1 | View Result |
| oxidoreductase activity, acting on the CH-NH2 group of donors, NAD or NADP as acceptor | 8 out of 7474 genes, 0.1% | 17 out of 40099 genes, 0.0% | 1 | View Result |
| hydrolase activity, acting on glycosyl bonds | 203 out of 7474 genes, 2.7% | 930 out of 40099 genes, 2.3% | 1 | View Result |
| aspartic-type peptidase activity | 30 out of 7474 genes, 0.4% | 104 out of 40099 genes, 0.3% | 1 | View Result |
| translation elongation factor activity | 64 out of 7474 genes, 0.9% | 257 out of 40099 genes, 0.6% | 1 | View Result |
| chitinase activity | 10 out of 7474 genes, 0.1% | 24 out of 40099 genes, 0.1% | 1 | View Result |
| rRNA binding | 60 out of 7474 genes, 0.8% | 239 out of 40099 genes, 0.6% | 1 | View Result |
| oxygen transporter activity | 6 out of 7474 genes, 0.1% | 11 out of 40099 genes, 0.0% | 1 | View Result |
| anion:cation symporter activity | 6 out of 7474 genes, 0.1% | 11 out of 40099 genes, 0.0% | 1 | View Result |
| secondary active transmembrane transporter activity | 140 out of 7474 genes, 1.9% | 623 out of 40099 genes, 1.6% | 1 | View Result |
| xyloglucan:xyloglucosyl transferase activity | 17 out of 7474 genes, 0.2% | 51 out of 40099 genes, 0.1% | 1 | View Result |
| drug transporter activity | 49 out of 7474 genes, 0.7% | 190 out of 40099 genes, 0.5% | 1 | View Result |
| oxidoreductase activity, acting on the CH-CH group of donors, NAD or NADP as acceptor | 176 out of 7474 genes, 2.4% | 801 out of 40099 genes, 2.0% | 1 | View Result |
| galactosyltransferase activity | 24 out of 7474 genes, 0.3% | 80 out of 40099 genes, 0.2% | 1 | View Result |
| isocitrate dehydrogenase activity | 13 out of 7474 genes, 0.2% | 36 out of 40099 genes, 0.1% | 1 | View Result |
| NADPH:quinone reductase activity | 10 out of 7474 genes, 0.1% | 25 out of 40099 genes, 0.1% | 1 | View Result |
| oxidoreductase activity, acting on the CH-CH group of donors | 209 out of 7474 genes, 2.8% | 970 out of 40099 genes, 2.4% | 1 | View Result |
| carboxylic acid binding | 50 out of 7474 genes, 0.7% | 197 out of 40099 genes, 0.5% | 1 | View Result |
| oxidoreductase activity, acting on paired donors, with incorporation or reduction of molecular oxygen | 167 out of 7474 genes, 2.2% | 762 out of 40099 genes, 1.9% | 1 | View Result |
| carbohydrate derivative binding | 7 out of 7474 genes, 0.1% | 15 out of 40099 genes, 0.0% | 1 | View Result |
| carboxylesterase activity | 35 out of 7474 genes, 0.5% | 130 out of 40099 genes, 0.3% | 1 | View Result |
| peroxiredoxin activity | 9 out of 7474 genes, 0.1% | 22 out of 40099 genes, 0.1% | 1 | View Result |
| carotenoid isomerase activity | 4 out of 7474 genes, 0.1% | 6 out of 40099 genes, 0.0% | 1 | View Result |
| L-leucine transaminase activity | 4 out of 7474 genes, 0.1% | 6 out of 40099 genes, 0.0% | 1 | View Result |
| L-valine transaminase activity | 4 out of 7474 genes, 0.1% | 6 out of 40099 genes, 0.0% | 1 | View Result |
| L-isoleucine transaminase activity | 4 out of 7474 genes, 0.1% | 6 out of 40099 genes, 0.0% | 1 | View Result |
| phosphoserine phosphatase activity | 6 out of 7474 genes, 0.1% | 12 out of 40099 genes, 0.0% | 1 | View Result |
| choline dehydrogenase activity | 6 out of 7474 genes, 0.1% | 12 out of 40099 genes, 0.0% | 1 | View Result |
| DNA polymerase processivity factor activity | 6 out of 7474 genes, 0.1% | 12 out of 40099 genes, 0.0% | 1 | View Result |
| aspartic-type endopeptidase activity | 28 out of 7474 genes, 0.4% | 100 out of 40099 genes, 0.2% | 1 | View Result |
| NADPH dehydrogenase activity | 5 out of 7474 genes, 0.1% | 9 out of 40099 genes, 0.0% | 1 | View Result |
| ribose-5-phosphate isomerase activity | 5 out of 7474 genes, 0.1% | 9 out of 40099 genes, 0.0% | 1 | View Result |
| NADP binding | 39 out of 7474 genes, 0.5% | 150 out of 40099 genes, 0.4% | 1 | View Result |
| galacturan 1,4-alpha-galacturonidase activity | 7 out of 7474 genes, 0.1% | 16 out of 40099 genes, 0.0% | 1 | View Result |
| inorganic diphosphatase activity | 17 out of 7474 genes, 0.2% | 55 out of 40099 genes, 0.1% | 1 | View Result |
| cinnamyl-alcohol dehydrogenase activity | 8 out of 7474 genes, 0.1% | 20 out of 40099 genes, 0.0% | 1 | View Result |
| minus-end-directed microtubule motor activity | 6 out of 7474 genes, 0.1% | 13 out of 40099 genes, 0.0% | 1 | View Result |
| mandelonitrile lyase activity | 6 out of 7474 genes, 0.1% | 13 out of 40099 genes, 0.0% | 1 | View Result |
| carnitine racemase activity | 3 out of 7474 genes, 0.0% | 4 out of 40099 genes, 0.0% | 1 | View Result |
| nicotinamidase activity | 3 out of 7474 genes, 0.0% | 4 out of 40099 genes, 0.0% | 1 | View Result |
| octanoyltransferase activity | 3 out of 7474 genes, 0.0% | 4 out of 40099 genes, 0.0% | 1 | View Result |
| lipoyltransferase activity | 3 out of 7474 genes, 0.0% | 4 out of 40099 genes, 0.0% | 1 | View Result |
| S-formylglutathione hydrolase activity | 3 out of 7474 genes, 0.0% | 4 out of 40099 genes, 0.0% | 1 | View Result |
| sinapyl alcohol dehydrogenase activity | 3 out of 7474 genes, 0.0% | 4 out of 40099 genes, 0.0% | 1 | View Result |
| monooxygenase activity | 114 out of 7474 genes, 1.5% | 514 out of 40099 genes, 1.3% | 1 | View Result |
| RNA binding | 496 out of 7474 genes, 6.6% | 2461 out of 40099 genes, 6.1% | 1 | View Result |
| aryl-alcohol dehydrogenase (NAD+) activity | 4 out of 7474 genes, 0.1% | 7 out of 40099 genes, 0.0% | 1 | View Result |
| manganese ion binding | 24 out of 7474 genes, 0.3% | 87 out of 40099 genes, 0.2% | 1 | View Result |
| carbon-sulfur lyase activity | 23 out of 7474 genes, 0.3% | 83 out of 40099 genes, 0.2% | 1 | View Result |
| sodium ion transmembrane transporter activity | 18 out of 7474 genes, 0.2% | 62 out of 40099 genes, 0.2% | 1 | View Result |
| procollagen-proline 4-dioxygenase activity | 6 out of 7474 genes, 0.1% | 14 out of 40099 genes, 0.0% | 1 | View Result |
| chitin binding | 6 out of 7474 genes, 0.1% | 14 out of 40099 genes, 0.0% | 1 | View Result |
| procollagen-proline dioxygenase activity | 6 out of 7474 genes, 0.1% | 14 out of 40099 genes, 0.0% | 1 | View Result |
| peptidyl-proline dioxygenase activity | 6 out of 7474 genes, 0.1% | 14 out of 40099 genes, 0.0% | 1 | View Result |
| peptidyl-proline 4-dioxygenase activity | 6 out of 7474 genes, 0.1% | 14 out of 40099 genes, 0.0% | 1 | View Result |
| coenzyme binding | 196 out of 7474 genes, 2.6% | 932 out of 40099 genes, 2.3% | 1 | View Result |
| oxidoreductase activity, acting on the CH-NH2 group of donors | 24 out of 7474 genes, 0.3% | 89 out of 40099 genes, 0.2% | 1 | View Result |
| auxin transmembrane transporter activity | 26 out of 7474 genes, 0.3% | 98 out of 40099 genes, 0.2% | 1 | View Result |
| IkappaB kinase activity | 2 out of 7474 genes, 0.0% | 2 out of 40099 genes, 0.0% | 1 | View Result |
| 1-aminocyclopropane-1-carboxylate deaminase activity | 2 out of 7474 genes, 0.0% | 2 out of 40099 genes, 0.0% | 1 | View Result |
| DNA-3-methyladenine glycosylase activity | 2 out of 7474 genes, 0.0% | 2 out of 40099 genes, 0.0% | 1 | View Result |
| isochorismatase activity | 2 out of 7474 genes, 0.0% | 2 out of 40099 genes, 0.0% | 1 | View Result |
| UDP-galactose:N-glycan beta-1,3-galactosyltransferase activity | 2 out of 7474 genes, 0.0% | 2 out of 40099 genes, 0.0% | 1 | View Result |
| 15-hydroxyprostaglandin dehydrogenase (NAD+) activity | 2 out of 7474 genes, 0.0% | 2 out of 40099 genes, 0.0% | 1 | View Result |
| D-cysteine desulfhydrase activity | 2 out of 7474 genes, 0.0% | 2 out of 40099 genes, 0.0% | 1 | View Result |
| DNA-3-methylbase glycosylase activity | 2 out of 7474 genes, 0.0% | 2 out of 40099 genes, 0.0% | 1 | View Result |
| phosphosulfolactate synthase activity | 2 out of 7474 genes, 0.0% | 2 out of 40099 genes, 0.0% | 1 | View Result |
| p-coumarate 3-hydroxylase activity | 2 out of 7474 genes, 0.0% | 2 out of 40099 genes, 0.0% | 1 | View Result |
| histone methyltransferase activity (H3-K36 specific) | 2 out of 7474 genes, 0.0% | 2 out of 40099 genes, 0.0% | 1 | View Result |
| beta-pyrazolylalanine synthase activity | 2 out of 7474 genes, 0.0% | 2 out of 40099 genes, 0.0% | 1 | View Result |
| 9,9'-dicis-carotene:quinone oxidoreductase activity | 2 out of 7474 genes, 0.0% | 2 out of 40099 genes, 0.0% | 1 | View Result |
| 7,9,9'-tricis-neurosporene:quinone oxidoreductase activity | 2 out of 7474 genes, 0.0% | 2 out of 40099 genes, 0.0% | 1 | View Result |
| brassinosteroid sulfotransferase activity | 2 out of 7474 genes, 0.0% | 2 out of 40099 genes, 0.0% | 1 | View Result |
| branched-chain-amino-acid transaminase activity | 7 out of 7474 genes, 0.1% | 18 out of 40099 genes, 0.0% | 1 | View Result |
| carboxylic ester hydrolase activity | 92 out of 7474 genes, 1.2% | 414 out of 40099 genes, 1.0% | 1 | View Result |
| auxin efflux transmembrane transporter activity | 20 out of 7474 genes, 0.3% | 72 out of 40099 genes, 0.2% | 1 | View Result |
| 3-isopropylmalate dehydratase activity | 5 out of 7474 genes, 0.1% | 11 out of 40099 genes, 0.0% | 1 | View Result |
| UDP-galactosyltransferase activity | 16 out of 7474 genes, 0.2% | 55 out of 40099 genes, 0.1% | 1 | View Result |
| Ras guanyl-nucleotide exchange factor activity | 9 out of 7474 genes, 0.1% | 26 out of 40099 genes, 0.1% | 1 | View Result |
| Rho guanyl-nucleotide exchange factor activity | 9 out of 7474 genes, 0.1% | 26 out of 40099 genes, 0.1% | 1 | View Result |
| 2-alkenal reductase [NAD(P)] activity | 147 out of 7474 genes, 2.0% | 690 out of 40099 genes, 1.7% | 1 | View Result |
| intramolecular oxidoreductase activity | 25 out of 7474 genes, 0.3% | 95 out of 40099 genes, 0.2% | 1 | View Result |
| carbohydrate transmembrane transporter activity | 49 out of 7474 genes, 0.7% | 207 out of 40099 genes, 0.5% | 1 | View Result |
| carbohydrate transporter activity | 49 out of 7474 genes, 0.7% | 207 out of 40099 genes, 0.5% | 1 | View Result |
| aldehyde-lyase activity | 15 out of 7474 genes, 0.2% | 51 out of 40099 genes, 0.1% | 1 | View Result |
| 1-phosphatidylinositol binding | 6 out of 7474 genes, 0.1% | 15 out of 40099 genes, 0.0% | 1 | View Result |
| amino acid binding | 36 out of 7474 genes, 0.5% | 147 out of 40099 genes, 0.4% | 1 | View Result |
| oxidoreductase activity, acting on the CH-NH group of donors, NAD or NADP as acceptor | 12 out of 7474 genes, 0.2% | 39 out of 40099 genes, 0.1% | 1 | View Result |
| NAD(P)H dehydrogenase (quinone) activity | 3 out of 7474 genes, 0.0% | 5 out of 40099 genes, 0.0% | 1 | View Result |
| fatty acid binding | 3 out of 7474 genes, 0.0% | 5 out of 40099 genes, 0.0% | 1 | View Result |
| sodium:dicarboxylate symporter activity | 3 out of 7474 genes, 0.0% | 5 out of 40099 genes, 0.0% | 1 | View Result |
| allene-oxide cyclase activity | 3 out of 7474 genes, 0.0% | 5 out of 40099 genes, 0.0% | 1 | View Result |
| 4,4-dimethyl-9beta,19-cyclopropylsterol-4alpha-methyl oxidase activity | 3 out of 7474 genes, 0.0% | 5 out of 40099 genes, 0.0% | 1 | View Result |
| antiporter activity | 62 out of 7474 genes, 0.8% | 272 out of 40099 genes, 0.7% | 1 | View Result |
| squalene monooxygenase activity | 7 out of 7474 genes, 0.1% | 19 out of 40099 genes, 0.0% | 1 | View Result |
| acetate-CoA ligase activity | 8 out of 7474 genes, 0.1% | 23 out of 40099 genes, 0.1% | 1 | View Result |
| citrate (Si)-synthase activity | 9 out of 7474 genes, 0.1% | 27 out of 40099 genes, 0.1% | 1 | View Result |
| galactosylxylosylprotein 3-beta-galactosyltransferase activity | 9 out of 7474 genes, 0.1% | 27 out of 40099 genes, 0.1% | 1 | View Result |
| mannosyltransferase activity | 14 out of 7474 genes, 0.2% | 48 out of 40099 genes, 0.1% | 1 | View Result |
| cofactor binding | 265 out of 7474 genes, 3.5% | 1298 out of 40099 genes, 3.2% | 1 | View Result |
| amide transmembrane transporter activity | 18 out of 7474 genes, 0.2% | 66 out of 40099 genes, 0.2% | 1 | View Result |
| microtubule binding | 44 out of 7474 genes, 0.6% | 187 out of 40099 genes, 0.5% | 1 | View Result |
| poly-pyrimidine tract binding | 22 out of 7474 genes, 0.3% | 84 out of 40099 genes, 0.2% | 1 | View Result |
| poly(U) RNA binding | 22 out of 7474 genes, 0.3% | 84 out of 40099 genes, 0.2% | 1 | View Result |
| anthranilate synthase activity | 5 out of 7474 genes, 0.1% | 12 out of 40099 genes, 0.0% | 1 | View Result |
| UDP-N-acetylmuramate dehydrogenase activity | 14 out of 7474 genes, 0.2% | 49 out of 40099 genes, 0.1% | 1 | View Result |
| cadmium ion transmembrane transporter activity | 10 out of 7474 genes, 0.1% | 32 out of 40099 genes, 0.1% | 1 | View Result |
| isomerase activity | 155 out of 7474 genes, 2.1% | 741 out of 40099 genes, 1.8% | 1 | View Result |
| peptidase inhibitor activity | 9 out of 7474 genes, 0.1% | 28 out of 40099 genes, 0.1% | 1 | View Result |
| peptidase regulator activity | 9 out of 7474 genes, 0.1% | 28 out of 40099 genes, 0.1% | 1 | View Result |
| alcohol dehydrogenase (NAD) activity | 7 out of 7474 genes, 0.1% | 20 out of 40099 genes, 0.0% | 1 | View Result |
| sodium:hydrogen antiporter activity | 7 out of 7474 genes, 0.1% | 20 out of 40099 genes, 0.0% | 1 | View Result |
| ATP:ADP antiporter activity | 8 out of 7474 genes, 0.1% | 24 out of 40099 genes, 0.1% | 1 | View Result |
| sulfotransferase activity | 8 out of 7474 genes, 0.1% | 24 out of 40099 genes, 0.1% | 1 | View Result |
| urea transmembrane transporter activity | 8 out of 7474 genes, 0.1% | 24 out of 40099 genes, 0.1% | 1 | View Result |
| transferase activity, transferring hexosyl groups | 227 out of 7474 genes, 3.0% | 1110 out of 40099 genes, 2.8% | 1 | View Result |
| phosphoribosylformylglycinamidine cyclo-ligase activity | 4 out of 7474 genes, 0.1% | 9 out of 40099 genes, 0.0% | 1 | View Result |
| G-protein coupled receptor kinase activity | 4 out of 7474 genes, 0.1% | 9 out of 40099 genes, 0.0% | 1 | View Result |
| phospholipase activator activity | 4 out of 7474 genes, 0.1% | 9 out of 40099 genes, 0.0% | 1 | View Result |
| cinnamoyl-CoA reductase activity | 4 out of 7474 genes, 0.1% | 9 out of 40099 genes, 0.0% | 1 | View Result |
| aspartate-prephenate aminotransferase activity | 4 out of 7474 genes, 0.1% | 9 out of 40099 genes, 0.0% | 1 | View Result |
| glutamate-prephenate aminotransferase activity | 4 out of 7474 genes, 0.1% | 9 out of 40099 genes, 0.0% | 1 | View Result |
| omega-3 fatty acid desaturase activity | 4 out of 7474 genes, 0.1% | 9 out of 40099 genes, 0.0% | 1 | View Result |
| lipase activator activity | 4 out of 7474 genes, 0.1% | 9 out of 40099 genes, 0.0% | 1 | View Result |
| 3R-hydroxyacyl-CoA dehydratase activity | 4 out of 7474 genes, 0.1% | 9 out of 40099 genes, 0.0% | 1 | View Result |
| polygalacturonase activity | 14 out of 7474 genes, 0.2% | 50 out of 40099 genes, 0.1% | 1 | View Result |
| organic acid:sodium symporter activity | 10 out of 7474 genes, 0.1% | 33 out of 40099 genes, 0.1% | 1 | View Result |
| active transmembrane transporter activity | 326 out of 7474 genes, 4.4% | 1627 out of 40099 genes, 4.1% | 1 | View Result |
| oxo-acid-lyase activity | 15 out of 7474 genes, 0.2% | 55 out of 40099 genes, 0.1% | 1 | View Result |
| UDP-glucose 4-epimerase activity | 9 out of 7474 genes, 0.1% | 29 out of 40099 genes, 0.1% | 1 | View Result |
| protochlorophyllide reductase activity | 9 out of 7474 genes, 0.1% | 29 out of 40099 genes, 0.1% | 1 | View Result |
| carbamoyl-phosphate synthase (glutamine-hydrolyzing) activity | 5 out of 7474 genes, 0.1% | 13 out of 40099 genes, 0.0% | 1 | View Result |
| naringenin 3-dioxygenase activity | 5 out of 7474 genes, 0.1% | 13 out of 40099 genes, 0.0% | 1 | View Result |
| solute:cation symporter activity | 50 out of 7474 genes, 0.7% | 221 out of 40099 genes, 0.6% | 1 | View Result |
| methyl indole-3-acetate esterase activity | 8 out of 7474 genes, 0.1% | 25 out of 40099 genes, 0.1% | 1 | View Result |
| protease binding | 6 out of 7474 genes, 0.1% | 17 out of 40099 genes, 0.0% | 1 | View Result |
| catalase activity | 16 out of 7474 genes, 0.2% | 60 out of 40099 genes, 0.1% | 1 | View Result |
| chlorophyll binding | 11 out of 7474 genes, 0.1% | 38 out of 40099 genes, 0.1% | 1 | View Result |
| 3-dehydroquinate dehydratase activity | 3 out of 7474 genes, 0.0% | 6 out of 40099 genes, 0.0% | 1 | View Result |
| adenylylsulfate kinase activity | 3 out of 7474 genes, 0.0% | 6 out of 40099 genes, 0.0% | 1 | View Result |
| fumarate hydratase activity | 3 out of 7474 genes, 0.0% | 6 out of 40099 genes, 0.0% | 1 | View Result |
| isocitrate lyase activity | 3 out of 7474 genes, 0.0% | 6 out of 40099 genes, 0.0% | 1 | View Result |
| eukaryotic translation initiation factor 2alpha kinase activity | 3 out of 7474 genes, 0.0% | 6 out of 40099 genes, 0.0% | 1 | View Result |
| shikimate 3-dehydrogenase (NADP+) activity | 3 out of 7474 genes, 0.0% | 6 out of 40099 genes, 0.0% | 1 | View Result |
| palmitoyl-(protein) hydrolase activity | 3 out of 7474 genes, 0.0% | 6 out of 40099 genes, 0.0% | 1 | View Result |
| auxin influx transmembrane transporter activity | 3 out of 7474 genes, 0.0% | 6 out of 40099 genes, 0.0% | 1 | View Result |
| lupeol synthase activity | 3 out of 7474 genes, 0.0% | 6 out of 40099 genes, 0.0% | 1 | View Result |
| pyruvate, phosphate dikinase activity | 3 out of 7474 genes, 0.0% | 6 out of 40099 genes, 0.0% | 1 | View Result |
| tubulin binding | 47 out of 7474 genes, 0.6% | 208 out of 40099 genes, 0.5% | 1 | View Result |
| solute:sodium symporter activity | 10 out of 7474 genes, 0.1% | 34 out of 40099 genes, 0.1% | 1 | View Result |
| symporter activity | 53 out of 7474 genes, 0.7% | 238 out of 40099 genes, 0.6% | 1 | View Result |
| oxidoreductase activity, acting on the CH-NH group of donors | 18 out of 7474 genes, 0.2% | 70 out of 40099 genes, 0.2% | 1 | View Result |
| ADP binding | 18 out of 7474 genes, 0.2% | 70 out of 40099 genes, 0.2% | 1 | View Result |
| intramolecular oxidoreductase activity, interconverting aldoses and ketoses | 12 out of 7474 genes, 0.2% | 43 out of 40099 genes, 0.1% | 1 | View Result |
| nucleoside diphosphate kinase activity | 9 out of 7474 genes, 0.1% | 30 out of 40099 genes, 0.1% | 1 | View Result |
| adenyl-nucleotide exchange factor activity | 2 out of 7474 genes, 0.0% | 3 out of 40099 genes, 0.0% | 1 | View Result |
| DNA (6-4) photolyase activity | 2 out of 7474 genes, 0.0% | 3 out of 40099 genes, 0.0% | 1 | View Result |
| N4-(beta-N-acetylglucosaminyl)-L-asparaginase activity | 2 out of 7474 genes, 0.0% | 3 out of 40099 genes, 0.0% | 1 | View Result |
| aspartate-semialdehyde dehydrogenase activity | 2 out of 7474 genes, 0.0% | 3 out of 40099 genes, 0.0% | 1 | View Result |
| cystathionine beta-lyase activity | 2 out of 7474 genes, 0.0% | 3 out of 40099 genes, 0.0% | 1 | View Result |
| cytidine deaminase activity | 2 out of 7474 genes, 0.0% | 3 out of 40099 genes, 0.0% | 1 | View Result |
| 4-alpha-glucanotransferase activity | 2 out of 7474 genes, 0.0% | 3 out of 40099 genes, 0.0% | 1 | View Result |
| diphosphomevalonate decarboxylase activity | 2 out of 7474 genes, 0.0% | 3 out of 40099 genes, 0.0% | 1 | View Result |
| saccharopine dehydrogenase activity | 2 out of 7474 genes, 0.0% | 3 out of 40099 genes, 0.0% | 1 | View Result |
| phosphatidylinositol-3,4,5-trisphosphate binding | 2 out of 7474 genes, 0.0% | 3 out of 40099 genes, 0.0% | 1 | View Result |
| tRNA guanylyltransferase activity | 2 out of 7474 genes, 0.0% | 3 out of 40099 genes, 0.0% | 1 | View Result |
| red or far-red light photoreceptor activity | 2 out of 7474 genes, 0.0% | 3 out of 40099 genes, 0.0% | 1 | View Result |
| carotene 7,8-desaturase activity | 2 out of 7474 genes, 0.0% | 3 out of 40099 genes, 0.0% | 1 | View Result |
| ubiquitin conjugating enzyme binding | 2 out of 7474 genes, 0.0% | 3 out of 40099 genes, 0.0% | 1 | View Result |
| aldehyde dehydrogenase (NADP+) activity | 2 out of 7474 genes, 0.0% | 3 out of 40099 genes, 0.0% | 1 | View Result |
| ribonuclease T2 activity | 2 out of 7474 genes, 0.0% | 3 out of 40099 genes, 0.0% | 1 | View Result |
| annealing helicase activity | 2 out of 7474 genes, 0.0% | 3 out of 40099 genes, 0.0% | 1 | View Result |
| histone kinase activity (H3-S28 specific) | 2 out of 7474 genes, 0.0% | 3 out of 40099 genes, 0.0% | 1 | View Result |
| protein binding involved in protein folding | 2 out of 7474 genes, 0.0% | 3 out of 40099 genes, 0.0% | 1 | View Result |
| small protein conjugating enzyme binding | 2 out of 7474 genes, 0.0% | 3 out of 40099 genes, 0.0% | 1 | View Result |
| acyl-[acyl-carrier-protein] desaturase activity | 2 out of 7474 genes, 0.0% | 3 out of 40099 genes, 0.0% | 1 | View Result |
| delta3,5-delta2,4-dienoyl-CoA isomerase activity | 2 out of 7474 genes, 0.0% | 3 out of 40099 genes, 0.0% | 1 | View Result |
| mitochondrial light strand promoter anti-sense binding | 2 out of 7474 genes, 0.0% | 3 out of 40099 genes, 0.0% | 1 | View Result |
| lactoylglutathione lyase activity | 8 out of 7474 genes, 0.1% | 26 out of 40099 genes, 0.1% | 1 | View Result |
| structural constituent of cell wall | 8 out of 7474 genes, 0.1% | 26 out of 40099 genes, 0.1% | 1 | View Result |
| oxidoreductase activity, acting on the CH-CH group of donors, quinone or related compound as acceptor | 8 out of 7474 genes, 0.1% | 26 out of 40099 genes, 0.1% | 1 | View Result |
| monocarboxylic acid binding | 8 out of 7474 genes, 0.1% | 26 out of 40099 genes, 0.1% | 1 | View Result |
| organic phosphonate transmembrane-transporting ATPase activity | 17 out of 7474 genes, 0.2% | 66 out of 40099 genes, 0.2% | 1 | View Result |
| organic phosphonate transmembrane transporter activity | 17 out of 7474 genes, 0.2% | 66 out of 40099 genes, 0.2% | 1 | View Result |
| organophosphate ester transmembrane transporter activity | 17 out of 7474 genes, 0.2% | 66 out of 40099 genes, 0.2% | 1 | View Result |
| cysteine-type endopeptidase inhibitor activity | 4 out of 7474 genes, 0.1% | 10 out of 40099 genes, 0.0% | 1 | View Result |
| RNA guanylyltransferase activity | 4 out of 7474 genes, 0.1% | 10 out of 40099 genes, 0.0% | 1 | View Result |
| histone kinase activity | 4 out of 7474 genes, 0.1% | 10 out of 40099 genes, 0.0% | 1 | View Result |
| endopeptidase inhibitor activity | 7 out of 7474 genes, 0.1% | 22 out of 40099 genes, 0.1% | 1 | View Result |
| endopeptidase regulator activity | 7 out of 7474 genes, 0.1% | 22 out of 40099 genes, 0.1% | 1 | View Result |
| oxidoreductase activity, oxidizing metal ions | 6 out of 7474 genes, 0.1% | 18 out of 40099 genes, 0.0% | 1 | View Result |
| desacetoxyvindoline 4-hydroxylase activity | 6 out of 7474 genes, 0.1% | 18 out of 40099 genes, 0.0% | 1 | View Result |
| beta-galactosidase activity | 16 out of 7474 genes, 0.2% | 62 out of 40099 genes, 0.2% | 1 | View Result |
| alpha-L-fucosidase activity | 5 out of 7474 genes, 0.1% | 14 out of 40099 genes, 0.0% | 1 | View Result |
| fucosidase activity | 5 out of 7474 genes, 0.1% | 14 out of 40099 genes, 0.0% | 1 | View Result |
| single-stranded RNA binding | 27 out of 7474 genes, 0.4% | 114 out of 40099 genes, 0.3% | 1 | View Result |
| fatty acid synthase activity | 17 out of 7474 genes, 0.2% | 67 out of 40099 genes, 0.2% | 1 | View Result |
| carbohydrate binding | 74 out of 7474 genes, 1.0% | 346 out of 40099 genes, 0.9% | 1 | View Result |
| amine transmembrane transporter activity | 9 out of 7474 genes, 0.1% | 31 out of 40099 genes, 0.1% | 1 | View Result |
| oxidoreductase activity, acting on NAD(P)H, quinone or similar compound as acceptor | 37 out of 7474 genes, 0.5% | 163 out of 40099 genes, 0.4% | 1 | View Result |
| sucrose synthase activity | 8 out of 7474 genes, 0.1% | 27 out of 40099 genes, 0.1% | 1 | View Result |
| galactosidase activity | 17 out of 7474 genes, 0.2% | 68 out of 40099 genes, 0.2% | 1 | View Result |
| steroid dehydrogenase activity | 15 out of 7474 genes, 0.2% | 59 out of 40099 genes, 0.1% | 1 | View Result |
| tRNA (guanine-N7-)-methyltransferase activity | 3 out of 7474 genes, 0.0% | 7 out of 40099 genes, 0.0% | 1 | View Result |
| xylose isomerase activity | 3 out of 7474 genes, 0.0% | 7 out of 40099 genes, 0.0% | 1 | View Result |
| L-alanine transmembrane transporter activity | 3 out of 7474 genes, 0.0% | 7 out of 40099 genes, 0.0% | 1 | View Result |
| alanine transmembrane transporter activity | 3 out of 7474 genes, 0.0% | 7 out of 40099 genes, 0.0% | 1 | View Result |
| L-tryptophan aminotransferase activity | 3 out of 7474 genes, 0.0% | 7 out of 40099 genes, 0.0% | 1 | View Result |
| carbon-oxygen lyase activity, acting on polysaccharides | 11 out of 7474 genes, 0.1% | 41 out of 40099 genes, 0.1% | 1 | View Result |
| pectate lyase activity | 11 out of 7474 genes, 0.1% | 41 out of 40099 genes, 0.1% | 1 | View Result |
| glycerate dehydrogenase activity | 5 out of 7474 genes, 0.1% | 15 out of 40099 genes, 0.0% | 1 | View Result |
| abscisic acid binding | 5 out of 7474 genes, 0.1% | 15 out of 40099 genes, 0.0% | 1 | View Result |
| methylammonium transmembrane transporter activity | 5 out of 7474 genes, 0.1% | 15 out of 40099 genes, 0.0% | 1 | View Result |
| isoprenoid binding | 5 out of 7474 genes, 0.1% | 15 out of 40099 genes, 0.0% | 1 | View Result |
| ammonia transmembrane transporter activity | 5 out of 7474 genes, 0.1% | 15 out of 40099 genes, 0.0% | 1 | View Result |
| pyrimidine nucleotide-sugar transmembrane transporter activity | 4 out of 7474 genes, 0.1% | 11 out of 40099 genes, 0.0% | 1 | View Result |
| cadmium-transporting ATPase activity | 4 out of 7474 genes, 0.1% | 11 out of 40099 genes, 0.0% | 1 | View Result |
| racemase and epimerase activity, acting on hydroxy acids and derivatives | 4 out of 7474 genes, 0.1% | 11 out of 40099 genes, 0.0% | 1 | View Result |
| NADPH binding | 4 out of 7474 genes, 0.1% | 11 out of 40099 genes, 0.0% | 1 | View Result |
| CoA-ligase activity | 23 out of 7474 genes, 0.3% | 98 out of 40099 genes, 0.2% | 1 | View Result |
| xylosyltransferase activity | 10 out of 7474 genes, 0.1% | 37 out of 40099 genes, 0.1% | 1 | View Result |
| actin filament binding | 25 out of 7474 genes, 0.3% | 108 out of 40099 genes, 0.3% | 1 | View Result |
| translation factor activity, nucleic acid binding | 127 out of 7474 genes, 1.7% | 623 out of 40099 genes, 1.6% | 1 | View Result |
| efflux transmembrane transporter activity | 21 out of 7474 genes, 0.3% | 89 out of 40099 genes, 0.2% | 1 | View Result |
| N,N-dimethylaniline monooxygenase activity | 7 out of 7474 genes, 0.1% | 24 out of 40099 genes, 0.1% | 1 | View Result |
| methyl jasmonate esterase activity | 7 out of 7474 genes, 0.1% | 24 out of 40099 genes, 0.1% | 1 | View Result |
| L-aspartate:2-oxoglutarate aminotransferase activity | 11 out of 7474 genes, 0.1% | 42 out of 40099 genes, 0.1% | 1 | View Result |
| zinc ion transmembrane transporter activity | 9 out of 7474 genes, 0.1% | 33 out of 40099 genes, 0.1% | 1 | View Result |
| basic amino acid transmembrane transporter activity | 6 out of 7474 genes, 0.1% | 20 out of 40099 genes, 0.0% | 1 | View Result |
| acid-thiol ligase activity | 23 out of 7474 genes, 0.3% | 100 out of 40099 genes, 0.2% | 1 | View Result |
| alpha-1,6-mannosyltransferase activity | 2 out of 7474 genes, 0.0% | 4 out of 40099 genes, 0.0% | 1 | View Result |
| gamma-glutamyltransferase activity | 2 out of 7474 genes, 0.0% | 4 out of 40099 genes, 0.0% | 1 | View Result |
| cholinesterase activity | 2 out of 7474 genes, 0.0% | 4 out of 40099 genes, 0.0% | 1 | View Result |
| leukotriene-A4 hydrolase activity | 2 out of 7474 genes, 0.0% | 4 out of 40099 genes, 0.0% | 1 | View Result |
| Rab geranylgeranyltransferase activity | 2 out of 7474 genes, 0.0% | 4 out of 40099 genes, 0.0% | 1 | View Result |
| sialyltransferase activity | 2 out of 7474 genes, 0.0% | 4 out of 40099 genes, 0.0% | 1 | View Result |
| nitrate:hydrogen symporter activity | 2 out of 7474 genes, 0.0% | 4 out of 40099 genes, 0.0% | 1 | View Result |
| 16:0 monogalactosyldiacylglycerol desaturase activity | 2 out of 7474 genes, 0.0% | 4 out of 40099 genes, 0.0% | 1 | View Result |
| acireductone dioxygenase [iron(II)-requiring] activity | 2 out of 7474 genes, 0.0% | 4 out of 40099 genes, 0.0% | 1 | View Result |
| L-galactose-1-phosphate phosphatase activity | 2 out of 7474 genes, 0.0% | 4 out of 40099 genes, 0.0% | 1 | View Result |
| gamma-aminobutyric acid transmembrane transporter activity | 2 out of 7474 genes, 0.0% | 4 out of 40099 genes, 0.0% | 1 | View Result |
| lysophosphatidic acid acyltransferase activity | 2 out of 7474 genes, 0.0% | 4 out of 40099 genes, 0.0% | 1 | View Result |
| galactinol-raffinose galactosyltransferase activity | 2 out of 7474 genes, 0.0% | 4 out of 40099 genes, 0.0% | 1 | View Result |
| GDP-mannose 3,5-epimerase activity | 2 out of 7474 genes, 0.0% | 4 out of 40099 genes, 0.0% | 1 | View Result |
| galactose-1-phosphate phosphatase activity | 2 out of 7474 genes, 0.0% | 4 out of 40099 genes, 0.0% | 1 | View Result |
| 5S rDNA binding | 2 out of 7474 genes, 0.0% | 4 out of 40099 genes, 0.0% | 1 | View Result |
| cysteine synthase activity | 5 out of 7474 genes, 0.1% | 16 out of 40099 genes, 0.0% | 1 | View Result |
| pyruvate decarboxylase activity | 5 out of 7474 genes, 0.1% | 16 out of 40099 genes, 0.0% | 1 | View Result |
| succinate-CoA ligase (GDP-forming) activity | 5 out of 7474 genes, 0.1% | 16 out of 40099 genes, 0.0% | 1 | View Result |
| cationic amino acid transmembrane transporter activity | 5 out of 7474 genes, 0.1% | 16 out of 40099 genes, 0.0% | 1 | View Result |
| xenobiotic-transporting ATPase activity | 21 out of 7474 genes, 0.3% | 91 out of 40099 genes, 0.2% | 1 | View Result |
| xenobiotic transporter activity | 21 out of 7474 genes, 0.3% | 91 out of 40099 genes, 0.2% | 1 | View Result |
| microtubule motor activity | 46 out of 7474 genes, 0.6% | 215 out of 40099 genes, 0.5% | 1 | View Result |
| phytochelatin transporter activity | 7 out of 7474 genes, 0.1% | 25 out of 40099 genes, 0.1% | 1 | View Result |
| phytochelatin transmembrane transporter activity | 7 out of 7474 genes, 0.1% | 25 out of 40099 genes, 0.1% | 1 | View Result |
| sugar transmembrane transporter activity | 41 out of 7474 genes, 0.5% | 190 out of 40099 genes, 0.5% | 1 | View Result |
| sterol 24-C-methyltransferase activity | 4 out of 7474 genes, 0.1% | 12 out of 40099 genes, 0.0% | 1 | View Result |
| cystathionine gamma-synthase activity | 4 out of 7474 genes, 0.1% | 12 out of 40099 genes, 0.0% | 1 | View Result |
| chlorophyll catabolite transmembrane transporter activity | 4 out of 7474 genes, 0.1% | 12 out of 40099 genes, 0.0% | 1 | View Result |
| glutathione S-conjugate-exporting ATPase activity | 4 out of 7474 genes, 0.1% | 12 out of 40099 genes, 0.0% | 1 | View Result |
| alkane 1-monooxygenase activity | 4 out of 7474 genes, 0.1% | 12 out of 40099 genes, 0.0% | 1 | View Result |
| intramolecular transferase activity, transferring hydroxy groups | 4 out of 7474 genes, 0.1% | 12 out of 40099 genes, 0.0% | 1 | View Result |
| glutathione S-conjugate-transporting ATPase activity | 4 out of 7474 genes, 0.1% | 12 out of 40099 genes, 0.0% | 1 | View Result |
| ferric-chelate reductase activity | 3 out of 7474 genes, 0.0% | 8 out of 40099 genes, 0.0% | 1 | View Result |
| actin monomer binding | 3 out of 7474 genes, 0.0% | 8 out of 40099 genes, 0.0% | 1 | View Result |
| choline kinase activity | 3 out of 7474 genes, 0.0% | 8 out of 40099 genes, 0.0% | 1 | View Result |
| oxalyl-CoA decarboxylase activity | 3 out of 7474 genes, 0.0% | 8 out of 40099 genes, 0.0% | 1 | View Result |
| flavonoid 3'-monooxygenase activity | 3 out of 7474 genes, 0.0% | 8 out of 40099 genes, 0.0% | 1 | View Result |
| delta12-fatty acid dehydrogenase activity | 3 out of 7474 genes, 0.0% | 8 out of 40099 genes, 0.0% | 1 | View Result |
| oxidoreductase activity, oxidizing metal ions, NAD or NADP as acceptor | 3 out of 7474 genes, 0.0% | 8 out of 40099 genes, 0.0% | 1 | View Result |
| 6-phosphogluconolactonase activity | 3 out of 7474 genes, 0.0% | 8 out of 40099 genes, 0.0% | 1 | View Result |
| translation repressor activity | 3 out of 7474 genes, 0.0% | 8 out of 40099 genes, 0.0% | 1 | View Result |
| 1-deoxy-D-xylulose-5-phosphate reductoisomerase activity | 3 out of 7474 genes, 0.0% | 8 out of 40099 genes, 0.0% | 1 | View Result |
| enone reductase activity | 3 out of 7474 genes, 0.0% | 8 out of 40099 genes, 0.0% | 1 | View Result |
| translation regulator activity | 3 out of 7474 genes, 0.0% | 8 out of 40099 genes, 0.0% | 1 | View Result |
| oxalate oxidase activity | 3 out of 7474 genes, 0.0% | 8 out of 40099 genes, 0.0% | 1 | View Result |
| glucuronosyl-N-acetylglucosaminyl-proteoglycan 4-alpha-N-acetylglucosaminyltransferase activity | 3 out of 7474 genes, 0.0% | 8 out of 40099 genes, 0.0% | 1 | View Result |
| carboxylic acid transmembrane transporter activity | 52 out of 7474 genes, 0.7% | 246 out of 40099 genes, 0.6% | 1 | View Result |
| serine-type peptidase activity | 75 out of 7474 genes, 1.0% | 363 out of 40099 genes, 0.9% | 1 | View Result |
| serine hydrolase activity | 75 out of 7474 genes, 1.0% | 363 out of 40099 genes, 0.9% | 1 | View Result |
| inward rectifier potassium channel activity | 8 out of 7474 genes, 0.1% | 30 out of 40099 genes, 0.1% | 1 | View Result |
| ribosome binding | 8 out of 7474 genes, 0.1% | 30 out of 40099 genes, 0.1% | 1 | View Result |
| acyl-CoA oxidase activity | 6 out of 7474 genes, 0.1% | 21 out of 40099 genes, 0.1% | 1 | View Result |
| quinone binding | 11 out of 7474 genes, 0.1% | 44 out of 40099 genes, 0.1% | 1 | View Result |
| transferase activity, transferring acyl groups, acyl groups converted into alkyl on transfer | 16 out of 7474 genes, 0.2% | 68 out of 40099 genes, 0.2% | 1 | View Result |
| oxidoreductase activity, acting on NAD(P)H | 67 out of 7474 genes, 0.9% | 324 out of 40099 genes, 0.8% | 1 | View Result |
| organic acid transmembrane transporter activity | 54 out of 7474 genes, 0.7% | 258 out of 40099 genes, 0.6% | 1 | View Result |
| flavin adenine dinucleotide binding | 63 out of 7474 genes, 0.8% | 304 out of 40099 genes, 0.8% | 1 | View Result |
| L-malate dehydrogenase activity | 7 out of 7474 genes, 0.1% | 26 out of 40099 genes, 0.1% | 1 | View Result |
| phosphorelay response regulator activity | 16 out of 7474 genes, 0.2% | 69 out of 40099 genes, 0.2% | 1 | View Result |
| C-4 methylsterol oxidase activity | 4 out of 7474 genes, 0.1% | 13 out of 40099 genes, 0.0% | 1 | View Result |
| mRNA 3'-UTR binding | 4 out of 7474 genes, 0.1% | 13 out of 40099 genes, 0.0% | 1 | View Result |
| ketol-acid reductoisomerase activity | 4 out of 7474 genes, 0.1% | 13 out of 40099 genes, 0.0% | 1 | View Result |
| serine-type endopeptidase inhibitor activity | 4 out of 7474 genes, 0.1% | 13 out of 40099 genes, 0.0% | 1 | View Result |
| phosphatidylcholine 1-acylhydrolase activity | 4 out of 7474 genes, 0.1% | 13 out of 40099 genes, 0.0% | 1 | View Result |
| malate transmembrane transporter activity | 4 out of 7474 genes, 0.1% | 13 out of 40099 genes, 0.0% | 1 | View Result |
| outward rectifier potassium channel activity | 4 out of 7474 genes, 0.1% | 13 out of 40099 genes, 0.0% | 1 | View Result |
| oxidoreductase activity, acting on paired donors, with incorporation or reduction of molecular oxygen, reduced iron-sulfur protein as one donor, and incorporation of one atom of oxygen | 4 out of 7474 genes, 0.1% | 13 out of 40099 genes, 0.0% | 1 | View Result |
| phospholipid:diacylglycerol acyltransferase activity | 4 out of 7474 genes, 0.1% | 13 out of 40099 genes, 0.0% | 1 | View Result |
| disulfide oxidoreductase activity | 32 out of 7474 genes, 0.4% | 149 out of 40099 genes, 0.4% | 1 | View Result |
| oxidoreductase activity, acting on paired donors, with incorporation or reduction of molecular oxygen, reduced flavin or flavoprotein as one donor, and incorporation of one atom of oxygen | 6 out of 7474 genes, 0.1% | 22 out of 40099 genes, 0.1% | 1 | View Result |
| diphosphate-fructose-6-phosphate 1-phosphotransferase activity | 6 out of 7474 genes, 0.1% | 22 out of 40099 genes, 0.1% | 1 | View Result |
| aromatase activity | 6 out of 7474 genes, 0.1% | 22 out of 40099 genes, 0.1% | 1 | View Result |
| hormone binding | 9 out of 7474 genes, 0.1% | 36 out of 40099 genes, 0.1% | 1 | View Result |
| nitrate transmembrane transporter activity | 10 out of 7474 genes, 0.1% | 41 out of 40099 genes, 0.1% | 1 | View Result |
| DNA primase activity | 3 out of 7474 genes, 0.0% | 9 out of 40099 genes, 0.0% | 1 | View Result |
| acetoacetyl-CoA reductase activity | 3 out of 7474 genes, 0.0% | 9 out of 40099 genes, 0.0% | 1 | View Result |
| ketoreductase activity | 3 out of 7474 genes, 0.0% | 9 out of 40099 genes, 0.0% | 1 | View Result |
| nucleoside-triphosphate diphosphatase activity | 3 out of 7474 genes, 0.0% | 9 out of 40099 genes, 0.0% | 1 | View Result |
| m7G(5')pppN diphosphatase activity | 3 out of 7474 genes, 0.0% | 9 out of 40099 genes, 0.0% | 1 | View Result |
| histidine phosphotransfer kinase activity | 7 out of 7474 genes, 0.1% | 27 out of 40099 genes, 0.1% | 1 | View Result |
| ligase activity, forming carbon-sulfur bonds | 29 out of 7474 genes, 0.4% | 135 out of 40099 genes, 0.3% | 1 | View Result |
| 3-deoxy-7-phosphoheptulonate synthase activity | 5 out of 7474 genes, 0.1% | 18 out of 40099 genes, 0.0% | 1 | View Result |
| glucose-6-phosphate dehydrogenase activity | 5 out of 7474 genes, 0.1% | 18 out of 40099 genes, 0.0% | 1 | View Result |
| sulfate adenylyltransferase (ATP) activity | 5 out of 7474 genes, 0.1% | 18 out of 40099 genes, 0.0% | 1 | View Result |
| 4-coumarate-CoA ligase activity | 5 out of 7474 genes, 0.1% | 18 out of 40099 genes, 0.0% | 1 | View Result |
| oxidoreductase activity, acting on the CH-NH2 group of donors, oxygen as acceptor | 13 out of 7474 genes, 0.2% | 56 out of 40099 genes, 0.1% | 1 | View Result |
| rDNA binding | 2 out of 7474 genes, 0.0% | 5 out of 40099 genes, 0.0% | 1 | View Result |
| 4-aminobutyrate transaminase activity | 2 out of 7474 genes, 0.0% | 5 out of 40099 genes, 0.0% | 1 | View Result |
| alkylbase DNA N-glycosylase activity | 2 out of 7474 genes, 0.0% | 5 out of 40099 genes, 0.0% | 1 | View Result |
| L-alanine:2-oxoglutarate aminotransferase activity | 2 out of 7474 genes, 0.0% | 5 out of 40099 genes, 0.0% | 1 | View Result |
| aspartate carbamoyltransferase activity | 2 out of 7474 genes, 0.0% | 5 out of 40099 genes, 0.0% | 1 | View Result |
| ornithine-oxo-acid transaminase activity | 2 out of 7474 genes, 0.0% | 5 out of 40099 genes, 0.0% | 1 | View Result |
| nucleobase:cation symporter activity | 2 out of 7474 genes, 0.0% | 5 out of 40099 genes, 0.0% | 1 | View Result |
| endoribonuclease activity, producing 3'-phosphomonoesters | 2 out of 7474 genes, 0.0% | 5 out of 40099 genes, 0.0% | 1 | View Result |
| endonuclease activity, active with either ribo- or deoxyribonucleic acids and producing 3'-phosphomonoesters | 2 out of 7474 genes, 0.0% | 5 out of 40099 genes, 0.0% | 1 | View Result |
| nucleoside-diphosphatase activity | 2 out of 7474 genes, 0.0% | 5 out of 40099 genes, 0.0% | 1 | View Result |
| peptide deformylase activity | 2 out of 7474 genes, 0.0% | 5 out of 40099 genes, 0.0% | 1 | View Result |
| phosphatidylinositol-3,4-bisphosphate binding | 2 out of 7474 genes, 0.0% | 5 out of 40099 genes, 0.0% | 1 | View Result |
| dehydrodolichyl diphosphate synthase activity | 2 out of 7474 genes, 0.0% | 5 out of 40099 genes, 0.0% | 1 | View Result |
| alanine-oxo-acid transaminase activity | 2 out of 7474 genes, 0.0% | 5 out of 40099 genes, 0.0% | 1 | View Result |
| aldose-6-phosphate reductase (NADPH) activity | 2 out of 7474 genes, 0.0% | 5 out of 40099 genes, 0.0% | 1 | View Result |
| delta4-3-oxosteroid 5beta-reductase activity | 2 out of 7474 genes, 0.0% | 5 out of 40099 genes, 0.0% | 1 | View Result |
| tocopherol O-methyltransferase activity | 2 out of 7474 genes, 0.0% | 5 out of 40099 genes, 0.0% | 1 | View Result |
| tropine dehydrogenase activity | 2 out of 7474 genes, 0.0% | 5 out of 40099 genes, 0.0% | 1 | View Result |
| L-tryptophan:2-oxoglutarate aminotransferase activity | 2 out of 7474 genes, 0.0% | 5 out of 40099 genes, 0.0% | 1 | View Result |
| beta-sitosterol UDP-glucosyltransferase activity | 2 out of 7474 genes, 0.0% | 5 out of 40099 genes, 0.0% | 1 | View Result |
| L-tryptophan:pyruvate aminotransferase activity | 2 out of 7474 genes, 0.0% | 5 out of 40099 genes, 0.0% | 1 | View Result |
| enoyl-CoA hydratase activity | 9 out of 7474 genes, 0.1% | 37 out of 40099 genes, 0.1% | 1 | View Result |
| fructose-bisphosphate aldolase activity | 6 out of 7474 genes, 0.1% | 23 out of 40099 genes, 0.1% | 1 | View Result |
| phosphopyruvate hydratase activity | 6 out of 7474 genes, 0.1% | 23 out of 40099 genes, 0.1% | 1 | View Result |
| myo-inositol:hydrogen symporter activity | 6 out of 7474 genes, 0.1% | 23 out of 40099 genes, 0.1% | 1 | View Result |
| S-(hydroxymethyl)glutathione dehydrogenase activity | 6 out of 7474 genes, 0.1% | 23 out of 40099 genes, 0.1% | 1 | View Result |
| inorganic phosphate transmembrane transporter activity | 11 out of 7474 genes, 0.1% | 47 out of 40099 genes, 0.1% | 1 | View Result |
| transferase activity, transferring alkyl or aryl (other than methyl) groups | 40 out of 7474 genes, 0.5% | 193 out of 40099 genes, 0.5% | 1 | View Result |
| arsenate reductase (glutaredoxin) activity | 4 out of 7474 genes, 0.1% | 14 out of 40099 genes, 0.0% | 1 | View Result |
| oxidoreductase activity, acting on phosphorus or arsenic in donors | 4 out of 7474 genes, 0.1% | 14 out of 40099 genes, 0.0% | 1 | View Result |
| oxidoreductase activity, acting on phosphorus or arsenic in donors, disulfide as acceptor | 4 out of 7474 genes, 0.1% | 14 out of 40099 genes, 0.0% | 1 | View Result |
| bile acid:sodium symporter activity | 7 out of 7474 genes, 0.1% | 28 out of 40099 genes, 0.1% | 1 | View Result |
| bile acid transmembrane transporter activity | 7 out of 7474 genes, 0.1% | 28 out of 40099 genes, 0.1% | 1 | View Result |
| solute:hydrogen symporter activity | 32 out of 7474 genes, 0.4% | 153 out of 40099 genes, 0.4% | 1 | View Result |
| oxidoreductase activity, acting on the CH-CH group of donors, oxygen as acceptor | 9 out of 7474 genes, 0.1% | 38 out of 40099 genes, 0.1% | 1 | View Result |
| C-methyltransferase activity | 5 out of 7474 genes, 0.1% | 19 out of 40099 genes, 0.0% | 1 | View Result |
| succinate dehydrogenase (ubiquinone) activity | 5 out of 7474 genes, 0.1% | 19 out of 40099 genes, 0.0% | 1 | View Result |
| protein xylosyltransferase activity | 5 out of 7474 genes, 0.1% | 19 out of 40099 genes, 0.0% | 1 | View Result |
| electron transporter, transferring electrons within CoQH2-cytochrome c reductase complex activity | 5 out of 7474 genes, 0.1% | 19 out of 40099 genes, 0.0% | 1 | View Result |
| transferase activity, transferring sulfur-containing groups | 12 out of 7474 genes, 0.2% | 53 out of 40099 genes, 0.1% | 1 | View Result |
| protein serine/threonine/tyrosine kinase activity | 47 out of 7474 genes, 0.6% | 231 out of 40099 genes, 0.6% | 1 | View Result |
| NAD binding | 46 out of 7474 genes, 0.6% | 226 out of 40099 genes, 0.6% | 1 | View Result |
| deoxyribodipyrimidine photo-lyase activity | 3 out of 7474 genes, 0.0% | 10 out of 40099 genes, 0.0% | 1 | View Result |
| dihydroxy-acid dehydratase activity | 3 out of 7474 genes, 0.0% | 10 out of 40099 genes, 0.0% | 1 | View Result |
| ferroxidase activity | 3 out of 7474 genes, 0.0% | 10 out of 40099 genes, 0.0% | 1 | View Result |
| phosphoglycerate kinase activity | 3 out of 7474 genes, 0.0% | 10 out of 40099 genes, 0.0% | 1 | View Result |
| dTDP-glucose 4,6-dehydratase activity | 3 out of 7474 genes, 0.0% | 10 out of 40099 genes, 0.0% | 1 | View Result |
| alternative oxidase activity | 3 out of 7474 genes, 0.0% | 10 out of 40099 genes, 0.0% | 1 | View Result |
| oxidoreductase activity, oxidizing metal ions, oxygen as acceptor | 3 out of 7474 genes, 0.0% | 10 out of 40099 genes, 0.0% | 1 | View Result |
| desulfoglucosinolate sulfotransferase activity | 3 out of 7474 genes, 0.0% | 10 out of 40099 genes, 0.0% | 1 | View Result |
| sphingosine N-acyltransferase activity | 3 out of 7474 genes, 0.0% | 10 out of 40099 genes, 0.0% | 1 | View Result |
| dihydrolipoyl dehydrogenase activity | 6 out of 7474 genes, 0.1% | 24 out of 40099 genes, 0.1% | 1 | View Result |
| 5S rRNA binding | 6 out of 7474 genes, 0.1% | 24 out of 40099 genes, 0.1% | 1 | View Result |
| L-ascorbic acid binding | 6 out of 7474 genes, 0.1% | 24 out of 40099 genes, 0.1% | 1 | View Result |
| acyl-CoA hydrolase activity | 6 out of 7474 genes, 0.1% | 24 out of 40099 genes, 0.1% | 1 | View Result |
| DNA topoisomerase activity | 7 out of 7474 genes, 0.1% | 29 out of 40099 genes, 0.1% | 1 | View Result |
| arsenite secondary active transmembrane transporter activity | 7 out of 7474 genes, 0.1% | 29 out of 40099 genes, 0.1% | 1 | View Result |
| arsenite-transmembrane transporting ATPase activity | 7 out of 7474 genes, 0.1% | 29 out of 40099 genes, 0.1% | 1 | View Result |
| identical protein binding | 102 out of 7474 genes, 1.4% | 519 out of 40099 genes, 1.3% | 1 | View Result |
| nucleotide-sugar transmembrane transporter activity | 8 out of 7474 genes, 0.1% | 34 out of 40099 genes, 0.1% | 1 | View Result |
| unfolded protein binding | 69 out of 7474 genes, 0.9% | 347 out of 40099 genes, 0.9% | 1 | View Result |
| anion transmembrane-transporting ATPase activity | 29 out of 7474 genes, 0.4% | 140 out of 40099 genes, 0.3% | 1 | View Result |
| potassium channel activity | 17 out of 7474 genes, 0.2% | 79 out of 40099 genes, 0.2% | 1 | View Result |
| transmembrane receptor protein tyrosine kinase activity | 10 out of 7474 genes, 0.1% | 44 out of 40099 genes, 0.1% | 1 | View Result |
| monocarboxylic acid transmembrane transporter activity | 10 out of 7474 genes, 0.1% | 44 out of 40099 genes, 0.1% | 1 | View Result |
| diphosphotransferase activity | 10 out of 7474 genes, 0.1% | 44 out of 40099 genes, 0.1% | 1 | View Result |
| RNA-directed DNA polymerase activity | 14 out of 7474 genes, 0.2% | 64 out of 40099 genes, 0.2% | 1 | View Result |
| Rab GDP-dissociation inhibitor activity | 4 out of 7474 genes, 0.1% | 15 out of 40099 genes, 0.0% | 1 | View Result |
| cyclo-ligase activity | 4 out of 7474 genes, 0.1% | 15 out of 40099 genes, 0.0% | 1 | View Result |
| pyrophosphatase activity | 643 out of 7474 genes, 8.6% | 3388 out of 40099 genes, 8.4% | 1 | View Result |
| organic anion transmembrane transporter activity | 79 out of 7474 genes, 1.1% | 401 out of 40099 genes, 1.0% | 1 | View Result |
| 3-dehydroquinate synthase activity | 2 out of 7474 genes, 0.0% | 6 out of 40099 genes, 0.0% | 1 | View Result |
| asparaginase activity | 2 out of 7474 genes, 0.0% | 6 out of 40099 genes, 0.0% | 1 | View Result |
| choline-phosphate cytidylyltransferase activity | 2 out of 7474 genes, 0.0% | 6 out of 40099 genes, 0.0% | 1 | View Result |
| ferredoxin-NADP+ reductase activity | 2 out of 7474 genes, 0.0% | 6 out of 40099 genes, 0.0% | 1 | View Result |
| polynucleotide 5'-phosphatase activity | 2 out of 7474 genes, 0.0% | 6 out of 40099 genes, 0.0% | 1 | View Result |
| intracellular cyclic nucleotide activated cation channel activity | 2 out of 7474 genes, 0.0% | 6 out of 40099 genes, 0.0% | 1 | View Result |
| four-way junction helicase activity | 2 out of 7474 genes, 0.0% | 6 out of 40099 genes, 0.0% | 1 | View Result |
| maleylacetoacetate isomerase activity | 2 out of 7474 genes, 0.0% | 6 out of 40099 genes, 0.0% | 1 | View Result |
| cycloartenol synthase activity | 2 out of 7474 genes, 0.0% | 6 out of 40099 genes, 0.0% | 1 | View Result |
| phosphopantetheine binding | 2 out of 7474 genes, 0.0% | 6 out of 40099 genes, 0.0% | 1 | View Result |
| maltose alpha-glucosidase activity | 2 out of 7474 genes, 0.0% | 6 out of 40099 genes, 0.0% | 1 | View Result |
| xyloglucan 6-xylosyltransferase activity | 2 out of 7474 genes, 0.0% | 6 out of 40099 genes, 0.0% | 1 | View Result |
| histone methyltransferase activity (H3-K4 specific) | 2 out of 7474 genes, 0.0% | 6 out of 40099 genes, 0.0% | 1 | View Result |
| cyclic nucleotide-gated ion channel activity | 2 out of 7474 genes, 0.0% | 6 out of 40099 genes, 0.0% | 1 | View Result |
| galactinol-sucrose galactosyltransferase activity | 2 out of 7474 genes, 0.0% | 6 out of 40099 genes, 0.0% | 1 | View Result |
| RNA polymerase binding | 2 out of 7474 genes, 0.0% | 6 out of 40099 genes, 0.0% | 1 | View Result |
| core RNA polymerase binding transcription factor activity | 5 out of 7474 genes, 0.1% | 20 out of 40099 genes, 0.0% | 1 | View Result |
| core DNA-dependent RNA polymerase binding promoter specificity activity | 5 out of 7474 genes, 0.1% | 20 out of 40099 genes, 0.0% | 1 | View Result |
| sulfate adenylyltransferase activity | 5 out of 7474 genes, 0.1% | 20 out of 40099 genes, 0.0% | 1 | View Result |
| GDP-dissociation inhibitor activity | 5 out of 7474 genes, 0.1% | 20 out of 40099 genes, 0.0% | 1 | View Result |
| phosphate ion transmembrane transporter activity | 5 out of 7474 genes, 0.1% | 20 out of 40099 genes, 0.0% | 1 | View Result |
| sigma factor activity | 5 out of 7474 genes, 0.1% | 20 out of 40099 genes, 0.0% | 1 | View Result |
| retinyl-palmitate esterase activity | 5 out of 7474 genes, 0.1% | 20 out of 40099 genes, 0.0% | 1 | View Result |
| chaperone binding | 5 out of 7474 genes, 0.1% | 20 out of 40099 genes, 0.0% | 1 | View Result |
| carbon-carbon lyase activity | 65 out of 7474 genes, 0.9% | 328 out of 40099 genes, 0.8% | 1 | View Result |
| cobalt ion binding | 45 out of 7474 genes, 0.6% | 224 out of 40099 genes, 0.6% | 1 | View Result |
| proton-transporting ATP synthase activity, rotational mechanism | 26 out of 7474 genes, 0.3% | 126 out of 40099 genes, 0.3% | 1 | View Result |
| voltage-gated potassium channel activity | 16 out of 7474 genes, 0.2% | 75 out of 40099 genes, 0.2% | 1 | View Result |
| serine-type exopeptidase activity | 16 out of 7474 genes, 0.2% | 75 out of 40099 genes, 0.2% | 1 | View Result |
| endodeoxyribonuclease activity | 6 out of 7474 genes, 0.1% | 25 out of 40099 genes, 0.1% | 1 | View Result |
| ribose phosphate diphosphokinase activity | 6 out of 7474 genes, 0.1% | 25 out of 40099 genes, 0.1% | 1 | View Result |
| cyclase activity | 6 out of 7474 genes, 0.1% | 25 out of 40099 genes, 0.1% | 1 | View Result |
| methyl salicylate esterase activity | 6 out of 7474 genes, 0.1% | 25 out of 40099 genes, 0.1% | 1 | View Result |
| NADH dehydrogenase activity | 24 out of 7474 genes, 0.3% | 116 out of 40099 genes, 0.3% | 1 | View Result |
| vitamin binding | 14 out of 7474 genes, 0.2% | 65 out of 40099 genes, 0.2% | 1 | View Result |
| protein domain specific binding | 14 out of 7474 genes, 0.2% | 65 out of 40099 genes, 0.2% | 1 | View Result |
| lyase activity | 232 out of 7474 genes, 3.1% | 1209 out of 40099 genes, 3.0% | 1 | View Result |
| deoxyribonuclease activity | 7 out of 7474 genes, 0.1% | 30 out of 40099 genes, 0.1% | 1 | View Result |
| hydrolase activity, acting on acid anhydrides, in phosphorus-containing anhydrides | 645 out of 7474 genes, 8.6% | 3404 out of 40099 genes, 8.5% | 1 | View Result |
| glycogenin glucosyltransferase activity | 8 out of 7474 genes, 0.1% | 35 out of 40099 genes, 0.1% | 1 | View Result |
| tau-protein kinase activity | 11 out of 7474 genes, 0.1% | 50 out of 40099 genes, 0.1% | 1 | View Result |
| copper ion transmembrane transporter activity | 9 out of 7474 genes, 0.1% | 40 out of 40099 genes, 0.1% | 1 | View Result |
| hydrolase activity, acting on acid anhydrides | 654 out of 7474 genes, 8.8% | 3454 out of 40099 genes, 8.6% | 1 | View Result |
| amino acid transmembrane transporter activity | 36 out of 7474 genes, 0.5% | 179 out of 40099 genes, 0.4% | 1 | View Result |
| solute:hydrogen antiporter activity | 17 out of 7474 genes, 0.2% | 81 out of 40099 genes, 0.2% | 1 | View Result |
| calcium:hydrogen antiporter activity | 3 out of 7474 genes, 0.0% | 11 out of 40099 genes, 0.0% | 1 | View Result |
| ether hydrolase activity | 3 out of 7474 genes, 0.0% | 11 out of 40099 genes, 0.0% | 1 | View Result |
| GTPase activating protein binding | 3 out of 7474 genes, 0.0% | 11 out of 40099 genes, 0.0% | 1 | View Result |
| protein histidine kinase activity | 16 out of 7474 genes, 0.2% | 76 out of 40099 genes, 0.2% | 1 | View Result |
| phosphotransferase activity, nitrogenous group as acceptor | 16 out of 7474 genes, 0.2% | 76 out of 40099 genes, 0.2% | 1 | View Result |
| pyruvate dehydrogenase activity | 12 out of 7474 genes, 0.2% | 56 out of 40099 genes, 0.1% | 1 | View Result |
| pyruvate dehydrogenase (acetyl-transferring) activity | 12 out of 7474 genes, 0.2% | 56 out of 40099 genes, 0.1% | 1 | View Result |
| DNA topoisomerase type II (ATP-hydrolyzing) activity | 4 out of 7474 genes, 0.1% | 16 out of 40099 genes, 0.0% | 1 | View Result |
| diacylglycerol O-acyltransferase activity | 4 out of 7474 genes, 0.1% | 16 out of 40099 genes, 0.0% | 1 | View Result |
| L-glutamate transmembrane transporter activity | 4 out of 7474 genes, 0.1% | 16 out of 40099 genes, 0.0% | 1 | View Result |
| molybdate ion transmembrane transporter activity | 4 out of 7474 genes, 0.1% | 16 out of 40099 genes, 0.0% | 1 | View Result |
| tRNA (guanine) methyltransferase activity | 4 out of 7474 genes, 0.1% | 16 out of 40099 genes, 0.0% | 1 | View Result |
| 1-aminocyclopropane-1-carboxylate synthase activity | 4 out of 7474 genes, 0.1% | 16 out of 40099 genes, 0.0% | 1 | View Result |
| arsenate reductase activity | 4 out of 7474 genes, 0.1% | 16 out of 40099 genes, 0.0% | 1 | View Result |
| DNA topoisomerase II activity | 4 out of 7474 genes, 0.1% | 16 out of 40099 genes, 0.0% | 1 | View Result |
| osmosensor activity | 5 out of 7474 genes, 0.1% | 21 out of 40099 genes, 0.1% | 1 | View Result |
| glucuronosyltransferase activity | 5 out of 7474 genes, 0.1% | 21 out of 40099 genes, 0.1% | 1 | View Result |
| phospholipase A2 activity | 6 out of 7474 genes, 0.1% | 26 out of 40099 genes, 0.1% | 1 | View Result |
| intramolecular lyase activity | 6 out of 7474 genes, 0.1% | 26 out of 40099 genes, 0.1% | 1 | View Result |
| thiamine pyrophosphate binding | 6 out of 7474 genes, 0.1% | 26 out of 40099 genes, 0.1% | 1 | View Result |
| intramolecular transferase activity | 32 out of 7474 genes, 0.4% | 160 out of 40099 genes, 0.4% | 1 | View Result |
| sugar:hydrogen symporter activity | 30 out of 7474 genes, 0.4% | 150 out of 40099 genes, 0.4% | 1 | View Result |
| cation:sugar symporter activity | 30 out of 7474 genes, 0.4% | 150 out of 40099 genes, 0.4% | 1 | View Result |
| cis-trans isomerase activity | 30 out of 7474 genes, 0.4% | 150 out of 40099 genes, 0.4% | 1 | View Result |
| iron ion binding | 118 out of 7474 genes, 1.6% | 614 out of 40099 genes, 1.5% | 1 | View Result |
| voltage-gated cation channel activity | 17 out of 7474 genes, 0.2% | 83 out of 40099 genes, 0.2% | 1 | View Result |
| water transmembrane transporter activity | 9 out of 7474 genes, 0.1% | 42 out of 40099 genes, 0.1% | 1 | View Result |
| water channel activity | 9 out of 7474 genes, 0.1% | 42 out of 40099 genes, 0.1% | 1 | View Result |
| ACP phosphopantetheine attachment site binding involved in fatty acid biosynthetic process | 2 out of 7474 genes, 0.0% | 7 out of 40099 genes, 0.0% | 1 | View Result |
| acetolactate synthase activity | 2 out of 7474 genes, 0.0% | 7 out of 40099 genes, 0.0% | 1 | View Result |
| dolichyl-phosphate-mannose-protein mannosyltransferase activity | 2 out of 7474 genes, 0.0% | 7 out of 40099 genes, 0.0% | 1 | View Result |
| estradiol 17-beta-dehydrogenase activity | 2 out of 7474 genes, 0.0% | 7 out of 40099 genes, 0.0% | 1 | View Result |
| homoserine dehydrogenase activity | 2 out of 7474 genes, 0.0% | 7 out of 40099 genes, 0.0% | 1 | View Result |
| mRNA guanylyltransferase activity | 2 out of 7474 genes, 0.0% | 7 out of 40099 genes, 0.0% | 1 | View Result |
| sarcosine oxidase activity | 2 out of 7474 genes, 0.0% | 7 out of 40099 genes, 0.0% | 1 | View Result |
| nitrate reductase activity | 2 out of 7474 genes, 0.0% | 7 out of 40099 genes, 0.0% | 1 | View Result |
| iron chelate transmembrane transporter activity | 2 out of 7474 genes, 0.0% | 7 out of 40099 genes, 0.0% | 1 | View Result |
| long-chain-enoyl-CoA hydratase activity | 2 out of 7474 genes, 0.0% | 7 out of 40099 genes, 0.0% | 1 | View Result |
| sterol 3-beta-glucosyltransferase activity | 2 out of 7474 genes, 0.0% | 7 out of 40099 genes, 0.0% | 1 | View Result |
| peptide-methionine (R)-S-oxide reductase activity | 2 out of 7474 genes, 0.0% | 7 out of 40099 genes, 0.0% | 1 | View Result |
| isoflavone 2'-hydroxylase activity | 2 out of 7474 genes, 0.0% | 7 out of 40099 genes, 0.0% | 1 | View Result |
| 3'-tRNA processing endoribonuclease activity | 2 out of 7474 genes, 0.0% | 7 out of 40099 genes, 0.0% | 1 | View Result |
| ribosomal large subunit binding | 2 out of 7474 genes, 0.0% | 7 out of 40099 genes, 0.0% | 1 | View Result |
| ACP phosphopantetheine attachment site binding | 2 out of 7474 genes, 0.0% | 7 out of 40099 genes, 0.0% | 1 | View Result |
| inositol phosphoceramide synthase activity | 2 out of 7474 genes, 0.0% | 7 out of 40099 genes, 0.0% | 1 | View Result |
| long-chain-alcohol oxidase activity | 2 out of 7474 genes, 0.0% | 7 out of 40099 genes, 0.0% | 1 | View Result |
| 2'-hydroxyisoflavone reductase activity | 2 out of 7474 genes, 0.0% | 7 out of 40099 genes, 0.0% | 1 | View Result |
| GDP-L-fucose synthase activity | 2 out of 7474 genes, 0.0% | 7 out of 40099 genes, 0.0% | 1 | View Result |
| prosthetic group binding | 2 out of 7474 genes, 0.0% | 7 out of 40099 genes, 0.0% | 1 | View Result |
| iron-nicotianamine transmembrane transporter activity | 2 out of 7474 genes, 0.0% | 7 out of 40099 genes, 0.0% | 1 | View Result |
| ATPase regulator activity | 2 out of 7474 genes, 0.0% | 7 out of 40099 genes, 0.0% | 1 | View Result |
| lysophospholipid acyltransferase activity | 2 out of 7474 genes, 0.0% | 7 out of 40099 genes, 0.0% | 1 | View Result |
| 8-methylthiopropyl glucosinolate S-oxygenase activity | 2 out of 7474 genes, 0.0% | 7 out of 40099 genes, 0.0% | 1 | View Result |
| succinate-CoA ligase activity | 7 out of 7474 genes, 0.1% | 32 out of 40099 genes, 0.1% | 1 | View Result |
| succinate-CoA ligase (ADP-forming) activity | 7 out of 7474 genes, 0.1% | 32 out of 40099 genes, 0.1% | 1 | View Result |
| dicarboxylic acid transmembrane transporter activity | 7 out of 7474 genes, 0.1% | 32 out of 40099 genes, 0.1% | 1 | View Result |
| UDP-xylosyltransferase activity | 6 out of 7474 genes, 0.1% | 27 out of 40099 genes, 0.1% | 1 | View Result |
| quercetin 3-O-glucosyltransferase activity | 6 out of 7474 genes, 0.1% | 27 out of 40099 genes, 0.1% | 1 | View Result |
| solute:cation antiporter activity | 18 out of 7474 genes, 0.2% | 89 out of 40099 genes, 0.2% | 1 | View Result |
| ATPase activity, coupled to transmembrane movement of ions, rotational mechanism | 31 out of 7474 genes, 0.4% | 157 out of 40099 genes, 0.4% | 1 | View Result |
| phosphotransferase activity, paired acceptors | 5 out of 7474 genes, 0.1% | 22 out of 40099 genes, 0.1% | 1 | View Result |
| 1,4-alpha-glucan branching enzyme activity | 3 out of 7474 genes, 0.0% | 12 out of 40099 genes, 0.0% | 1 | View Result |
| adenosine kinase activity | 3 out of 7474 genes, 0.0% | 12 out of 40099 genes, 0.0% | 1 | View Result |
| tetrahydrofolylpolyglutamate synthase activity | 3 out of 7474 genes, 0.0% | 12 out of 40099 genes, 0.0% | 1 | View Result |
| peptide-methionine (S)-S-oxide reductase activity | 3 out of 7474 genes, 0.0% | 12 out of 40099 genes, 0.0% | 1 | View Result |
| GTP diphosphokinase activity | 3 out of 7474 genes, 0.0% | 12 out of 40099 genes, 0.0% | 1 | View Result |
| UDP-L-rhamnose synthase activity | 3 out of 7474 genes, 0.0% | 12 out of 40099 genes, 0.0% | 1 | View Result |
| oxidoreductase activity, acting on iron-sulfur proteins as donors | 3 out of 7474 genes, 0.0% | 12 out of 40099 genes, 0.0% | 1 | View Result |
| (E)-beta-ocimene synthase activity | 3 out of 7474 genes, 0.0% | 12 out of 40099 genes, 0.0% | 1 | View Result |
| UDP-glucose 4,6-dehydratase activity | 3 out of 7474 genes, 0.0% | 12 out of 40099 genes, 0.0% | 1 | View Result |
| metal ion:hydrogen antiporter activity | 3 out of 7474 genes, 0.0% | 12 out of 40099 genes, 0.0% | 1 | View Result |
| nucleoside-triphosphatase activity | 608 out of 7474 genes, 8.1% | 3229 out of 40099 genes, 8.1% | 1 | View Result |
| glutathione peroxidase activity | 4 out of 7474 genes, 0.1% | 17 out of 40099 genes, 0.0% | 1 | View Result |
| C4-dicarboxylate transmembrane transporter activity | 4 out of 7474 genes, 0.1% | 17 out of 40099 genes, 0.0% | 1 | View Result |
| oxidoreductase activity, acting on other nitrogenous compounds as donors | 4 out of 7474 genes, 0.1% | 17 out of 40099 genes, 0.0% | 1 | View Result |
| oxidosqualene cyclase activity | 4 out of 7474 genes, 0.1% | 17 out of 40099 genes, 0.0% | 1 | View Result |
| UDP-glucuronate 4-epimerase activity | 4 out of 7474 genes, 0.1% | 17 out of 40099 genes, 0.0% | 1 | View Result |
| reticuline oxidase activity | 4 out of 7474 genes, 0.1% | 17 out of 40099 genes, 0.0% | 1 | View Result |
| protein disulfide oxidoreductase activity | 22 out of 7474 genes, 0.3% | 110 out of 40099 genes, 0.3% | 1 | View Result |
| triglyceride lipase activity | 12 out of 7474 genes, 0.2% | 58 out of 40099 genes, 0.1% | 1 | View Result |
| cytoskeletal protein binding | 97 out of 7474 genes, 1.3% | 506 out of 40099 genes, 1.3% | 1 | View Result |
| phosphatidylinositol phosphate kinase activity | 10 out of 7474 genes, 0.1% | 48 out of 40099 genes, 0.1% | 1 | View Result |
| oxidoreductase activity, acting on the aldehyde or oxo group of donors, disulfide as acceptor | 15 out of 7474 genes, 0.2% | 74 out of 40099 genes, 0.2% | 1 | View Result |
| translation initiation factor activity | 63 out of 7474 genes, 0.8% | 327 out of 40099 genes, 0.8% | 1 | View Result |
| superoxide dismutase activity | 7 out of 7474 genes, 0.1% | 33 out of 40099 genes, 0.1% | 1 | View Result |
| oxidoreductase activity, acting on superoxide radicals as acceptor | 7 out of 7474 genes, 0.1% | 33 out of 40099 genes, 0.1% | 1 | View Result |
| myo-inositol transmembrane transporter activity | 6 out of 7474 genes, 0.1% | 28 out of 40099 genes, 0.1% | 1 | View Result |
| phosphotransferase activity, phosphate group as acceptor | 19 out of 7474 genes, 0.3% | 96 out of 40099 genes, 0.2% | 1 | View Result |
| Ran GTPase binding | 10 out of 7474 genes, 0.1% | 49 out of 40099 genes, 0.1% | 1 | View Result |
| potassium ion binding | 10 out of 7474 genes, 0.1% | 49 out of 40099 genes, 0.1% | 1 | View Result |
| alkali metal ion binding | 10 out of 7474 genes, 0.1% | 49 out of 40099 genes, 0.1% | 1 | View Result |
| serine-type carboxypeptidase activity | 14 out of 7474 genes, 0.2% | 70 out of 40099 genes, 0.2% | 1 | View Result |
| 3-oxoacyl-[acyl-carrier-protein] synthase activity | 5 out of 7474 genes, 0.1% | 23 out of 40099 genes, 0.1% | 1 | View Result |
| telomeric DNA binding | 5 out of 7474 genes, 0.1% | 23 out of 40099 genes, 0.1% | 1 | View Result |
| flavonol synthase activity | 5 out of 7474 genes, 0.1% | 23 out of 40099 genes, 0.1% | 1 | View Result |
| oxidoreductase activity, acting on a sulfur group of donors, NAD(P) as acceptor | 9 out of 7474 genes, 0.1% | 44 out of 40099 genes, 0.1% | 1 | View Result |
| oxidoreductase activity, acting on the CH-NH group of donors, oxygen as acceptor | 4 out of 7474 genes, 0.1% | 18 out of 40099 genes, 0.0% | 1 | View Result |
| oxidoreductase activity, acting on X-H and Y-H to form an X-Y bond | 4 out of 7474 genes, 0.1% | 18 out of 40099 genes, 0.0% | 1 | View Result |
| oxidoreductase activity, acting on X-H and Y-H to form an X-Y bond, with oxygen as acceptor | 4 out of 7474 genes, 0.1% | 18 out of 40099 genes, 0.0% | 1 | View Result |
| anthocyanidin 3-O-glucosyltransferase activity | 4 out of 7474 genes, 0.1% | 18 out of 40099 genes, 0.0% | 1 | View Result |
| Ras GTPase binding | 12 out of 7474 genes, 0.2% | 60 out of 40099 genes, 0.1% | 1 | View Result |
| small GTPase binding | 12 out of 7474 genes, 0.2% | 60 out of 40099 genes, 0.1% | 1 | View Result |
| guanylate kinase activity | 3 out of 7474 genes, 0.0% | 13 out of 40099 genes, 0.0% | 1 | View Result |
| isocitrate dehydrogenase (NAD+) activity | 3 out of 7474 genes, 0.0% | 13 out of 40099 genes, 0.0% | 1 | View Result |
| tryptophan synthase activity | 3 out of 7474 genes, 0.0% | 13 out of 40099 genes, 0.0% | 1 | View Result |
| ammonium transmembrane transporter activity | 3 out of 7474 genes, 0.0% | 13 out of 40099 genes, 0.0% | 1 | View Result |
| arginine decarboxylase activity | 3 out of 7474 genes, 0.0% | 13 out of 40099 genes, 0.0% | 1 | View Result |
| organic cation transmembrane transporter activity | 3 out of 7474 genes, 0.0% | 13 out of 40099 genes, 0.0% | 1 | View Result |
| polyamine transmembrane transporter activity | 3 out of 7474 genes, 0.0% | 13 out of 40099 genes, 0.0% | 1 | View Result |
| calcium:cation antiporter activity | 3 out of 7474 genes, 0.0% | 13 out of 40099 genes, 0.0% | 1 | View Result |
| molybdate transmembrane-transporting ATPase activity | 3 out of 7474 genes, 0.0% | 13 out of 40099 genes, 0.0% | 1 | View Result |
| flavonoid 3',5'-hydroxylase activity | 3 out of 7474 genes, 0.0% | 13 out of 40099 genes, 0.0% | 1 | View Result |
| glucan 1,4-alpha-maltotetraohydrolase activity | 3 out of 7474 genes, 0.0% | 13 out of 40099 genes, 0.0% | 1 | View Result |
| UDP-arabinopyranose mutase activity | 3 out of 7474 genes, 0.0% | 13 out of 40099 genes, 0.0% | 1 | View Result |
| cation:cation antiporter activity | 15 out of 7474 genes, 0.2% | 76 out of 40099 genes, 0.2% | 1 | View Result |
| DNA helicase activity | 18 out of 7474 genes, 0.2% | 92 out of 40099 genes, 0.2% | 1 | View Result |
| 3-beta-hydroxy-delta5-steroid dehydrogenase activity | 2 out of 7474 genes, 0.0% | 8 out of 40099 genes, 0.0% | 1 | View Result |
| ethanolamine kinase activity | 2 out of 7474 genes, 0.0% | 8 out of 40099 genes, 0.0% | 1 | View Result |
| beta-glucuronidase activity | 2 out of 7474 genes, 0.0% | 8 out of 40099 genes, 0.0% | 1 | View Result |
| polyribonucleotide nucleotidyltransferase activity | 2 out of 7474 genes, 0.0% | 8 out of 40099 genes, 0.0% | 1 | View Result |
| protein geranylgeranyltransferase activity | 2 out of 7474 genes, 0.0% | 8 out of 40099 genes, 0.0% | 1 | View Result |
| alanine-glyoxylate transaminase activity | 2 out of 7474 genes, 0.0% | 8 out of 40099 genes, 0.0% | 1 | View Result |
| amino-terminal vacuolar sorting propeptide binding | 2 out of 7474 genes, 0.0% | 8 out of 40099 genes, 0.0% | 1 | View Result |
| silicate transmembrane transporter activity | 2 out of 7474 genes, 0.0% | 8 out of 40099 genes, 0.0% | 1 | View Result |
| oxidoreductase activity, acting on iron-sulfur proteins as donors, NAD or NADP as acceptor | 2 out of 7474 genes, 0.0% | 8 out of 40099 genes, 0.0% | 1 | View Result |
| carboxyl- or carbamoyltransferase activity | 2 out of 7474 genes, 0.0% | 8 out of 40099 genes, 0.0% | 1 | View Result |
| amine-lyase activity | 2 out of 7474 genes, 0.0% | 8 out of 40099 genes, 0.0% | 1 | View Result |
| strictosidine synthase activity | 2 out of 7474 genes, 0.0% | 8 out of 40099 genes, 0.0% | 1 | View Result |
| oxidoreductase activity, acting on the CH-OH group of donors, quinone or similar compound as acceptor | 2 out of 7474 genes, 0.0% | 8 out of 40099 genes, 0.0% | 1 | View Result |
| coniferyl-aldehyde dehydrogenase activity | 2 out of 7474 genes, 0.0% | 8 out of 40099 genes, 0.0% | 1 | View Result |
| inositol monophosphate 3-phosphatase activity | 2 out of 7474 genes, 0.0% | 8 out of 40099 genes, 0.0% | 1 | View Result |
| inositol monophosphate 4-phosphatase activity | 2 out of 7474 genes, 0.0% | 8 out of 40099 genes, 0.0% | 1 | View Result |
| pyruvate kinase activity | 10 out of 7474 genes, 0.1% | 50 out of 40099 genes, 0.1% | 1 | View Result |
| malate dehydrogenase activity | 20 out of 7474 genes, 0.3% | 103 out of 40099 genes, 0.3% | 1 | View Result |
| succinate dehydrogenase activity | 6 out of 7474 genes, 0.1% | 29 out of 40099 genes, 0.1% | 1 | View Result |
| protein phosphorylated amino acid binding | 6 out of 7474 genes, 0.1% | 29 out of 40099 genes, 0.1% | 1 | View Result |
| phosphoprotein binding | 6 out of 7474 genes, 0.1% | 29 out of 40099 genes, 0.1% | 1 | View Result |
| serine-type endopeptidase activity | 39 out of 7474 genes, 0.5% | 205 out of 40099 genes, 0.5% | 1 | View Result |
| acetyl-CoA C-acyltransferase activity | 5 out of 7474 genes, 0.1% | 24 out of 40099 genes, 0.1% | 1 | View Result |
| aldehyde dehydrogenase [NAD(P)+] activity | 5 out of 7474 genes, 0.1% | 24 out of 40099 genes, 0.1% | 1 | View Result |
| anthranilate phosphoribosyltransferase activity | 5 out of 7474 genes, 0.1% | 24 out of 40099 genes, 0.1% | 1 | View Result |
| acidic amino acid transmembrane transporter activity | 8 out of 7474 genes, 0.1% | 40 out of 40099 genes, 0.1% | 1 | View Result |
| monovalent cation:hydrogen antiporter activity | 11 out of 7474 genes, 0.1% | 56 out of 40099 genes, 0.1% | 1 | View Result |
| plastid sigma factor activity | 4 out of 7474 genes, 0.1% | 19 out of 40099 genes, 0.0% | 1 | View Result |
| succinate-semialdehyde dehydrogenase (NAD+) activity | 4 out of 7474 genes, 0.1% | 19 out of 40099 genes, 0.0% | 1 | View Result |
| polyol transmembrane transporter activity | 7 out of 7474 genes, 0.1% | 35 out of 40099 genes, 0.1% | 1 | View Result |
| steroid dehydrogenase activity, acting on the CH-OH group of donors, NAD or NADP as acceptor | 7 out of 7474 genes, 0.1% | 35 out of 40099 genes, 0.1% | 1 | View Result |
| oxidoreductase activity, acting on single donors with incorporation of molecular oxygen | 34 out of 7474 genes, 0.5% | 180 out of 40099 genes, 0.4% | 1 | View Result |
| 6-phosphofructokinase activity | 6 out of 7474 genes, 0.1% | 30 out of 40099 genes, 0.1% | 1 | View Result |
| glutathione transferase activity | 6 out of 7474 genes, 0.1% | 30 out of 40099 genes, 0.1% | 1 | View Result |
| guanylyltransferase activity | 6 out of 7474 genes, 0.1% | 30 out of 40099 genes, 0.1% | 1 | View Result |
| glycogen phosphorylase activity | 3 out of 7474 genes, 0.0% | 14 out of 40099 genes, 0.0% | 1 | View Result |
| arginine transmembrane transporter activity | 3 out of 7474 genes, 0.0% | 14 out of 40099 genes, 0.0% | 1 | View Result |
| L-lysine transmembrane transporter activity | 3 out of 7474 genes, 0.0% | 14 out of 40099 genes, 0.0% | 1 | View Result |
| heme transporter activity | 3 out of 7474 genes, 0.0% | 14 out of 40099 genes, 0.0% | 1 | View Result |
| heme-transporting ATPase activity | 3 out of 7474 genes, 0.0% | 14 out of 40099 genes, 0.0% | 1 | View Result |
| oxidoreductase activity, acting on CH or CH2 groups, NAD or NADP as acceptor | 3 out of 7474 genes, 0.0% | 14 out of 40099 genes, 0.0% | 1 | View Result |
| transcription coactivator activity | 5 out of 7474 genes, 0.1% | 25 out of 40099 genes, 0.1% | 1 | View Result |
| ribokinase activity | 5 out of 7474 genes, 0.1% | 25 out of 40099 genes, 0.1% | 1 | View Result |
| cysteine-type endopeptidase activity | 10 out of 7474 genes, 0.1% | 52 out of 40099 genes, 0.1% | 1 | View Result |
| oxidoreductase activity, acting on paired donors, with oxidation of a pair of donors resulting in the reduction of molecular oxygen to two molecules of water | 10 out of 7474 genes, 0.1% | 52 out of 40099 genes, 0.1% | 1 | View Result |
| oxidoreductase activity, acting on paired donors, with incorporation or reduction of molecular oxygen, 2-oxoglutarate as one donor, and incorporation of one atom each of oxygen into both donors | 36 out of 7474 genes, 0.5% | 192 out of 40099 genes, 0.5% | 1 | View Result |
| anion transmembrane transporter activity | 123 out of 7474 genes, 1.6% | 660 out of 40099 genes, 1.6% | 1 | View Result |
| arsenite transmembrane transporter activity | 7 out of 7474 genes, 0.1% | 36 out of 40099 genes, 0.1% | 1 | View Result |
| alcohol transmembrane transporter activity | 7 out of 7474 genes, 0.1% | 36 out of 40099 genes, 0.1% | 1 | View Result |
| monosaccharide binding | 7 out of 7474 genes, 0.1% | 36 out of 40099 genes, 0.1% | 1 | View Result |
| organic hydroxy compound transmembrane transporter activity | 7 out of 7474 genes, 0.1% | 36 out of 40099 genes, 0.1% | 1 | View Result |
| DNA (cytosine-5-)-methyltransferase activity | 2 out of 7474 genes, 0.0% | 9 out of 40099 genes, 0.0% | 1 | View Result |
| carbonyl reductase (NADPH) activity | 2 out of 7474 genes, 0.0% | 9 out of 40099 genes, 0.0% | 1 | View Result |
| formamidase activity | 2 out of 7474 genes, 0.0% | 9 out of 40099 genes, 0.0% | 1 | View Result |
| glucose-6-phosphate isomerase activity | 2 out of 7474 genes, 0.0% | 9 out of 40099 genes, 0.0% | 1 | View Result |
| 1-deoxy-D-xylulose-5-phosphate synthase activity | 2 out of 7474 genes, 0.0% | 9 out of 40099 genes, 0.0% | 1 | View Result |
| carbon-monoxide oxygenase activity | 2 out of 7474 genes, 0.0% | 9 out of 40099 genes, 0.0% | 1 | View Result |
| inositol monophosphate 1-phosphatase activity | 2 out of 7474 genes, 0.0% | 9 out of 40099 genes, 0.0% | 1 | View Result |
| DNA-methyltransferase activity | 2 out of 7474 genes, 0.0% | 9 out of 40099 genes, 0.0% | 1 | View Result |
| vacuolar sorting signal binding | 2 out of 7474 genes, 0.0% | 9 out of 40099 genes, 0.0% | 1 | View Result |
| oxidoreductase activity, acting on the aldehyde or oxo group of donors, cytochrome as acceptor | 2 out of 7474 genes, 0.0% | 9 out of 40099 genes, 0.0% | 1 | View Result |
| 12-oxophytodienoate reductase activity | 2 out of 7474 genes, 0.0% | 9 out of 40099 genes, 0.0% | 1 | View Result |
| DEAD/H-box RNA helicase binding | 2 out of 7474 genes, 0.0% | 9 out of 40099 genes, 0.0% | 1 | View Result |
| GDP binding | 2 out of 7474 genes, 0.0% | 9 out of 40099 genes, 0.0% | 1 | View Result |
| cytokinin dehydrogenase activity | 2 out of 7474 genes, 0.0% | 9 out of 40099 genes, 0.0% | 1 | View Result |
| kinase inhibitor activity | 2 out of 7474 genes, 0.0% | 9 out of 40099 genes, 0.0% | 1 | View Result |
| structural constituent of chromatin | 2 out of 7474 genes, 0.0% | 9 out of 40099 genes, 0.0% | 1 | View Result |
| sucrose-phosphate synthase activity | 2 out of 7474 genes, 0.0% | 9 out of 40099 genes, 0.0% | 1 | View Result |
| pectinesterase inhibitor activity | 2 out of 7474 genes, 0.0% | 9 out of 40099 genes, 0.0% | 1 | View Result |
| 4-hydroxy-3-methylbut-2-en-1-yl diphosphate reductase activity | 2 out of 7474 genes, 0.0% | 9 out of 40099 genes, 0.0% | 1 | View Result |
| inositol monophosphate phosphatase activity | 2 out of 7474 genes, 0.0% | 9 out of 40099 genes, 0.0% | 1 | View Result |
| carbohydrate derivative transporter activity | 21 out of 7474 genes, 0.3% | 112 out of 40099 genes, 0.3% | 1 | View Result |
| transmembrane transporter activity | 518 out of 7474 genes, 6.9% | 2784 out of 40099 genes, 6.9% | 1 | View Result |
| dTDP-4-dehydrorhamnose reductase activity | 4 out of 7474 genes, 0.1% | 20 out of 40099 genes, 0.0% | 1 | View Result |
| indole-3-acetic acid amido synthetase activity | 4 out of 7474 genes, 0.1% | 20 out of 40099 genes, 0.0% | 1 | View Result |
| pyridoxine:NADP 4-dehydrogenase activity | 4 out of 7474 genes, 0.1% | 20 out of 40099 genes, 0.0% | 1 | View Result |
| secondary active sulfate transmembrane transporter activity | 8 out of 7474 genes, 0.1% | 42 out of 40099 genes, 0.1% | 1 | View Result |
| phosphorelay sensor kinase activity | 10 out of 7474 genes, 0.1% | 53 out of 40099 genes, 0.1% | 1 | View Result |
| CoA hydrolase activity | 10 out of 7474 genes, 0.1% | 53 out of 40099 genes, 0.1% | 1 | View Result |
| ARF GTPase activator activity | 5 out of 7474 genes, 0.1% | 26 out of 40099 genes, 0.1% | 1 | View Result |
| phosphatidate phosphatase activity | 5 out of 7474 genes, 0.1% | 26 out of 40099 genes, 0.1% | 1 | View Result |
| small conjugating protein binding | 7 out of 7474 genes, 0.1% | 37 out of 40099 genes, 0.1% | 1 | View Result |
| ubiquitin binding | 7 out of 7474 genes, 0.1% | 37 out of 40099 genes, 0.1% | 1 | View Result |
| GPI-anchor transamidase activity | 3 out of 7474 genes, 0.0% | 15 out of 40099 genes, 0.0% | 1 | View Result |
| ferric iron binding | 3 out of 7474 genes, 0.0% | 15 out of 40099 genes, 0.0% | 1 | View Result |
| starch synthase activity | 3 out of 7474 genes, 0.0% | 15 out of 40099 genes, 0.0% | 1 | View Result |
| (+)-abscisic acid 8'-hydroxylase activity | 3 out of 7474 genes, 0.0% | 15 out of 40099 genes, 0.0% | 1 | View Result |
| ADP transmembrane transporter activity | 3 out of 7474 genes, 0.0% | 15 out of 40099 genes, 0.0% | 1 | View Result |
| peptide-transporting ATPase activity | 3 out of 7474 genes, 0.0% | 15 out of 40099 genes, 0.0% | 1 | View Result |
| trans-2-enoyl-CoA reductase (NADPH) activity | 3 out of 7474 genes, 0.0% | 15 out of 40099 genes, 0.0% | 1 | View Result |
| primary active transmembrane transporter activity | 186 out of 7474 genes, 2.5% | 1005 out of 40099 genes, 2.5% | 1 | View Result |
| acid phosphatase activity | 19 out of 7474 genes, 0.3% | 103 out of 40099 genes, 0.3% | 1 | View Result |
| nucleoside transmembrane transporter activity | 12 out of 7474 genes, 0.2% | 65 out of 40099 genes, 0.2% | 1 | View Result |
| amidase activity | 4 out of 7474 genes, 0.1% | 21 out of 40099 genes, 0.1% | 1 | View Result |
| sphingomyelin phosphodiesterase activity | 4 out of 7474 genes, 0.1% | 21 out of 40099 genes, 0.1% | 1 | View Result |
| tRNA methyltransferase activity | 4 out of 7474 genes, 0.1% | 21 out of 40099 genes, 0.1% | 1 | View Result |
| auxin binding | 4 out of 7474 genes, 0.1% | 21 out of 40099 genes, 0.1% | 1 | View Result |
| phosphate transmembrane transporter activity | 23 out of 7474 genes, 0.3% | 126 out of 40099 genes, 0.3% | 1 | View Result |
| oxidoreductase activity, acting on single donors with incorporation of molecular oxygen, incorporation of two atoms of oxygen | 25 out of 7474 genes, 0.3% | 137 out of 40099 genes, 0.3% | 1 | View Result |
| nitrilase activity | 2 out of 7474 genes, 0.0% | 10 out of 40099 genes, 0.0% | 1 | View Result |
| RNA-directed RNA polymerase activity | 2 out of 7474 genes, 0.0% | 10 out of 40099 genes, 0.0% | 1 | View Result |
| alditol:NADP+ 1-oxidoreductase activity | 2 out of 7474 genes, 0.0% | 10 out of 40099 genes, 0.0% | 1 | View Result |
| glycogen debranching enzyme activity | 2 out of 7474 genes, 0.0% | 10 out of 40099 genes, 0.0% | 1 | View Result |
| hydroxyacylglutathione hydrolase activity | 2 out of 7474 genes, 0.0% | 10 out of 40099 genes, 0.0% | 1 | View Result |
| hydroxymethylglutaryl-CoA lyase activity | 2 out of 7474 genes, 0.0% | 10 out of 40099 genes, 0.0% | 1 | View Result |
| ribonuclease III activity | 2 out of 7474 genes, 0.0% | 10 out of 40099 genes, 0.0% | 1 | View Result |
| G-protein coupled GABA receptor activity | 2 out of 7474 genes, 0.0% | 10 out of 40099 genes, 0.0% | 1 | View Result |
| protein prenyltransferase activity | 2 out of 7474 genes, 0.0% | 10 out of 40099 genes, 0.0% | 1 | View Result |
| high affinity secondary active ammonium transmembrane transporter activity | 2 out of 7474 genes, 0.0% | 10 out of 40099 genes, 0.0% | 1 | View Result |
| dipeptidase activity | 2 out of 7474 genes, 0.0% | 10 out of 40099 genes, 0.0% | 1 | View Result |
| hydrolase activity, acting on carbon-nitrogen (but not peptide) bonds, in nitriles | 2 out of 7474 genes, 0.0% | 10 out of 40099 genes, 0.0% | 1 | View Result |
| GABA receptor activity | 2 out of 7474 genes, 0.0% | 10 out of 40099 genes, 0.0% | 1 | View Result |
| D-erythro-sphingosine kinase activity | 2 out of 7474 genes, 0.0% | 10 out of 40099 genes, 0.0% | 1 | View Result |
| tRNA dihydrouridine synthase activity | 2 out of 7474 genes, 0.0% | 10 out of 40099 genes, 0.0% | 1 | View Result |
| hydroperoxide dehydratase activity | 2 out of 7474 genes, 0.0% | 10 out of 40099 genes, 0.0% | 1 | View Result |
| alpha-glucan, water dikinase activity | 2 out of 7474 genes, 0.0% | 10 out of 40099 genes, 0.0% | 1 | View Result |
| malate dehydrogenase (oxaloacetate-decarboxylating) activity | 5 out of 7474 genes, 0.1% | 27 out of 40099 genes, 0.1% | 1 | View Result |
| poly(A)-specific ribonuclease activity | 8 out of 7474 genes, 0.1% | 44 out of 40099 genes, 0.1% | 1 | View Result |
| phosphofructokinase activity | 8 out of 7474 genes, 0.1% | 44 out of 40099 genes, 0.1% | 1 | View Result |
| ribonucleoprotein complex binding | 8 out of 7474 genes, 0.1% | 44 out of 40099 genes, 0.1% | 1 | View Result |
| thiolester hydrolase activity | 32 out of 7474 genes, 0.4% | 176 out of 40099 genes, 0.4% | 1 | View Result |
| pyridoxal phosphate binding | 54 out of 7474 genes, 0.7% | 296 out of 40099 genes, 0.7% | 1 | View Result |
| fatty-acyl-CoA binding | 3 out of 7474 genes, 0.0% | 16 out of 40099 genes, 0.0% | 1 | View Result |
| double-stranded telomeric DNA binding | 3 out of 7474 genes, 0.0% | 16 out of 40099 genes, 0.0% | 1 | View Result |
| triose-phosphate transmembrane transporter activity | 3 out of 7474 genes, 0.0% | 16 out of 40099 genes, 0.0% | 1 | View Result |
| DNA-dependent ATPase activity | 21 out of 7474 genes, 0.3% | 116 out of 40099 genes, 0.3% | 1 | View Result |
| P-P-bond-hydrolysis-driven transmembrane transporter activity | 184 out of 7474 genes, 2.5% | 1002 out of 40099 genes, 2.5% | 1 | View Result |
| glucosyltransferase activity | 93 out of 7474 genes, 1.2% | 509 out of 40099 genes, 1.3% | 1 | View Result |
| UDP-glucosyltransferase activity | 88 out of 7474 genes, 1.2% | 482 out of 40099 genes, 1.2% | 1 | View Result |
| cation channel activity | 18 out of 7474 genes, 0.2% | 100 out of 40099 genes, 0.2% | 1 | View Result |
| oxidoreductase activity, acting on a sulfur group of donors | 49 out of 7474 genes, 0.7% | 270 out of 40099 genes, 0.7% | 1 | View Result |
| galactolipase activity | 4 out of 7474 genes, 0.1% | 22 out of 40099 genes, 0.1% | 1 | View Result |
| actin binding | 49 out of 7474 genes, 0.7% | 271 out of 40099 genes, 0.7% | 1 | View Result |
| ligand-gated ion channel activity | 13 out of 7474 genes, 0.2% | 73 out of 40099 genes, 0.2% | 1 | View Result |
| ligand-gated channel activity | 13 out of 7474 genes, 0.2% | 73 out of 40099 genes, 0.2% | 1 | View Result |
| dioxygenase activity | 55 out of 7474 genes, 0.7% | 304 out of 40099 genes, 0.8% | 1 | View Result |
| endopeptidase activity | 115 out of 7474 genes, 1.5% | 631 out of 40099 genes, 1.6% | 1 | View Result |
| protein tyrosine phosphatase activity | 11 out of 7474 genes, 0.1% | 62 out of 40099 genes, 0.2% | 1 | View Result |
| cellulose synthase activity | 18 out of 7474 genes, 0.2% | 101 out of 40099 genes, 0.3% | 1 | View Result |
| translation release factor activity, codon specific | 6 out of 7474 genes, 0.1% | 34 out of 40099 genes, 0.1% | 1 | View Result |
| RNA cap binding | 2 out of 7474 genes, 0.0% | 11 out of 40099 genes, 0.0% | 1 | View Result |
| RNA polymerase II regulatory region sequence-specific DNA binding | 2 out of 7474 genes, 0.0% | 11 out of 40099 genes, 0.0% | 1 | View Result |
| RNA polymerase II core promoter sequence-specific DNA binding | 2 out of 7474 genes, 0.0% | 11 out of 40099 genes, 0.0% | 1 | View Result |
| RNA polymerase II regulatory region DNA binding | 2 out of 7474 genes, 0.0% | 11 out of 40099 genes, 0.0% | 1 | View Result |
| core promoter sequence-specific DNA binding | 2 out of 7474 genes, 0.0% | 11 out of 40099 genes, 0.0% | 1 | View Result |
| N-acetyl-gamma-glutamyl-phosphate reductase activity | 2 out of 7474 genes, 0.0% | 11 out of 40099 genes, 0.0% | 1 | View Result |
| glycylpeptide N-tetradecanoyltransferase activity | 2 out of 7474 genes, 0.0% | 11 out of 40099 genes, 0.0% | 1 | View Result |
| guanylate cyclase activity | 2 out of 7474 genes, 0.0% | 11 out of 40099 genes, 0.0% | 1 | View Result |
| ribonuclease H activity | 2 out of 7474 genes, 0.0% | 11 out of 40099 genes, 0.0% | 1 | View Result |
| phosphoglycerate dehydrogenase activity | 2 out of 7474 genes, 0.0% | 11 out of 40099 genes, 0.0% | 1 | View Result |
| 1,4-dihydroxy-2-naphthoyl-CoA synthase activity | 2 out of 7474 genes, 0.0% | 11 out of 40099 genes, 0.0% | 1 | View Result |
| carotene beta-ring hydroxylase activity | 2 out of 7474 genes, 0.0% | 11 out of 40099 genes, 0.0% | 1 | View Result |
| phosphate ion transmembrane-transporting ATPase activity | 2 out of 7474 genes, 0.0% | 11 out of 40099 genes, 0.0% | 1 | View Result |
| sulfite reductase activity | 2 out of 7474 genes, 0.0% | 11 out of 40099 genes, 0.0% | 1 | View Result |
| oxidoreductase activity, acting on a sulfur group of donors, iron-sulfur protein as acceptor | 2 out of 7474 genes, 0.0% | 11 out of 40099 genes, 0.0% | 1 | View Result |
| myristoyltransferase activity | 2 out of 7474 genes, 0.0% | 11 out of 40099 genes, 0.0% | 1 | View Result |
| polyamine oxidase activity | 2 out of 7474 genes, 0.0% | 11 out of 40099 genes, 0.0% | 1 | View Result |
| sulfite reductase (ferredoxin) activity | 2 out of 7474 genes, 0.0% | 11 out of 40099 genes, 0.0% | 1 | View Result |
| 3-hydroxyacyl-CoA dehydrogenase activity | 3 out of 7474 genes, 0.0% | 17 out of 40099 genes, 0.0% | 1 | View Result |
| siRNA binding | 3 out of 7474 genes, 0.0% | 17 out of 40099 genes, 0.0% | 1 | View Result |
| alpha-N-arabinofuranosidase activity | 3 out of 7474 genes, 0.0% | 17 out of 40099 genes, 0.0% | 1 | View Result |
| NADH dehydrogenase (quinone) activity | 18 out of 7474 genes, 0.2% | 102 out of 40099 genes, 0.3% | 1 | View Result |
| DNA polymerase activity | 25 out of 7474 genes, 0.3% | 141 out of 40099 genes, 0.4% | 1 | View Result |
| ATP citrate synthase activity | 4 out of 7474 genes, 0.1% | 23 out of 40099 genes, 0.1% | 1 | View Result |
| 1-phosphatidylinositol 4-kinase activity | 4 out of 7474 genes, 0.1% | 23 out of 40099 genes, 0.1% | 1 | View Result |
| phosphoglycerate mutase activity | 4 out of 7474 genes, 0.1% | 23 out of 40099 genes, 0.1% | 1 | View Result |
| fatty acid elongase activity | 4 out of 7474 genes, 0.1% | 23 out of 40099 genes, 0.1% | 1 | View Result |
| oligosaccharyl transferase activity | 5 out of 7474 genes, 0.1% | 29 out of 40099 genes, 0.1% | 1 | View Result |
| nucleobase-containing compound kinase activity | 24 out of 7474 genes, 0.3% | 136 out of 40099 genes, 0.3% | 1 | View Result |
| transcription factor binding | 9 out of 7474 genes, 0.1% | 52 out of 40099 genes, 0.1% | 1 | View Result |
| histone binding | 15 out of 7474 genes, 0.2% | 86 out of 40099 genes, 0.2% | 1 | View Result |
| fatty acid ligase activity | 6 out of 7474 genes, 0.1% | 35 out of 40099 genes, 0.1% | 1 | View Result |
| sulfur compound binding | 6 out of 7474 genes, 0.1% | 35 out of 40099 genes, 0.1% | 1 | View Result |
| racemase and epimerase activity | 25 out of 7474 genes, 0.3% | 142 out of 40099 genes, 0.4% | 1 | View Result |
| carbon-oxygen lyase activity | 91 out of 7474 genes, 1.2% | 505 out of 40099 genes, 1.3% | 1 | View Result |
| proton-transporting ATPase activity, rotational mechanism | 16 out of 7474 genes, 0.2% | 92 out of 40099 genes, 0.2% | 1 | View Result |
| C-acyltransferase activity | 7 out of 7474 genes, 0.1% | 41 out of 40099 genes, 0.1% | 1 | View Result |
| peptidyl-prolyl cis-trans isomerase activity | 24 out of 7474 genes, 0.3% | 137 out of 40099 genes, 0.3% | 1 | View Result |
| carboxypeptidase activity | 24 out of 7474 genes, 0.3% | 137 out of 40099 genes, 0.3% | 1 | View Result |
| cellulose synthase (UDP-forming) activity | 17 out of 7474 genes, 0.2% | 98 out of 40099 genes, 0.2% | 1 | View Result |
| hydro-lyase activity | 62 out of 7474 genes, 0.8% | 348 out of 40099 genes, 0.9% | 1 | View Result |
| core promoter binding | 3 out of 7474 genes, 0.0% | 18 out of 40099 genes, 0.0% | 1 | View Result |
| adenylate kinase activity | 3 out of 7474 genes, 0.0% | 18 out of 40099 genes, 0.0% | 1 | View Result |
| asparagine synthase (glutamine-hydrolyzing) activity | 3 out of 7474 genes, 0.0% | 18 out of 40099 genes, 0.0% | 1 | View Result |
| phosphatidylinositol phospholipase C activity | 3 out of 7474 genes, 0.0% | 18 out of 40099 genes, 0.0% | 1 | View Result |
| phosphoglycolate phosphatase activity | 3 out of 7474 genes, 0.0% | 18 out of 40099 genes, 0.0% | 1 | View Result |
| endo-1,4-beta-xylanase activity | 3 out of 7474 genes, 0.0% | 18 out of 40099 genes, 0.0% | 1 | View Result |
| vitamin transporter activity | 3 out of 7474 genes, 0.0% | 18 out of 40099 genes, 0.0% | 1 | View Result |
| transmembrane receptor protein serine/threonine kinase activity | 4 out of 7474 genes, 0.1% | 24 out of 40099 genes, 0.1% | 1 | View Result |
| MAP kinase kinase activity | 4 out of 7474 genes, 0.1% | 24 out of 40099 genes, 0.1% | 1 | View Result |
| calcium-dependent protein serine/threonine phosphatase activity | 4 out of 7474 genes, 0.1% | 24 out of 40099 genes, 0.1% | 1 | View Result |
| auxin:hydrogen symporter activity | 4 out of 7474 genes, 0.1% | 24 out of 40099 genes, 0.1% | 1 | View Result |
| 4-hydroxy-3-methylbut-2-en-1-yl diphosphate synthase activity | 4 out of 7474 genes, 0.1% | 24 out of 40099 genes, 0.1% | 1 | View Result |
| oxidoreductase activity, acting on CH or CH2 groups | 16 out of 7474 genes, 0.2% | 93 out of 40099 genes, 0.2% | 1 | View Result |
| TBP-class protein binding | 5 out of 7474 genes, 0.1% | 30 out of 40099 genes, 0.1% | 1 | View Result |
| hydroxymethylglutaryl-CoA reductase (NADPH) activity | 2 out of 7474 genes, 0.0% | 12 out of 40099 genes, 0.0% | 1 | View Result |
| mannose-1-phosphate guanylyltransferase activity | 2 out of 7474 genes, 0.0% | 12 out of 40099 genes, 0.0% | 1 | View Result |
| inositol-3-phosphate synthase activity | 2 out of 7474 genes, 0.0% | 12 out of 40099 genes, 0.0% | 1 | View Result |
| poly(A) RNA binding | 2 out of 7474 genes, 0.0% | 12 out of 40099 genes, 0.0% | 1 | View Result |
| 5-amino-6-(5-phosphoribosylamino)uracil reductase activity | 2 out of 7474 genes, 0.0% | 12 out of 40099 genes, 0.0% | 1 | View Result |
| dTDP-4-dehydrorhamnose 3,5-epimerase activity | 2 out of 7474 genes, 0.0% | 12 out of 40099 genes, 0.0% | 1 | View Result |
| citrate transmembrane transporter activity | 2 out of 7474 genes, 0.0% | 12 out of 40099 genes, 0.0% | 1 | View Result |
| tricarboxylic acid transmembrane transporter activity | 2 out of 7474 genes, 0.0% | 12 out of 40099 genes, 0.0% | 1 | View Result |
| oxidoreductase activity, acting on NAD(P)H, NAD(P) as acceptor | 2 out of 7474 genes, 0.0% | 12 out of 40099 genes, 0.0% | 1 | View Result |
| snoRNA binding | 2 out of 7474 genes, 0.0% | 12 out of 40099 genes, 0.0% | 1 | View Result |
| bis(5'-adenosyl)-pentaphosphatase activity | 2 out of 7474 genes, 0.0% | 12 out of 40099 genes, 0.0% | 1 | View Result |
| taxane 13-alpha-hydroxylase activity | 2 out of 7474 genes, 0.0% | 12 out of 40099 genes, 0.0% | 1 | View Result |
| carbon-nitrogen ligase activity, with glutamine as amido-N-donor | 14 out of 7474 genes, 0.2% | 82 out of 40099 genes, 0.2% | 1 | View Result |
| damaged DNA binding | 11 out of 7474 genes, 0.1% | 65 out of 40099 genes, 0.2% | 1 | View Result |
| voltage-gated ion channel activity | 23 out of 7474 genes, 0.3% | 133 out of 40099 genes, 0.3% | 1 | View Result |
| voltage-gated channel activity | 23 out of 7474 genes, 0.3% | 133 out of 40099 genes, 0.3% | 1 | View Result |
| transferase activity, transferring glycosyl groups | 306 out of 7474 genes, 4.1% | 1681 out of 40099 genes, 4.2% | 1 | View Result |
| protein tyrosine/serine/threonine phosphatase activity | 8 out of 7474 genes, 0.1% | 48 out of 40099 genes, 0.1% | 1 | View Result |
| cyclic nucleotide binding | 9 out of 7474 genes, 0.1% | 54 out of 40099 genes, 0.1% | 1 | View Result |
| NADH dehydrogenase (ubiquinone) activity | 17 out of 7474 genes, 0.2% | 100 out of 40099 genes, 0.2% | 1 | View Result |
| nucleobase-containing compound transmembrane transporter activity | 23 out of 7474 genes, 0.3% | 134 out of 40099 genes, 0.3% | 1 | View Result |
| racemase and epimerase activity, acting on carbohydrates and derivatives | 21 out of 7474 genes, 0.3% | 123 out of 40099 genes, 0.3% | 1 | View Result |
| DNA photolyase activity | 4 out of 7474 genes, 0.1% | 25 out of 40099 genes, 0.1% | 1 | View Result |
| UDP-glucuronate decarboxylase activity | 4 out of 7474 genes, 0.1% | 25 out of 40099 genes, 0.1% | 1 | View Result |
| hydrolase activity, acting on ether bonds | 6 out of 7474 genes, 0.1% | 37 out of 40099 genes, 0.1% | 1 | View Result |
| endoribonuclease activity | 19 out of 7474 genes, 0.3% | 112 out of 40099 genes, 0.3% | 1 | View Result |
| inositol-polyphosphate 5-phosphatase activity | 3 out of 7474 genes, 0.0% | 19 out of 40099 genes, 0.0% | 1 | View Result |
| phosphatidylcholine-sterol O-acyltransferase activity | 3 out of 7474 genes, 0.0% | 19 out of 40099 genes, 0.0% | 1 | View Result |
| uracil phosphoribosyltransferase activity | 3 out of 7474 genes, 0.0% | 19 out of 40099 genes, 0.0% | 1 | View Result |
| DNA N-glycosylase activity | 3 out of 7474 genes, 0.0% | 19 out of 40099 genes, 0.0% | 1 | View Result |
| myrcene synthase activity | 3 out of 7474 genes, 0.0% | 19 out of 40099 genes, 0.0% | 1 | View Result |
| structure-specific DNA binding | 23 out of 7474 genes, 0.3% | 135 out of 40099 genes, 0.3% | 1 | View Result |
| pattern binding | 9 out of 7474 genes, 0.1% | 55 out of 40099 genes, 0.1% | 1 | View Result |
| polysaccharide binding | 9 out of 7474 genes, 0.1% | 55 out of 40099 genes, 0.1% | 1 | View Result |
| macromolecule transmembrane transporter activity | 18 out of 7474 genes, 0.2% | 107 out of 40099 genes, 0.3% | 1 | View Result |
| P-P-bond-hydrolysis-driven protein transmembrane transporter activity | 15 out of 7474 genes, 0.2% | 90 out of 40099 genes, 0.2% | 1 | View Result |
| transition metal ion transmembrane transporter activity | 25 out of 7474 genes, 0.3% | 147 out of 40099 genes, 0.4% | 1 | View Result |
| phosphoethanolamine N-methyltransferase activity | 2 out of 7474 genes, 0.0% | 13 out of 40099 genes, 0.0% | 1 | View Result |
| phosphatidylinositol-3-phosphatase activity | 2 out of 7474 genes, 0.0% | 13 out of 40099 genes, 0.0% | 1 | View Result |
| 3'(2'),5'-bisphosphate nucleotidase activity | 2 out of 7474 genes, 0.0% | 13 out of 40099 genes, 0.0% | 1 | View Result |
| phosphorus-oxygen lyase activity | 2 out of 7474 genes, 0.0% | 13 out of 40099 genes, 0.0% | 1 | View Result |
| nucleotide phosphatase activity | 2 out of 7474 genes, 0.0% | 13 out of 40099 genes, 0.0% | 1 | View Result |
| dihydrokaempferol 4-reductase activity | 2 out of 7474 genes, 0.0% | 13 out of 40099 genes, 0.0% | 1 | View Result |
| phosphatidylinositol-3,5-bisphosphate 3-phosphatase activity | 2 out of 7474 genes, 0.0% | 13 out of 40099 genes, 0.0% | 1 | View Result |
| 3'-5'-exoribonuclease activity | 12 out of 7474 genes, 0.2% | 73 out of 40099 genes, 0.2% | 1 | View Result |
| protein homodimerization activity | 45 out of 7474 genes, 0.6% | 260 out of 40099 genes, 0.6% | 1 | View Result |
| transcription regulatory region sequence-specific DNA binding | 5 out of 7474 genes, 0.1% | 32 out of 40099 genes, 0.1% | 1 | View Result |
| cofactor transporter activity | 5 out of 7474 genes, 0.1% | 32 out of 40099 genes, 0.1% | 1 | View Result |
| methyl-CpG binding | 4 out of 7474 genes, 0.1% | 26 out of 40099 genes, 0.1% | 1 | View Result |
| plus-end-directed microtubule motor activity | 4 out of 7474 genes, 0.1% | 26 out of 40099 genes, 0.1% | 1 | View Result |
| microtubule minus-end binding | 4 out of 7474 genes, 0.1% | 26 out of 40099 genes, 0.1% | 1 | View Result |
| nucleotide kinase activity | 10 out of 7474 genes, 0.1% | 62 out of 40099 genes, 0.2% | 1 | View Result |
| transcription regulatory region DNA binding | 11 out of 7474 genes, 0.1% | 68 out of 40099 genes, 0.2% | 1 | View Result |
| nucleotide transmembrane transporter activity | 12 out of 7474 genes, 0.2% | 74 out of 40099 genes, 0.2% | 1 | View Result |
| GTPase binding | 12 out of 7474 genes, 0.2% | 74 out of 40099 genes, 0.2% | 1 | View Result |
| motor activity | 54 out of 7474 genes, 0.7% | 313 out of 40099 genes, 0.8% | 1 | View Result |
| protein kinase C activity | 8 out of 7474 genes, 0.1% | 51 out of 40099 genes, 0.1% | 1 | View Result |
| acylglycerol O-acyltransferase activity | 8 out of 7474 genes, 0.1% | 51 out of 40099 genes, 0.1% | 1 | View Result |
| prenyltransferase activity | 6 out of 7474 genes, 0.1% | 39 out of 40099 genes, 0.1% | 1 | View Result |
| long-chain fatty acid-CoA ligase activity | 5 out of 7474 genes, 0.1% | 33 out of 40099 genes, 0.1% | 1 | View Result |
| hydrolase activity, hydrolyzing N-glycosyl compounds | 5 out of 7474 genes, 0.1% | 33 out of 40099 genes, 0.1% | 1 | View Result |
| 6-phosphofructo-2-kinase activity | 2 out of 7474 genes, 0.0% | 14 out of 40099 genes, 0.0% | 1 | View Result |
| copper-exporting ATPase activity | 2 out of 7474 genes, 0.0% | 14 out of 40099 genes, 0.0% | 1 | View Result |
| adenosylmethionine decarboxylase activity | 2 out of 7474 genes, 0.0% | 14 out of 40099 genes, 0.0% | 1 | View Result |
| galactokinase activity | 2 out of 7474 genes, 0.0% | 14 out of 40099 genes, 0.0% | 1 | View Result |
| glycine dehydrogenase (decarboxylating) activity | 2 out of 7474 genes, 0.0% | 14 out of 40099 genes, 0.0% | 1 | View Result |
| dihydrolipoyllysine-residue acetyltransferase activity | 2 out of 7474 genes, 0.0% | 14 out of 40099 genes, 0.0% | 1 | View Result |
| folic acid transporter activity | 2 out of 7474 genes, 0.0% | 14 out of 40099 genes, 0.0% | 1 | View Result |
| UDP-4-keto-6-deoxy-glucose-3,5-epimerase activity | 2 out of 7474 genes, 0.0% | 14 out of 40099 genes, 0.0% | 1 | View Result |
| UDP-4-keto-rhamnose-4-keto-reductase activity | 2 out of 7474 genes, 0.0% | 14 out of 40099 genes, 0.0% | 1 | View Result |
| phosphoglycerate transmembrane transporter activity | 2 out of 7474 genes, 0.0% | 14 out of 40099 genes, 0.0% | 1 | View Result |
| S-acetyltransferase activity | 2 out of 7474 genes, 0.0% | 14 out of 40099 genes, 0.0% | 1 | View Result |
| oxidoreductase activity, acting on the CH-NH2 group of donors, disulfide as acceptor | 2 out of 7474 genes, 0.0% | 14 out of 40099 genes, 0.0% | 1 | View Result |
| oxidoreductase activity, acting on NAD(P)H, nitrogenous group as acceptor | 2 out of 7474 genes, 0.0% | 14 out of 40099 genes, 0.0% | 1 | View Result |
| lipoate-protein ligase activity | 2 out of 7474 genes, 0.0% | 14 out of 40099 genes, 0.0% | 1 | View Result |
| lipoate synthase activity | 2 out of 7474 genes, 0.0% | 14 out of 40099 genes, 0.0% | 1 | View Result |
| SUMO ligase activity | 2 out of 7474 genes, 0.0% | 14 out of 40099 genes, 0.0% | 1 | View Result |
| 3'-5' DNA helicase activity | 2 out of 7474 genes, 0.0% | 14 out of 40099 genes, 0.0% | 1 | View Result |
| phosphatidylinositol monophosphate phosphatase activity | 2 out of 7474 genes, 0.0% | 14 out of 40099 genes, 0.0% | 1 | View Result |
| 11-beta-hydroxysteroid dehydrogenase (NADP+) activity | 2 out of 7474 genes, 0.0% | 14 out of 40099 genes, 0.0% | 1 | View Result |
| poly-purine tract binding | 2 out of 7474 genes, 0.0% | 14 out of 40099 genes, 0.0% | 1 | View Result |
| phosphatidylinositol-3,5-bisphosphate binding | 2 out of 7474 genes, 0.0% | 14 out of 40099 genes, 0.0% | 1 | View Result |
| protein binding transcription factor activity | 23 out of 7474 genes, 0.3% | 139 out of 40099 genes, 0.3% | 1 | View Result |
| polynucleotide adenylyltransferase activity | 4 out of 7474 genes, 0.1% | 27 out of 40099 genes, 0.1% | 1 | View Result |
| AMP binding | 4 out of 7474 genes, 0.1% | 27 out of 40099 genes, 0.1% | 1 | View Result |
| 1-phosphatidylinositol-4-phosphate 5-kinase activity | 4 out of 7474 genes, 0.1% | 27 out of 40099 genes, 0.1% | 1 | View Result |
| sulfurtransferase activity | 4 out of 7474 genes, 0.1% | 27 out of 40099 genes, 0.1% | 1 | View Result |
| fructose-2,6-bisphosphate 2-phosphatase activity | 3 out of 7474 genes, 0.0% | 21 out of 40099 genes, 0.1% | 1 | View Result |
| monosaccharide transmembrane transporter activity | 3 out of 7474 genes, 0.0% | 21 out of 40099 genes, 0.1% | 1 | View Result |
| lipoxygenase activity | 7 out of 7474 genes, 0.1% | 46 out of 40099 genes, 0.1% | 1 | View Result |
| sugar-phosphatase activity | 6 out of 7474 genes, 0.1% | 40 out of 40099 genes, 0.1% | 1 | View Result |
| protein transmembrane transporter activity | 15 out of 7474 genes, 0.2% | 94 out of 40099 genes, 0.2% | 1 | View Result |
| methionine adenosyltransferase activity | 5 out of 7474 genes, 0.1% | 34 out of 40099 genes, 0.1% | 1 | View Result |
| macrolide binding | 5 out of 7474 genes, 0.1% | 34 out of 40099 genes, 0.1% | 1 | View Result |
| FK506 binding | 5 out of 7474 genes, 0.1% | 34 out of 40099 genes, 0.1% | 1 | View Result |
| glucose-1-phosphate adenylyltransferase activity | 5 out of 7474 genes, 0.1% | 34 out of 40099 genes, 0.1% | 1 | View Result |
| MAP kinase kinase kinase activity | 38 out of 7474 genes, 0.5% | 227 out of 40099 genes, 0.6% | 1 | View Result |
| cytidylyltransferase activity | 4 out of 7474 genes, 0.1% | 28 out of 40099 genes, 0.1% | 1 | View Result |
| carboxy-lyase activity | 32 out of 7474 genes, 0.4% | 193 out of 40099 genes, 0.5% | 1 | View Result |
| regulatory region DNA binding | 11 out of 7474 genes, 0.1% | 71 out of 40099 genes, 0.2% | 1 | View Result |
| regulatory region nucleic acid binding | 11 out of 7474 genes, 0.1% | 71 out of 40099 genes, 0.2% | 1 | View Result |
| ubiquitin-specific protease activity | 9 out of 7474 genes, 0.1% | 59 out of 40099 genes, 0.1% | 1 | View Result |
| trehalose-phosphatase activity | 8 out of 7474 genes, 0.1% | 53 out of 40099 genes, 0.1% | 1 | View Result |
| purine nucleoside transmembrane transporter activity | 7 out of 7474 genes, 0.1% | 47 out of 40099 genes, 0.1% | 1 | View Result |
| intracellular ligand-gated ion channel activity | 2 out of 7474 genes, 0.0% | 15 out of 40099 genes, 0.0% | 1 | View Result |
| 1-aminocyclopropane-1-carboxylate oxidase activity | 2 out of 7474 genes, 0.0% | 15 out of 40099 genes, 0.0% | 1 | View Result |
| dihydrolipoamide S-acyltransferase activity | 2 out of 7474 genes, 0.0% | 15 out of 40099 genes, 0.0% | 1 | View Result |
| carbohydrate phosphatase activity | 15 out of 7474 genes, 0.2% | 95 out of 40099 genes, 0.2% | 1 | View Result |
| signal sequence binding | 6 out of 7474 genes, 0.1% | 41 out of 40099 genes, 0.1% | 1 | View Result |
| ribonuclease activity | 36 out of 7474 genes, 0.5% | 217 out of 40099 genes, 0.5% | 1 | View Result |
| 1-phosphatidylinositol-3-phosphate 5-kinase activity | 3 out of 7474 genes, 0.0% | 22 out of 40099 genes, 0.1% | 1 | View Result |
| phosphorylase activity | 5 out of 7474 genes, 0.1% | 35 out of 40099 genes, 0.1% | 1 | View Result |
| electron carrier activity | 117 out of 7474 genes, 1.6% | 672 out of 40099 genes, 1.7% | 1 | View Result |
| UDP-glycosyltransferase activity | 141 out of 7474 genes, 1.9% | 806 out of 40099 genes, 2.0% | 1 | View Result |
| cyclin-dependent protein serine/threonine kinase activity | 8 out of 7474 genes, 0.1% | 54 out of 40099 genes, 0.1% | 1 | View Result |
| cyclin-dependent protein kinase activity | 8 out of 7474 genes, 0.1% | 54 out of 40099 genes, 0.1% | 1 | View Result |
| 3-hydroxyisobutyryl-CoA hydrolase activity | 4 out of 7474 genes, 0.1% | 29 out of 40099 genes, 0.1% | 1 | View Result |
| glyceraldehyde-3-phosphate dehydrogenase (NAD+) (phosphorylating) activity | 4 out of 7474 genes, 0.1% | 29 out of 40099 genes, 0.1% | 1 | View Result |
| alpha-amylase activity | 4 out of 7474 genes, 0.1% | 29 out of 40099 genes, 0.1% | 1 | View Result |
| double-stranded methylated DNA binding | 4 out of 7474 genes, 0.1% | 29 out of 40099 genes, 0.1% | 1 | View Result |
| drug binding | 6 out of 7474 genes, 0.1% | 42 out of 40099 genes, 0.1% | 1 | View Result |
| potassium ion transmembrane transporter activity | 29 out of 7474 genes, 0.4% | 179 out of 40099 genes, 0.4% | 1 | View Result |
| metalloexopeptidase activity | 5 out of 7474 genes, 0.1% | 36 out of 40099 genes, 0.1% | 1 | View Result |
| calcium-dependent protein kinase C activity | 2 out of 7474 genes, 0.0% | 16 out of 40099 genes, 0.0% | 1 | View Result |
| succinate-semialdehyde dehydrogenase [NAD(P)+] activity | 2 out of 7474 genes, 0.0% | 16 out of 40099 genes, 0.0% | 1 | View Result |
| radical SAM enzyme activity | 2 out of 7474 genes, 0.0% | 16 out of 40099 genes, 0.0% | 1 | View Result |
| modified amino acid binding | 2 out of 7474 genes, 0.0% | 16 out of 40099 genes, 0.0% | 1 | View Result |
| transporter activity | 638 out of 7474 genes, 8.5% | 3533 out of 40099 genes, 8.8% | 1 | View Result |
| oxidoreductase activity, acting on a sulfur group of donors, disulfide as acceptor | 8 out of 7474 genes, 0.1% | 55 out of 40099 genes, 0.1% | 1 | View Result |
| GTP-dependent protein binding | 3 out of 7474 genes, 0.0% | 23 out of 40099 genes, 0.1% | 1 | View Result |
| demethylase activity | 3 out of 7474 genes, 0.0% | 23 out of 40099 genes, 0.1% | 1 | View Result |
| 5'-3' DNA helicase activity | 3 out of 7474 genes, 0.0% | 23 out of 40099 genes, 0.1% | 1 | View Result |
| ATP-dependent 5'-3' DNA helicase activity | 3 out of 7474 genes, 0.0% | 23 out of 40099 genes, 0.1% | 1 | View Result |
| beta-amylase activity | 4 out of 7474 genes, 0.1% | 30 out of 40099 genes, 0.1% | 1 | View Result |
| single-stranded DNA binding | 6 out of 7474 genes, 0.1% | 43 out of 40099 genes, 0.1% | 1 | View Result |
| oxidoreductase activity, acting on the aldehyde or oxo group of donors | 64 out of 7474 genes, 0.9% | 381 out of 40099 genes, 1.0% | 1 | View Result |
| alcohol binding | 9 out of 7474 genes, 0.1% | 62 out of 40099 genes, 0.2% | 1 | View Result |
| ATP-dependent helicase activity | 72 out of 7474 genes, 1.0% | 427 out of 40099 genes, 1.1% | 1 | View Result |
| purine NTP-dependent helicase activity | 72 out of 7474 genes, 1.0% | 427 out of 40099 genes, 1.1% | 1 | View Result |
| exoribonuclease activity | 13 out of 7474 genes, 0.2% | 87 out of 40099 genes, 0.2% | 1 | View Result |
| exonuclease activity, active with either ribo- or deoxyribonucleic acids and producing 5'-phosphomonoesters | 13 out of 7474 genes, 0.2% | 87 out of 40099 genes, 0.2% | 1 | View Result |
| exoribonuclease activity, producing 5'-phosphomonoesters | 13 out of 7474 genes, 0.2% | 87 out of 40099 genes, 0.2% | 1 | View Result |
| ATPase activity | 333 out of 7474 genes, 4.5% | 1876 out of 40099 genes, 4.7% | 1 | View Result |
| methyl-CpNpG binding | 3 out of 7474 genes, 0.0% | 24 out of 40099 genes, 0.1% | 1 | View Result |
| methyl-CpNpN binding | 3 out of 7474 genes, 0.0% | 24 out of 40099 genes, 0.1% | 1 | View Result |
| S-acyltransferase activity | 3 out of 7474 genes, 0.0% | 24 out of 40099 genes, 0.1% | 1 | View Result |
| RNA polymerase II transcription factor binding transcription factor activity | 2 out of 7474 genes, 0.0% | 17 out of 40099 genes, 0.0% | 1 | View Result |
| RNA polymerase II transcription cofactor activity | 2 out of 7474 genes, 0.0% | 17 out of 40099 genes, 0.0% | 1 | View Result |
| peptide receptor activity | 2 out of 7474 genes, 0.0% | 17 out of 40099 genes, 0.0% | 1 | View Result |
| ionotropic glutamate receptor activity | 2 out of 7474 genes, 0.0% | 17 out of 40099 genes, 0.0% | 1 | View Result |
| excitatory extracellular ligand-gated ion channel activity | 2 out of 7474 genes, 0.0% | 17 out of 40099 genes, 0.0% | 1 | View Result |
| extracellular-glutamate-gated ion channel activity | 2 out of 7474 genes, 0.0% | 17 out of 40099 genes, 0.0% | 1 | View Result |
| calcium-dependent phospholipid binding | 2 out of 7474 genes, 0.0% | 17 out of 40099 genes, 0.0% | 1 | View Result |
| phosphatidylinositol-4,5-bisphosphate binding | 2 out of 7474 genes, 0.0% | 17 out of 40099 genes, 0.0% | 1 | View Result |
| cyclohydrolase activity | 2 out of 7474 genes, 0.0% | 17 out of 40099 genes, 0.0% | 1 | View Result |
| phosphatidylinositol bisphosphate phosphatase activity | 2 out of 7474 genes, 0.0% | 17 out of 40099 genes, 0.0% | 1 | View Result |
| histone methyltransferase activity (H3-K9 specific) | 2 out of 7474 genes, 0.0% | 17 out of 40099 genes, 0.0% | 1 | View Result |
| catalytic activity | 4841 out of 7474 genes, 64.8% | 26181 out of 40099 genes, 65.3% | 1 | View Result |
| hexokinase activity | 4 out of 7474 genes, 0.1% | 31 out of 40099 genes, 0.1% | 1 | View Result |
| phospholipase C activity | 4 out of 7474 genes, 0.1% | 31 out of 40099 genes, 0.1% | 1 | View Result |
| phosphoenolpyruvate carboxykinase activity | 8 out of 7474 genes, 0.1% | 57 out of 40099 genes, 0.1% | 1 | View Result |
| inorganic anion transmembrane transporter activity | 40 out of 7474 genes, 0.5% | 247 out of 40099 genes, 0.6% | 1 | View Result |
| pseudouridine synthase activity | 5 out of 7474 genes, 0.1% | 38 out of 40099 genes, 0.1% | 1 | View Result |
| deaminase activity | 5 out of 7474 genes, 0.1% | 38 out of 40099 genes, 0.1% | 1 | View Result |
| phosphoenolpyruvate carboxylase activity | 7 out of 7474 genes, 0.1% | 51 out of 40099 genes, 0.1% | 1 | View Result |
| transcription factor binding transcription factor activity | 18 out of 7474 genes, 0.2% | 119 out of 40099 genes, 0.3% | 1 | View Result |
| transcription cofactor activity | 18 out of 7474 genes, 0.2% | 119 out of 40099 genes, 0.3% | 1 | View Result |
| amide binding | 9 out of 7474 genes, 0.1% | 64 out of 40099 genes, 0.2% | 1 | View Result |
| double-stranded DNA binding | 15 out of 7474 genes, 0.2% | 101 out of 40099 genes, 0.3% | 1 | View Result |
| polygalacturonate 4-alpha-galacturonosyltransferase activity | 15 out of 7474 genes, 0.2% | 101 out of 40099 genes, 0.3% | 1 | View Result |
| protein tyrosine kinase activity | 111 out of 7474 genes, 1.5% | 652 out of 40099 genes, 1.6% | 1 | View Result |
| o-succinylbenzoate-CoA ligase activity | 3 out of 7474 genes, 0.0% | 25 out of 40099 genes, 0.1% | 1 | View Result |
| DNA-directed DNA polymerase activity | 11 out of 7474 genes, 0.1% | 77 out of 40099 genes, 0.2% | 1 | View Result |
| phosphatidate cytidylyltransferase activity | 2 out of 7474 genes, 0.0% | 18 out of 40099 genes, 0.0% | 1 | View Result |
| calcium channel activity | 2 out of 7474 genes, 0.0% | 18 out of 40099 genes, 0.0% | 1 | View Result |
| glutamate receptor activity | 2 out of 7474 genes, 0.0% | 18 out of 40099 genes, 0.0% | 1 | View Result |
| oxysterol binding | 2 out of 7474 genes, 0.0% | 18 out of 40099 genes, 0.0% | 1 | View Result |
| glycerophosphodiester phosphodiesterase activity | 2 out of 7474 genes, 0.0% | 18 out of 40099 genes, 0.0% | 1 | View Result |
| mannose-phosphate guanylyltransferase activity | 2 out of 7474 genes, 0.0% | 18 out of 40099 genes, 0.0% | 1 | View Result |
| myosin binding | 2 out of 7474 genes, 0.0% | 18 out of 40099 genes, 0.0% | 1 | View Result |
| myosin tail binding | 2 out of 7474 genes, 0.0% | 18 out of 40099 genes, 0.0% | 1 | View Result |
| myosin heavy chain binding | 2 out of 7474 genes, 0.0% | 18 out of 40099 genes, 0.0% | 1 | View Result |
| copper-transporting ATPase activity | 2 out of 7474 genes, 0.0% | 18 out of 40099 genes, 0.0% | 1 | View Result |
| myosin XI tail binding | 2 out of 7474 genes, 0.0% | 18 out of 40099 genes, 0.0% | 1 | View Result |
| threonine-type endopeptidase activity | 10 out of 7474 genes, 0.1% | 71 out of 40099 genes, 0.2% | 1 | View Result |
| threonine-type peptidase activity | 10 out of 7474 genes, 0.1% | 71 out of 40099 genes, 0.2% | 1 | View Result |
| carbohydrate kinase activity | 18 out of 7474 genes, 0.2% | 120 out of 40099 genes, 0.3% | 1 | View Result |
| intramolecular transferase activity, phosphotransferases | 7 out of 7474 genes, 0.1% | 52 out of 40099 genes, 0.1% | 1 | View Result |
| peptide binding | 7 out of 7474 genes, 0.1% | 52 out of 40099 genes, 0.1% | 1 | View Result |
| protein histidine kinase binding | 7 out of 7474 genes, 0.1% | 52 out of 40099 genes, 0.1% | 1 | View Result |
| tRNA binding | 5 out of 7474 genes, 0.1% | 39 out of 40099 genes, 0.1% | 1 | View Result |
| chromatin binding | 52 out of 7474 genes, 0.7% | 320 out of 40099 genes, 0.8% | 1 | View Result |
| amylase activity | 8 out of 7474 genes, 0.1% | 59 out of 40099 genes, 0.1% | 1 | View Result |
| substrate-specific channel activity | 40 out of 7474 genes, 0.5% | 251 out of 40099 genes, 0.6% | 1 | View Result |
| hydrolase activity, acting on carbon-nitrogen (but not peptide) bonds, in linear amides | 18 out of 7474 genes, 0.2% | 121 out of 40099 genes, 0.3% | 1 | View Result |
| translation release factor activity | 7 out of 7474 genes, 0.1% | 53 out of 40099 genes, 0.1% | 1 | View Result |
| translation termination factor activity | 7 out of 7474 genes, 0.1% | 53 out of 40099 genes, 0.1% | 1 | View Result |
| adenosylhomocysteinase activity | 3 out of 7474 genes, 0.0% | 26 out of 40099 genes, 0.1% | 1 | View Result |
| fructokinase activity | 3 out of 7474 genes, 0.0% | 26 out of 40099 genes, 0.1% | 1 | View Result |
| trialkylsulfonium hydrolase activity | 3 out of 7474 genes, 0.0% | 26 out of 40099 genes, 0.1% | 1 | View Result |
| peptide transporter activity | 12 out of 7474 genes, 0.2% | 85 out of 40099 genes, 0.2% | 1 | View Result |
| phospholipase activity | 27 out of 7474 genes, 0.4% | 176 out of 40099 genes, 0.4% | 1 | View Result |
| guanyl-nucleotide exchange factor activity | 11 out of 7474 genes, 0.1% | 79 out of 40099 genes, 0.2% | 1 | View Result |
| glucokinase activity | 2 out of 7474 genes, 0.0% | 19 out of 40099 genes, 0.0% | 1 | View Result |
| calcium-dependent protein serine/threonine kinase activity | 2 out of 7474 genes, 0.0% | 19 out of 40099 genes, 0.0% | 1 | View Result |
| chlorophyllide a oxygenase [overall] activity | 2 out of 7474 genes, 0.0% | 19 out of 40099 genes, 0.0% | 1 | View Result |
| calcium-dependent protein kinase activity | 2 out of 7474 genes, 0.0% | 19 out of 40099 genes, 0.0% | 1 | View Result |
| oxidoreductase activity, acting on NAD(P)H, heme protein as acceptor | 2 out of 7474 genes, 0.0% | 19 out of 40099 genes, 0.0% | 1 | View Result |
| hydrolase activity, acting on carbon-nitrogen (but not peptide) bonds, in cyclic amides | 2 out of 7474 genes, 0.0% | 19 out of 40099 genes, 0.0% | 1 | View Result |
| channel activity | 40 out of 7474 genes, 0.5% | 253 out of 40099 genes, 0.6% | 1 | View Result |
| passive transmembrane transporter activity | 40 out of 7474 genes, 0.5% | 253 out of 40099 genes, 0.6% | 1 | View Result |
| transferase activity, transferring pentosyl groups | 22 out of 7474 genes, 0.3% | 147 out of 40099 genes, 0.4% | 1 | View Result |
| RNA polymerase activity | 22 out of 7474 genes, 0.3% | 147 out of 40099 genes, 0.4% | 1 | View Result |
| enzyme regulator activity | 90 out of 7474 genes, 1.2% | 541 out of 40099 genes, 1.3% | 1 | View Result |
| phosphatidylinositol kinase activity | 4 out of 7474 genes, 0.1% | 34 out of 40099 genes, 0.1% | 1 | View Result |
| alpha,alpha-trehalose-phosphate synthase (UDP-forming) activity | 5 out of 7474 genes, 0.1% | 41 out of 40099 genes, 0.1% | 1 | View Result |
| alpha-glucosidase activity | 3 out of 7474 genes, 0.0% | 27 out of 40099 genes, 0.1% | 1 | View Result |
| methylated histone residue binding | 3 out of 7474 genes, 0.0% | 27 out of 40099 genes, 0.1% | 1 | View Result |
| ubiquitin thiolesterase activity | 13 out of 7474 genes, 0.2% | 93 out of 40099 genes, 0.2% | 1 | View Result |
| mRNA binding | 19 out of 7474 genes, 0.3% | 130 out of 40099 genes, 0.3% | 1 | View Result |
| clathrin binding | 12 out of 7474 genes, 0.2% | 87 out of 40099 genes, 0.2% | 1 | View Result |
| sulfate transmembrane transporter activity | 9 out of 7474 genes, 0.1% | 68 out of 40099 genes, 0.2% | 1 | View Result |
| calcium ion binding | 107 out of 7474 genes, 1.4% | 641 out of 40099 genes, 1.6% | 1 | View Result |
| gated channel activity | 28 out of 7474 genes, 0.4% | 185 out of 40099 genes, 0.5% | 1 | View Result |
| ion gated channel activity | 28 out of 7474 genes, 0.4% | 185 out of 40099 genes, 0.5% | 1 | View Result |
| NAD+ kinase activity | 2 out of 7474 genes, 0.0% | 20 out of 40099 genes, 0.0% | 1 | View Result |
| transferase activity, transferring amino-acyl groups | 2 out of 7474 genes, 0.0% | 20 out of 40099 genes, 0.0% | 1 | View Result |
| CDP-alcohol phosphatidyltransferase activity | 2 out of 7474 genes, 0.0% | 20 out of 40099 genes, 0.0% | 1 | View Result |
| Hsp70 protein binding | 2 out of 7474 genes, 0.0% | 20 out of 40099 genes, 0.0% | 1 | View Result |
| starch binding | 2 out of 7474 genes, 0.0% | 20 out of 40099 genes, 0.0% | 1 | View Result |
| monovalent inorganic cation transmembrane transporter activity | 111 out of 7474 genes, 1.5% | 664 out of 40099 genes, 1.7% | 1 | View Result |
| non-membrane spanning protein tyrosine kinase activity | 34 out of 7474 genes, 0.5% | 221 out of 40099 genes, 0.6% | 1 | View Result |
| SNAP receptor activity | 10 out of 7474 genes, 0.1% | 75 out of 40099 genes, 0.2% | 1 | View Result |
| voltage-gated chloride channel activity | 4 out of 7474 genes, 0.1% | 35 out of 40099 genes, 0.1% | 1 | View Result |
| nucleotidase activity | 4 out of 7474 genes, 0.1% | 35 out of 40099 genes, 0.1% | 1 | View Result |
| potassium:hydrogen antiporter activity | 4 out of 7474 genes, 0.1% | 35 out of 40099 genes, 0.1% | 1 | View Result |
| transferase activity, transferring aldehyde or ketonic groups | 4 out of 7474 genes, 0.1% | 35 out of 40099 genes, 0.1% | 1 | View Result |
| potassium ion antiporter activity | 4 out of 7474 genes, 0.1% | 35 out of 40099 genes, 0.1% | 1 | View Result |
| extracellular ligand-gated ion channel activity | 5 out of 7474 genes, 0.1% | 42 out of 40099 genes, 0.1% | 1 | View Result |
| FMN binding | 16 out of 7474 genes, 0.2% | 113 out of 40099 genes, 0.3% | 1 | View Result |
| voltage-gated anion channel activity | 6 out of 7474 genes, 0.1% | 49 out of 40099 genes, 0.1% | 1 | View Result |
| microtubule-severing ATPase activity | 21 out of 7474 genes, 0.3% | 144 out of 40099 genes, 0.4% | 1 | View Result |
| 3-chloroallyl aldehyde dehydrogenase activity | 8 out of 7474 genes, 0.1% | 63 out of 40099 genes, 0.2% | 1 | View Result |
| beta-1,4-mannosylglycoprotein 4-beta-N-acetylglucosaminyltransferase activity | 2 out of 7474 genes, 0.0% | 21 out of 40099 genes, 0.1% | 1 | View Result |
| G-protein coupled receptor activity | 2 out of 7474 genes, 0.0% | 21 out of 40099 genes, 0.1% | 1 | View Result |
| adenine nucleotide transmembrane transporter activity | 4 out of 7474 genes, 0.1% | 36 out of 40099 genes, 0.1% | 1 | View Result |
| chloride channel activity | 4 out of 7474 genes, 0.1% | 36 out of 40099 genes, 0.1% | 1 | View Result |
| purine ribonucleotide transmembrane transporter activity | 4 out of 7474 genes, 0.1% | 36 out of 40099 genes, 0.1% | 1 | View Result |
| purine nucleotide transmembrane transporter activity | 4 out of 7474 genes, 0.1% | 36 out of 40099 genes, 0.1% | 1 | View Result |
| sulfur compound transmembrane transporter activity | 9 out of 7474 genes, 0.1% | 70 out of 40099 genes, 0.2% | 1 | View Result |
| ATP-dependent DNA helicase activity | 7 out of 7474 genes, 0.1% | 57 out of 40099 genes, 0.1% | 1 | View Result |
| malic enzyme activity | 7 out of 7474 genes, 0.1% | 57 out of 40099 genes, 0.1% | 1 | View Result |
| ATPase activity, coupled to transmembrane movement of substances | 147 out of 7474 genes, 2.0% | 874 out of 40099 genes, 2.2% | 1 | View Result |
| ATPase activity, coupled to movement of substances | 147 out of 7474 genes, 2.0% | 875 out of 40099 genes, 2.2% | 1 | View Result |
| acyl-CoA dehydrogenase activity | 8 out of 7474 genes, 0.1% | 64 out of 40099 genes, 0.2% | 1 | View Result |
| carbon-oxygen lyase activity, acting on phosphates | 11 out of 7474 genes, 0.1% | 84 out of 40099 genes, 0.2% | 1 | View Result |
| small conjugating protein-specific protease activity | 9 out of 7474 genes, 0.1% | 71 out of 40099 genes, 0.2% | 1 | View Result |
| glycine hydroxymethyltransferase activity | 5 out of 7474 genes, 0.1% | 44 out of 40099 genes, 0.1% | 1 | View Result |
| anion channel activity | 6 out of 7474 genes, 0.1% | 51 out of 40099 genes, 0.1% | 1 | View Result |
| protein kinase binding | 16 out of 7474 genes, 0.2% | 116 out of 40099 genes, 0.3% | 1 | View Result |
| DNA ligase (ATP) activity | 4 out of 7474 genes, 0.1% | 37 out of 40099 genes, 0.1% | 1 | View Result |
| chloride transmembrane transporter activity | 4 out of 7474 genes, 0.1% | 37 out of 40099 genes, 0.1% | 1 | View Result |
| O-acetyltransferase activity | 4 out of 7474 genes, 0.1% | 37 out of 40099 genes, 0.1% | 1 | View Result |
| hydrolase activity, acting on acid anhydrides, catalyzing transmembrane movement of substances | 149 out of 7474 genes, 2.0% | 889 out of 40099 genes, 2.2% | 1 | View Result |
| inositol hexakisphosphate binding | 2 out of 7474 genes, 0.0% | 22 out of 40099 genes, 0.1% | 1 | View Result |
| phosphogluconate dehydrogenase (decarboxylating) activity | 2 out of 7474 genes, 0.0% | 22 out of 40099 genes, 0.1% | 1 | View Result |
| 5'-nucleotidase activity | 2 out of 7474 genes, 0.0% | 22 out of 40099 genes, 0.1% | 1 | View Result |
| sterol binding | 2 out of 7474 genes, 0.0% | 22 out of 40099 genes, 0.1% | 1 | View Result |
| DNA binding | 439 out of 7474 genes, 5.9% | 2506 out of 40099 genes, 6.2% | 1 | View Result |
| nucleotide diphosphatase activity | 3 out of 7474 genes, 0.0% | 30 out of 40099 genes, 0.1% | 1 | View Result |
| 7S RNA binding | 3 out of 7474 genes, 0.0% | 30 out of 40099 genes, 0.1% | 1 | View Result |
| ion channel activity | 31 out of 7474 genes, 0.4% | 209 out of 40099 genes, 0.5% | 1 | View Result |
| transmembrane signaling receptor activity | 30 out of 7474 genes, 0.4% | 203 out of 40099 genes, 0.5% | 1 | View Result |
| transmembrane receptor protein kinase activity | 16 out of 7474 genes, 0.2% | 117 out of 40099 genes, 0.3% | 1 | View Result |
| sequence-specific DNA binding | 76 out of 7474 genes, 1.0% | 474 out of 40099 genes, 1.2% | 1 | View Result |
| DNA-directed RNA polymerase activity | 19 out of 7474 genes, 0.3% | 136 out of 40099 genes, 0.3% | 1 | View Result |
| iron ion transmembrane transporter activity | 7 out of 7474 genes, 0.1% | 59 out of 40099 genes, 0.1% | 1 | View Result |
| lipase activity | 41 out of 7474 genes, 0.5% | 270 out of 40099 genes, 0.7% | 1 | View Result |
| DNA ligase activity | 4 out of 7474 genes, 0.1% | 38 out of 40099 genes, 0.1% | 1 | View Result |
| inositol trisphosphate phosphatase activity | 4 out of 7474 genes, 0.1% | 38 out of 40099 genes, 0.1% | 1 | View Result |
| aspartate kinase activity | 2 out of 7474 genes, 0.0% | 23 out of 40099 genes, 0.1% | 1 | View Result |
| kinase activator activity | 2 out of 7474 genes, 0.0% | 23 out of 40099 genes, 0.1% | 1 | View Result |
| phosphatidylinositol phosphate phosphatase activity | 2 out of 7474 genes, 0.0% | 23 out of 40099 genes, 0.1% | 1 | View Result |
| receptor activity | 47 out of 7474 genes, 0.6% | 307 out of 40099 genes, 0.8% | 1 | View Result |
| hydrogen ion transmembrane transporter activity | 77 out of 7474 genes, 1.0% | 483 out of 40099 genes, 1.2% | 1 | View Result |
| inositol phosphate phosphatase activity | 6 out of 7474 genes, 0.1% | 53 out of 40099 genes, 0.1% | 1 | View Result |
| oxidoreductase activity, acting on the aldehyde or oxo group of donors, NAD or NADP as acceptor | 41 out of 7474 genes, 0.5% | 273 out of 40099 genes, 0.7% | 1 | View Result |
| helicase activity | 113 out of 7474 genes, 1.5% | 693 out of 40099 genes, 1.7% | 1 | View Result |
| nucleoside kinase activity | 6 out of 7474 genes, 0.1% | 54 out of 40099 genes, 0.1% | 1 | View Result |
| anion:anion antiporter activity | 3 out of 7474 genes, 0.0% | 32 out of 40099 genes, 0.1% | 1 | View Result |
| oxidoreductase activity, acting on the CH-OH group of donors, oxygen as acceptor | 8 out of 7474 genes, 0.1% | 68 out of 40099 genes, 0.2% | 1 | View Result |
| metal ion transmembrane transporter activity | 94 out of 7474 genes, 1.3% | 585 out of 40099 genes, 1.5% | 1 | View Result |
| hydrolase activity | 1620 out of 7474 genes, 21.7% | 8982 out of 40099 genes, 22.4% | 1 | View Result |
| dolichyl-diphosphooligosaccharide-protein glycotransferase activity | 2 out of 7474 genes, 0.0% | 24 out of 40099 genes, 0.1% | 1 | View Result |
| caffeoyl-CoA O-methyltransferase activity | 2 out of 7474 genes, 0.0% | 24 out of 40099 genes, 0.1% | 1 | View Result |
| transferase activity, transferring acyl groups | 122 out of 7474 genes, 1.6% | 747 out of 40099 genes, 1.9% | 1 | View Result |
| very-long-chain-(S)-2-hydroxy-acid oxidase activity | 4 out of 7474 genes, 0.1% | 40 out of 40099 genes, 0.1% | 1 | View Result |
| long-chain-(S)-2-hydroxy-long-chain-acid oxidase activity | 4 out of 7474 genes, 0.1% | 40 out of 40099 genes, 0.1% | 1 | View Result |
| medium-chain-(S)-2-hydroxy-acid oxidase activity | 4 out of 7474 genes, 0.1% | 40 out of 40099 genes, 0.1% | 1 | View Result |
| adenylyltransferase activity | 13 out of 7474 genes, 0.2% | 102 out of 40099 genes, 0.3% | 1 | View Result |
| kinase binding | 17 out of 7474 genes, 0.2% | 128 out of 40099 genes, 0.3% | 1 | View Result |
| nucleic acid binding | 1075 out of 7474 genes, 14.4% | 6022 out of 40099 genes, 15.0% | 1 | View Result |
| glycolate oxidase activity | 5 out of 7474 genes, 0.1% | 48 out of 40099 genes, 0.1% | 1 | View Result |
| oxidoreductase activity, acting on single donors with incorporation of molecular oxygen, incorporation of one atom of oxygen (internal monooxygenases or internal mixed function oxidases) | 3 out of 7474 genes, 0.0% | 33 out of 40099 genes, 0.1% | 1 | View Result |
| 1-acylglycerol-3-phosphate O-acyltransferase activity | 2 out of 7474 genes, 0.0% | 25 out of 40099 genes, 0.1% | 1 | View Result |
| UTP:glucose-1-phosphate uridylyltransferase activity | 2 out of 7474 genes, 0.0% | 25 out of 40099 genes, 0.1% | 1 | View Result |
| acetyl-CoA carboxylase activity | 2 out of 7474 genes, 0.0% | 25 out of 40099 genes, 0.1% | 1 | View Result |
| diacylglycerol kinase activity | 2 out of 7474 genes, 0.0% | 25 out of 40099 genes, 0.1% | 1 | View Result |
| ligase activity, forming phosphoric ester bonds | 4 out of 7474 genes, 0.1% | 41 out of 40099 genes, 0.1% | 1 | View Result |
| cis-zeatin O-beta-D-glucosyltransferase activity | 4 out of 7474 genes, 0.1% | 41 out of 40099 genes, 0.1% | 1 | View Result |
| ATPase activity, coupled | 263 out of 7474 genes, 3.5% | 1550 out of 40099 genes, 3.9% | 1 | View Result |
| RNA polymerase II carboxy-terminal domain kinase activity | 7 out of 7474 genes, 0.1% | 63 out of 40099 genes, 0.2% | 1 | View Result |
| O-methyltransferase activity | 8 out of 7474 genes, 0.1% | 70 out of 40099 genes, 0.2% | 1 | View Result |
| neutral amino acid transmembrane transporter activity | 8 out of 7474 genes, 0.1% | 70 out of 40099 genes, 0.2% | 1 | View Result |
| enzyme binding | 49 out of 7474 genes, 0.7% | 326 out of 40099 genes, 0.8% | 1 | View Result |
| carbon-nitrogen lyase activity | 5 out of 7474 genes, 0.1% | 49 out of 40099 genes, 0.1% | 1 | View Result |
| phosphatidylinositol phosphate binding | 3 out of 7474 genes, 0.0% | 34 out of 40099 genes, 0.1% | 1 | View Result |
| RNA helicase activity | 7 out of 7474 genes, 0.1% | 64 out of 40099 genes, 0.2% | 1 | View Result |
| hydrogen-exporting ATPase activity, phosphorylative mechanism | 7 out of 7474 genes, 0.1% | 64 out of 40099 genes, 0.2% | 1 | View Result |
| photoreceptor activity | 2 out of 7474 genes, 0.0% | 26 out of 40099 genes, 0.1% | 1 | View Result |
| UTP-monosaccharide-1-phosphate uridylyltransferase activity | 2 out of 7474 genes, 0.0% | 26 out of 40099 genes, 0.1% | 1 | View Result |
| signaling receptor activity | 40 out of 7474 genes, 0.5% | 275 out of 40099 genes, 0.7% | 1 | View Result |
| transferase activity, transferring acyl groups other than amino-acyl groups | 87 out of 7474 genes, 1.2% | 554 out of 40099 genes, 1.4% | 1 | View Result |
| ATP transmembrane transporter activity | 3 out of 7474 genes, 0.0% | 35 out of 40099 genes, 0.1% | 1 | View Result |
| NAD(P)H oxidase activity | 3 out of 7474 genes, 0.0% | 35 out of 40099 genes, 0.1% | 1 | View Result |
| NAPE-specific phospholipase D activity | 3 out of 7474 genes, 0.0% | 35 out of 40099 genes, 0.1% | 1 | View Result |
| calmodulin binding | 58 out of 7474 genes, 0.8% | 384 out of 40099 genes, 1.0% | 1 | View Result |
| (S)-2-hydroxy-acid oxidase activity | 5 out of 7474 genes, 0.1% | 51 out of 40099 genes, 0.1% | 1 | View Result |
| carbonate dehydratase activity | 5 out of 7474 genes, 0.1% | 51 out of 40099 genes, 0.1% | 1 | View Result |
| hydrolase activity, acting on carbon-nitrogen (but not peptide) bonds, in cyclic amidines | 5 out of 7474 genes, 0.1% | 51 out of 40099 genes, 0.1% | 1 | View Result |
| phosphotransferase activity, for other substituted phosphate groups | 2 out of 7474 genes, 0.0% | 27 out of 40099 genes, 0.1% | 1 | View Result |
| MAP kinase phosphatase activity | 2 out of 7474 genes, 0.0% | 27 out of 40099 genes, 0.1% | 1 | View Result |
| uridylyltransferase activity | 2 out of 7474 genes, 0.0% | 27 out of 40099 genes, 0.1% | 1 | View Result |
| ubiquitin protein ligase binding | 8 out of 7474 genes, 0.1% | 73 out of 40099 genes, 0.2% | 1 | View Result |
| small conjugating protein ligase binding | 8 out of 7474 genes, 0.1% | 73 out of 40099 genes, 0.2% | 1 | View Result |
| heat shock protein binding | 20 out of 7474 genes, 0.3% | 153 out of 40099 genes, 0.4% | 1 | View Result |
| L-amino acid transmembrane transporter activity | 6 out of 7474 genes, 0.1% | 59 out of 40099 genes, 0.1% | 1 | View Result |
| oxidoreductase activity, acting on the aldehyde or oxo group of donors, oxygen as acceptor | 6 out of 7474 genes, 0.1% | 59 out of 40099 genes, 0.1% | 1 | View Result |
| ubiquinol-cytochrome-c reductase activity | 3 out of 7474 genes, 0.0% | 36 out of 40099 genes, 0.1% | 1 | View Result |
| oxidoreductase activity, acting on diphenols and related substances as donors, cytochrome as acceptor | 3 out of 7474 genes, 0.0% | 36 out of 40099 genes, 0.1% | 1 | View Result |
| oxidoreductase activity, acting on NAD(P)H, oxygen as acceptor | 3 out of 7474 genes, 0.0% | 36 out of 40099 genes, 0.1% | 1 | View Result |
| trans-zeatin O-beta-D-glucosyltransferase activity | 4 out of 7474 genes, 0.1% | 44 out of 40099 genes, 0.1% | 1 | View Result |
| histone deacetylase activity | 2 out of 7474 genes, 0.0% | 28 out of 40099 genes, 0.1% | 1 | View Result |
| steroid binding | 2 out of 7474 genes, 0.0% | 28 out of 40099 genes, 0.1% | 1 | View Result |
| signal transducer activity | 129 out of 7474 genes, 1.7% | 805 out of 40099 genes, 2.0% | 1 | View Result |
| molecular transducer activity | 129 out of 7474 genes, 1.7% | 805 out of 40099 genes, 2.0% | 1 | View Result |
| protein binding | 1058 out of 7474 genes, 14.2% | 5972 out of 40099 genes, 14.9% | 1 | View Result |
| divalent inorganic cation transmembrane transporter activity | 36 out of 7474 genes, 0.5% | 256 out of 40099 genes, 0.6% | 1 | View Result |
| endoribonuclease activity, producing 5'-phosphomonoesters | 8 out of 7474 genes, 0.1% | 75 out of 40099 genes, 0.2% | 1 | View Result |
| ferrochelatase activity | 5 out of 7474 genes, 0.1% | 53 out of 40099 genes, 0.1% | 1 | View Result |
| phosphotransferase activity, carboxyl group as acceptor | 5 out of 7474 genes, 0.1% | 53 out of 40099 genes, 0.1% | 1 | View Result |
| 4 iron, 4 sulfur cluster binding | 24 out of 7474 genes, 0.3% | 182 out of 40099 genes, 0.5% | 1 | View Result |
| biotin carboxylase activity | 2 out of 7474 genes, 0.0% | 29 out of 40099 genes, 0.1% | 1 | View Result |
| uridine kinase activity | 2 out of 7474 genes, 0.0% | 29 out of 40099 genes, 0.1% | 1 | View Result |
| receptor signaling protein serine/threonine kinase activity | 45 out of 7474 genes, 0.6% | 313 out of 40099 genes, 0.8% | 1 | View Result |
| receptor signaling protein activity | 45 out of 7474 genes, 0.6% | 313 out of 40099 genes, 0.8% | 1 | View Result |
| substrate-specific transmembrane transporter activity | 402 out of 7474 genes, 5.4% | 2357 out of 40099 genes, 5.9% | 1 | View Result |
| hydroxymethyl-, formyl- and related transferase activity | 5 out of 7474 genes, 0.1% | 54 out of 40099 genes, 0.1% | 1 | View Result |
| histone-lysine N-methyltransferase activity | 5 out of 7474 genes, 0.1% | 54 out of 40099 genes, 0.1% | 1 | View Result |
| endonuclease activity, active with either ribo- or deoxyribonucleic acids and producing 5'-phosphomonoesters | 9 out of 7474 genes, 0.1% | 84 out of 40099 genes, 0.2% | 1 | View Result |
| serine O-acetyltransferase activity | 2 out of 7474 genes, 0.0% | 30 out of 40099 genes, 0.1% | 1 | View Result |
| serine O-acyltransferase activity | 2 out of 7474 genes, 0.0% | 30 out of 40099 genes, 0.1% | 1 | View Result |
| ammonia-lyase activity | 2 out of 7474 genes, 0.0% | 30 out of 40099 genes, 0.1% | 1 | View Result |
| protein deacetylase activity | 2 out of 7474 genes, 0.0% | 30 out of 40099 genes, 0.1% | 1 | View Result |
| transmembrane receptor protein serine/threonine kinase binding | 2 out of 7474 genes, 0.0% | 30 out of 40099 genes, 0.1% | 1 | View Result |
| magnesium ion binding | 58 out of 7474 genes, 0.8% | 395 out of 40099 genes, 1.0% | 1 | View Result |
| receptor serine/threonine kinase binding | 5 out of 7474 genes, 0.1% | 55 out of 40099 genes, 0.1% | 1 | View Result |
| small GTPase regulator activity | 27 out of 7474 genes, 0.4% | 204 out of 40099 genes, 0.5% | 1 | View Result |
| CoA carboxylase activity | 3 out of 7474 genes, 0.0% | 39 out of 40099 genes, 0.1% | 1 | View Result |
| terpene synthase activity | 7 out of 7474 genes, 0.1% | 71 out of 40099 genes, 0.2% | 1 | View Result |
| kinase regulator activity | 7 out of 7474 genes, 0.1% | 71 out of 40099 genes, 0.2% | 1 | View Result |
| lysine N-methyltransferase activity | 5 out of 7474 genes, 0.1% | 56 out of 40099 genes, 0.1% | 1 | View Result |
| protein-lysine N-methyltransferase activity | 5 out of 7474 genes, 0.1% | 56 out of 40099 genes, 0.1% | 1 | View Result |
| nucleobase transmembrane transporter activity | 2 out of 7474 genes, 0.0% | 31 out of 40099 genes, 0.1% | 1 | View Result |
| intramolecular oxidoreductase activity, interconverting keto- and enol-groups | 2 out of 7474 genes, 0.0% | 31 out of 40099 genes, 0.1% | 1 | View Result |
| deacetylase activity | 2 out of 7474 genes, 0.0% | 31 out of 40099 genes, 0.1% | 1 | View Result |
| peptidase activity, acting on L-amino acid peptides | 202 out of 7474 genes, 2.7% | 1240 out of 40099 genes, 3.1% | 1 | View Result |
| lipid binding | 65 out of 7474 genes, 0.9% | 441 out of 40099 genes, 1.1% | 1 | View Result |
| cysteine-type peptidase activity | 33 out of 7474 genes, 0.4% | 245 out of 40099 genes, 0.6% | 1 | View Result |
| 3'-5' exonuclease activity | 16 out of 7474 genes, 0.2% | 135 out of 40099 genes, 0.3% | 1 | View Result |
| heterocyclic compound binding | 2844 out of 7474 genes, 38.1% | 15714 out of 40099 genes, 39.2% | 1 | View Result |
| aconitate hydratase activity | 11 out of 7474 genes, 0.1% | 101 out of 40099 genes, 0.3% | 1 | View Result |
| nucleotidyltransferase activity | 83 out of 7474 genes, 1.1% | 550 out of 40099 genes, 1.4% | 1 | View Result |
| Rab GTPase activator activity | 2 out of 7474 genes, 0.0% | 32 out of 40099 genes, 0.1% | 1 | View Result |
| peptidase activity | 241 out of 7474 genes, 3.2% | 1470 out of 40099 genes, 3.7% | 1 | View Result |
| organic cyclic compound binding | 2846 out of 7474 genes, 38.1% | 15741 out of 40099 genes, 39.3% | 1 | View Result |
| small protein activating enzyme activity | 3 out of 7474 genes, 0.0% | 42 out of 40099 genes, 0.1% | 1 | View Result |
| ligase activity, forming carbon-carbon bonds | 3 out of 7474 genes, 0.0% | 42 out of 40099 genes, 0.1% | 1 | View Result |
| ferric iron transmembrane transporter activity | 2 out of 7474 genes, 0.0% | 33 out of 40099 genes, 0.1% | 1 | View Result |
| ferric-transporting ATPase activity | 2 out of 7474 genes, 0.0% | 33 out of 40099 genes, 0.1% | 1 | View Result |
| trivalent inorganic cation transmembrane transporter activity | 2 out of 7474 genes, 0.0% | 33 out of 40099 genes, 0.1% | 1 | View Result |
| histone acetyltransferase activity | 9 out of 7474 genes, 0.1% | 89 out of 40099 genes, 0.2% | 1 | View Result |
| hydrolase activity, acting on carbon-nitrogen (but not peptide) bonds | 30 out of 7474 genes, 0.4% | 230 out of 40099 genes, 0.6% | 1 | View Result |
| MAP kinase activity | 6 out of 7474 genes, 0.1% | 67 out of 40099 genes, 0.2% | 1 | View Result |
| receptor binding | 6 out of 7474 genes, 0.1% | 68 out of 40099 genes, 0.2% | 1 | View Result |
| lipid transporter activity | 16 out of 7474 genes, 0.2% | 140 out of 40099 genes, 0.3% | 1 | View Result |
| aryl-aldehyde oxidase activity | 3 out of 7474 genes, 0.0% | 44 out of 40099 genes, 0.1% | 1 | View Result |
| ARF guanyl-nucleotide exchange factor activity | 2 out of 7474 genes, 0.0% | 35 out of 40099 genes, 0.1% | 1 | View Result |
| rRNA methyltransferase activity | 2 out of 7474 genes, 0.0% | 35 out of 40099 genes, 0.1% | 1 | View Result |
| exopeptidase activity | 37 out of 7474 genes, 0.5% | 279 out of 40099 genes, 0.7% | 1 | View Result |
| ATPase activity, coupled to transmembrane movement of ions | 97 out of 7474 genes, 1.3% | 646 out of 40099 genes, 1.6% | 1 | View Result |
| anion binding | 1399 out of 7474 genes, 18.7% | 7917 out of 40099 genes, 19.7% | 1 | View Result |
| phospholipase D activity | 3 out of 7474 genes, 0.0% | 45 out of 40099 genes, 0.1% | 1 | View Result |
| N-acyltransferase activity | 20 out of 7474 genes, 0.3% | 170 out of 40099 genes, 0.4% | 1 | View Result |
| nucleoside-triphosphatase regulator activity | 30 out of 7474 genes, 0.4% | 236 out of 40099 genes, 0.6% | 1 | View Result |
| O-acyltransferase activity | 16 out of 7474 genes, 0.2% | 143 out of 40099 genes, 0.4% | 1 | View Result |
| metalloendopeptidase activity | 22 out of 7474 genes, 0.3% | 184 out of 40099 genes, 0.5% | 1 | View Result |
| aldehyde dehydrogenase (NAD) activity | 8 out of 7474 genes, 0.1% | 86 out of 40099 genes, 0.2% | 1 | View Result |
| calcium ion transmembrane transporter activity | 15 out of 7474 genes, 0.2% | 137 out of 40099 genes, 0.3% | 1 | View Result |
| myosin heavy chain kinase activity | 7 out of 7474 genes, 0.1% | 79 out of 40099 genes, 0.2% | 1 | View Result |
| ATP-dependent peptidase activity | 8 out of 7474 genes, 0.1% | 88 out of 40099 genes, 0.2% | 1 | View Result |
| double-stranded RNA binding | 4 out of 7474 genes, 0.1% | 56 out of 40099 genes, 0.1% | 1 | View Result |
| binding | 4536 out of 7474 genes, 60.7% | 24872 out of 40099 genes, 62.0% | 1 | View Result |
| enzyme activator activity | 24 out of 7474 genes, 0.3% | 201 out of 40099 genes, 0.5% | 1 | View Result |
| RNA methyltransferase activity | 6 out of 7474 genes, 0.1% | 73 out of 40099 genes, 0.2% | 1 | View Result |
| amino acid kinase activity | 2 out of 7474 genes, 0.0% | 38 out of 40099 genes, 0.1% | 1 | View Result |
| mRNA 5'-UTR binding | 2 out of 7474 genes, 0.0% | 38 out of 40099 genes, 0.1% | 1 | View Result |
| 2 iron, 2 sulfur cluster binding | 10 out of 7474 genes, 0.1% | 104 out of 40099 genes, 0.3% | 1 | View Result |
| endonuclease activity | 23 out of 7474 genes, 0.3% | 195 out of 40099 genes, 0.5% | 1 | View Result |
| acetylglucosaminyltransferase activity | 13 out of 7474 genes, 0.2% | 126 out of 40099 genes, 0.3% | 1 | View Result |
| protein kinase regulator activity | 4 out of 7474 genes, 0.1% | 57 out of 40099 genes, 0.1% | 1 | View Result |
| GTPase regulator activity | 28 out of 7474 genes, 0.4% | 229 out of 40099 genes, 0.6% | 1 | View Result |
| aldose 1-epimerase activity | 3 out of 7474 genes, 0.0% | 49 out of 40099 genes, 0.1% | 1 | View Result |
| phospholipid transporter activity | 8 out of 7474 genes, 0.1% | 92 out of 40099 genes, 0.2% | 1 | View Result |
| aldehyde oxidase activity | 3 out of 7474 genes, 0.0% | 51 out of 40099 genes, 0.1% | 1 | View Result |
| oligopeptide transporter activity | 3 out of 7474 genes, 0.0% | 51 out of 40099 genes, 0.1% | 1 | View Result |
| phosphoric diester hydrolase activity | 18 out of 7474 genes, 0.2% | 167 out of 40099 genes, 0.4% | 1 | View Result |
| inorganic cation transmembrane transporter activity | 152 out of 7474 genes, 2.0% | 999 out of 40099 genes, 2.5% | 1 | View Result |
| calcium-transporting ATPase activity | 10 out of 7474 genes, 0.1% | 110 out of 40099 genes, 0.3% | 1 | View Result |
| ion transmembrane transporter activity | 320 out of 7474 genes, 4.3% | 1976 out of 40099 genes, 4.9% | 1 | View Result |
| phosphatidylinositol binding | 18 out of 7474 genes, 0.2% | 169 out of 40099 genes, 0.4% | 1 | View Result |
| iron-sulfur cluster binding | 53 out of 7474 genes, 0.7% | 400 out of 40099 genes, 1.0% | 1 | View Result |
| metal cluster binding | 53 out of 7474 genes, 0.7% | 400 out of 40099 genes, 1.0% | 1 | View Result |
| cation transmembrane transporter activity | 229 out of 7474 genes, 3.1% | 1453 out of 40099 genes, 3.6% | 1 | View Result |
| GTPase activator activity | 12 out of 7474 genes, 0.2% | 126 out of 40099 genes, 0.3% | 1 | View Result |
| N-acetyltransferase activity | 15 out of 7474 genes, 0.2% | 149 out of 40099 genes, 0.4% | 1 | View Result |
| Rho GTPase activator activity | 4 out of 7474 genes, 0.1% | 63 out of 40099 genes, 0.2% | 1 | View Result |
| ubiquitin-protein ligase activity | 85 out of 7474 genes, 1.1% | 602 out of 40099 genes, 1.5% | 1 | View Result |
| 1,3-beta-D-glucan synthase activity | 7 out of 7474 genes, 0.1% | 89 out of 40099 genes, 0.2% | 1 | View Result |
| small conjugating protein ligase activity | 87 out of 7474 genes, 1.2% | 615 out of 40099 genes, 1.5% | 1 | View Result |
| ion binding | 2391 out of 7474 genes, 32.0% | 13429 out of 40099 genes, 33.5% | 1 | View Result |
| oxidoreductase activity, acting on CH or CH2 groups, with an iron-sulfur protein as acceptor | 4 out of 7474 genes, 0.1% | 65 out of 40099 genes, 0.2% | 1 | View Result |
| magnesium ion transmembrane transporter activity | 4 out of 7474 genes, 0.1% | 66 out of 40099 genes, 0.2% | 1 | View Result |
| acid-amino acid ligase activity | 103 out of 7474 genes, 1.4% | 723 out of 40099 genes, 1.8% | 1 | View Result |
| aminopeptidase activity | 9 out of 7474 genes, 0.1% | 110 out of 40099 genes, 0.3% | 1 | View Result |
| purine nucleoside binding | 1199 out of 7474 genes, 16.0% | 6937 out of 40099 genes, 17.3% | 1 | View Result |
| purine ribonucleoside binding | 1199 out of 7474 genes, 16.0% | 6937 out of 40099 genes, 17.3% | 1 | View Result |
| purine ribonucleotide binding | 1199 out of 7474 genes, 16.0% | 6938 out of 40099 genes, 17.3% | 1 | View Result |
| purine nucleotide binding | 1202 out of 7474 genes, 16.1% | 6956 out of 40099 genes, 17.3% | 1 | View Result |
| mannosidase activity | 3 out of 7474 genes, 0.0% | 59 out of 40099 genes, 0.1% | 1 | View Result |
| nucleoside binding | 1201 out of 7474 genes, 16.1% | 6957 out of 40099 genes, 17.3% | 1 | View Result |
| nucleotide binding | 1813 out of 7474 genes, 24.3% | 10323 out of 40099 genes, 25.7% | 1 | View Result |
| nucleoside phosphate binding | 1813 out of 7474 genes, 24.3% | 10323 out of 40099 genes, 25.7% | 1 | View Result |
| cation-transporting ATPase activity | 72 out of 7474 genes, 1.0% | 536 out of 40099 genes, 1.3% | 1 | View Result |
| phospholipid-translocating ATPase activity | 6 out of 7474 genes, 0.1% | 87 out of 40099 genes, 0.2% | 1 | View Result |
| ribonucleoside binding | 1200 out of 7474 genes, 16.1% | 6955 out of 40099 genes, 17.3% | 1 | View Result |
| purine ribonucleoside triphosphate binding | 1177 out of 7474 genes, 15.7% | 6828 out of 40099 genes, 17.0% | 1 | View Result |
| transition metal ion binding | 621 out of 7474 genes, 8.3% | 3727 out of 40099 genes, 9.3% | 1 | View Result |
| protein self-association | 5 out of 7474 genes, 0.1% | 79 out of 40099 genes, 0.2% | 1 | View Result |
| substrate-specific transporter activity | 429 out of 7474 genes, 5.7% | 2640 out of 40099 genes, 6.6% | 1 | View Result |
| small molecule binding | 1838 out of 7474 genes, 24.6% | 10472 out of 40099 genes, 26.1% | 1 | View Result |
| ribonucleotide binding | 1215 out of 7474 genes, 16.3% | 7048 out of 40099 genes, 17.6% | 1 | View Result |
| exonuclease activity | 21 out of 7474 genes, 0.3% | 204 out of 40099 genes, 0.5% | 1 | View Result |
| acetyltransferase activity | 22 out of 7474 genes, 0.3% | 213 out of 40099 genes, 0.5% | 1 | View Result |
| cation binding | 1153 out of 7474 genes, 15.4% | 6722 out of 40099 genes, 16.8% | 1 | View Result |
| ligase activity, forming carbon-nitrogen bonds | 140 out of 7474 genes, 1.9% | 967 out of 40099 genes, 2.4% | 1 | View Result |
| metallopeptidase activity | 37 out of 7474 genes, 0.5% | 320 out of 40099 genes, 0.8% | 1 | View Result |
| metal ion binding | 1122 out of 7474 genes, 15.0% | 6560 out of 40099 genes, 16.4% | 1 | View Result |
| transferase activity, transferring phosphorus-containing groups | 990 out of 7474 genes, 13.2% | 5827 out of 40099 genes, 14.5% | 1 | View Result |
| hydrolase activity, acting on ester bonds | 360 out of 7474 genes, 4.8% | 2266 out of 40099 genes, 5.7% | 1 | View Result |
| citrate hydro-lyase (cis-aconitate-forming) activity | 4 out of 7474 genes, 0.1% | 75 out of 40099 genes, 0.2% | 1 | View Result |
| isocitrate hydro-lyase (cis-aconitate-forming) activity | 4 out of 7474 genes, 0.1% | 75 out of 40099 genes, 0.2% | 1 | View Result |
| Ras GTPase activator activity | 6 out of 7474 genes, 0.1% | 95 out of 40099 genes, 0.2% | 1 | View Result |
| protein kinase activity | 583 out of 7474 genes, 7.8% | 3553 out of 40099 genes, 8.9% | 1 | View Result |
| sequence-specific DNA binding transcription factor activity | 159 out of 7474 genes, 2.1% | 1093 out of 40099 genes, 2.7% | 1 | View Result |
| kinase activity | 814 out of 7474 genes, 10.9% | 4860 out of 40099 genes, 12.1% | 1 | View Result |
| nucleic acid binding transcription factor activity | 160 out of 7474 genes, 2.1% | 1101 out of 40099 genes, 2.7% | 1 | View Result |
| nuclease activity | 53 out of 7474 genes, 0.7% | 434 out of 40099 genes, 1.1% | 1 | View Result |
| calmodulin-dependent protein kinase activity | 29 out of 7474 genes, 0.4% | 274 out of 40099 genes, 0.7% | 1 | View Result |
| protein transporter activity | 32 out of 7474 genes, 0.4% | 302 out of 40099 genes, 0.8% | 1 | View Result |
| ATPase activity, coupled to transmembrane movement of ions, phosphorylative mechanism | 29 out of 7474 genes, 0.4% | 288 out of 40099 genes, 0.7% | 1 | View Result |
| protein serine/threonine phosphatase activity | 45 out of 7474 genes, 0.6% | 402 out of 40099 genes, 1.0% | 1 | View Result |
| phosphatase activity | 130 out of 7474 genes, 1.7% | 952 out of 40099 genes, 2.4% | 1 | View Result |
| phosphotransferase activity, alcohol group as acceptor | 645 out of 7474 genes, 8.6% | 3981 out of 40099 genes, 9.9% | 1 | View Result |
| protein serine/threonine kinase activity | 374 out of 7474 genes, 5.0% | 2420 out of 40099 genes, 6.0% | 1 | View Result |
| phosphoprotein phosphatase activity | 77 out of 7474 genes, 1.0% | 625 out of 40099 genes, 1.6% | 1 | View Result |
| S-adenosylmethionine-dependent methyltransferase activity | 32 out of 7474 genes, 0.4% | 325 out of 40099 genes, 0.8% | 1 | View Result |
| N-methyltransferase activity | 11 out of 7474 genes, 0.1% | 166 out of 40099 genes, 0.4% | 1 | View Result |
| arginine N-methyltransferase activity | 2 out of 7474 genes, 0.0% | 77 out of 40099 genes, 0.2% | 1 | View Result |
| protein-arginine N-methyltransferase activity | 2 out of 7474 genes, 0.0% | 77 out of 40099 genes, 0.2% | 1 | View Result |
| phospholipid binding | 32 out of 7474 genes, 0.4% | 330 out of 40099 genes, 0.8% | 1 | View Result |
| transferase activity | 1739 out of 7474 genes, 23.3% | 10166 out of 40099 genes, 25.4% | 1 | View Result |
| histone methyltransferase activity | 6 out of 7474 genes, 0.1% | 123 out of 40099 genes, 0.3% | 1 | View Result |
| aminoacyl-tRNA ligase activity | 18 out of 7474 genes, 0.2% | 227 out of 40099 genes, 0.6% | 1 | View Result |
| adenyl nucleotide binding | 993 out of 7474 genes, 13.3% | 6033 out of 40099 genes, 15.0% | 1 | View Result |
| ligase activity, forming carbon-oxygen bonds | 18 out of 7474 genes, 0.2% | 232 out of 40099 genes, 0.6% | 1 | View Result |
| ligase activity, forming aminoacyl-tRNA and related compounds | 18 out of 7474 genes, 0.2% | 232 out of 40099 genes, 0.6% | 1 | View Result |
| adenyl ribonucleotide binding | 991 out of 7474 genes, 13.3% | 6029 out of 40099 genes, 15.0% | 1 | View Result |
| protein methyltransferase activity | 9 out of 7474 genes, 0.1% | 160 out of 40099 genes, 0.4% | 1 | View Result |
| phosphoric ester hydrolase activity | 149 out of 7474 genes, 2.0% | 1126 out of 40099 genes, 2.8% | 1 | View Result |
| ATP binding | 967 out of 7474 genes, 12.9% | 5917 out of 40099 genes, 14.8% | 1 | View Result |
| ligase activity | 223 out of 7474 genes, 3.0% | 1658 out of 40099 genes, 4.1% | 1 | View Result |
| methyltransferase activity | 87 out of 7474 genes, 1.2% | 788 out of 40099 genes, 2.0% | 1 | View Result |
| transferase activity, transferring one-carbon groups | 89 out of 7474 genes, 1.2% | 813 out of 40099 genes, 2.0% | 1 | View Result |
| zinc ion binding | 286 out of 7474 genes, 3.8% | 2194 out of 40099 genes, 5.5% | 1 | View Result |

| Gene Ontology term | Genes annotated to the term |
| --- | --- |
| structural molecule activity | CL2222.Contig1\_All, CL12504.Contig2\_All, Unigene32114\_All, Unigene14305\_All, CL5814.Contig2\_All, CL1339.Contig5\_All, CL7290.Contig1\_All, CL6620.Contig1\_All, CL4943.Contig2\_All, CL1338.Contig1\_All, CL11227.Contig2\_All, CL7850.Contig1\_All, CL4661.Contig2\_All, CL116.Contig3\_All, CL8213.Contig3\_All, CL565.Contig5\_All, CL11610.Contig2\_All, Unigene6078\_All, CL54.Contig2\_All, Unigene31443\_All, Unigene55306\_All, CL10884.Contig1\_All, CL6156.Contig1\_All, CL386.Contig2\_All, Unigene43894\_All, Unigene61423\_All, CL1397.Contig2\_All, Unigene6440\_All, CL7290.Contig2\_All, CL9899.Contig1\_All, Unigene2148\_All, CL4661.Contig3\_All, CL943.Contig3\_All, CL2300.Contig1\_All, Unigene28643\_All, CL12452.Contig2\_All, CL5814.Contig1\_All, CL13747.Contig2\_All, Unigene15881\_All, Unigene8497\_All, Unigene56244\_All, Unigene2386\_All, Unigene1036\_All, CL484.Contig5\_All, Unigene16373\_All, CL10104.Contig2\_All, Unigene28167\_All, Unigene25552\_All, Unigene4385\_All, CL1449.Contig2\_All, CL13487.Contig2\_All, Unigene56915\_All, CL7514.Contig2\_All, CL10937.Contig2\_All, CL1808.Contig2\_All, Unigene24849\_All, Unigene24742\_All, Unigene18911\_All, CL11693.Contig2\_All, Unigene19828\_All, Unigene39099\_All, CL8662.Contig4\_All, Unigene901\_All, CL2586.Contig1\_All, CL4354.Contig1\_All, CL7101.Contig1\_All, CL1438.Contig2\_All, CL2339.Contig1\_All, CL90.Contig3\_All, Unigene56245\_All, CL8744.Contig5\_All, CL11948.Contig2\_All, Unigene17539\_All, CL854.Contig3\_All, CL11803.Contig2\_All, CL10837.Contig2\_All, Unigene17239\_All, CL7850.Contig2\_All, Unigene15423\_All, CL3466.Contig1\_All, Unigene36120\_All, Unigene27328\_All, Unigene8877\_All, CL2641.Contig4\_All, Unigene26301\_All, CL7864.Contig1\_All, Unigene63219\_All, CL6318.Contig3\_All, CL12585.Contig1\_All, Unigene32076\_All, CL10876.Contig1\_All, CL10047.Contig1\_All, Unigene16408\_All, Unigene39287\_All, Unigene29776\_All, CL1567.Contig1\_All, CL6620.Contig2\_All, Unigene35736\_All, Unigene42315\_All, Unigene23065\_All, CL8848.Contig2\_All, Unigene63405\_All, CL2642.Contig2\_All, CL7336.Contig3\_All, CL11726.Contig1\_All, Unigene16581\_All, Unigene6614\_All, Unigene17593\_All, Unigene25625\_All, Unigene12757\_All, CL9824.Contig1\_All, Unigene43614\_All, Unigene62150\_All, CL6318.Contig1\_All, CL10950.Contig2\_All, Unigene32999\_All, CL2752.Contig2\_All, CL508.Contig2\_All, Unigene42555\_All, CL11526.Contig1\_All, Unigene2584\_All, Unigene41288\_All, Unigene4820\_All, CL7864.Contig2\_All, Unigene29240\_All, CL5794.Contig2\_All, Unigene31710\_All, Unigene7068\_All, Unigene6780\_All, CL5128.Contig3\_All, CL10328.Contig2\_All, CL5052.Contig2\_All, Unigene40435\_All, CL10859.Contig1\_All, CL461.Contig3\_All, CL11773.Contig1\_All, CL1849.Contig2\_All, Unigene30370\_All, CL5666.Contig1\_All, Unigene22240\_All, CL2463.Contig2\_All, CL852.Contig4\_All, CL8081.Contig1\_All, CL10096.Contig1\_All, CL6238.Contig6\_All, CL2988.Contig2\_All, CL717.Contig1\_All, CL1808.Contig1\_All, Unigene57094\_All, CL9457.Contig2\_All, Unigene29652\_All, Unigene6077\_All, Unigene2518\_All, CL386.Contig1\_All, Unigene9264\_All, CL3897.Contig1\_All, Unigene24498\_All, CL7996.Contig2\_All, Unigene12231\_All, Unigene8825\_All, Unigene33046\_All, Unigene56692\_All, Unigene61614\_All, Unigene36210\_All, Unigene32702\_All, Unigene15519\_All, Unigene58336\_All, Unigene33863\_All, CL5652.Contig1\_All, CL6218.Contig1\_All, CL4087.Contig1\_All, CL711.Contig2\_All, CL13689.Contig3\_All, CL4437.Contig2\_All, Unigene40215\_All, CL4604.Contig3\_All, Unigene23581\_All, CL4701.Contig2\_All, Unigene12043\_All, CL1152.Contig4\_All, Unigene11948\_All, CL10986.Contig2\_All, Unigene18909\_All, CL4734.Contig4\_All, Unigene19923\_All, CL1249.Contig1\_All, Unigene62791\_All, CL12385.Contig1\_All, CL717.Contig2\_All, Unigene61540\_All, Unigene57006\_All, CL3060.Contig4\_All, Unigene13127\_All, Unigene29010\_All, CL2586.Contig2\_All, CL11510.Contig1\_All, CL1152.Contig2\_All, Unigene7773\_All, Unigene8532\_All, Unigene41308\_All, CL12183.Contig2\_All, CL4734.Contig8\_All, CL6667.Contig2\_All, CL2468.Contig1\_All, Unigene39837\_All, Unigene39288\_All, CL8720.Contig1\_All, Unigene18802\_All, CL2964.Contig12\_All, CL2752.Contig1\_All, Unigene4726\_All, CL5454.Contig1\_All, CL2641.Contig5\_All, CL4159.Contig2\_All, CL13218.Contig1\_All, CL12523.Contig3\_All, CL1148.Contig1\_All, CL6163.Contig3\_All, CL639.Contig2\_All, Unigene42725\_All, Unigene17153\_All, Unigene34402\_All, Unigene57766\_All, CL5945.Contig3\_All, CL5949.Contig1\_All, CL3681.Contig1\_All, Unigene6437\_All, Unigene7018\_All, Unigene37205\_All, Unigene13296\_All, Unigene42439\_All, CL13955.Contig2\_All, Unigene30297\_All, CL11927.Contig2\_All, Unigene26320\_All, CL5555.Contig2\_All, Unigene16469\_All, Unigene28704\_All, CL13011.Contig1\_All, Unigene15486\_All, Unigene6168\_All, CL12218.Contig1\_All, CL3880.Contig3\_All, CL3696.Contig2\_All, Unigene15631\_All, CL4492.Contig1\_All, CL484.Contig1\_All, Unigene55970\_All, Unigene11101\_All, CL1477.Contig2\_All, CL326.Contig2\_All, Unigene34301\_All, CL5949.Contig2\_All, Unigene7340\_All, CL3060.Contig1\_All, CL2730.Contig1\_All, CL8384.Contig2\_All, Unigene6920\_All, CL1597.Contig1\_All, Unigene26124\_All, CL11059.Contig1\_All, CL2829.Contig1\_All, CL7725.Contig1\_All, CL2555.Contig3\_All, Unigene33360\_All, Unigene19894\_All, Unigene62016\_All, CL2040.Contig1\_All, CL2339.Contig2\_All, CL2300.Contig4\_All, CL3603.Contig5\_All, Unigene14921\_All, CL13403.Contig1\_All, CL6238.Contig4\_All, CL10563.Contig1\_All, Unigene19421\_All, Unigene60000\_All, Unigene61227\_All, CL371.Contig1\_All, Unigene4693\_All, CL9204.Contig1\_All, CL2964.Contig13\_All, Unigene21939\_All, CL4137.Contig3\_All, CL3698.Contig5\_All, Unigene59302\_All, CL14005.Contig1\_All, Unigene60270\_All, Unigene29598\_All, Unigene5929\_All, Unigene42983\_All, Unigene23043\_All, Unigene57776\_All, Unigene2429\_All, CL2964.Contig3\_All, CL905.Contig3\_All, CL7997.Contig2\_All, CL13218.Contig2\_All, CL5683.Contig2\_All, Unigene15552\_All, CL8846.Contig1\_All, Unigene11514\_All, Unigene8801\_All, Unigene42338\_All, CL91.Contig6\_All, Unigene24131\_All, CL3191.Contig3\_All, Unigene42882\_All, Unigene61986\_All, CL11693.Contig1\_All, Unigene22230\_All, Unigene29705\_All, CL2339.Contig5\_All, Unigene16255\_All, Unigene44963\_All, CL3269.Contig6\_All, CL12624.Contig1\_All, CL13899.Contig2\_All, Unigene26040\_All, Unigene21808\_All, Unigene15896\_All, CL7936.Contig1\_All, CL3143.Contig1\_All, CL6335.Contig1\_All, CL6815.Contig3\_All, CL3295.Contig2\_All, CL2555.Contig2\_All, Unigene24605\_All, CL4159.Contig1\_All, CL7564.Contig1\_All, Unigene4831\_All, CL12878.Contig1\_All, CL2048.Contig4\_All, CL6385.Contig3\_All, CL13689.Contig2\_All, CL6724.Contig2\_All, Unigene19130\_All, Unigene9453\_All, CL4828.Contig4\_All, CL10853.Contig2\_All, Unigene6347\_All, Unigene58013\_All, CL12348.Contig1\_All, CL8473.Contig1\_All, Unigene22171\_All, Unigene9339\_All, CL1782.Contig3\_All, CL9574.Contig1\_All, Unigene56781\_All, CL4413.Contig1\_All, Unigene55017\_All, Unigene56188\_All, Unigene55213\_All, Unigene29291\_All, Unigene22212\_All, CL26.Contig3\_All, CL11275.Contig2\_All, CL7042.Contig2\_All, CL10701.Contig1\_All, Unigene19811\_All, CL13899.Contig5\_All, CL3284.Contig4\_All, CL7336.Contig4\_All, CL1071.Contig2\_All, CL13949.Contig1\_All, CL1026.Contig1\_All, CL6052.Contig1\_All, CL3269.Contig2\_All, Unigene29818\_All, Unigene32311\_All, CL8252.Contig1\_All, CL13850.Contig1\_All, Unigene10448\_All, CL784.Contig4\_All, CL10620.Contig2\_All, CL11478.Contig1\_All, CL12585.Contig2\_All, Unigene19302\_All, Unigene32180\_All, CL12385.Contig2\_All, CL3173.Contig3\_All, Unigene19008\_All, CL13545.Contig1\_All, CL10794.Contig2\_All, CL7636.Contig2\_All, CL12065.Contig1\_All, CL10844.Contig3\_All, CL7636.Contig1\_All, Unigene16117\_All, Unigene22803\_All, CL386.Contig3\_All, CL2079.Contig2\_All, Unigene21196\_All, CL3875.Contig1\_All, CL13295.Contig2\_All, CL9503.Contig1\_All, Unigene7402\_All, Unigene10801\_All, CL10853.Contig1\_All, Unigene35629\_All, Unigene57619\_All, CL9779.Contig1\_All, CL5922.Contig1\_All, CL80.Contig1\_All, Unigene43063\_All, Unigene16128\_All, CL114.Contig2\_All, Unigene58710\_All, Unigene33649\_All, Unigene42825\_All, Unigene3781\_All, CL6519.Contig2\_All, CL1911.Contig7\_All, Unigene805\_All, Unigene33862\_All, Unigene42356\_All, Unigene58861\_All, Unigene25790\_All, CL6398.Contig2\_All, CL10794.Contig1\_All, Unigene54729\_All, CL6406.Contig2\_All, Unigene64332\_All, CL4420.Contig1\_All, Unigene60364\_All, Unigene8052\_All, CL4068.Contig2\_All, CL12437.Contig2\_All, CL2163.Contig2\_All, CL4794.Contig1\_All, Unigene2805\_All, CL1230.Contig4\_All, Unigene32983\_All, Unigene28775\_All, CL4087.Contig2\_All, CL7514.Contig1\_All, CL9858.Contig4\_All, Unigene40509\_All, CL5206.Contig1\_All, CL90.Contig2\_All, Unigene22417\_All, CL8314.Contig2\_All, Unigene13534\_All, Unigene11830\_All, CL3269.Contig1\_All, Unigene4672\_All, Unigene58622\_All, Unigene22244\_All, CL5487.Contig1\_All, CL6563.Contig1\_All, CL1249.Contig2\_All, Unigene41968\_All, CL1771.Contig3\_All, CL13437.Contig3\_All, CL10514.Contig2\_All, Unigene22337\_All, Unigene2030\_All, Unigene17552\_All, Unigene40018\_All, CL3191.Contig2\_All, CL4364.Contig2\_All, CL7336.Contig1\_All, Unigene41182\_All, Unigene19364\_All, CL1911.Contig6\_All, CL10453.Contig1\_All, Unigene22846\_All, CL1449.Contig1\_All, Unigene23138\_All, CL12218.Contig2\_All, Unigene60032\_All, CL7704.Contig1\_All, Unigene62562\_All, CL644.Contig3\_All, CL10604.Contig3\_All, Unigene13280\_All, CL3979.Contig3\_All, Unigene39022\_All, CL9824.Contig2\_All, CL5794.Contig3\_All, CL11592.Contig2\_All, Unigene56615\_All, Unigene2737\_All, CL1652.Contig2\_All, Unigene10138\_All, CL1849.Contig1\_All, CL7336.Contig2\_All, Unigene25956\_All, Unigene55757\_All, CL1461.Contig3\_All, CL2964.Contig8\_All, CL12606.Contig2\_All, Unigene56776\_All, CL5052.Contig1\_All, Unigene44184\_All, CL3698.Contig3\_All, CL9738.Contig1\_All, Unigene36244\_All, Unigene34889\_All, CL7461.Contig2\_All, Unigene25614\_All, Unigene15487\_All, CL1461.Contig2\_All, Unigene42502\_All, Unigene2757\_All, Unigene22228\_All, Unigene29415\_All, Unigene18885\_All, Unigene15951\_All, CL18.Contig4\_All, Unigene33359\_All, Unigene7840\_All, CL3305.Contig1\_All, CL5863.Contig3\_All, Unigene15744\_All, CL9204.Contig4\_All, Unigene11284\_All, CL11673.Contig1\_All, Unigene29658\_All, CL8531.Contig1\_All, CL12583.Contig1\_All, Unigene39461\_All, CL2222.Contig2\_All, CL4230.Contig1\_All, CL3295.Contig4\_All, Unigene55113\_All, CL1911.Contig9\_All, CL9574.Contig3\_All, CL12894.Contig1\_All, CL644.Contig4\_All, Unigene6000\_All, CL6519.Contig1\_All, CL14004.Contig2\_All, CL2468.Contig2\_All, Unigene29178\_All, Unigene62163\_All, Unigene16256\_All, Unigene11244\_All, CL11610.Contig1\_All, CL1.Contig7\_All, CL4492.Contig2\_All, CL8081.Contig2\_All, Unigene1390\_All, CL12624.Contig3\_All, Unigene57337\_All, Unigene8237\_All, CL4069.Contig2\_All, CL780.Contig2\_All, Unigene36053\_All, CL7262.Contig1\_All, Unigene3111\_All, CL3583.Contig1\_All, Unigene34596\_All, CL4701.Contig1\_All, CL10098.Contig1\_All, CL3269.Contig3\_All, CL6815.Contig2\_All, CL8696.Contig1\_All, Unigene2577\_All, CL1339.Contig4\_All, CL5988.Contig3\_All, CL10884.Contig2\_All, Unigene36628\_All, CL10105.Contig1\_All, Unigene38556\_All, Unigene55668\_All, Unigene62007\_All, Unigene4536\_All, Unigene63999\_All, CL9351.Contig2\_All, Unigene28819\_All, CL1911.Contig8\_All, Unigene13493\_All, CL9050.Contig1\_All, Unigene34358\_All, CL13403.Contig2\_All, Unigene23980\_All, Unigene2225\_All, Unigene57765\_All, Unigene2850\_All, Unigene55797\_All, CL9141.Contig1\_All, Unigene39954\_All, CL9193.Contig1\_All, CL12178.Contig1\_All, CL367.Contig1\_All, Unigene32598\_All, Unigene46996\_All, Unigene23149\_All, CL4661.Contig1\_All, CL1911.Contig10\_All, CL2339.Contig4\_All, Unigene2295\_All, CL7444.Contig1\_All, Unigene41232\_All, Unigene60594\_All, CL3583.Contig2\_All, Unigene60249\_All, CL3880.Contig2\_All, CL12643.Contig2\_All, CL565.Contig2\_All, CL4978.Contig2\_All, CL4069.Contig1\_All, Unigene15946\_All, Unigene7670\_All, Unigene24438\_All, Unigene4181\_All, Unigene35447\_All, CL91.Contig9\_All, Unigene62219\_All, Unigene23259\_All, CL7997.Contig1\_All, Unigene12825\_All, CL13299.Contig4\_All, Unigene3390\_All, CL9799.Contig1\_All, CL2163.Contig1\_All, Unigene36121\_All, CL1782.Contig5\_All, Unigene55877\_All, CL3804.Contig2\_All, CL9050.Contig2\_All, CL4226.Contig2\_All, CL1948.Contig3\_All, CL1230.Contig2\_All, CL7073.Contig1\_All, Unigene25491\_All, Unigene19846\_All, Unigene31948\_All, Unigene61202\_All, CL8918.Contig1\_All, CL12820.Contig1\_All, CL5945.Contig4\_All, Unigene56276\_All, Unigene39218\_All, Unigene41921\_All, CL854.Contig4\_All, Unigene42188\_All, CL5652.Contig2\_All, Unigene16516\_All, CL11693.Contig3\_All, Unigene10707\_All, Unigene24550\_All, CL644.Contig1\_All, Unigene31212\_All, CL2339.Contig3\_All, Unigene22234\_All, Unigene3774\_All, CL7725.Contig2\_All, CL4909.Contig2\_All, Unigene22940\_All, Unigene45573\_All, Unigene56322\_All, CL10934.Contig2\_All, CL3949.Contig1\_All, Unigene60318\_All, CL2048.Contig3\_All, Unigene42446\_All, Unigene16309\_All, Unigene22291\_All, CL2048.Contig2\_All, CL7996.Contig3\_All |
| structural constituent of ribosome | CL2222.Contig1\_All, CL12504.Contig2\_All, Unigene32114\_All, CL5814.Contig2\_All, CL1339.Contig5\_All, CL7290.Contig1\_All, CL6620.Contig1\_All, CL1338.Contig1\_All, CL11227.Contig2\_All, CL7850.Contig1\_All, CL4661.Contig2\_All, CL116.Contig3\_All, CL565.Contig5\_All, CL11610.Contig2\_All, Unigene6078\_All, CL54.Contig2\_All, Unigene55306\_All, CL10884.Contig1\_All, CL6156.Contig1\_All, CL386.Contig2\_All, Unigene43894\_All, Unigene61423\_All, CL1397.Contig2\_All, Unigene6440\_All, CL7290.Contig2\_All, CL9899.Contig1\_All, Unigene2148\_All, CL4661.Contig3\_All, CL943.Contig3\_All, CL2300.Contig1\_All, Unigene28643\_All, CL12452.Contig2\_All, CL5814.Contig1\_All, CL13747.Contig2\_All, Unigene15881\_All, Unigene8497\_All, Unigene1036\_All, CL484.Contig5\_All, CL10104.Contig2\_All, Unigene28167\_All, Unigene25552\_All, Unigene4385\_All, CL1449.Contig2\_All, CL13487.Contig2\_All, CL7514.Contig2\_All, CL10937.Contig2\_All, CL1808.Contig2\_All, Unigene24849\_All, Unigene24742\_All, Unigene18911\_All, CL11693.Contig2\_All, Unigene39099\_All, CL8662.Contig4\_All, CL2586.Contig1\_All, CL4354.Contig1\_All, CL7101.Contig1\_All, CL1438.Contig2\_All, CL2339.Contig1\_All, CL90.Contig3\_All, CL8744.Contig5\_All, CL11948.Contig2\_All, Unigene17539\_All, CL854.Contig3\_All, CL11803.Contig2\_All, CL10837.Contig2\_All, Unigene17239\_All, CL7850.Contig2\_All, Unigene15423\_All, CL3466.Contig1\_All, Unigene36120\_All, Unigene27328\_All, Unigene8877\_All, Unigene26301\_All, Unigene63219\_All, CL6318.Contig3\_All, CL12585.Contig1\_All, Unigene32076\_All, CL10876.Contig1\_All, CL10047.Contig1\_All, Unigene16408\_All, Unigene39287\_All, Unigene29776\_All, CL1567.Contig1\_All, Unigene35736\_All, CL6620.Contig2\_All, Unigene42315\_All, Unigene23065\_All, CL8848.Contig2\_All, Unigene63405\_All, CL2642.Contig2\_All, CL7336.Contig3\_All, CL11726.Contig1\_All, Unigene16581\_All, Unigene6614\_All, Unigene17593\_All, Unigene25625\_All, CL9824.Contig1\_All, Unigene62150\_All, CL6318.Contig1\_All, CL10950.Contig2\_All, Unigene32999\_All, CL2752.Contig2\_All, CL508.Contig2\_All, Unigene42555\_All, CL11526.Contig1\_All, Unigene2584\_All, Unigene4820\_All, Unigene29240\_All, CL5794.Contig2\_All, Unigene31710\_All, Unigene7068\_All, CL5128.Contig3\_All, CL10328.Contig2\_All, CL5052.Contig2\_All, Unigene40435\_All, CL10859.Contig1\_All, CL461.Contig3\_All, CL11773.Contig1\_All, CL1849.Contig2\_All, Unigene30370\_All, CL5666.Contig1\_All, Unigene22240\_All, CL2463.Contig2\_All, CL852.Contig4\_All, CL8081.Contig1\_All, CL10096.Contig1\_All, CL6238.Contig6\_All, CL2988.Contig2\_All, CL717.Contig1\_All, CL1808.Contig1\_All, Unigene57094\_All, CL9457.Contig2\_All, Unigene29652\_All, Unigene6077\_All, Unigene2518\_All, CL386.Contig1\_All, Unigene9264\_All, CL3897.Contig1\_All, Unigene24498\_All, CL7996.Contig2\_All, Unigene12231\_All, Unigene8825\_All, Unigene33046\_All, Unigene56692\_All, Unigene61614\_All, Unigene36210\_All, Unigene32702\_All, Unigene15519\_All, Unigene58336\_All, CL6218.Contig1\_All, CL4087.Contig1\_All, CL711.Contig2\_All, CL13689.Contig3\_All, Unigene40215\_All, CL4604.Contig3\_All, Unigene23581\_All, CL4701.Contig2\_All, Unigene12043\_All, CL1152.Contig4\_All, Unigene11948\_All, CL10986.Contig2\_All, Unigene18909\_All, Unigene19923\_All, CL1249.Contig1\_All, Unigene62791\_All, CL12385.Contig1\_All, CL717.Contig2\_All, Unigene61540\_All, Unigene57006\_All, CL3060.Contig4\_All, Unigene13127\_All, Unigene29010\_All, CL2586.Contig2\_All, CL1152.Contig2\_All, Unigene8532\_All, CL6667.Contig2\_All, CL2468.Contig1\_All, Unigene39837\_All, Unigene39288\_All, CL8720.Contig1\_All, Unigene18802\_All, CL2752.Contig1\_All, Unigene4726\_All, CL5454.Contig1\_All, CL13218.Contig1\_All, CL12523.Contig3\_All, CL1148.Contig1\_All, CL6163.Contig3\_All, Unigene42725\_All, Unigene17153\_All, Unigene34402\_All, CL5945.Contig3\_All, CL5949.Contig1\_All, CL3681.Contig1\_All, Unigene6437\_All, Unigene7018\_All, Unigene37205\_All, Unigene13296\_All, Unigene42439\_All, CL13955.Contig2\_All, Unigene30297\_All, Unigene26320\_All, CL5555.Contig2\_All, Unigene16469\_All, Unigene28704\_All, CL13011.Contig1\_All, Unigene15486\_All, Unigene6168\_All, CL12218.Contig1\_All, CL3880.Contig3\_All, CL3696.Contig2\_All, Unigene15631\_All, CL4492.Contig1\_All, CL484.Contig1\_All, Unigene55970\_All, Unigene11101\_All, CL1477.Contig2\_All, CL326.Contig2\_All, Unigene34301\_All, CL5949.Contig2\_All, Unigene7340\_All, CL3060.Contig1\_All, CL2730.Contig1\_All, CL8384.Contig2\_All, Unigene6920\_All, CL1597.Contig1\_All, Unigene26124\_All, CL11059.Contig1\_All, CL2829.Contig1\_All, CL7725.Contig1\_All, CL2555.Contig3\_All, Unigene19894\_All, CL2040.Contig1\_All, Unigene62016\_All, CL2339.Contig2\_All, CL2300.Contig4\_All, CL3603.Contig5\_All, Unigene14921\_All, CL13403.Contig1\_All, CL6238.Contig4\_All, CL10563.Contig1\_All, Unigene61227\_All, Unigene60000\_All, CL371.Contig1\_All, Unigene4693\_All, CL9204.Contig1\_All, Unigene21939\_All, CL4137.Contig3\_All, CL3698.Contig5\_All, Unigene59302\_All, CL14005.Contig1\_All, Unigene60270\_All, Unigene29598\_All, Unigene42983\_All, Unigene23043\_All, Unigene57776\_All, Unigene2429\_All, CL905.Contig3\_All, CL7997.Contig2\_All, CL13218.Contig2\_All, CL5683.Contig2\_All, Unigene15552\_All, CL8846.Contig1\_All, Unigene11514\_All, Unigene8801\_All, Unigene42338\_All, Unigene24131\_All, CL3191.Contig3\_All, Unigene61986\_All, Unigene42882\_All, CL11693.Contig1\_All, Unigene22230\_All, Unigene29705\_All, CL2339.Contig5\_All, Unigene16255\_All, Unigene44963\_All, CL3269.Contig6\_All, Unigene26040\_All, Unigene21808\_All, Unigene15896\_All, CL7936.Contig1\_All, CL3143.Contig1\_All, CL6335.Contig1\_All, CL6815.Contig3\_All, CL3295.Contig2\_All, CL2555.Contig2\_All, Unigene24605\_All, CL7564.Contig1\_All, Unigene4831\_All, CL12878.Contig1\_All, CL2048.Contig4\_All, CL6385.Contig3\_All, CL13689.Contig2\_All, CL6724.Contig2\_All, Unigene19130\_All, Unigene9453\_All, CL4828.Contig4\_All, CL10853.Contig2\_All, Unigene58013\_All, CL12348.Contig1\_All, CL8473.Contig1\_All, Unigene22171\_All, Unigene9339\_All, CL1782.Contig3\_All, CL4413.Contig1\_All, Unigene55017\_All, Unigene56188\_All, Unigene55213\_All, Unigene29291\_All, Unigene22212\_All, CL11275.Contig2\_All, CL26.Contig3\_All, CL10701.Contig1\_All, CL7042.Contig2\_All, Unigene19811\_All, CL3284.Contig4\_All, CL7336.Contig4\_All, CL1071.Contig2\_All, CL1026.Contig1\_All, CL6052.Contig1\_All, CL3269.Contig2\_All, Unigene29818\_All, Unigene32311\_All, CL8252.Contig1\_All, CL13850.Contig1\_All, Unigene10448\_All, CL784.Contig4\_All, CL11478.Contig1\_All, CL12585.Contig2\_All, Unigene32180\_All, CL12385.Contig2\_All, CL3173.Contig3\_All, Unigene19008\_All, CL10794.Contig2\_All, CL7636.Contig2\_All, CL12065.Contig1\_All, CL10844.Contig3\_All, CL7636.Contig1\_All, Unigene16117\_All, Unigene22803\_All, CL386.Contig3\_All, CL2079.Contig2\_All, CL3875.Contig1\_All, CL13295.Contig2\_All, CL9503.Contig1\_All, CL10853.Contig1\_All, Unigene35629\_All, CL9779.Contig1\_All, CL5922.Contig1\_All, CL80.Contig1\_All, Unigene43063\_All, CL114.Contig2\_All, Unigene58710\_All, Unigene42825\_All, CL6519.Contig2\_All, CL1911.Contig7\_All, Unigene805\_All, Unigene42356\_All, Unigene58861\_All, Unigene25790\_All, CL6398.Contig2\_All, CL10794.Contig1\_All, Unigene54729\_All, CL6406.Contig2\_All, Unigene64332\_All, CL4420.Contig1\_All, Unigene60364\_All, Unigene8052\_All, CL4068.Contig2\_All, CL12437.Contig2\_All, CL2163.Contig2\_All, CL4794.Contig1\_All, Unigene2805\_All, CL1230.Contig4\_All, Unigene32983\_All, Unigene28775\_All, CL4087.Contig2\_All, CL7514.Contig1\_All, CL9858.Contig4\_All, Unigene40509\_All, CL5206.Contig1\_All, CL90.Contig2\_All, CL8314.Contig2\_All, Unigene11830\_All, Unigene22417\_All, CL3269.Contig1\_All, Unigene4672\_All, CL1249.Contig2\_All, Unigene41968\_All, CL1771.Contig3\_All, CL13437.Contig3\_All, CL10514.Contig2\_All, Unigene22337\_All, Unigene2030\_All, Unigene17552\_All, Unigene40018\_All, CL3191.Contig2\_All, CL4364.Contig2\_All, CL7336.Contig1\_All, Unigene19364\_All, CL1911.Contig6\_All, CL10453.Contig1\_All, Unigene22846\_All, CL1449.Contig1\_All, Unigene23138\_All, CL12218.Contig2\_All, CL7704.Contig1\_All, Unigene60032\_All, Unigene62562\_All, CL644.Contig3\_All, CL10604.Contig3\_All, CL3979.Contig3\_All, Unigene39022\_All, Unigene13280\_All, CL9824.Contig2\_All, CL5794.Contig3\_All, CL11592.Contig2\_All, Unigene56615\_All, Unigene2737\_All, CL1652.Contig2\_All, Unigene10138\_All, CL1849.Contig1\_All, CL7336.Contig2\_All, Unigene25956\_All, Unigene55757\_All, CL1461.Contig3\_All, Unigene56776\_All, CL5052.Contig1\_All, Unigene44184\_All, CL3698.Contig3\_All, CL9738.Contig1\_All, Unigene36244\_All, Unigene34889\_All, CL7461.Contig2\_All, Unigene25614\_All, Unigene15487\_All, CL1461.Contig2\_All, Unigene42502\_All, Unigene2757\_All, Unigene22228\_All, Unigene29415\_All, Unigene18885\_All, Unigene15951\_All, CL18.Contig4\_All, Unigene7840\_All, CL3305.Contig1\_All, CL5863.Contig3\_All, Unigene15744\_All, CL9204.Contig4\_All, Unigene11284\_All, CL11673.Contig1\_All, Unigene29658\_All, CL8531.Contig1\_All, CL12583.Contig1\_All, Unigene39461\_All, CL2222.Contig2\_All, CL4230.Contig1\_All, CL3295.Contig4\_All, Unigene55113\_All, CL1911.Contig9\_All, CL12894.Contig1\_All, CL644.Contig4\_All, Unigene6000\_All, CL6519.Contig1\_All, CL14004.Contig2\_All, Unigene29178\_All, CL2468.Contig2\_All, Unigene16256\_All, Unigene62163\_All, CL11610.Contig1\_All, CL1.Contig7\_All, CL4492.Contig2\_All, CL8081.Contig2\_All, Unigene57337\_All, Unigene8237\_All, Unigene1390\_All, CL4069.Contig2\_All, CL780.Contig2\_All, Unigene36053\_All, CL7262.Contig1\_All, Unigene3111\_All, CL3583.Contig1\_All, Unigene34596\_All, CL4701.Contig1\_All, CL10098.Contig1\_All, CL6815.Contig2\_All, CL3269.Contig3\_All, Unigene2577\_All, CL1339.Contig4\_All, CL5988.Contig3\_All, CL10884.Contig2\_All, Unigene36628\_All, CL10105.Contig1\_All, Unigene38556\_All, Unigene55668\_All, Unigene62007\_All, Unigene63999\_All, CL9351.Contig2\_All, Unigene28819\_All, CL1911.Contig8\_All, Unigene13493\_All, CL9050.Contig1\_All, Unigene34358\_All, CL13403.Contig2\_All, Unigene23980\_All, Unigene2225\_All, Unigene2850\_All, Unigene55797\_All, CL9141.Contig1\_All, Unigene39954\_All, CL9193.Contig1\_All, CL12178.Contig1\_All, CL367.Contig1\_All, Unigene46996\_All, Unigene32598\_All, Unigene23149\_All, CL4661.Contig1\_All, CL1911.Contig10\_All, CL2339.Contig4\_All, Unigene2295\_All, CL7444.Contig1\_All, Unigene41232\_All, Unigene60594\_All, CL3583.Contig2\_All, Unigene60249\_All, CL3880.Contig2\_All, CL12643.Contig2\_All, CL565.Contig2\_All, CL4978.Contig2\_All, CL4069.Contig1\_All, Unigene15946\_All, Unigene7670\_All, Unigene24438\_All, Unigene62219\_All, CL7997.Contig1\_All, Unigene23259\_All, Unigene12825\_All, CL13299.Contig4\_All, CL2163.Contig1\_All, Unigene36121\_All, CL1782.Contig5\_All, Unigene55877\_All, CL3804.Contig2\_All, CL9050.Contig2\_All, CL4226.Contig2\_All, CL1948.Contig3\_All, CL1230.Contig2\_All, CL7073.Contig1\_All, Unigene19846\_All, Unigene25491\_All, Unigene31948\_All, Unigene61202\_All, CL12820.Contig1\_All, CL5945.Contig4\_All, Unigene39218\_All, Unigene41921\_All, CL854.Contig4\_All, Unigene42188\_All, Unigene16516\_All, CL11693.Contig3\_All, Unigene10707\_All, Unigene24550\_All, CL644.Contig1\_All, Unigene31212\_All, CL2339.Contig3\_All, Unigene22234\_All, CL7725.Contig2\_All, Unigene3774\_All, CL4909.Contig2\_All, Unigene45573\_All, Unigene56322\_All, CL10934.Contig2\_All, Unigene60318\_All, CL2048.Contig3\_All, Unigene42446\_All, Unigene16309\_All, Unigene22291\_All, CL2048.Contig2\_All, CL7996.Contig3\_All |
| L-ascorbate oxidase activity | CL3277.Contig3\_All, Unigene41171\_All, CL6808.Contig3\_All, Unigene2988\_All, CL9898.Contig2\_All, CL4447.Contig3\_All, CL9898.Contig3\_All, Unigene17790\_All, Unigene60844\_All, Unigene26248\_All, CL9898.Contig1\_All, Unigene60699\_All, CL9937.Contig3\_All, CL9937.Contig5\_All, CL4447.Contig2\_All, CL9830.Contig1\_All, Unigene3468\_All, CL9937.Contig1\_All, Unigene55217\_All, Unigene20743\_All, Unigene25507\_All, CL7892.Contig3\_All, Unigene13148\_All, CL4447.Contig1\_All, CL13573.Contig2\_All, CL6808.Contig2\_All, CL3277.Contig2\_All, CL3277.Contig1\_All, CL9937.Contig2\_All, CL7892.Contig4\_All, Unigene25851\_All, Unigene2134\_All, CL6808.Contig4\_All, CL13312.Contig1\_All, Unigene6945\_All, Unigene32232\_All, CL6808.Contig5\_All, CL9830.Contig3\_All, Unigene8225\_All, CL13573.Contig1\_All, CL9830.Contig2\_All |
| oxidoreductase activity, acting on diphenols and related substances as donors, oxygen as acceptor | CL3277.Contig3\_All, Unigene41171\_All, CL6808.Contig3\_All, Unigene2988\_All, CL9898.Contig2\_All, CL4447.Contig3\_All, CL9898.Contig3\_All, Unigene17790\_All, Unigene60844\_All, Unigene26248\_All, CL9898.Contig1\_All, Unigene60699\_All, CL9937.Contig3\_All, CL9937.Contig5\_All, CL4447.Contig2\_All, CL9830.Contig1\_All, Unigene3468\_All, CL9937.Contig1\_All, Unigene55217\_All, Unigene20743\_All, Unigene25507\_All, CL7892.Contig3\_All, Unigene13148\_All, CL8866.Contig1\_All, CL4447.Contig1\_All, CL13573.Contig2\_All, CL6808.Contig2\_All, CL3277.Contig2\_All, CL3277.Contig1\_All, CL9937.Contig2\_All, CL7892.Contig4\_All, Unigene25851\_All, Unigene2134\_All, CL8866.Contig3\_All, CL6808.Contig4\_All, CL8866.Contig2\_All, CL13312.Contig1\_All, Unigene6945\_All, Unigene32232\_All, CL6808.Contig5\_All, CL9830.Contig3\_All, Unigene8225\_All, CL13573.Contig1\_All, CL9830.Contig2\_All |
| oxidoreductase activity | CL3879.Contig1\_All, Unigene63816\_All, CL5287.Contig1\_All, CL8737.Contig1\_All, CL3930.Contig4\_All, CL10054.Contig2\_All, Unigene10484\_All, CL4943.Contig2\_All, CL1338.Contig1\_All, Unigene20235\_All, Unigene31737\_All, Unigene38900\_All, CL1491.Contig3\_All, CL11925.Contig1\_All, Unigene59054\_All, Unigene42184\_All, Unigene34671\_All, CL10919.Contig1\_All, CL2487.Contig1\_All, Unigene41301\_All, Unigene502\_All, CL13232.Contig2\_All, Unigene46084\_All, CL6502.Contig1\_All, Unigene25297\_All, CL9772.Contig1\_All, CL7843.Contig3\_All, CL11594.Contig1\_All, Unigene17633\_All, Unigene2293\_All, CL258.Contig2\_All, Unigene8100\_All, CL12637.Contig2\_All, CL7517.Contig2\_All, CL2139.Contig5\_All, CL11011.Contig3\_All, CL51.Contig2\_All, Unigene2988\_All, CL9898.Contig2\_All, CL12922.Contig2\_All, Unigene41646\_All, Unigene3321\_All, CL10515.Contig2\_All, CL1431.Contig2\_All, Unigene56094\_All, CL1808.Contig2\_All, Unigene24849\_All, Unigene13422\_All, CL11644.Contig1\_All, CL12376.Contig2\_All, Unigene20743\_All, Unigene34452\_All, Unigene26129\_All, CL3610.Contig2\_All, CL8001.Contig1\_All, CL10427.Contig4\_All, Unigene12103\_All, CL3277.Contig1\_All, CL8389.Contig3\_All, CL6289.Contig1\_All, Unigene33964\_All, Unigene220\_All, Unigene55188\_All, Unigene24554\_All, Unigene56096\_All, Unigene42939\_All, Unigene24435\_All, CL211.Contig3\_All, CL12628.Contig2\_All, CL5663.Contig2\_All, CL9830.Contig3\_All, CL7032.Contig2\_All, Unigene60317\_All, CL8737.Contig2\_All, Unigene38927\_All, Unigene912\_All, CL12898.Contig3\_All, Unigene17130\_All, Unigene7919\_All, CL6415.Contig2\_All, CL6201.Contig4\_All, CL2087.Contig1\_All, CL5527.Contig1\_All, CL196.Contig1\_All, Unigene57948\_All, Unigene10269\_All, Unigene59012\_All, Unigene54412\_All, Unigene42473\_All, CL1195.Contig2\_All, Unigene60007\_All, CL1575.Contig2\_All, CL98.Contig9\_All, CL11418.Contig1\_All, CL3038.Contig1\_All, Unigene3652\_All, Unigene62497\_All, CL1491.Contig2\_All, CL1425.Contig2\_All, CL1207.Contig3\_All, CL1395.Contig2\_All, CL2330.Contig4\_All, Unigene58317\_All, Unigene33233\_All, Unigene4665\_All, Unigene20852\_All, Unigene28560\_All, CL6441.Contig1\_All, Unigene6066\_All, Unigene16566\_All, CL7649.Contig1\_All, CL7181.Contig2\_All, CL2587.Contig1\_All, CL4725.Contig2\_All, CL1203.Contig4\_All, Unigene39970\_All, CL8835.Contig2\_All, CL11392.Contig2\_All, CL3991.Contig2\_All, Unigene42928\_All, Unigene3469\_All, CL9898.Contig3\_All, Unigene33962\_All, CL7677.Contig2\_All, CL6526.Contig2\_All, Unigene3226\_All, Unigene29581\_All, CL2077.Contig2\_All, CL8446.Contig2\_All, CL11742.Contig1\_All, CL1808.Contig1\_All, Unigene2266\_All, CL9787.Contig1\_All, Unigene59549\_All, CL13433.Contig1\_All, CL7371.Contig1\_All, CL9892.Contig3\_All, CL11002.Contig1\_All, CL8045.Contig1\_All, Unigene15519\_All, CL109.Contig1\_All, CL8866.Contig3\_All, CL11074.Contig1\_All, CL6810.Contig4\_All, Unigene35224\_All, CL2200.Contig2\_All, Unigene58406\_All, CL620.Contig2\_All, Unigene33961\_All, Unigene15957\_All, CL7109.Contig3\_All, Unigene57168\_All, Unigene27302\_All, Unigene23765\_All, CL2225.Contig1\_All, CL11361.Contig2\_All, Unigene3242\_All, Unigene30293\_All, CL1239.Contig2\_All, CL13689.Contig3\_All, CL3596.Contig2\_All, CL8132.Contig1\_All, Unigene15306\_All, Unigene27052\_All, CL6791.Contig4\_All, Unigene24018\_All, Unigene19782\_All, CL4781.Contig2\_All, CL5167.Contig5\_All, CL173.Contig17\_All, CL12933.Contig1\_All, Unigene57201\_All, Unigene45080\_All, Unigene59858\_All, CL173.Contig44\_All, Unigene63349\_All, CL6526.Contig1\_All, Unigene25738\_All, Unigene23799\_All, CL173.Contig35\_All, Unigene41308\_All, Unigene55841\_All, CL8389.Contig2\_All, Unigene12972\_All, Unigene1238\_All, CL2814.Contig6\_All, CL1779.Contig5\_All, Unigene39526\_All, Unigene39204\_All, CL13573.Contig1\_All, CL9830.Contig2\_All, Unigene3061\_All, Unigene9422\_All, Unigene60002\_All, CL98.Contig1\_All, CL12577.Contig2\_All, Unigene4039\_All, Unigene42426\_All, CL11074.Contig2\_All, Unigene56676\_All, Unigene47014\_All, CL3807.Contig3\_All, CL9898.Contig1\_All, CL9651.Contig2\_All, Unigene30252\_All, Unigene6988\_All, Unigene40247\_All, Unigene56098\_All, Unigene5760\_All, CL8380.Contig3\_All, CL3405.Contig5\_All, CL5273.Contig1\_All, Unigene55451\_All, Unigene40499\_All, CL13997.Contig3\_All, Unigene61025\_All, CL4982.Contig2\_All, Unigene10603\_All, CL5178.Contig2\_All, CL331.Contig2\_All, Unigene39649\_All, CL5747.Contig1\_All, Unigene54876\_All, CL6573.Contig1\_All, Unigene35905\_All, CL12933.Contig2\_All, Unigene33138\_All, Unigene13776\_All, Unigene35095\_All, CL3194.Contig1\_All, Unigene38128\_All, CL1416.Contig1\_All, CL6808.Contig5\_All, CL109.Contig5\_All, Unigene57130\_All, Unigene43076\_All, CL14008.Contig2\_All, Unigene9305\_All, CL1134.Contig1\_All, Unigene42744\_All, CL1232.Contig10\_All, Unigene29846\_All, Unigene37432\_All, Unigene26248\_All, Unigene54807\_All, Unigene12174\_All, CL3566.Contig1\_All, CL10875.Contig1\_All, CL3894.Contig3\_All, CL6730.Contig2\_All, CL2040.Contig1\_All, CL2176.Contig3\_All, Unigene9763\_All, Unigene58821\_All, CL12942.Contig1\_All, Unigene3737\_All, CL5672.Contig1\_All, CL983.Contig1\_All, Unigene23051\_All, CL9651.Contig3\_All, Unigene17028\_All, Unigene46903\_All, CL9572.Contig2\_All, Unigene7449\_All, Unigene57038\_All, Unigene60226\_All, Unigene63707\_All, Unigene60681\_All, CL8497.Contig1\_All, Unigene47694\_All, CL1288.Contig4\_All, Unigene35908\_All, Unigene37291\_All, Unigene17327\_All, Unigene40521\_All, CL7218.Contig3\_All, Unigene7531\_All, Unigene56026\_All, CL7818.Contig6\_All, Unigene58323\_All, Unigene31179\_All, Unigene40537\_All, CL1232.Contig6\_All, CL3593.Contig2\_All, Unigene7288\_All, Unigene55021\_All, CL923.Contig3\_All, Unigene28458\_All, CL238.Contig47\_All, CL7100.Contig2\_All, CL6808.Contig2\_All, CL173.Contig11\_All, CL2596.Contig2\_All, CL2710.Contig1\_All, CL3296.Contig1\_All, CL3930.Contig5\_All, CL8523.Contig1\_All, Unigene63784\_All, CL12466.Contig2\_All, CL2003.Contig6\_All, Unigene53362\_All, Unigene36124\_All, Unigene17424\_All, CL11644.Contig2\_All, Unigene21226\_All, CL51.Contig10\_All, Unigene13945\_All, Unigene24303\_All, Unigene58426\_All, CL7484.Contig2\_All, Unigene39020\_All, CL7825.Contig1\_All, CL6062.Contig1\_All, CL10115.Contig1\_All, Unigene8722\_All, Unigene18571\_All, CL6119.Contig1\_All, CL5138.Contig4\_All, CL1239.Contig1\_All, Unigene23174\_All, CL5511.Contig4\_All, CL10185.Contig1\_All, CL5296.Contig5\_All, Unigene35573\_All, CL7390.Contig1\_All, CL7004.Contig2\_All, CL2912.Contig1\_All, CL6808.Contig4\_All, CL1283.Contig4\_All, Unigene56958\_All, CL11453.Contig2\_All, CL6022.Contig2\_All, Unigene14707\_All, CL6022.Contig4\_All, CL6579.Contig2\_All, CL1776.Contig3\_All, CL8682.Contig1\_All, Unigene60699\_All, CL9937.Contig5\_All, CL9766.Contig4\_All, Unigene54422\_All, CL5309.Contig1\_All, CL2120.Contig4\_All, CL3875.Contig1\_All, CL6775.Contig2\_All, CL2814.Contig3\_All, CL581.Contig3\_All, Unigene58124\_All, Unigene61811\_All, Unigene54456\_All, Unigene33156\_All, CL288.Contig1\_All, Unigene36489\_All, Unigene59773\_All, CL1752.Contig1\_All, CL1310.Contig3\_All, CL8237.Contig2\_All, Unigene14151\_All, CL3764.Contig2\_All, Unigene41299\_All, Unigene32718\_All, Unigene18781\_All, CL7712.Contig2\_All, CL10120.Contig1\_All, CL2502.Contig3\_All, CL2176.Contig4\_All, Unigene63439\_All, CL6117.Contig2\_All, Unigene12095\_All, Unigene26932\_All, Unigene16753\_All, CL7100.Contig3\_All, CL8195.Contig2\_All, Unigene2390\_All, CL9830.Contig1\_All, CL9937.Contig1\_All, CL5500.Contig2\_All, Unigene15971\_All, Unigene12487\_All, Unigene29237\_All, CL683.Contig2\_All, Unigene36370\_All, Unigene7046\_All, Unigene18990\_All, Unigene34162\_All, CL14006.Contig1\_All, Unigene30804\_All, Unigene23512\_All, Unigene7124\_All, CL891.Contig6\_All, CL13437.Contig3\_All, Unigene32232\_All, Unigene32711\_All, Unigene37180\_All, CL4483.Contig3\_All, CL1690.Contig1\_All, Unigene57585\_All, Unigene34986\_All, Unigene41171\_All, CL98.Contig12\_All, CL861.Contig2\_All, Unigene45248\_All, CL7743.Contig3\_All, CL10998.Contig1\_All, Unigene26998\_All, Unigene41323\_All, CL1107.Contig1\_All, Unigene23606\_All, Unigene38732\_All, CL4447.Contig2\_All, CL1347.Contig3\_All, CL11028.Contig1\_All, CL683.Contig3\_All, Unigene26734\_All, CL11450.Contig1\_All, Unigene38504\_All, Unigene21627\_All, Unigene30566\_All, CL11002.Contig2\_All, CL12964.Contig1\_All, CL6791.Contig1\_All, Unigene58618\_All, Unigene5926\_All, CL1016.Contig2\_All, CL13390.Contig2\_All, Unigene54327\_All, CL13312.Contig1\_All, CL4044.Contig1\_All, Unigene3643\_All, Unigene6945\_All, Unigene6483\_All, CL12434.Contig2\_All, Unigene9281\_All, Unigene10721\_All, Unigene20348\_All, CL891.Contig2\_All, CL4084.Contig11\_All, CL11087.Contig2\_All, CL5832.Contig1\_All, Unigene29585\_All, CL4812.Contig3\_All, Unigene40795\_All, Unigene18047\_All, CL13995.Contig1\_All, CL12769.Contig2\_All, CL8814.Contig4\_All, Unigene40786\_All, CL5008.Contig1\_All, CL2378.Contig1\_All, CL5311.Contig1\_All, Unigene13148\_All, CL4609.Contig3\_All, CL8866.Contig1\_All, CL9128.Contig1\_All, CL2814.Contig4\_All, Unigene17142\_All, CL12503.Contig2\_All, Unigene61476\_All, CL1053.Contig2\_All, CL3610.Contig5\_All, Unigene12122\_All, Unigene54863\_All, Unigene8225\_All, CL609.Contig49\_All, Unigene57674\_All, CL2077.Contig1\_All, CL109.Contig7\_All, CL9152.Contig2\_All, CL7298.Contig2\_All, Unigene41207\_All, Unigene14349\_All, CL12729.Contig1\_All, CL8799.Contig2\_All, CL11777.Contig1\_All, CL7512.Contig1\_All, CL361.Contig5\_All, Unigene39790\_All, Unigene55815\_All, CL11865.Contig1\_All, Unigene57260\_All, CL6289.Contig2\_All, CL8019.Contig1\_All, Unigene32386\_All, Unigene65641\_All, CL98.Contig6\_All, Unigene41797\_All, Unigene23511\_All, Unigene25662\_All, CL2537.Contig2\_All, Unigene60207\_All, CL8073.Contig1\_All, Unigene13446\_All, CL5527.Contig4\_All, CL2702.Contig4\_All, CL1455.Contig2\_All, Unigene20331\_All, CL2179.Contig3\_All, CL11094.Contig2\_All, Unigene29872\_All, Unigene33089\_All, CL9538.Contig1\_All, CL5875.Contig1\_All, Unigene10549\_All, Unigene35704\_All, CL3527.Contig1\_All, Unigene63708\_All, Unigene702\_All, Unigene61043\_All, CL4067.Contig3\_All, Unigene32479\_All, Unigene6721\_All, CL6201.Contig3\_All, Unigene26365\_All, CL7181.Contig5\_All, CL9390.Contig1\_All, Unigene4069\_All, Unigene50040\_All, CL609.Contig45\_All, CL609.Contig50\_All, Unigene39534\_All, Unigene19331\_All, CL6487.Contig3\_All, Unigene54710\_All, Unigene24912\_All, Unigene4289\_All, Unigene56993\_All, Unigene51140\_All, CL6496.Contig1\_All, CL4483.Contig1\_All, Unigene6476\_All, Unigene42793\_All, CL8472.Contig3\_All, Unigene22442\_All, CL1001.Contig2\_All, Unigene29002\_All, Unigene29920\_All, CL3827.Contig2\_All, CL7892.Contig4\_All, CL10926.Contig2\_All, Unigene43937\_All, Unigene59694\_All, CL831.Contig6\_All, Unigene1929\_All, CL7818.Contig5\_All, Unigene21948\_All, CL1895.Contig15\_All, Unigene18611\_All, CL479.Contig2\_All, CL5146.Contig4\_All, Unigene30012\_All, CL9572.Contig1\_All, CL1814.Contig4\_All, CL7804.Contig1\_All, CL10399.Contig2\_All, CL1283.Contig5\_All, Unigene33618\_All, Unigene41493\_All, CL9494.Contig2\_All, Unigene54427\_All, CL1895.Contig13\_All, CL1491.Contig1\_All, Unigene24004\_All, Unigene57352\_All, CL10466.Contig1\_All, CL3719.Contig12\_All, CL2024.Contig3\_All, CL11965.Contig4\_All, CL891.Contig1\_All, Unigene42966\_All, Unigene43661\_All, Unigene56024\_All, Unigene34694\_All, Unigene17182\_All, Unigene41591\_All, Unigene27064\_All, CL2228.Contig5\_All, CL9639.Contig2\_All, CL5167.Contig1\_All, CL6201.Contig2\_All, CL649.Contig1\_All, CL9328.Contig1\_All, Unigene15719\_All, CL11077.Contig1\_All, Unigene29349\_All, CL1016.Contig1\_All, CL3078.Contig1\_All, CL1431.Contig1\_All, Unigene14805\_All, Unigene39960\_All, CL9578.Contig2\_All, CL847.Contig1\_All, CL3333.Contig2\_All, CL3764.Contig1\_All, CL949.Contig3\_All, CL1195.Contig3\_All, Unigene55939\_All, CL5567.Contig2\_All, CL3333.Contig4\_All, CL6791.Contig2\_All, Unigene44382\_All, CL6119.Contig2\_All, CL3593.Contig3\_All, Unigene55739\_All, CL7447.Contig1\_All, CL12452.Contig2\_All, CL3930.Contig3\_All, CL6808.Contig3\_All, Unigene15416\_All, CL109.Contig6\_All, Unigene4846\_All, Unigene56665\_All, CL1045.Contig1\_All, CL7061.Contig3\_All, CL10054.Contig1\_All, CL8493.Contig4\_All, CL4773.Contig2\_All, CL8118.Contig2\_All, Unigene25507\_All, Unigene59793\_All, CL11731.Contig1\_All, Unigene29884\_All, Unigene27257\_All, Unigene4144\_All, CL9639.Contig1\_All, Unigene49975\_All, Unigene3851\_All, CL5082.Contig1\_All, Unigene55119\_All, Unigene54321\_All, Unigene15535\_All, CL2032.Contig5\_All, CL10185.Contig2\_All, Unigene25365\_All, CL2777.Contig1\_All, Unigene8763\_All, Unigene59471\_All, CL23.Contig1\_All, CL51.Contig1\_All, CL7004.Contig1\_All, CL5527.Contig6\_All, CL10029.Contig1\_All, Unigene33885\_All, Unigene17790\_All, Unigene57584\_All, Unigene60844\_All, CL238.Contig33\_All, Unigene57139\_All, CL1310.Contig2\_All, Unigene55745\_All, CL1442.Contig5\_All, Unigene3383\_All, CL3273.Contig1\_All, Unigene5483\_All, Unigene59048\_All, CL2814.Contig5\_All, Unigene3614\_All, CL2032.Contig2\_All, CL5287.Contig2\_All, CL11715.Contig4\_All, CL3719.Contig11\_All, Unigene40545\_All, Unigene2034\_All, CL2032.Contig7\_All, Unigene55746\_All, Unigene58117\_All, CL631.Contig2\_All, CL9528.Contig1\_All, CL5008.Contig3\_All, CL10595.Contig3\_All, CL1776.Contig1\_All, Unigene59572\_All, Unigene59106\_All, Unigene65156\_All, CL891.Contig8\_All, CL7181.Contig4\_All, CL13232.Contig1\_All, CL3332.Contig1\_All, CL7061.Contig4\_All, Unigene29003\_All, CL3261.Contig4\_All, CL8464.Contig1\_All, CL815.Contig2\_All, Unigene60254\_All, Unigene40267\_All, Unigene27402\_All, Unigene4398\_All, CL1923.Contig3\_All, CL6415.Contig3\_All, CL13893.Contig1\_All, CL7371.Contig2\_All, CL923.Contig4\_All, CL2710.Contig2\_All, Unigene20050\_All, Unigene4783\_All, CL10719.Contig1\_All, Unigene27006\_All, Unigene41649\_All, CL2596.Contig1\_All, CL2702.Contig2\_All, CL4209.Contig1\_All, CL4791.Contig2\_All, Unigene20078\_All, Unigene62583\_All, CL5672.Contig2\_All, Unigene40600\_All, CL6062.Contig2\_All, CL11535.Contig3\_All, Unigene11881\_All, Unigene20151\_All, Unigene33791\_All, Unigene1034\_All, Unigene23747\_All, Unigene39069\_All, CL7677.Contig1\_All, Unigene14776\_All, CL2233.Contig3\_All, Unigene13893\_All, CL3524.Contig1\_All, Unigene105\_All, CL3930.Contig1\_All, Unigene11358\_All, CL13821.Contig1\_All, CL11169.Contig1\_All, CL2024.Contig2\_All, CL12184.Contig1\_All, CL11715.Contig2\_All, Unigene27357\_All, Unigene33950\_All, CL479.Contig5\_All, CL3780.Contig2\_All, CL7457.Contig2\_All, CL4994.Contig9\_All, CL6005.Contig1\_All, Unigene26130\_All, CL8118.Contig1\_All, CL4812.Contig5\_All, Unigene54419\_All, CL9395.Contig1\_All, CL7072.Contig2\_All, CL11051.Contig2\_All, CL7990.Contig1\_All, CL1375.Contig1\_All, CL6555.Contig7\_All, Unigene6374\_All, CL2825.Contig1\_All, Unigene57305\_All, Unigene56231\_All, CL1232.Contig14\_All, CL831.Contig3\_All, Unigene56577\_All, Unigene58425\_All, CL13490.Contig1\_All, Unigene286\_All, CL3277.Contig3\_All, CL1053.Contig1\_All, Unigene28096\_All, CL109.Contig4\_All, Unigene27428\_All, Unigene26344\_All, CL9706.Contig7\_All, CL5672.Contig3\_All, CL12628.Contig1\_All, Unigene38008\_All, Unigene56432\_All, Unigene12613\_All, Unigene42971\_All, CL1814.Contig3\_All, CL1395.Contig1\_All, CL7310.Contig1\_All, Unigene29194\_All, Unigene56805\_All, Unigene54999\_All, Unigene6952\_All, CL2032.Contig4\_All, CL11300.Contig2\_All, CL12466.Contig4\_All, CL10551.Contig1\_All, Unigene21243\_All, Unigene21981\_All, Unigene59047\_All, CL6136.Contig2\_All, CL10040.Contig2\_All, CL12668.Contig1\_All, Unigene4376\_All, CL9973.Contig3\_All, Unigene55840\_All, Unigene36019\_All, CL10396.Contig1\_All, Unigene20850\_All, CL11627.Contig2\_All, CL6804.Contig2\_All, CL1726.Contig2\_All, Unigene16517\_All, CL13997.Contig1\_All, Unigene15457\_All, Unigene10055\_All, CL3744.Contig2\_All, Unigene42390\_All, CL4447.Contig3\_All, CL12720.Contig2\_All, CL5185.Contig2\_All, CL12810.Contig1\_All, Unigene4105\_All, CL1310.Contig1\_All, CL2225.Contig2\_All, CL4791.Contig1\_All, Unigene589\_All, CL7512.Contig2\_All, Unigene15134\_All, CL5178.Contig6\_All, Unigene26267\_All, CL1779.Contig2\_All, CL3507.Contig4\_All, Unigene9046\_All, CL4782.Contig2\_All, Unigene28755\_All, Unigene10725\_All, Unigene56997\_All, Unigene60014\_All, Unigene20125\_All, CL772.Contig2\_All, Unigene3685\_All, CL11627.Contig3\_All, CL9625.Contig2\_All, CL7712.Contig5\_All, Unigene15289\_All, Unigene62417\_All, Unigene15415\_All, Unigene3732\_All, Unigene38507\_All, CL5025.Contig3\_All, CL5870.Contig1\_All, CL2330.Contig2\_All, Unigene55161\_All, Unigene1557\_All, CL11417.Contig2\_All, Unigene59586\_All, CL9645.Contig1\_All, CL9937.Contig3\_All, Unigene61438\_All, Unigene40378\_All, Unigene11831\_All, Unigene20279\_All, Unigene13603\_All, CL2260.Contig2\_All, CL1997.Contig2\_All, Unigene30020\_All, Unigene55335\_All, Unigene38651\_All, CL3443.Contig2\_All, CL6201.Contig1\_All, Unigene59421\_All, Unigene54413\_All, CL5870.Contig2\_All, CL3277.Contig2\_All, CL238.Contig42\_All, CL3283.Contig3\_All, Unigene25851\_All, CL10971.Contig2\_All, CL11392.Contig1\_All, Unigene33749\_All, CL8258.Contig2\_All, CL1698.Contig1\_All, Unigene61369\_All, CL12392.Contig1\_All, Unigene51332\_All, CL2032.Contig1\_All, CL13689.Contig2\_All, CL173.Contig24\_All, CL3610.Contig7\_All, Unigene21330\_All, CL11688.Contig1\_All, Unigene3468\_All, CL4533.Contig3\_All, CL4405.Contig4\_All, CL6304.Contig2\_All, Unigene25845\_All, Unigene30126\_All, Unigene59478\_All, CL6865.Contig1\_All, Unigene38690\_All, CL949.Contig1\_All, CL2757.Contig3\_All, Unigene22601\_All, CL3527.Contig2\_All, CL1575.Contig1\_All, CL238.Contig57\_All, CL9766.Contig3\_All, CL5178.Contig5\_All, Unigene26619\_All, Unigene48153\_All, Unigene18514\_All, CL7971.Contig2\_All, CL11167.Contig1\_All, Unigene56097\_All, CL9327.Contig1\_All, CL4656.Contig1\_All, Unigene56835\_All, CL10650.Contig1\_All, Unigene64438\_All, Unigene10664\_All, Unigene31739\_All, Unigene1913\_All, Unigene43534\_All, CL5296.Contig1\_All, CL10267.Contig1\_All, CL5138.Contig1\_All, CL8234.Contig1\_All, CL9751.Contig2\_All, Unigene54418\_All, Unigene62552\_All, CL831.Contig2\_All, Unigene28145\_All, CL9711.Contig3\_All, CL7715.Contig3\_All, CL10551.Contig3\_All, Unigene14894\_All, CL1232.Contig8\_All, CL7892.Contig3\_All, Unigene42611\_All, Unigene58324\_All, Unigene55459\_All, Unigene58669\_All, CL1986.Contig2\_All, CL194.Contig3\_All, Unigene64694\_All, CL5146.Contig3\_All, CL2372.Contig1\_All, Unigene46513\_All, CL581.Contig1\_All, CL1895.Contig14\_All, CL3574.Contig5\_All, CL1817.Contig1\_All, CL7993.Contig1\_All, Unigene35075\_All, CL6775.Contig1\_All, Unigene4205\_All, CL603.Contig5\_All, Unigene12455\_All, Unigene32418\_All, CL6434.Contig4\_All, Unigene58783\_All, CL98.Contig18\_All, CL9258.Contig1\_All, Unigene14655\_All, CL10179.Contig1\_All, CL109.Contig2\_All, Unigene42870\_All, CL10224.Contig1\_All, CL7377.Contig2\_All, Unigene43171\_All, CL12972.Contig1\_All, CL12717.Contig1\_All, Unigene976\_All, CL5723.Contig3\_All, CL9400.Contig2\_All, Unigene36282\_All, CL8835.Contig1\_All, CL5397.Contig3\_All, CL10595.Contig1\_All, Unigene60039\_All, CL4443.Contig2\_All, CL2711.Contig1\_All, CL238.Contig39\_All, CL12434.Contig3\_All, CL8866.Contig2\_All, CL5537.Contig1\_All, Unigene14167\_All, CL7512.Contig3\_All, Unigene4557\_All, CL2588.Contig1\_All, CL3333.Contig1\_All, CL6903.Contig2\_All, Unigene58820\_All, Unigene56095\_All, Unigene11976\_All, Unigene46995\_All, CL13309.Contig1\_All, Unigene20086\_All, CL8389.Contig1\_All, Unigene10514\_All, Unigene38704\_All, Unigene62303\_All, Unigene58150\_All, CL12769.Contig1\_All, Unigene62945\_All, CL6117.Contig1\_All, CL13976.Contig1\_All, Unigene41361\_All, CL9538.Contig2\_All, Unigene57963\_All, CL6791.Contig5\_All, CL3925.Contig3\_All, CL238.Contig40\_All, CL5537.Contig2\_All, Unigene23920\_All, Unigene30105\_All, Unigene21419\_All, CL5823.Contig1\_All, Unigene18527\_All, CL11777.Contig2\_All, Unigene61740\_All, Unigene30214\_All, CL5818.Contig3\_All, Unigene12838\_All, CL361.Contig1\_All, Unigene13680\_All, CL1589.Contig8\_All, CL9651.Contig1\_All, Unigene63665\_All, CL7712.Contig1\_All, Unigene7551\_All, Unigene654\_All, Unigene34759\_All, CL911.Contig1\_All, CL6730.Contig3\_All, CL10551.Contig2\_All, CL2221.Contig4\_All, CL7971.Contig3\_All, CL7715.Contig1\_All, CL2024.Contig1\_All, CL2588.Contig2\_All, CL4447.Contig1\_All, Unigene42146\_All, Unigene12548\_All, Unigene10156\_All, CL8313.Contig2\_All, Unigene40276\_All, Unigene58202\_All, CL6177.Contig2\_All, Unigene4590\_All, CL4168.Contig1\_All, Unigene565\_All, CL2233.Contig9\_All, CL737.Contig1\_All, Unigene34388\_All, CL7032.Contig1\_All, CL8682.Contig2\_All, Unigene37444\_All, CL6062.Contig3\_All, Unigene2577\_All, CL1705.Contig2\_All, CL3780.Contig1\_All, Unigene63761\_All, CL10396.Contig2\_All, CL4483.Contig2\_All, Unigene26135\_All, CL11418.Contig2\_All, CL5178.Contig4\_All, CL1491.Contig5\_All, CL1288.Contig3\_All, Unigene7095\_All, Unigene31267\_All, Unigene22107\_All, Unigene16775\_All, Unigene29642\_All, CL10515.Contig1\_All, Unigene9780\_All, Unigene13541\_All, CL3014.Contig2\_All, Unigene29847\_All, CL12394.Contig2\_All, Unigene4339\_All, CL4405.Contig3\_All, CL8799.Contig1\_All, CL7072.Contig1\_All, CL173.Contig14\_All, Unigene17192\_All, Unigene29492\_All, Unigene2134\_All, CL5710.Contig2\_All, CL7719.Contig2\_All, CL1001.Contig3\_All, Unigene4259\_All, Unigene29396\_All, CL3002.Contig2\_All, CL11965.Contig3\_All, Unigene60021\_All, CL6107.Contig2\_All, Unigene21324\_All, CL13963.Contig1\_All, Unigene7376\_All, Unigene44648\_All, Unigene30121\_All, CL6869.Contig3\_All, CL1326.Contig2\_All, CL12376.Contig1\_All, Unigene55407\_All, CL2517.Contig9\_All, Unigene61417\_All, Unigene36907\_All, Unigene20309\_All, CL379.Contig1\_All, Unigene56806\_All, CL12431.Contig1\_All, CL479.Contig4\_All, Unigene59884\_All, Unigene13789\_All, CL8258.Contig1\_All, CL13573.Contig2\_All, CL2495.Contig1\_All, CL603.Contig1\_All, Unigene38510\_All, Unigene7410\_All, CL3894.Contig1\_All, CL3981.Contig2\_All, Unigene20496\_All, CL2067.Contig2\_All, CL4773.Contig1\_All, CL12396.Contig1\_All, CL9128.Contig2\_All, CL1817.Contig5\_All, CL9494.Contig5\_All, Unigene37129\_All, Unigene25097\_All, Unigene41430\_All, CL7520.Contig1\_All, CL4443.Contig1\_All, CL7540.Contig1\_All, CL5178.Contig3\_All, CL2583.Contig1\_All, Unigene55217\_All, Unigene54650\_All, CL5818.Contig2\_All, Unigene60904\_All, Unigene61007\_All, CL7228.Contig3\_All, Unigene30619\_All, CL2523.Contig1\_All, Unigene29888\_All, Unigene19285\_All, Unigene41520\_All, CL9937.Contig2\_All, Unigene60725\_All, CL5311.Contig2\_All, Unigene58449\_All, Unigene55183\_All, Unigene488\_All, Unigene59816\_All, Unigene15776\_All |
| protein heterodimerization activity | Unigene26228\_All, Unigene14232\_All, CL925.Contig1\_All, Unigene7225\_All, Unigene54439\_All, Unigene16906\_All, Unigene58596\_All, Unigene34037\_All, Unigene2140\_All, Unigene32903\_All, CL925.Contig4\_All, Unigene27222\_All, Unigene22502\_All, Unigene54414\_All, Unigene814\_All, CL1993.Contig5\_All, Unigene59365\_All, CL3125.Contig2\_All, Unigene59271\_All, Unigene60054\_All, Unigene59272\_All, CL626.Contig1\_All, CL1993.Contig7\_All, CL3125.Contig1\_All, Unigene26304\_All, CL13928.Contig3\_All, Unigene59413\_All, Unigene7222\_All, Unigene23337\_All, Unigene13015\_All, CL1993.Contig10\_All, CL1229.Contig8\_All, Unigene32033\_All, Unigene11037\_All, CL13928.Contig2\_All, Unigene18068\_All, Unigene7585\_All, CL1229.Contig14\_All, CL1229.Contig3\_All, Unigene39204\_All, CL8242.Contig2\_All, CL6283.Contig1\_All, CL1993.Contig12\_All, CL5699.Contig3\_All, CL6978.Contig1\_All, Unigene27654\_All, Unigene35395\_All, Unigene1471\_All, CL1993.Contig4\_All, CL12409.Contig1\_All, Unigene27205\_All, CL1229.Contig7\_All, Unigene46995\_All, CL1993.Contig3\_All, CL1229.Contig10\_All, CL37.Contig7\_All, CL10546.Contig1\_All, CL5958.Contig1\_All, CL608.Contig1\_All, Unigene54423\_All, CL5750.Contig1\_All, Unigene56286\_All, Unigene20992\_All, CL11028.Contig1\_All, Unigene1479\_All, CL1229.Contig5\_All, Unigene16775\_All, CL1993.Contig6\_All, Unigene18850\_All, CL1993.Contig8\_All, Unigene35022\_All, Unigene5932\_All, CL1229.Contig2\_All, Unigene2267\_All, Unigene18782\_All, CL5268.Contig1\_All, Unigene59412\_All, Unigene25841\_All, CL13755.Contig1\_All, Unigene5212\_All, CL13407.Contig2\_All, CL3960.Contig2\_All, Unigene20722\_All, CL1993.Contig1\_All, CL13407.Contig1\_All, Unigene35457\_All, CL1229.Contig4\_All, CL5750.Contig2\_All, Unigene54415\_All, Unigene18169\_All, CL1993.Contig11\_All, Unigene6889\_All, CL1993.Contig2\_All, Unigene54417\_All, Unigene6536\_All, Unigene40494\_All, CL9125.Contig2\_All, CL1229.Contig6\_All, CL5958.Contig2\_All, Unigene19297\_All, CL13928.Contig1\_All, CL2071.Contig5\_All, CL1993.Contig9\_All |
| structural constituent of cytoskeleton | Unigene41182\_All, Unigene5929\_All, Unigene19302\_All, Unigene14305\_All, CL2964.Contig12\_All, Unigene57776\_All, Unigene56244\_All, Unigene2386\_All, Unigene33359\_All, CL2964.Contig3\_All, Unigene16373\_All, Unigene56276\_All, Unigene33360\_All, Unigene60249\_All, CL13545.Contig1\_All, Unigene56915\_All, Unigene6347\_All, CL7636.Contig2\_All, CL91.Contig6\_All, CL7636.Contig1\_All, Unigene4536\_All, Unigene21196\_All, Unigene12757\_All, Unigene57766\_All, Unigene19828\_All, Unigene7402\_All, CL9574.Contig1\_All, CL9574.Contig3\_All, Unigene10801\_All, Unigene43614\_All, Unigene13534\_All, Unigene56781\_All, Unigene4181\_All, Unigene901\_All, CL91.Contig9\_All, CL12624.Contig1\_All, CL13899.Contig2\_All, Unigene57619\_All, CL11526.Contig1\_All, Unigene11244\_All, Unigene58622\_All, Unigene57765\_All, Unigene41288\_All, CL13899.Contig5\_All, Unigene22244\_All, CL2964.Contig8\_All, Unigene3390\_All, CL12624.Contig3\_All, CL9799.Contig1\_All, Unigene19421\_All, CL13949.Contig1\_All, Unigene16128\_All, CL12606.Contig2\_All, Unigene56245\_All, Unigene3781\_All, CL2964.Contig13\_All, Unigene6780\_All, Unigene33863\_All, Unigene33862\_All, CL8696.Contig1\_All |
| hydroquinone:oxygen oxidoreductase activity | CL4447.Contig1\_All, CL13573.Contig2\_All, CL9898.Contig2\_All, CL4447.Contig3\_All, Unigene17790\_All, CL9898.Contig3\_All, Unigene60844\_All, Unigene26248\_All, CL9898.Contig1\_All, CL7892.Contig4\_All, Unigene60699\_All, CL9937.Contig2\_All, CL9937.Contig3\_All, CL9937.Contig5\_All, Unigene2134\_All, CL4447.Contig2\_All, CL9937.Contig1\_All, Unigene55217\_All, Unigene8225\_All, CL13573.Contig1\_All, CL7892.Contig3\_All |
| oxidoreductase activity, acting on diphenols and related substances as donors | CL3277.Contig3\_All, Unigene41171\_All, CL6808.Contig3\_All, Unigene2988\_All, CL9898.Contig2\_All, CL4447.Contig3\_All, CL9898.Contig3\_All, Unigene17790\_All, Unigene60844\_All, Unigene26248\_All, CL9898.Contig1\_All, Unigene60699\_All, CL9937.Contig3\_All, CL7218.Contig3\_All, CL9937.Contig5\_All, CL4447.Contig2\_All, CL9830.Contig1\_All, Unigene3468\_All, CL9787.Contig1\_All, CL9937.Contig1\_All, Unigene55217\_All, Unigene20743\_All, Unigene25507\_All, CL7892.Contig3\_All, Unigene13148\_All, CL8866.Contig1\_All, CL4447.Contig1\_All, CL13573.Contig2\_All, CL6808.Contig2\_All, CL3277.Contig2\_All, CL3277.Contig1\_All, CL9937.Contig2\_All, CL7892.Contig4\_All, Unigene25851\_All, Unigene2134\_All, CL8866.Contig3\_All, CL6808.Contig4\_All, CL8866.Contig2\_All, CL13312.Contig1\_All, Unigene6945\_All, Unigene32232\_All, CL6808.Contig5\_All, CL7109.Contig3\_All, Unigene8225\_All, CL9830.Contig3\_All, CL13573.Contig1\_All, CL9830.Contig2\_All |
| peroxidase activity | Unigene10721\_All, Unigene13945\_All, Unigene27052\_All, Unigene55407\_All, CL173.Contig24\_All, Unigene654\_All, CL2024.Contig2\_All, CL6791.Contig4\_All, CL3078.Contig1\_All, CL10179.Contig1\_All, Unigene6476\_All, CL2024.Contig1\_All, CL1575.Contig2\_All, CL5138.Contig4\_All, CL173.Contig17\_All, Unigene29237\_All, Unigene12548\_All, CL5511.Contig4\_All, CL10185.Contig1\_All, Unigene62497\_All, Unigene9046\_All, Unigene40276\_All, Unigene29002\_All, Unigene46084\_All, Unigene55939\_All, CL1395.Contig2\_All, CL5397.Contig3\_All, CL173.Contig44\_All, CL9400.Contig2\_All, Unigene55746\_All, CL6791.Contig2\_All, CL1575.Contig1\_All, Unigene23799\_All, CL173.Contig35\_All, CL11167.Contig1\_All, CL3593.Contig3\_All, Unigene32711\_All, CL7181.Contig2\_All, Unigene59106\_All, Unigene15415\_All, Unigene60681\_All, CL8497.Contig1\_All, CL7181.Contig4\_All, Unigene43534\_All, CL5138.Contig1\_All, Unigene11976\_All, Unigene59586\_All, CL7743.Contig3\_All, Unigene56665\_All, CL13309.Contig1\_All, CL7371.Contig2\_All, Unigene10514\_All, CL2537.Contig2\_All, Unigene40537\_All, CL1395.Contig1\_All, CL3593.Contig2\_All, CL11644.Contig1\_All, Unigene22107\_All, Unigene27006\_All, Unigene55335\_All, Unigene40499\_All, CL7371.Contig1\_All, Unigene13541\_All, CL9538.Contig2\_All, CL11535.Contig3\_All, Unigene61811\_All, CL173.Contig11\_All, CL2024.Contig3\_All, CL5082.Contig1\_All, CL173.Contig14\_All, Unigene33089\_All, Unigene58449\_All, CL9538.Contig1\_All, Unigene29492\_All, Unigene64694\_All, CL6791.Contig5\_All, CL6791.Contig1\_All, CL10185.Contig2\_All, Unigene488\_All, Unigene14776\_All, Unigene15957\_All, Unigene15457\_All, Unigene57168\_All, Unigene23765\_All, CL7181.Contig5\_All, CL11644.Contig2\_All |
| oxidoreductase activity, acting on peroxide as acceptor | Unigene10721\_All, Unigene13945\_All, Unigene27052\_All, Unigene55407\_All, CL173.Contig24\_All, Unigene654\_All, CL2024.Contig2\_All, CL6791.Contig4\_All, CL3078.Contig1\_All, CL10179.Contig1\_All, Unigene6476\_All, CL2024.Contig1\_All, CL1575.Contig2\_All, CL5138.Contig4\_All, CL173.Contig17\_All, Unigene29237\_All, Unigene12548\_All, CL5511.Contig4\_All, CL10185.Contig1\_All, Unigene62497\_All, Unigene9046\_All, Unigene40276\_All, Unigene29002\_All, Unigene46084\_All, Unigene55939\_All, CL1395.Contig2\_All, CL5397.Contig3\_All, CL173.Contig44\_All, CL9400.Contig2\_All, Unigene55746\_All, CL6791.Contig2\_All, CL1575.Contig1\_All, Unigene23799\_All, CL173.Contig35\_All, CL11167.Contig1\_All, CL3593.Contig3\_All, Unigene32711\_All, CL7181.Contig2\_All, Unigene59106\_All, Unigene15415\_All, Unigene60681\_All, CL8497.Contig1\_All, CL7181.Contig4\_All, Unigene43534\_All, CL5138.Contig1\_All, Unigene11976\_All, Unigene59586\_All, CL7743.Contig3\_All, Unigene56665\_All, CL13309.Contig1\_All, CL7371.Contig2\_All, Unigene10514\_All, CL2537.Contig2\_All, Unigene40537\_All, CL1395.Contig1\_All, CL3593.Contig2\_All, CL11644.Contig1\_All, Unigene22107\_All, Unigene27006\_All, Unigene55335\_All, Unigene40499\_All, CL7371.Contig1\_All, Unigene13541\_All, CL9538.Contig2\_All, CL11535.Contig3\_All, Unigene61811\_All, CL173.Contig11\_All, CL2024.Contig3\_All, CL5082.Contig1\_All, CL173.Contig14\_All, Unigene33089\_All, Unigene58449\_All, CL9538.Contig1\_All, Unigene29492\_All, Unigene64694\_All, CL6791.Contig5\_All, CL6791.Contig1\_All, CL10185.Contig2\_All, Unigene488\_All, Unigene14776\_All, Unigene15957\_All, Unigene15457\_All, Unigene57168\_All, Unigene23765\_All, CL7181.Contig5\_All, CL11644.Contig2\_All |
| hydrogen-translocating pyrophosphatase activity | CL479.Contig6\_All, Unigene31329\_All, CL6201.Contig1\_All, Unigene32233\_All, Unigene6405\_All, CL479.Contig2\_All, CL2967.Contig2\_All, CL479.Contig1\_All, CL6201.Contig2\_All, CL6201.Contig4\_All, Unigene62513\_All, Unigene2876\_All, CL2967.Contig3\_All, CL479.Contig3\_All, CL1442.Contig5\_All, CL10910.Contig1\_All, CL6201.Contig3\_All, CL1113.Contig2\_All, CL479.Contig4\_All, CL10307.Contig1\_All, CL479.Contig5\_All, Unigene37012\_All |
| GTPase activity | CL896.Contig15\_All, Unigene56323\_All, CL1222.Contig9\_All, Unigene14305\_All, Unigene14583\_All, Unigene33359\_All, Unigene13688\_All, CL65.Contig1\_All, CL896.Contig30\_All, CL1974.Contig3\_All, CL9286.Contig1\_All, CL2782.Contig1\_All, Unigene6347\_All, CL3049.Contig5\_All, Unigene27997\_All, Unigene3973\_All, Unigene56781\_All, Unigene7418\_All, CL12325.Contig1\_All, CL65.Contig2\_All, Unigene11244\_All, Unigene56324\_All, CL12624.Contig3\_All, CL13949.Contig1\_All, Unigene61511\_All, Unigene6773\_All, Unigene7098\_All, CL721.Contig9\_All, CL13973.Contig1\_All, Unigene42382\_All, CL896.Contig18\_All, CL4961.Contig1\_All, Unigene40080\_All, Unigene25646\_All, Unigene42887\_All, Unigene19302\_All, CL8558.Contig1\_All, CL2964.Contig12\_All, CL3019.Contig2\_All, CL896.Contig11\_All, Unigene56244\_All, Unigene2386\_All, CL366.Contig4\_All, Unigene16373\_All, Unigene358\_All, Unigene56915\_All, CL13545.Contig1\_All, CL896.Contig13\_All, CL2504.Contig1\_All, Unigene4536\_All, Unigene21196\_All, CL2782.Contig2\_All, Unigene57766\_All, Unigene59157\_All, Unigene19828\_All, Unigene7402\_All, CL11358.Contig4\_All, Unigene10801\_All, Unigene901\_All, CL1084.Contig3\_All, Unigene13396\_All, Unigene57619\_All, CL3049.Contig1\_All, CL13641.Contig3\_All, Unigene57765\_All, Unigene29969\_All, CL13476.Contig4\_All, Unigene1316\_All, CL9257.Contig5\_All, Unigene34884\_All, Unigene56245\_All, Unigene3781\_All, CL896.Contig19\_All, CL896.Contig33\_All, Unigene33862\_All, Unigene23394\_All, CL8248.Contig7\_All, CL13861.Contig5\_All, CL12697.Contig1\_All, Unigene56196\_All, Unigene44279\_All, CL1084.Contig4\_All, CL896.Contig20\_All, CL4186.Contig2\_All, CL2579.Contig2\_All, Unigene30740\_All, Unigene33360\_All, CL13378.Contig1\_All, CL6985.Contig4\_All, Unigene4349\_All, CL366.Contig1\_All, Unigene12757\_All, CL3019.Contig1\_All, Unigene30179\_All, Unigene13534\_All, CL4324.Contig2\_All, Unigene12291\_All, Unigene43614\_All, Unigene4181\_All, CL896.Contig24\_All, CL91.Contig9\_All, CL13596.Contig4\_All, CL721.Contig4\_All, Unigene8835\_All, CL3820.Contig3\_All, Unigene58622\_All, Unigene22244\_All, Unigene61490\_All, Unigene41288\_All, CL13641.Contig1\_All, Unigene3390\_All, CL9286.Contig2\_All, CL9799.Contig1\_All, Unigene19421\_All, Unigene2588\_All, CL2964.Contig13\_All, Unigene5641\_All, Unigene6780\_All, CL896.Contig16\_All, CL896.Contig17\_All, Unigene28581\_All, CL553.Contig2\_All, Unigene32107\_All, CL13378.Contig2\_All, Unigene41282\_All, CL2907.Contig3\_All, CL896.Contig25\_All, Unigene1982\_All, Unigene5929\_All, CL11358.Contig2\_All, CL896.Contig26\_All, CL2964.Contig3\_All, Unigene30962\_All, Unigene56276\_All, Unigene27024\_All, CL91.Contig6\_All, Unigene58085\_All, Unigene57248\_All, Unigene30448\_All, CL6178.Contig3\_All, CL12484.Contig2\_All, Unigene6846\_All, Unigene58864\_All, CL12697.Contig2\_All, Unigene12371\_All, Unigene61177\_All, Unigene3674\_All, CL12624.Contig1\_All, CL2964.Contig8\_All, Unigene259\_All, Unigene14807\_All, CL896.Contig27\_All, Unigene14730\_All, CL10354.Contig1\_All, CL896.Contig5\_All, CL12484.Contig1\_All, Unigene33863\_All, CL6985.Contig1\_All, CL9286.Contig3\_All, Unigene60300\_All, CL826.Contig1\_All, CL2320.Contig2\_All |
| antioxidant activity | Unigene10721\_All, Unigene13945\_All, CL12898.Contig3\_All, Unigene27052\_All, Unigene55407\_All, CL7825.Contig1\_All, CL173.Contig24\_All, Unigene654\_All, CL2024.Contig2\_All, CL6791.Contig4\_All, CL3078.Contig1\_All, CL10179.Contig1\_All, Unigene6476\_All, CL8814.Contig4\_All, CL2024.Contig1\_All, CL1575.Contig2\_All, CL5138.Contig4\_All, CL173.Contig17\_All, Unigene29237\_All, Unigene12548\_All, CL5511.Contig4\_All, CL10185.Contig1\_All, Unigene62497\_All, Unigene9046\_All, Unigene40276\_All, Unigene22601\_All, Unigene29002\_All, Unigene46084\_All, Unigene55939\_All, CL1395.Contig2\_All, CL5397.Contig3\_All, CL173.Contig44\_All, CL9400.Contig2\_All, Unigene55746\_All, CL6791.Contig2\_All, CL1575.Contig1\_All, Unigene23799\_All, CL173.Contig35\_All, CL11167.Contig1\_All, CL3593.Contig3\_All, CL9625.Contig2\_All, CL9327.Contig1\_All, Unigene32711\_All, CL7181.Contig2\_All, Unigene59106\_All, Unigene15415\_All, Unigene60681\_All, CL8497.Contig1\_All, CL7181.Contig4\_All, Unigene43534\_All, CL5138.Contig1\_All, Unigene11976\_All, Unigene59586\_All, CL7743.Contig3\_All, Unigene56665\_All, CL13309.Contig1\_All, CL7371.Contig2\_All, Unigene10514\_All, Unigene11831\_All, Unigene25662\_All, CL2537.Contig2\_All, Unigene40537\_All, CL3593.Contig2\_All, CL1395.Contig1\_All, CL11644.Contig1\_All, Unigene22107\_All, Unigene62945\_All, Unigene27006\_All, Unigene55335\_All, Unigene40499\_All, CL7371.Contig1\_All, CL1455.Contig2\_All, Unigene13541\_All, CL9538.Contig2\_All, CL11535.Contig3\_All, Unigene61811\_All, CL173.Contig11\_All, CL2024.Contig3\_All, CL5082.Contig1\_All, CL173.Contig14\_All, Unigene33089\_All, Unigene58449\_All, CL9538.Contig1\_All, Unigene29492\_All, Unigene64694\_All, CL6791.Contig1\_All, CL6791.Contig5\_All, CL10185.Contig2\_All, Unigene488\_All, Unigene14776\_All, Unigene33961\_All, Unigene61043\_All, Unigene15957\_All, CL11491.Contig1\_All, Unigene15457\_All, Unigene57168\_All, Unigene23765\_All, CL7181.Contig5\_All, CL11644.Contig2\_All |
| aspartyl esterase activity | Unigene20570\_All, Unigene62708\_All, Unigene16215\_All, Unigene39814\_All, Unigene58302\_All, CL7661.Contig1\_All, Unigene56753\_All, Unigene11251\_All, Unigene5361\_All, CL2390.Contig3\_All, CL2390.Contig1\_All, Unigene7322\_All, CL3136.Contig2\_All, Unigene61904\_All, CL4292.Contig3\_All, CL7661.Contig2\_All, Unigene10181\_All, CL4292.Contig1\_All, CL8796.Contig2\_All, Unigene35873\_All |
| protein dimerization activity | Unigene26228\_All, Unigene14232\_All, Unigene3242\_All, Unigene7225\_All, CL3613.Contig3\_All, Unigene27170\_All, Unigene55248\_All, CL11598.Contig2\_All, CL87.Contig2\_All, CL1413.Contig1\_All, Unigene34037\_All, CL1481.Contig13\_All, Unigene2140\_All, CL925.Contig4\_All, Unigene54414\_All, Unigene48099\_All, Unigene814\_All, CL4776.Contig1\_All, CL1993.Contig5\_All, Unigene59365\_All, Unigene18047\_All, Unigene60054\_All, Unigene59272\_All, Unigene57783\_All, CL1993.Contig7\_All, CL967.Contig6\_All, Unigene26304\_All, CL13928.Contig3\_All, Unigene7222\_All, CL5286.Contig2\_All, CL9395.Contig1\_All, CL1390.Contig2\_All, Unigene57590\_All, Unigene13015\_All, CL1229.Contig8\_All, Unigene33789\_All, CL7316.Contig2\_All, Unigene39698\_All, CL1481.Contig2\_All, Unigene11037\_All, Unigene13760\_All, CL1002.Contig2\_All, Unigene16780\_All, Unigene34381\_All, CL8943.Contig1\_All, CL10085.Contig3\_All, CL1229.Contig3\_All, CL967.Contig7\_All, Unigene59986\_All, Unigene39204\_All, Unigene56835\_All, CL8242.Contig2\_All, Unigene19567\_All, CL10650.Contig1\_All, CL6283.Contig1\_All, CL1993.Contig12\_All, CL5699.Contig3\_All, Unigene55249\_All, Unigene27654\_All, CL1993.Contig4\_All, Unigene35395\_All, CL12409.Contig1\_All, Unigene1468\_All, CL8095.Contig2\_All, Unigene27205\_All, CL8558.Contig1\_All, CL10654.Contig2\_All, CL10654.Contig1\_All, Unigene34149\_All, CL1993.Contig3\_All, CL6289.Contig2\_All, Unigene8067\_All, CL37.Contig7\_All, Unigene26522\_All, CL5958.Contig1\_All, CL7316.Contig1\_All, Unigene8972\_All, CL1229.Contig5\_All, Unigene1479\_All, CL4293.Contig1\_All, Unigene16775\_All, Unigene62596\_All, Unigene40707\_All, Unigene42278\_All, CL6222.Contig1\_All, CL1993.Contig8\_All, Unigene60846\_All, Unigene25841\_All, CL1390.Contig1\_All, CL13755.Contig1\_All, Unigene30102\_All, Unigene5212\_All, Unigene27060\_All, CL9354.Contig4\_All, CL3960.Contig2\_All, CL1993.Contig1\_All, Unigene1478\_All, CL13407.Contig1\_All, Unigene35457\_All, CL1229.Contig4\_All, CL6289.Contig1\_All, Unigene54415\_All, Unigene26385\_All, Unigene60107\_All, CL1993.Contig11\_All, Unigene58690\_All, Unigene6889\_All, Unigene7928\_All, Unigene58270\_All, CL1993.Contig2\_All, Unigene6536\_All, Unigene54417\_All, Unigene40494\_All, Unigene39696\_All, CL13928.Contig1\_All, CL7688.Contig2\_All, CL2071.Contig5\_All, CL10085.Contig2\_All, CL1993.Contig9\_All, CL925.Contig1\_All, Unigene59471\_All, Unigene58045\_All, Unigene54439\_All, Unigene16906\_All, Unigene58596\_All, Unigene6250\_All, CL9656.Contig3\_All, Unigene3687\_All, Unigene32903\_All, Unigene27222\_All, Unigene22502\_All, Unigene61417\_All, CL3125.Contig2\_All, Unigene59271\_All, Unigene60273\_All, CL626.Contig1\_All, CL3196.Contig5\_All, CL5286.Contig1\_All, CL3125.Contig1\_All, Unigene59413\_All, Unigene21454\_All, Unigene23337\_All, CL1993.Contig10\_All, Unigene58317\_All, CL9272.Contig1\_All, Unigene3569\_All, Unigene11552\_All, CL4847.Contig1\_All, Unigene32033\_All, Unigene18068\_All, CL13928.Contig2\_All, Unigene7585\_All, CL1229.Contig14\_All, Unigene26246\_All, CL7370.Contig1\_All, Unigene58997\_All, CL6222.Contig2\_All, CL12396.Contig1\_All, Unigene34713\_All, CL6978.Contig1\_All, CL87.Contig3\_All, Unigene10084\_All, Unigene1471\_All, CL7188.Contig1\_All, Unigene38540\_All, CL1229.Contig7\_All, Unigene46995\_All, CL3831.Contig2\_All, CL1229.Contig10\_All, CL10546.Contig1\_All, CL2187.Contig2\_All, Unigene35613\_All, CL608.Contig1\_All, Unigene17052\_All, CL2260.Contig2\_All, Unigene54423\_All, CL7818.Contig2\_All, CL5750.Contig1\_All, Unigene56286\_All, Unigene20992\_All, CL11028.Contig1\_All, CL1993.Contig6\_All, CL4681.Contig1\_All, Unigene17682\_All, Unigene18850\_All, Unigene35022\_All, Unigene5932\_All, CL1229.Contig2\_All, Unigene2267\_All, Unigene18782\_All, CL5268.Contig1\_All, Unigene59412\_All, Unigene61431\_All, CL13407.Contig2\_All, Unigene20722\_All, CL2440.Contig1\_All, CL5750.Contig2\_All, Unigene10286\_All, Unigene18169\_All, Unigene34380\_All, CL9125.Contig2\_All, CL1229.Contig6\_All, CL5958.Contig2\_All, Unigene19297\_All, CL7688.Contig3\_All, Unigene23386\_All, Unigene3440\_All, Unigene39158\_All |
| beta-glucosidase activity | CL1913.Contig1\_All, CL3868.Contig2\_All, CL385.Contig7\_All, Unigene60460\_All, CL385.Contig6\_All, Unigene16765\_All, CL3160.Contig2\_All, CL385.Contig3\_All, CL10796.Contig2\_All, CL3280.Contig1\_All, Unigene41339\_All, CL633.Contig1\_All, CL9307.Contig1\_All, CL8551.Contig3\_All, CL385.Contig9\_All, Unigene30453\_All, CL633.Contig2\_All, Unigene13792\_All, Unigene55413\_All, CL4118.Contig1\_All, CL385.Contig5\_All, Unigene9450\_All, CL13470.Contig3\_All, CL5990.Contig2\_All, Unigene20465\_All, Unigene26161\_All, CL9293.Contig2\_All, CL1913.Contig2\_All, Unigene61065\_All, Unigene61176\_All, CL2168.Contig2\_All, Unigene21967\_All, CL5990.Contig1\_All, CL385.Contig1\_All, Unigene61508\_All, CL3421.Contig1\_All, CL12823.Contig1\_All, CL9610.Contig2\_All, Unigene62965\_All, CL3868.Contig1\_All |
| cellulase activity | Unigene61418\_All, CL2457.Contig1\_All, Unigene60222\_All, Unigene55955\_All, CL982.Contig5\_All, CL6141.Contig7\_All, CL3395.Contig2\_All, CL982.Contig1\_All, Unigene64775\_All, Unigene32356\_All, CL982.Contig4\_All, CL5325.Contig2\_All, Unigene60005\_All, CL5325.Contig1\_All, Unigene24573\_All, CL982.Contig3\_All, CL982.Contig6\_All, Unigene23554\_All, Unigene58374\_All, Unigene21171\_All, CL6141.Contig9\_All, Unigene62888\_All, CL982.Contig2\_All |
| pectinesterase activity | Unigene20570\_All, CL3277.Contig3\_All, Unigene62708\_All, Unigene16215\_All, Unigene39814\_All, Unigene58302\_All, CL7661.Contig1\_All, Unigene56753\_All, CL3277.Contig2\_All, CL3277.Contig1\_All, Unigene11251\_All, Unigene56056\_All, Unigene5361\_All, CL2390.Contig3\_All, CL2390.Contig1\_All, Unigene7322\_All, CL8686.Contig2\_All, CL3136.Contig2\_All, Unigene61904\_All, CL4292.Contig3\_All, CL7661.Contig2\_All, CL4292.Contig1\_All, Unigene10181\_All, CL8796.Contig2\_All, Unigene35873\_All |
| tetrapyrrole binding | Unigene25165\_All, CL1384.Contig4\_All, Unigene12838\_All, CL2799.Contig1\_All, CL4161.Contig4\_All, CL8132.Contig1\_All, CL1608.Contig3\_All, CL9328.Contig1\_All, Unigene27052\_All, CL173.Contig24\_All, Unigene654\_All, CL2024.Contig2\_All, CL12184.Contig1\_All, CL6791.Contig4\_All, CL7715.Contig1\_All, Unigene38900\_All, CL2024.Contig1\_All, CL213.Contig8\_All, CL213.Contig2\_All, CL5138.Contig4\_All, CL173.Contig17\_All, CL787.Contig2\_All, CL5511.Contig4\_All, CL10185.Contig1\_All, Unigene40276\_All, CL173.Contig44\_All, Unigene25297\_All, CL6791.Contig2\_All, CL3333.Contig4\_All, CL6555.Contig7\_All, Unigene44382\_All, CL173.Contig35\_All, Unigene8100\_All, CL11167.Contig1\_All, Unigene12972\_All, Unigene58425\_All, Unigene60002\_All, CL13490.Contig1\_All, Unigene31739\_All, Unigene25504\_All, CL5296.Contig1\_All, CL2799.Contig4\_All, CL5138.Contig1\_All, Unigene56665\_All, CL1045.Contig1\_All, CL9711.Contig3\_All, CL2537.Contig2\_All, CL7715.Contig3\_All, CL1395.Contig1\_All, CL10025.Contig1\_All, Unigene22107\_All, CL11644.Contig1\_All, CL3875.Contig1\_All, Unigene58911\_All, Unigene13541\_All, CL11300.Contig2\_All, Unigene29847\_All, Unigene55459\_All, Unigene18987\_All, Unigene29872\_All, CL5082.Contig1\_All, CL173.Contig14\_All, Unigene33089\_All, CL9538.Contig1\_All, Unigene29492\_All, CL10185.Contig2\_All, CL960.Contig2\_All, CL8237.Contig2\_All, Unigene14151\_All, CL12628.Contig2\_All, CL11627.Contig2\_All, CL5663.Contig2\_All, CL7181.Contig5\_All, CL1817.Contig1\_All, CL8347.Contig2\_All, CL10659.Contig1\_All, Unigene55407\_All, CL13917.Contig2\_All, CL10179.Contig1\_All, Unigene6476\_All, Unigene29237\_All, CL10224.Contig1\_All, CL8347.Contig1\_All, Unigene62497\_All, Unigene29002\_All, CL1395.Contig2\_All, CL2330.Contig4\_All, CL1384.Contig1\_All, Unigene18990\_All, CL5397.Contig3\_All, Unigene55746\_All, Unigene23512\_All, Unigene22717\_All, Unigene3685\_All, CL11627.Contig3\_All, Unigene32711\_All, CL7181.Contig2\_All, Unigene59106\_All, Unigene57657\_All, CL960.Contig1\_All, Unigene15415\_All, Unigene65156\_All, Unigene32218\_All, CL1817.Contig5\_All, Unigene60681\_All, CL3429.Contig2\_All, CL7181.Contig4\_All, CL6903.Contig2\_All, CL2330.Contig2\_All, Unigene37291\_All, CL213.Contig7\_All, CL11417.Contig2\_All, Unigene11976\_All, CL7743.Contig3\_All, Unigene33962\_All, Unigene29581\_All, Unigene61438\_All, CL787.Contig1\_All, CL7371.Contig2\_All, Unigene40537\_All, CL3429.Contig1\_All, Unigene55335\_All, Unigene27006\_All, CL7371.Contig1\_All, Unigene30566\_All, Unigene62583\_All, CL9538.Contig2\_All, CL11535.Contig3\_All, CL173.Contig11\_All, CL2024.Contig3\_All, CL4161.Contig1\_All, Unigene23747\_All, Unigene58449\_All, CL10659.Contig2\_All, CL6791.Contig5\_All, CL6791.Contig1\_All, Unigene5926\_All, CL3930.Contig5\_All, Unigene488\_All, Unigene43661\_All, Unigene14776\_All, Unigene15957\_All, CL4161.Contig2\_All, Unigene13893\_All, CL4161.Contig3\_All, Unigene23765\_All, CL11644.Contig2\_All |
| FMN reductase activity | CL7072.Contig1\_All, CL7072.Contig2\_All, Unigene27257\_All, Unigene29003\_All, Unigene4069\_All |
| glucan endo-1,3-beta-D-glucosidase activity | Unigene13792\_All, CL3868.Contig2\_All, Unigene55413\_All, Unigene60460\_All, Unigene16765\_All, CL3160.Contig2\_All, CL10796.Contig2\_All, CL3280.Contig1\_All, Unigene20465\_All, Unigene26161\_All, CL633.Contig1\_All, CL9307.Contig1\_All, CL9293.Contig2\_All, Unigene61065\_All, Unigene21967\_All, Unigene30453\_All, CL633.Contig2\_All, Unigene61508\_All, CL12823.Contig1\_All, CL3868.Contig1\_All, Unigene62965\_All |
| nutrient reservoir activity | CL7100.Contig2\_All, Unigene22601\_All, Unigene32233\_All, CL2967.Contig2\_All, Unigene56732\_All, CL7100.Contig3\_All, Unigene62513\_All, CL3993.Contig3\_All, Unigene2876\_All, CL9625.Contig2\_All, Unigene25662\_All, CL6700.Contig1\_All, CL2967.Contig3\_All, Unigene61043\_All, Unigene29909\_All, CL6181.Contig2\_All, CL10910.Contig1\_All, CL8814.Contig4\_All, Unigene21419\_All, CL10307.Contig1\_All, Unigene10624\_All, Unigene28979\_All |
| glucosidase activity | CL1913.Contig1\_All, CL3868.Contig2\_All, CL385.Contig7\_All, Unigene60460\_All, CL385.Contig6\_All, Unigene16765\_All, CL3160.Contig2\_All, CL12460.Contig1\_All, CL385.Contig3\_All, CL10796.Contig2\_All, CL3280.Contig1\_All, Unigene41339\_All, CL633.Contig1\_All, CL9307.Contig1\_All, CL8551.Contig3\_All, CL385.Contig9\_All, Unigene30453\_All, CL633.Contig2\_All, Unigene37456\_All, Unigene13792\_All, Unigene55413\_All, CL4118.Contig1\_All, CL385.Contig5\_All, Unigene9450\_All, CL13470.Contig3\_All, CL5990.Contig2\_All, Unigene20465\_All, Unigene26161\_All, CL9293.Contig2\_All, CL1913.Contig2\_All, Unigene61065\_All, CL5360.Contig3\_All, CL5360.Contig4\_All, Unigene61176\_All, CL2168.Contig2\_All, Unigene21967\_All, Unigene26792\_All, CL5990.Contig1\_All, Unigene61508\_All, CL385.Contig1\_All, CL3421.Contig1\_All, CL12823.Contig1\_All, CL9610.Contig2\_All, Unigene62965\_All, CL3868.Contig1\_All |
| heme binding | Unigene25165\_All, Unigene12838\_All, CL2799.Contig1\_All, CL4161.Contig4\_All, CL8132.Contig1\_All, CL1608.Contig3\_All, CL9328.Contig1\_All, Unigene27052\_All, CL173.Contig24\_All, Unigene654\_All, CL2024.Contig2\_All, CL12184.Contig1\_All, CL6791.Contig4\_All, CL7715.Contig1\_All, Unigene38900\_All, CL2024.Contig1\_All, CL5138.Contig4\_All, CL173.Contig17\_All, CL787.Contig2\_All, CL5511.Contig4\_All, CL10185.Contig1\_All, Unigene40276\_All, CL173.Contig44\_All, Unigene25297\_All, CL6791.Contig2\_All, CL3333.Contig4\_All, CL6555.Contig7\_All, Unigene44382\_All, CL173.Contig35\_All, Unigene8100\_All, CL11167.Contig1\_All, Unigene12972\_All, Unigene58425\_All, Unigene60002\_All, CL13490.Contig1\_All, Unigene31739\_All, Unigene25504\_All, CL5296.Contig1\_All, CL2799.Contig4\_All, CL5138.Contig1\_All, Unigene56665\_All, CL1045.Contig1\_All, CL9711.Contig3\_All, CL2537.Contig2\_All, CL7715.Contig3\_All, CL1395.Contig1\_All, Unigene22107\_All, CL11644.Contig1\_All, CL3875.Contig1\_All, Unigene58911\_All, Unigene13541\_All, CL11300.Contig2\_All, Unigene29847\_All, Unigene55459\_All, Unigene18987\_All, Unigene29872\_All, CL5082.Contig1\_All, CL173.Contig14\_All, Unigene33089\_All, CL9538.Contig1\_All, Unigene29492\_All, CL10185.Contig2\_All, CL960.Contig2\_All, CL8237.Contig2\_All, Unigene14151\_All, CL12628.Contig2\_All, CL11627.Contig2\_All, CL5663.Contig2\_All, CL7181.Contig5\_All, CL1817.Contig1\_All, CL10659.Contig1\_All, Unigene55407\_All, CL13917.Contig2\_All, CL10179.Contig1\_All, Unigene6476\_All, Unigene29237\_All, CL10224.Contig1\_All, Unigene62497\_All, Unigene29002\_All, CL1395.Contig2\_All, CL2330.Contig4\_All, Unigene18990\_All, CL5397.Contig3\_All, Unigene55746\_All, Unigene23512\_All, Unigene22717\_All, Unigene3685\_All, CL11627.Contig3\_All, Unigene32711\_All, CL7181.Contig2\_All, Unigene59106\_All, Unigene57657\_All, CL960.Contig1\_All, Unigene15415\_All, Unigene65156\_All, CL1817.Contig5\_All, Unigene60681\_All, CL7181.Contig4\_All, CL6903.Contig2\_All, CL2330.Contig2\_All, Unigene37291\_All, CL11417.Contig2\_All, Unigene11976\_All, CL7743.Contig3\_All, Unigene33962\_All, Unigene29581\_All, Unigene61438\_All, CL787.Contig1\_All, CL7371.Contig2\_All, Unigene40537\_All, Unigene55335\_All, Unigene27006\_All, CL7371.Contig1\_All, Unigene30566\_All, Unigene62583\_All, CL9538.Contig2\_All, CL11535.Contig3\_All, CL173.Contig11\_All, CL2024.Contig3\_All, CL4161.Contig1\_All, Unigene23747\_All, Unigene58449\_All, CL10659.Contig2\_All, CL6791.Contig5\_All, CL6791.Contig1\_All, Unigene5926\_All, CL3930.Contig5\_All, Unigene488\_All, Unigene43661\_All, Unigene14776\_All, Unigene15957\_All, CL4161.Contig2\_All, Unigene13893\_All, CL4161.Contig3\_All, Unigene23765\_All, CL11644.Contig2\_All |
| glutamate synthase activity, NAD(P)H as acceptor | Unigene26932\_All, CL2702.Contig2\_All, CL2702.Contig4\_All, Unigene702\_All, Unigene23920\_All, Unigene39960\_All, CL5832.Contig1\_All |
| NADP+ binding | Unigene29396\_All, CL9128.Contig2\_All, CL9128.Contig1\_All, CL3610.Contig2\_All, CL3610.Contig7\_All, CL12503.Contig2\_All, CL3610.Contig5\_All |
| L-tyrosine aminotransferase activity | CL10234.Contig2\_All, Unigene4967\_All, CL10234.Contig1\_All, CL142.Contig2\_All, CL2877.Contig1\_All, CL142.Contig1\_All, Unigene22816\_All |
| GTP binding | CL13393.Contig1\_All, CL896.Contig15\_All, Unigene56323\_All, CL1222.Contig9\_All, CL2589.Contig2\_All, Unigene14305\_All, Unigene14583\_All, Unigene41882\_All, CL5260.Contig2\_All, Unigene33359\_All, Unigene13688\_All, CL8114.Contig3\_All, CL896.Contig30\_All, CL1974.Contig3\_All, CL9891.Contig1\_All, CL8114.Contig1\_All, CL9286.Contig1\_All, CL2782.Contig1\_All, Unigene6347\_All, Unigene7380\_All, CL3049.Contig5\_All, CL5116.Contig1\_All, Unigene27997\_All, Unigene3973\_All, Unigene56781\_All, CL967.Contig6\_All, CL2589.Contig6\_All, Unigene7418\_All, CL12325.Contig1\_All, CL6256.Contig2\_All, Unigene11244\_All, Unigene56324\_All, CL12624.Contig3\_All, CL13949.Contig1\_All, Unigene39698\_All, Unigene26271\_All, Unigene61511\_All, Unigene6773\_All, Unigene7098\_All, CL967.Contig7\_All, Unigene14819\_All, CL13973.Contig1\_All, Unigene42382\_All, CL896.Contig18\_All, CL4961.Contig1\_All, Unigene61142\_All, Unigene40080\_All, Unigene25646\_All, Unigene42887\_All, CL7207.Contig2\_All, Unigene19302\_All, CL2964.Contig12\_All, CL8558.Contig1\_All, CL3019.Contig2\_All, CL896.Contig11\_All, Unigene56244\_All, Unigene2386\_All, CL366.Contig4\_All, Unigene7432\_All, Unigene16373\_All, Unigene358\_All, CL8220.Contig3\_All, Unigene24620\_All, CL13545.Contig1\_All, Unigene56915\_All, CL896.Contig13\_All, CL2504.Contig1\_All, Unigene4536\_All, Unigene21196\_All, CL2782.Contig2\_All, Unigene57766\_All, Unigene59157\_All, Unigene19828\_All, Unigene7402\_All, CL11358.Contig4\_All, Unigene10801\_All, Unigene54944\_All, Unigene901\_All, CL1084.Contig3\_All, CL1323.Contig2\_All, Unigene13396\_All, Unigene17432\_All, Unigene57619\_All, CL3049.Contig1\_All, CL13641.Contig3\_All, CL13393.Contig2\_All, Unigene57765\_All, Unigene29969\_All, CL13476.Contig4\_All, Unigene1316\_All, CL9257.Contig5\_All, CL5313.Contig3\_All, CL5260.Contig1\_All, CL10245.Contig1\_All, Unigene56245\_All, Unigene3781\_All, CL896.Contig19\_All, CL896.Contig33\_All, Unigene33862\_All, Unigene23394\_All, CL8248.Contig7\_All, CL967.Contig8\_All, CL13861.Contig5\_All, Unigene60959\_All, CL12697.Contig1\_All, Unigene55760\_All, Unigene56196\_All, CL1084.Contig4\_All, Unigene44279\_All, CL896.Contig20\_All, CL4186.Contig2\_All, CL12946.Contig1\_All, Unigene17163\_All, CL9362.Contig1\_All, CL2579.Contig2\_All, Unigene30740\_All, Unigene33360\_All, CL13378.Contig1\_All, CL6985.Contig4\_All, Unigene54938\_All, CL2589.Contig3\_All, Unigene4349\_All, CL6188.Contig3\_All, Unigene12757\_All, CL366.Contig1\_All, CL3019.Contig1\_All, Unigene30179\_All, Unigene13534\_All, CL4324.Contig2\_All, Unigene12291\_All, Unigene43614\_All, Unigene4181\_All, CL896.Contig24\_All, CL91.Contig9\_All, CL13596.Contig4\_All, Unigene43514\_All, Unigene8835\_All, CL3820.Contig3\_All, Unigene36146\_All, Unigene58622\_All, Unigene41288\_All, Unigene61490\_All, Unigene28645\_All, Unigene22244\_All, CL13641.Contig1\_All, Unigene3390\_All, CL9286.Contig2\_All, CL9799.Contig1\_All, Unigene19421\_All, Unigene2588\_All, CL2964.Contig13\_All, Unigene6780\_All, CL896.Contig16\_All, CL896.Contig17\_All, Unigene28581\_All, CL553.Contig2\_All, CL6426.Contig1\_All, Unigene7153\_All, Unigene32107\_All, CL13378.Contig2\_All, Unigene46442\_All, CL1021.Contig1\_All, Unigene41282\_All, CL2907.Contig3\_All, CL896.Contig25\_All, Unigene1982\_All, CL11257.Contig3\_All, Unigene5929\_All, CL1323.Contig1\_All, CL11257.Contig1\_All, CL11358.Contig2\_All, CL896.Contig26\_All, CL13596.Contig6\_All, CL2964.Contig3\_All, Unigene30962\_All, Unigene56276\_All, Unigene27024\_All, Unigene17052\_All, CL91.Contig6\_All, Unigene58085\_All, Unigene30448\_All, Unigene57248\_All, CL518.Contig2\_All, CL6178.Contig3\_All, CL10456.Contig2\_All, CL12484.Contig2\_All, Unigene6846\_All, Unigene58864\_All, CL12697.Contig2\_All, CL5300.Contig2\_All, CL3768.Contig2\_All, Unigene12371\_All, Unigene61177\_All, Unigene3674\_All, CL12624.Contig1\_All, CL2964.Contig8\_All, Unigene259\_All, Unigene14807\_All, Unigene55287\_All, CL896.Contig27\_All, Unigene14730\_All, CL896.Contig5\_All, CL10354.Contig1\_All, CL12484.Contig1\_All, Unigene33863\_All, CL5278.Contig2\_All, CL6985.Contig1\_All, CL9286.Contig3\_All, Unigene60300\_All, Unigene56733\_All, Unigene14004\_All, CL7522.Contig1\_All, CL2320.Contig2\_All |
| guanyl ribonucleotide binding | CL13393.Contig1\_All, CL896.Contig15\_All, Unigene56323\_All, CL1222.Contig9\_All, CL2589.Contig2\_All, Unigene14305\_All, Unigene14583\_All, Unigene41882\_All, CL5260.Contig2\_All, Unigene33359\_All, Unigene13688\_All, CL8114.Contig3\_All, CL896.Contig30\_All, CL1974.Contig3\_All, CL9891.Contig1\_All, CL8114.Contig1\_All, CL9286.Contig1\_All, CL2782.Contig1\_All, Unigene6347\_All, Unigene7380\_All, CL3049.Contig5\_All, CL5116.Contig1\_All, Unigene27997\_All, Unigene3973\_All, Unigene56781\_All, CL967.Contig6\_All, CL2589.Contig6\_All, Unigene7418\_All, CL12325.Contig1\_All, CL6256.Contig2\_All, Unigene11244\_All, Unigene56324\_All, CL12624.Contig3\_All, CL13949.Contig1\_All, Unigene39698\_All, Unigene26271\_All, Unigene61511\_All, Unigene6773\_All, Unigene7098\_All, CL967.Contig7\_All, Unigene14819\_All, CL13973.Contig1\_All, Unigene42382\_All, CL896.Contig18\_All, CL4961.Contig1\_All, Unigene61142\_All, Unigene40080\_All, Unigene25646\_All, Unigene42887\_All, CL7207.Contig2\_All, Unigene19302\_All, CL2964.Contig12\_All, CL8558.Contig1\_All, CL3019.Contig2\_All, CL896.Contig11\_All, Unigene56244\_All, Unigene2386\_All, CL366.Contig4\_All, Unigene7432\_All, Unigene16373\_All, Unigene358\_All, CL8220.Contig3\_All, Unigene24620\_All, CL13545.Contig1\_All, Unigene56915\_All, CL896.Contig13\_All, CL2504.Contig1\_All, Unigene4536\_All, Unigene21196\_All, CL2782.Contig2\_All, Unigene57766\_All, Unigene59157\_All, Unigene19828\_All, Unigene7402\_All, CL11358.Contig4\_All, Unigene10801\_All, Unigene54944\_All, Unigene901\_All, CL1084.Contig3\_All, CL1323.Contig2\_All, Unigene13396\_All, Unigene17432\_All, Unigene57619\_All, CL3049.Contig1\_All, CL13641.Contig3\_All, CL13393.Contig2\_All, Unigene57765\_All, Unigene29969\_All, CL13476.Contig4\_All, Unigene1316\_All, CL9257.Contig5\_All, CL5313.Contig3\_All, CL5260.Contig1\_All, CL10245.Contig1\_All, Unigene56245\_All, Unigene3781\_All, CL896.Contig19\_All, CL896.Contig33\_All, Unigene33862\_All, Unigene23394\_All, CL8248.Contig7\_All, CL967.Contig8\_All, CL13861.Contig5\_All, Unigene60959\_All, CL12697.Contig1\_All, Unigene55760\_All, Unigene56196\_All, CL1084.Contig4\_All, Unigene44279\_All, CL896.Contig20\_All, CL4186.Contig2\_All, CL12946.Contig1\_All, Unigene17163\_All, CL9362.Contig1\_All, CL2579.Contig2\_All, Unigene30740\_All, Unigene33360\_All, CL13378.Contig1\_All, CL6985.Contig4\_All, Unigene54938\_All, CL2589.Contig3\_All, Unigene4349\_All, CL6188.Contig3\_All, Unigene12757\_All, CL366.Contig1\_All, CL3019.Contig1\_All, Unigene30179\_All, Unigene13534\_All, CL4324.Contig2\_All, Unigene12291\_All, Unigene43614\_All, Unigene4181\_All, CL896.Contig24\_All, CL91.Contig9\_All, CL13596.Contig4\_All, Unigene43514\_All, Unigene8835\_All, CL3820.Contig3\_All, Unigene36146\_All, Unigene58622\_All, Unigene41288\_All, Unigene61490\_All, Unigene28645\_All, Unigene22244\_All, CL13641.Contig1\_All, Unigene3390\_All, CL9286.Contig2\_All, CL9799.Contig1\_All, Unigene19421\_All, Unigene2588\_All, CL2964.Contig13\_All, Unigene6780\_All, CL896.Contig16\_All, CL896.Contig17\_All, Unigene28581\_All, CL553.Contig2\_All, CL6426.Contig1\_All, Unigene7153\_All, Unigene32107\_All, CL13378.Contig2\_All, Unigene46442\_All, CL1021.Contig1\_All, Unigene41282\_All, CL2907.Contig3\_All, CL896.Contig25\_All, Unigene1982\_All, CL11257.Contig3\_All, Unigene5929\_All, CL1323.Contig1\_All, CL11257.Contig1\_All, CL11358.Contig2\_All, CL896.Contig26\_All, CL13596.Contig6\_All, CL2964.Contig3\_All, Unigene30962\_All, Unigene56276\_All, Unigene27024\_All, Unigene17052\_All, CL91.Contig6\_All, Unigene58085\_All, Unigene30448\_All, Unigene57248\_All, CL518.Contig2\_All, CL6178.Contig3\_All, CL10456.Contig2\_All, CL12484.Contig2\_All, Unigene6846\_All, Unigene58864\_All, CL12697.Contig2\_All, CL5300.Contig2\_All, CL3768.Contig2\_All, Unigene12371\_All, Unigene61177\_All, Unigene3674\_All, CL12624.Contig1\_All, CL2964.Contig8\_All, Unigene259\_All, Unigene14807\_All, Unigene55287\_All, CL896.Contig27\_All, Unigene14730\_All, CL896.Contig5\_All, CL10354.Contig1\_All, CL12484.Contig1\_All, Unigene33863\_All, CL5278.Contig2\_All, CL6985.Contig1\_All, CL9286.Contig3\_All, Unigene60300\_All, Unigene56733\_All, Unigene14004\_All, CL7522.Contig1\_All, CL2320.Contig2\_All |
| enzyme inhibitor activity | CL8559.Contig1\_All, CL9424.Contig1\_All, CL10777.Contig2\_All, Unigene58302\_All, CL9310.Contig1\_All, Unigene60767\_All, CL9706.Contig7\_All, CL10209.Contig2\_All, Unigene11251\_All, CL1005.Contig1\_All, CL8559.Contig2\_All, CL10209.Contig1\_All, Unigene12387\_All, CL2390.Contig1\_All, Unigene7322\_All, CL3136.Contig2\_All, CL4292.Contig1\_All, Unigene10181\_All, Unigene55451\_All, CL12131.Contig2\_All, Unigene15971\_All, CL8796.Contig2\_All, CL11038.Contig1\_All, Unigene41946\_All, CL1005.Contig2\_All, Unigene16215\_All, Unigene18854\_All, CL2022.Contig1\_All, Unigene5361\_All, CL2390.Contig3\_All, CL8686.Contig2\_All, Unigene61904\_All, CL4292.Contig3\_All, Unigene36095\_All, Unigene9218\_All |
| oxidoreductase activity, acting on the CH-OH group of donors, NAD or NADP as acceptor | Unigene21226\_All, Unigene27064\_All, CL3879.Contig1\_All, Unigene30214\_All, Unigene61740\_All, CL3596.Contig2\_All, CL8737.Contig1\_All, CL9651.Contig1\_All, Unigene39020\_All, CL3610.Contig7\_All, CL6062.Contig1\_All, CL12769.Contig2\_All, CL4781.Contig2\_All, CL11925.Contig1\_All, CL2378.Contig1\_All, CL9128.Contig1\_All, CL12933.Contig1\_All, CL949.Contig1\_All, CL7390.Contig1\_All, CL949.Contig3\_All, Unigene54419\_All, CL7004.Contig2\_All, CL7072.Contig2\_All, CL2814.Contig4\_All, Unigene17142\_All, CL12503.Contig2\_All, Unigene63349\_All, Unigene61476\_All, CL2912.Contig1\_All, CL737.Contig1\_All, CL5178.Contig5\_All, Unigene56231\_All, Unigene18514\_All, CL258.Contig2\_All, CL1053.Contig2\_All, CL3610.Contig5\_All, CL1283.Contig4\_All, CL2814.Contig6\_All, Unigene56958\_All, CL8682.Contig2\_All, CL6062.Contig3\_All, CL1053.Contig1\_All, Unigene4039\_All, Unigene47014\_All, CL8682.Contig1\_All, Unigene14349\_All, CL4483.Contig2\_All, Unigene30252\_All, Unigene54418\_All, Unigene26135\_All, CL11865.Contig1\_All, CL5178.Contig4\_All, Unigene54422\_All, Unigene31267\_All, CL2120.Contig4\_All, CL8073.Contig1\_All, CL11731.Contig1\_All, CL6775.Contig2\_All, Unigene6952\_All, CL5273.Contig1\_All, CL2814.Contig3\_All, Unigene9780\_All, CL3610.Contig2\_All, CL8001.Contig1\_All, Unigene58324\_All, Unigene10603\_All, CL5178.Contig2\_All, CL7072.Contig1\_All, CL1986.Contig2\_All, Unigene17192\_All, Unigene55119\_All, Unigene33964\_All, Unigene59773\_All, CL12933.Contig2\_All, Unigene33138\_All, Unigene42939\_All, CL2372.Contig1\_All, CL9973.Contig3\_All, CL1001.Contig3\_All, Unigene29396\_All, Unigene32479\_All, CL4067.Contig3\_All, Unigene25365\_All, CL3574.Contig5\_All, Unigene26365\_All, Unigene57130\_All, Unigene59471\_All, Unigene60021\_All, CL7004.Contig1\_All, CL6775.Contig1\_All, Unigene912\_All, Unigene44648\_All, CL12720.Contig2\_All, CL5185.Contig2\_All, CL6487.Contig3\_All, Unigene54710\_All, CL6869.Contig3\_All, CL7100.Contig3\_All, CL3894.Contig3\_All, Unigene59012\_All, CL5178.Contig6\_All, Unigene3383\_All, Unigene54412\_All, CL4483.Contig1\_All, Unigene14655\_All, CL3273.Contig1\_All, CL5500.Contig2\_All, CL2814.Contig5\_All, Unigene42870\_All, Unigene43171\_All, Unigene22442\_All, CL12972.Contig1\_All, CL1001.Contig2\_All, CL1207.Contig3\_All, Unigene60039\_All, CL10595.Contig1\_All, Unigene4665\_All, Unigene59694\_All, CL3894.Contig1\_All, CL12434.Contig3\_All, CL9651.Contig3\_All, Unigene23051\_All, CL10595.Contig3\_All, CL4483.Contig3\_All, Unigene14167\_All, CL12396.Contig1\_All, CL9128.Contig2\_All, Unigene47694\_All, Unigene3469\_All, Unigene1557\_All, Unigene46995\_All, CL9645.Contig1\_All, Unigene58323\_All, Unigene31179\_All, CL2260.Contig2\_All, CL5178.Contig3\_All, Unigene2266\_All, CL11028.Contig1\_All, CL12769.Contig1\_All, Unigene20050\_All, CL1283.Contig5\_All, Unigene61007\_All, CL9892.Contig3\_All, CL3443.Contig2\_All, Unigene54413\_All, CL6062.Contig2\_All, CL7100.Contig2\_All, Unigene33791\_All, CL3296.Contig1\_All, CL3283.Contig3\_All, Unigene55183\_All, Unigene39069\_All, CL13390.Contig2\_All, CL3925.Contig3\_All, CL5537.Contig2\_All, CL1698.Contig1\_All, CL12434.Contig2\_All, Unigene36124\_All, Unigene51332\_All, Unigene9281\_All |
| nicotianamine synthase activity | CL8760.Contig3\_All, Unigene27935\_All, Unigene17042\_All, Unigene29715\_All, Unigene31273\_All, CL8760.Contig2\_All |
| ribonucleoside-diphosphate reductase activity, thioredoxin disulfide as acceptor | CL9766.Contig3\_All, CL9766.Contig4\_All, Unigene63816\_All, CL11777.Contig2\_All, CL631.Contig2\_All, Unigene33885\_All, Unigene42928\_All, CL11777.Contig1\_All, Unigene220\_All, Unigene55815\_All |
| oxidoreductase activity, acting on CH or CH2 groups, disulfide as acceptor | CL9766.Contig3\_All, CL9766.Contig4\_All, Unigene63816\_All, CL11777.Contig2\_All, CL631.Contig2\_All, Unigene33885\_All, Unigene42928\_All, CL11777.Contig1\_All, Unigene220\_All, Unigene55815\_All |
| guanyl nucleotide binding | CL13393.Contig1\_All, CL896.Contig15\_All, Unigene56323\_All, CL1222.Contig9\_All, CL2589.Contig2\_All, Unigene14305\_All, Unigene14583\_All, Unigene41882\_All, CL5260.Contig2\_All, Unigene33359\_All, Unigene13688\_All, CL8114.Contig3\_All, CL896.Contig30\_All, CL1974.Contig3\_All, CL9891.Contig1\_All, CL8114.Contig1\_All, CL9286.Contig1\_All, CL2782.Contig1\_All, Unigene6347\_All, Unigene7380\_All, CL3049.Contig5\_All, CL5116.Contig1\_All, Unigene27997\_All, Unigene3973\_All, Unigene56781\_All, CL967.Contig6\_All, CL2589.Contig6\_All, Unigene7418\_All, CL12325.Contig1\_All, CL6256.Contig2\_All, Unigene11244\_All, Unigene56324\_All, CL12624.Contig3\_All, CL13949.Contig1\_All, Unigene39698\_All, Unigene26271\_All, Unigene61511\_All, Unigene6773\_All, Unigene7098\_All, CL967.Contig7\_All, Unigene14819\_All, CL13973.Contig1\_All, Unigene42382\_All, CL896.Contig18\_All, CL4961.Contig1\_All, Unigene61142\_All, Unigene40080\_All, Unigene25646\_All, Unigene42887\_All, CL7207.Contig2\_All, Unigene19302\_All, CL2964.Contig12\_All, CL8558.Contig1\_All, CL3019.Contig2\_All, CL896.Contig11\_All, Unigene56244\_All, Unigene2386\_All, CL366.Contig4\_All, Unigene7432\_All, Unigene16373\_All, Unigene358\_All, CL8220.Contig3\_All, Unigene24620\_All, CL13545.Contig1\_All, Unigene56915\_All, CL896.Contig13\_All, CL2504.Contig1\_All, Unigene4536\_All, Unigene21196\_All, CL2782.Contig2\_All, Unigene57766\_All, Unigene59157\_All, Unigene19828\_All, Unigene7402\_All, CL11358.Contig4\_All, Unigene10801\_All, Unigene54944\_All, Unigene901\_All, CL1084.Contig3\_All, CL1323.Contig2\_All, Unigene13396\_All, Unigene17432\_All, Unigene57619\_All, CL3049.Contig1\_All, CL13641.Contig3\_All, CL13393.Contig2\_All, Unigene57765\_All, Unigene29969\_All, CL13476.Contig4\_All, Unigene1316\_All, CL9257.Contig5\_All, CL5313.Contig3\_All, CL5260.Contig1\_All, CL10245.Contig1\_All, Unigene56245\_All, Unigene3781\_All, CL896.Contig19\_All, CL896.Contig33\_All, Unigene33862\_All, Unigene23394\_All, CL8248.Contig7\_All, CL967.Contig8\_All, CL13861.Contig5\_All, Unigene60959\_All, CL12697.Contig1\_All, Unigene55760\_All, Unigene56196\_All, CL1084.Contig4\_All, Unigene44279\_All, CL896.Contig20\_All, CL4186.Contig2\_All, CL12946.Contig1\_All, Unigene17163\_All, CL9362.Contig1\_All, CL2579.Contig2\_All, Unigene30740\_All, Unigene33360\_All, CL13378.Contig1\_All, CL6985.Contig4\_All, Unigene54938\_All, CL2589.Contig3\_All, Unigene4349\_All, CL6188.Contig3\_All, Unigene12757\_All, CL366.Contig1\_All, CL3019.Contig1\_All, Unigene30179\_All, Unigene13534\_All, CL4324.Contig2\_All, Unigene12291\_All, Unigene43614\_All, Unigene4181\_All, CL896.Contig24\_All, CL91.Contig9\_All, CL13596.Contig4\_All, Unigene43514\_All, Unigene8835\_All, CL3820.Contig3\_All, Unigene36146\_All, Unigene58622\_All, Unigene41288\_All, Unigene61490\_All, Unigene28645\_All, Unigene22244\_All, CL13641.Contig1\_All, Unigene3390\_All, CL9286.Contig2\_All, CL9799.Contig1\_All, Unigene19421\_All, Unigene2588\_All, CL2964.Contig13\_All, Unigene6780\_All, CL896.Contig16\_All, CL896.Contig17\_All, Unigene28581\_All, CL553.Contig2\_All, CL6426.Contig1\_All, Unigene7153\_All, Unigene32107\_All, CL13378.Contig2\_All, Unigene46442\_All, CL1021.Contig1\_All, Unigene41282\_All, CL2907.Contig3\_All, CL896.Contig25\_All, Unigene1982\_All, CL11257.Contig3\_All, Unigene5929\_All, CL1323.Contig1\_All, CL11257.Contig1\_All, CL11358.Contig2\_All, CL896.Contig26\_All, CL13596.Contig6\_All, CL2964.Contig3\_All, Unigene30962\_All, Unigene56276\_All, Unigene27024\_All, Unigene17052\_All, CL91.Contig6\_All, Unigene58085\_All, Unigene30448\_All, Unigene57248\_All, CL518.Contig2\_All, CL6178.Contig3\_All, CL10456.Contig2\_All, CL12484.Contig2\_All, Unigene6846\_All, Unigene58864\_All, CL12697.Contig2\_All, CL5300.Contig2\_All, CL3768.Contig2\_All, Unigene12371\_All, Unigene61177\_All, Unigene3674\_All, CL12624.Contig1\_All, CL2964.Contig8\_All, Unigene259\_All, Unigene14807\_All, Unigene55287\_All, CL896.Contig27\_All, Unigene14730\_All, CL896.Contig5\_All, CL10354.Contig1\_All, CL12484.Contig1\_All, Unigene33863\_All, CL5278.Contig2\_All, CL6985.Contig1\_All, CL9286.Contig3\_All, Unigene60300\_All, Unigene56733\_All, Unigene14004\_All, CL7522.Contig1\_All, CL2320.Contig2\_All |
| hydrolase activity, hydrolyzing O-glycosyl compounds | CL385.Contig7\_All, Unigene60460\_All, CL7143.Contig2\_All, Unigene16765\_All, CL385.Contig3\_All, CL4361.Contig1\_All, CL7842.Contig2\_All, CL633.Contig1\_All, CL13816.Contig2\_All, Unigene10807\_All, CL1405.Contig1\_All, CL3868.Contig4\_All, Unigene61637\_All, CL982.Contig3\_All, CL9423.Contig4\_All, Unigene57756\_All, CL3077.Contig1\_All, CL11516.Contig2\_All, Unigene56228\_All, CL10317.Contig1\_All, Unigene62369\_All, CL10693.Contig3\_All, CL385.Contig5\_All, CL13470.Contig3\_All, CL1405.Contig3\_All, CL13014.Contig1\_All, CL1962.Contig1\_All, Unigene29928\_All, Unigene37745\_All, Unigene4204\_All, CL5360.Contig4\_All, Unigene61176\_All, CL2168.Contig2\_All, CL10810.Contig1\_All, CL8912.Contig1\_All, Unigene21967\_All, CL5013.Contig2\_All, Unigene61508\_All, Unigene20553\_All, Unigene54925\_All, CL7143.Contig4\_All, CL9307.Contig1\_All, CL5325.Contig2\_All, CL4043.Contig4\_All, CL9662.Contig2\_All, CL4540.Contig10\_All, CL4361.Contig3\_All, Unigene37456\_All, Unigene13792\_All, Unigene61437\_All, Unigene60222\_All, Unigene15204\_All, Unigene26143\_All, Unigene55955\_All, CL973.Contig3\_All, Unigene2512\_All, Unigene9450\_All, CL4361.Contig2\_All, CL11636.Contig2\_All, CL3077.Contig2\_All, CL13014.Contig3\_All, Unigene20465\_All, CL9359.Contig3\_All, CL5013.Contig1\_All, Unigene14791\_All, Unigene18733\_All, CL4409.Contig1\_All, CL982.Contig1\_All, CL3381.Contig2\_All, CL6141.Contig10\_All, CL982.Contig4\_All, Unigene60259\_All, Unigene57944\_All, CL10693.Contig1\_All, Unigene23554\_All, CL2475.Contig2\_All, Unigene26792\_All, CL385.Contig1\_All, CL12823.Contig1\_All, CL10317.Contig2\_All, Unigene14060\_All, CL1913.Contig1\_All, Unigene19705\_All, Unigene33166\_All, CL4043.Contig3\_All, CL3160.Contig2\_All, CL12460.Contig1\_All, Unigene41339\_All, Unigene60714\_All, CL1962.Contig5\_All, CL3765.Contig1\_All, Unigene64775\_All, Unigene20369\_All, CL1842.Contig1\_All, CL385.Contig9\_All, CL9662.Contig1\_All, Unigene21171\_All, CL633.Contig2\_All, Unigene29236\_All, CL2604.Contig2\_All, CL3155.Contig3\_All, Unigene3543\_All, Unigene61217\_All, CL219.Contig1\_All, CL4118.Contig1\_All, Unigene20145\_All, Unigene40353\_All, Unigene26161\_All, Unigene6944\_All, Unigene20392\_All, Unigene30112\_All, Unigene15775\_All, CL1913.Contig2\_All, Unigene46659\_All, CL5360.Contig2\_All, CL5325.Contig1\_All, Unigene24573\_All, Unigene54520\_All, CL982.Contig6\_All, Unigene6838\_All, CL312.Contig2\_All, Unigene59369\_All, CL9359.Contig2\_All, Unigene58912\_All, CL2475.Contig1\_All, CL9610.Contig2\_All, Unigene20634\_All, CL3868.Contig2\_All, Unigene12770\_All, CL385.Contig6\_All, Unigene13736\_All, Unigene60074\_All, CL982.Contig5\_All, Unigene19891\_All, CL3280.Contig1\_All, Unigene57755\_All, CL10796.Contig2\_All, CL6141.Contig7\_All, Unigene64242\_All, CL3155.Contig1\_All, Unigene139\_All, Unigene32356\_All, CL8551.Contig3\_All, CL7143.Contig5\_All, Unigene26617\_All, CL9423.Contig1\_All, CL6982.Contig3\_All, CL8395.Contig2\_All, CL13816.Contig1\_All, Unigene62888\_All, Unigene30453\_All, CL10634.Contig1\_All, CL982.Contig2\_All, Unigene57314\_All, Unigene61418\_All, Unigene55413\_All, CL2457.Contig1\_All, Unigene4803\_All, Unigene65913\_All, Unigene602\_All, CL5990.Contig2\_All, CL3395.Contig2\_All, Unigene60999\_All, Unigene30006\_All, CL9293.Contig2\_All, Unigene60005\_All, Unigene61065\_All, CL5360.Contig3\_All, Unigene10260\_All, CL6982.Contig2\_All, Unigene58374\_All, CL6141.Contig9\_All, CL2674.Contig6\_All, CL257.Contig3\_All, Unigene6108\_All, CL5990.Contig1\_All, CL3381.Contig3\_All, CL3421.Contig1\_All, Unigene61439\_All, Unigene26454\_All, Unigene48334\_All, Unigene36083\_All, Unigene62965\_All, CL3868.Contig1\_All, CL8594.Contig2\_All |
| inositol 3-alpha-galactosyltransferase activity | Unigene8864\_All, CL2618.Contig2\_All, CL2618.Contig3\_All, Unigene27662\_All, Unigene22307\_All |
| integrase activity | Unigene35204\_All, Unigene63754\_All, Unigene65415\_All, Unigene37614\_All |
| glutamate synthase activity | Unigene26932\_All, CL2702.Contig2\_All, CL2702.Contig4\_All, Unigene702\_All, Unigene23920\_All, Unigene39960\_All, CL5832.Contig1\_All |
| alcohol dehydrogenase (NADP+) activity | CL9128.Contig2\_All, CL9128.Contig1\_All, CL3610.Contig2\_All, CL3610.Contig7\_All, Unigene43171\_All, CL3610.Contig5\_All, Unigene2266\_All, Unigene29396\_All, CL4483.Contig3\_All, CL4483.Contig1\_All, CL12503.Contig2\_All |
| aldo-keto reductase (NADP) activity | CL9128.Contig2\_All, Unigene9780\_All, CL9128.Contig1\_All, CL3610.Contig2\_All, Unigene43171\_All, CL7004.Contig1\_All, CL7004.Contig2\_All, Unigene46995\_All, CL12503.Contig2\_All, Unigene54710\_All, Unigene33964\_All, CL3610.Contig7\_All, Unigene31179\_All, CL3610.Contig5\_All, Unigene2266\_All, CL4067.Contig3\_All, CL11028.Contig1\_All, Unigene29396\_All, CL4483.Contig3\_All, CL4483.Contig1\_All |
| L-phenylalanine aminotransferase activity | CL514.Contig3\_All, CL142.Contig2\_All, CL2877.Contig1\_All, CL6717.Contig4\_All, CL12061.Contig2\_All, CL4718.Contig3\_All, CL889.Contig2\_All, Unigene38488\_All, Unigene4967\_All, CL10234.Contig1\_All, CL6717.Contig3\_All, Unigene22816\_All, CL10234.Contig2\_All, CL142.Contig1\_All, CL6717.Contig2\_All, CL12061.Contig3\_All, CL10823.Contig2\_All |
| L-tyrosine:2-oxoglutarate aminotransferase activity | CL10234.Contig2\_All, CL10234.Contig1\_All, CL142.Contig2\_All, CL2877.Contig1\_All, CL142.Contig1\_All, Unigene22816\_All |
| protein C-terminus binding | CL8673.Contig2\_All, CL12778.Contig1\_All, CL8673.Contig1\_All, Unigene41298\_All, CL5249.Contig1\_All, CL5249.Contig2\_All |
| glutamate synthase (NADH) activity | Unigene26932\_All, CL2702.Contig2\_All, CL2702.Contig4\_All, Unigene702\_All, Unigene23920\_All, CL5832.Contig1\_All |
| nicotianamine aminotransferase activity | CL10234.Contig2\_All, CL10234.Contig1\_All, CL142.Contig2\_All, CL2877.Contig1\_All, CL142.Contig1\_All, Unigene22816\_All |
| inositol oxygenase activity | Unigene25845\_All, CL10551.Contig1\_All, CL6865.Contig1\_All, Unigene21330\_All, CL10551.Contig2\_All, CL10551.Contig3\_All |
| copper ion binding | Unigene47188\_All, Unigene36238\_All, Unigene61740\_All, Unigene324\_All, Unigene61095\_All, Unigene16902\_All, CL5818.Contig3\_All, Unigene46439\_All, Unigene3362\_All, Unigene45891\_All, Unigene56878\_All, CL3536.Contig2\_All, Unigene29445\_All, Unigene877\_All, Unigene3468\_All, CL7715.Contig1\_All, CL810.Contig1\_All, CL12769.Contig2\_All, Unigene13148\_All, Unigene42184\_All, CL3683.Contig1\_All, CL514.Contig3\_All, CL4447.Contig1\_All, CL967.Contig6\_All, Unigene28965\_All, CL949.Contig1\_All, CL1531.Contig5\_All, CL1430.Contig11\_All, CL949.Contig3\_All, Unigene17142\_All, Unigene61476\_All, Unigene25738\_All, Unigene41235\_All, CL6808.Contig4\_All, CL2530.Contig2\_All, CL4011.Contig1\_All, Unigene56231\_All, Unigene57305\_All, CL12183.Contig2\_All, Unigene61511\_All, CL967.Contig7\_All, Unigene33589\_All, Unigene8225\_All, CL13973.Contig1\_All, CL9830.Contig2\_All, CL13573.Contig1\_All, Unigene42382\_All, CL1430.Contig6\_All, CL3277.Contig3\_All, CL2999.Contig1\_All, CL6808.Contig3\_All, Unigene2988\_All, CL9898.Contig2\_All, Unigene27753\_All, CL1430.Contig4\_All, Unigene14349\_All, CL11890.Contig1\_All, CL9898.Contig1\_All, Unigene54825\_All, Unigene60699\_All, CL5481.Contig2\_All, CL9937.Contig5\_All, CL1108.Contig3\_All, Unigene16699\_All, CL12350.Contig1\_All, CL7715.Contig3\_All, Unigene57432\_All, Unigene20743\_All, Unigene3798\_All, Unigene25507\_All, CL7892.Contig3\_All, CL4572.Contig1\_All, CL13964.Contig25\_All, Unigene14076\_All, Unigene42439\_All, CL12394.Contig2\_All, Unigene18987\_All, Unigene58669\_All, CL6842.Contig1\_All, CL3277.Contig1\_All, Unigene4175\_All, Unigene55119\_All, CL13020.Contig2\_All, CL5313.Contig3\_All, Unigene2134\_All, Unigene20716\_All, Unigene25657\_All, Unigene9754\_All, CL960.Contig2\_All, CL8349.Contig3\_All, CL810.Contig2\_All, CL6808.Contig5\_All, CL13517.Contig1\_All, CL9830.Contig3\_All, Unigene18781\_All, CL967.Contig8\_All, Unigene9305\_All, CL810.Contig4\_All, Unigene38927\_All, Unigene7376\_All, Unigene42744\_All, Unigene42390\_All, Unigene60069\_All, CL4447.Contig3\_All, Unigene19331\_All, Unigene17790\_All, Unigene32589\_All, Unigene60844\_All, Unigene54807\_All, Unigene26248\_All, CL4011.Contig2\_All, Unigene42120\_All, CL9531.Contig2\_All, CL4548.Contig1\_All, CL2530.Contig1\_All, CL3894.Contig3\_All, CL9830.Contig1\_All, CL98.Contig18\_All, CL9937.Contig1\_All, Unigene3614\_All, Unigene59884\_All, CL13573.Contig2\_All, Unigene36370\_All, CL7902.Contig1\_All, Unigene61490\_All, CL14006.Contig1\_All, CL4572.Contig4\_All, CL7892.Contig4\_All, Unigene33027\_All, Unigene4201\_All, CL3894.Contig1\_All, CL3329.Contig2\_All, CL13948.Contig3\_All, Unigene18418\_All, Unigene32232\_All, CL7649.Contig1\_All, Unigene18084\_All, Unigene57657\_All, CL8458.Contig1\_All, CL960.Contig1\_All, Unigene10747\_All, Unigene14167\_All, Unigene7449\_All, Unigene7127\_All, Unigene33797\_All, CL2907.Contig3\_All, Unigene63707\_All, Unigene41171\_All, CL9494.Contig5\_All, CL889.Contig2\_All, CL9898.Contig3\_All, CL12272.Contig1\_All, CL9937.Contig3\_All, Unigene39749\_All, CL8256.Contig1\_All, Unigene7531\_All, Unigene41723\_All, Unigene20287\_All, CL4447.Contig2\_All, Unigene17052\_All, CL2260.Contig2\_All, Unigene55217\_All, CL5818.Contig2\_All, Unigene41493\_All, CL9494.Contig2\_All, Unigene16800\_All, Unigene61007\_All, Unigene30619\_All, CL12350.Contig2\_All, CL3329.Contig1\_All, CL1430.Contig13\_All, CL3329.Contig5\_All, CL2882.Contig7\_All, Unigene39688\_All, CL6808.Contig2\_All, CL10298.Contig1\_All, CL3277.Contig2\_All, CL3890.Contig1\_All, Unigene22400\_All, CL9937.Contig2\_All, Unigene25851\_All, Unigene18784\_All, CL11744.Contig2\_All, CL8349.Contig1\_All, CL13312.Contig1\_All, Unigene6945\_All, Unigene140\_All, CL13989.Contig2\_All, Unigene33591\_All, CL2320.Contig3\_All, Unigene51332\_All |
| primary amine oxidase activity | Unigene9305\_All, CL5818.Contig3\_All, Unigene7376\_All, Unigene54807\_All, CL14006.Contig1\_All, Unigene7449\_All, CL13997.Contig1\_All, CL5818.Contig2\_All, Unigene41493\_All, CL13997.Contig3\_All |
| mannan synthase activity | CL1693.Contig2\_All, Unigene9786\_All, CL1693.Contig1\_All, Unigene23382\_All, CL11911.Contig2\_All, CL4539.Contig1\_All, CL7513.Contig2\_All, CL1693.Contig3\_All, Unigene58118\_All, CL7513.Contig1\_All |
| oxygen binding | CL2032.Contig4\_All, CL2032.Contig1\_All, Unigene30566\_All, Unigene29847\_All, Unigene55459\_All, CL5296.Contig5\_All, Unigene12838\_All, Unigene4339\_All, CL2032.Contig7\_All, CL4161.Contig4\_All, CL4161.Contig1\_All, Unigene29872\_All, Unigene54456\_All, CL9328.Contig1\_All, CL1045.Contig1\_All, CL2032.Contig5\_All, CL9711.Contig3\_All, Unigene3685\_All, Unigene22717\_All, CL8237.Contig2\_All, CL13917.Contig2\_All, CL4161.Contig2\_All, Unigene13893\_All, CL4161.Contig3\_All, Unigene65156\_All, CL12392.Contig1\_All, CL1817.Contig1\_All, CL2032.Contig2\_All |
| intramolecular oxidoreductase activity, transposing C=C bonds | CL8539.Contig2\_All, CL9060.Contig1\_All, CL2859.Contig2\_All, CL8539.Contig1\_All, CL6487.Contig3\_All, CL6726.Contig2\_All, CL6726.Contig1\_All |
| glutamate-ammonia ligase activity | Unigene10781\_All, CL4920.Contig4\_All, Unigene54986\_All, Unigene34041\_All, Unigene9659\_All, Unigene6689\_All, CL2450.Contig1\_All, Unigene6688\_All, Unigene6691\_All, Unigene20225\_All, Unigene6690\_All, Unigene6687\_All |
| beta-1,4-mannosyltransferase activity | CL1693.Contig2\_All, Unigene9786\_All, CL1693.Contig1\_All, Unigene23382\_All, CL11911.Contig2\_All, CL4539.Contig1\_All, CL7513.Contig2\_All, CL1693.Contig3\_All, Unigene58118\_All, CL7513.Contig1\_All |
| L-phenylalanine:2-oxoglutarate aminotransferase activity | CL514.Contig3\_All, CL142.Contig2\_All, CL2877.Contig1\_All, CL6717.Contig4\_All, CL12061.Contig2\_All, CL4718.Contig3\_All, CL889.Contig2\_All, Unigene38488\_All, CL10234.Contig1\_All, CL6717.Contig3\_All, Unigene22816\_All, CL10234.Contig2\_All, CL142.Contig1\_All, CL6717.Contig2\_All, CL12061.Contig3\_All, CL10823.Contig2\_All |
| transaminase activity | Unigene44241\_All, CL944.Contig3\_All, CL944.Contig2\_All, CL2999.Contig1\_All, CL944.Contig1\_All, CL6717.Contig4\_All, CL12061.Contig2\_All, CL4718.Contig3\_All, CL889.Contig2\_All, Unigene38488\_All, CL10234.Contig1\_All, CL10900.Contig2\_All, Unigene56757\_All, Unigene22816\_All, CL4148.Contig2\_All, Unigene14785\_All, Unigene60701\_All, Unigene47896\_All, Unigene39106\_All, CL10195.Contig1\_All, CL10234.Contig2\_All, CL2998.Contig1\_All, Unigene42476\_All, CL142.Contig1\_All, CL12061.Contig3\_All, CL13562.Contig2\_All, CL514.Contig3\_All, CL1131.Contig2\_All, CL6493.Contig1\_All, Unigene36388\_All, CL142.Contig2\_All, CL2877.Contig1\_All, Unigene38558\_All, Unigene40623\_All, Unigene14568\_All, CL2998.Contig3\_All, CL3890.Contig1\_All, Unigene4967\_All, CL6717.Contig3\_All, Unigene23445\_All, Unigene19806\_All, CL2998.Contig2\_All, Unigene33277\_All, Unigene20988\_All, Unigene16162\_All, Unigene23658\_All, CL6717.Contig2\_All, CL10823.Contig2\_All |
| transferase activity, transferring nitrogenous groups | Unigene44241\_All, CL944.Contig3\_All, CL944.Contig2\_All, CL2999.Contig1\_All, CL944.Contig1\_All, CL6717.Contig4\_All, CL12061.Contig2\_All, CL4718.Contig3\_All, CL889.Contig2\_All, Unigene38488\_All, CL10234.Contig1\_All, CL10900.Contig2\_All, Unigene56757\_All, Unigene22816\_All, CL4148.Contig2\_All, Unigene14785\_All, Unigene60701\_All, Unigene47896\_All, Unigene39106\_All, CL10195.Contig1\_All, CL10234.Contig2\_All, CL2998.Contig1\_All, Unigene42476\_All, CL142.Contig1\_All, CL12061.Contig3\_All, CL13562.Contig2\_All, CL514.Contig3\_All, CL1131.Contig2\_All, CL6493.Contig1\_All, Unigene36388\_All, CL142.Contig2\_All, CL2877.Contig1\_All, Unigene38558\_All, Unigene40623\_All, Unigene14568\_All, CL2998.Contig3\_All, CL3890.Contig1\_All, Unigene4967\_All, CL6717.Contig3\_All, Unigene23445\_All, Unigene19806\_All, CL2998.Contig2\_All, Unigene33277\_All, Unigene20988\_All, Unigene16162\_All, Unigene23658\_All, CL6717.Contig2\_All, CL10823.Contig2\_All |
| drug transmembrane transporter activity | Unigene7976\_All, Unigene56095\_All, CL8254.Contig2\_All, CL11788.Contig2\_All, CL11788.Contig1\_All, Unigene56646\_All, CL4077.Contig1\_All, CL13880.Contig1\_All, CL12679.Contig4\_All, Unigene18110\_All, CL2225.Contig2\_All, CL1946.Contig2\_All, CL8910.Contig2\_All, CL12679.Contig2\_All, CL10296.Contig2\_All, CL1946.Contig1\_All, Unigene56094\_All, CL12418.Contig1\_All, CL7499.Contig9\_All, Unigene16770\_All, CL2129.Contig2\_All, CL9027.Contig14\_All, CL8254.Contig1\_All, Unigene55696\_All, Unigene35383\_All, Unigene56928\_All, Unigene63864\_All, CL10764.Contig3\_All, Unigene41679\_All, Unigene55203\_All, CL3975.Contig2\_All, CL920.Contig2\_All, CL3922.Contig1\_All, CL3281.Contig1\_All, Unigene65301\_All, CL920.Contig3\_All, Unigene20789\_All, CL10352.Contig3\_All, Unigene56096\_All, Unigene56097\_All, Unigene13591\_All, CL3622.Contig2\_All, Unigene18751\_All, CL7499.Contig6\_All, CL4077.Contig2\_All, CL9027.Contig8\_All, CL2225.Contig1\_All, Unigene17765\_All, Unigene60226\_All |
| oxidoreductase activity, acting on paired donors, with incorporation or reduction of molecular oxygen, NAD(P)H as one donor, and incorporation of one atom of oxygen | CL1817.Contig5\_All, CL3333.Contig1\_All, CL2330.Contig2\_All, CL3261.Contig4\_All, Unigene57584\_All, CL3930.Contig4\_All, Unigene41646\_All, Unigene33962\_All, CL238.Contig33\_All, CL12628.Contig1\_All, CL9711.Contig3\_All, CL12184.Contig1\_All, Unigene60207\_All, CL683.Contig3\_All, Unigene5483\_All, Unigene59048\_All, Unigene34671\_All, CL4994.Contig9\_All, Unigene30566\_All, CL10224.Contig1\_All, CL683.Contig2\_All, Unigene29847\_All, CL238.Contig47\_All, CL3333.Contig2\_All, CL3507.Contig4\_All, CL238.Contig42\_All, Unigene20151\_All, Unigene29872\_All, CL10040.Contig2\_All, Unigene59047\_All, Unigene23512\_All, CL7843.Contig3\_All, CL3333.Contig4\_All, CL238.Contig57\_All, CL2233.Contig9\_All, CL238.Contig39\_All, Unigene44382\_All, CL3930.Contig5\_All, Unigene55841\_All, CL238.Contig40\_All, CL11627.Contig3\_All, CL2233.Contig3\_All, CL11627.Contig2\_All, CL12628.Contig2\_All, CL2587.Contig1\_All, Unigene13893\_All, CL1690.Contig1\_All, Unigene3061\_All, Unigene60002\_All, Unigene58425\_All, CL3930.Contig3\_All, CL1817.Contig1\_All, Unigene57585\_All |
| dodecenoyl-CoA delta-isomerase activity | CL9060.Contig1\_All, CL6487.Contig3\_All, CL6726.Contig2\_All, CL6726.Contig1\_All |
| intramolecular transferase activity, transferring amino groups | CL13562.Contig2\_All, Unigene14785\_All, Unigene47896\_All, Unigene16162\_All |
| histone serine kinase activity | CL12982.Contig1\_All, Unigene35757\_All, CL9134.Contig1\_All, Unigene57733\_All |
| histone kinase activity (H3-S10 specific) | CL12982.Contig1\_All, Unigene35757\_All, CL9134.Contig1\_All, Unigene57733\_All |
| glutamate-1-semialdehyde 2,1-aminomutase activity | CL13562.Contig2\_All, Unigene14785\_All, Unigene47896\_All, Unigene16162\_All |
| geranylgeranyl reductase activity | CL1491.Contig5\_All, CL1491.Contig3\_All, CL1491.Contig1\_All, CL1491.Contig2\_All |
| oxidoreductase activity, acting on CH-OH group of donors | Unigene21226\_All, Unigene27064\_All, CL3879.Contig1\_All, Unigene30214\_All, Unigene61740\_All, CL3596.Contig2\_All, Unigene24303\_All, Unigene11358\_All, CL8737.Contig1\_All, CL9651.Contig1\_All, Unigene39020\_All, CL3610.Contig7\_All, CL6062.Contig1\_All, CL4533.Contig3\_All, CL12769.Contig2\_All, CL4781.Contig2\_All, Unigene40786\_All, CL11925.Contig1\_All, CL2378.Contig1\_All, CL9128.Contig1\_All, CL12933.Contig1\_All, CL949.Contig1\_All, CL7390.Contig1\_All, CL949.Contig3\_All, Unigene54419\_All, CL7004.Contig2\_All, CL7072.Contig2\_All, CL2814.Contig4\_All, Unigene17142\_All, CL12503.Contig2\_All, Unigene63349\_All, CL6526.Contig1\_All, Unigene61476\_All, CL2912.Contig1\_All, CL737.Contig1\_All, CL5178.Contig5\_All, Unigene56231\_All, Unigene18514\_All, CL258.Contig2\_All, CL1053.Contig2\_All, CL3610.Contig5\_All, CL1232.Contig14\_All, CL1283.Contig4\_All, Unigene55739\_All, CL2814.Contig6\_All, Unigene56958\_All, CL8682.Contig2\_All, CL6022.Contig2\_All, CL6062.Contig3\_All, CL7298.Contig2\_All, CL1053.Contig1\_All, CL6022.Contig4\_All, CL12577.Contig2\_All, Unigene4039\_All, Unigene63761\_All, Unigene47014\_All, CL8682.Contig1\_All, Unigene14349\_All, CL4483.Contig2\_All, Unigene30252\_All, Unigene54418\_All, Unigene26135\_All, CL11865.Contig1\_All, CL5178.Contig4\_All, Unigene54422\_All, Unigene31267\_All, CL2120.Contig4\_All, CL8073.Contig1\_All, CL1232.Contig8\_All, CL11731.Contig1\_All, CL6775.Contig2\_All, Unigene59793\_All, Unigene6952\_All, CL5273.Contig1\_All, CL2814.Contig3\_All, Unigene9780\_All, CL3610.Contig2\_All, CL8001.Contig1\_All, Unigene58324\_All, Unigene10603\_All, CL5178.Contig2\_All, CL7072.Contig1\_All, CL1986.Contig2\_All, Unigene17192\_All, Unigene55119\_All, Unigene33964\_All, Unigene59773\_All, Unigene54321\_All, CL12933.Contig2\_All, Unigene33138\_All, Unigene42939\_All, CL2372.Contig1\_All, CL9973.Contig3\_All, CL1001.Contig3\_All, Unigene29396\_All, Unigene32479\_All, CL4067.Contig3\_All, Unigene25365\_All, CL3574.Contig5\_All, Unigene26365\_All, Unigene57130\_All, Unigene59471\_All, CL8737.Contig2\_All, Unigene60021\_All, CL7004.Contig1\_All, CL1232.Contig10\_All, CL6775.Contig1\_All, Unigene912\_All, Unigene44648\_All, CL12720.Contig2\_All, CL5185.Contig2\_All, Unigene12174\_All, CL6487.Contig3\_All, Unigene54710\_All, CL6869.Contig3\_All, CL7100.Contig3\_All, CL3894.Contig3\_All, Unigene59012\_All, CL5178.Contig6\_All, Unigene3383\_All, Unigene54412\_All, CL4483.Contig1\_All, Unigene14655\_All, CL3273.Contig1\_All, CL5500.Contig2\_All, CL2814.Contig5\_All, Unigene42870\_All, Unigene43171\_All, Unigene3652\_All, Unigene22442\_All, CL12972.Contig1\_All, CL1001.Contig2\_All, CL1207.Contig3\_All, Unigene60039\_All, CL10595.Contig1\_All, Unigene4665\_All, Unigene59694\_All, CL3894.Contig1\_All, CL12434.Contig3\_All, Unigene23051\_All, CL9651.Contig3\_All, CL10595.Contig3\_All, CL4483.Contig3\_All, Unigene37180\_All, Unigene14167\_All, CL12396.Contig1\_All, Unigene39970\_All, CL9128.Contig2\_All, Unigene47694\_All, Unigene55161\_All, Unigene3469\_All, Unigene1557\_All, Unigene46995\_All, CL9645.Contig1\_All, Unigene4398\_All, CL6526.Contig2\_All, Unigene58323\_All, Unigene31179\_All, CL2260.Contig2\_All, CL5178.Contig3\_All, Unigene2266\_All, CL1232.Contig6\_All, CL12769.Contig1\_All, CL11028.Contig1\_All, Unigene20050\_All, CL1283.Contig5\_All, Unigene41649\_All, Unigene61007\_All, CL9892.Contig3\_All, CL3443.Contig2\_All, Unigene54413\_All, CL6062.Contig2\_All, CL7100.Contig2\_All, Unigene33791\_All, CL3296.Contig1\_All, CL3283.Contig3\_All, Unigene55183\_All, Unigene39069\_All, CL13390.Contig2\_All, CL3925.Contig3\_All, CL5537.Contig2\_All, CL1698.Contig1\_All, Unigene61369\_All, CL12434.Contig2\_All, Unigene36124\_All, Unigene51332\_All, Unigene9281\_All |
| isocitrate dehydrogenase (NADP+) activity | CL3894.Contig1\_All, Unigene56231\_All, CL3894.Contig3\_All, CL12769.Contig1\_All, Unigene14349\_All, CL12769.Contig2\_All, Unigene25365\_All, Unigene17142\_All, Unigene14167\_All, Unigene51332\_All |
| ammonia ligase activity | Unigene10781\_All, CL4920.Contig4\_All, Unigene54986\_All, Unigene34041\_All, Unigene9659\_All, Unigene6689\_All, CL2450.Contig1\_All, Unigene6688\_All, CL6468.Contig1\_All, Unigene6691\_All, Unigene20225\_All, Unigene6690\_All, Unigene6687\_All |
| acid-ammonia (or amide) ligase activity | Unigene10781\_All, CL4920.Contig4\_All, Unigene54986\_All, Unigene34041\_All, Unigene9659\_All, Unigene6689\_All, CL2450.Contig1\_All, Unigene6688\_All, CL6468.Contig1\_All, Unigene6691\_All, Unigene20225\_All, Unigene6690\_All, Unigene6687\_All |
| (1->3)-beta-D-glucan binding | CL10110.Contig2\_All, Unigene58480\_All, CL10110.Contig1\_All |
| methylglutaconyl-CoA hydratase activity | CL6344.Contig3\_All, CL6344.Contig2\_All, CL6344.Contig1\_All |
| stearoyl-CoA 9-desaturase activity | CL8523.Contig1\_All, CL5008.Contig3\_All, CL5008.Contig1\_All |
| CMP-N-acetylneuraminate transmembrane transporter activity | CL410.Contig3\_All, CL410.Contig2\_All, CL410.Contig1\_All |
| sialic acid transmembrane transporter activity | CL410.Contig3\_All, CL410.Contig2\_All, CL410.Contig1\_All |
| acyl-CoA desaturase activity | CL8523.Contig1\_All, CL5008.Contig3\_All, CL5008.Contig1\_All |
| indoleacetamide hydrolase activity | Unigene55185\_All, Unigene55184\_All, Unigene55186\_All |
| 3-oxoacyl-[acyl-carrier-protein] reductase (NADPH) activity | CL4483.Contig2\_All, CL4483.Contig3\_All, Unigene60021\_All, CL12434.Contig3\_All, CL4483.Contig1\_All, CL12434.Contig2\_All, CL9651.Contig3\_All, CL9651.Contig1\_All |
| oxidoreductase activity, acting on the CH-NH2 group of donors, NAD or NADP as acceptor | Unigene26932\_All, Unigene702\_All, Unigene23920\_All, Unigene19331\_All, CL2702.Contig4\_All, CL2702.Contig2\_All, CL5832.Contig1\_All, Unigene39960\_All |
| hydrolase activity, acting on glycosyl bonds | CL5381.Contig1\_All, CL385.Contig7\_All, Unigene15168\_All, Unigene60460\_All, CL7143.Contig2\_All, Unigene16765\_All, CL385.Contig3\_All, CL4361.Contig1\_All, CL7842.Contig2\_All, CL633.Contig1\_All, CL13816.Contig2\_All, Unigene10807\_All, CL1405.Contig1\_All, Unigene57799\_All, CL3868.Contig4\_All, Unigene61637\_All, CL982.Contig3\_All, Unigene28522\_All, CL9423.Contig4\_All, Unigene57756\_All, CL3077.Contig1\_All, CL11516.Contig2\_All, Unigene56228\_All, CL10317.Contig1\_All, Unigene62369\_All, CL10693.Contig3\_All, CL385.Contig5\_All, CL13470.Contig3\_All, CL1405.Contig3\_All, Unigene7305\_All, CL13014.Contig1\_All, CL1962.Contig1\_All, Unigene29928\_All, Unigene37745\_All, Unigene4204\_All, CL5360.Contig4\_All, Unigene61176\_All, Unigene61133\_All, CL2168.Contig2\_All, CL10810.Contig1\_All, CL8912.Contig1\_All, Unigene21967\_All, CL5013.Contig2\_All, Unigene61508\_All, Unigene20553\_All, CL8592.Contig1\_All, Unigene16758\_All, Unigene54925\_All, CL7143.Contig4\_All, CL9307.Contig1\_All, CL8592.Contig2\_All, CL5325.Contig2\_All, CL4043.Contig4\_All, CL9662.Contig2\_All, CL4540.Contig10\_All, CL4361.Contig3\_All, Unigene37456\_All, Unigene13792\_All, Unigene61437\_All, Unigene60222\_All, Unigene15204\_All, Unigene26143\_All, Unigene55955\_All, CL973.Contig3\_All, Unigene2512\_All, Unigene9450\_All, CL4361.Contig2\_All, CL11636.Contig2\_All, CL3077.Contig2\_All, CL13014.Contig3\_All, Unigene20465\_All, CL9359.Contig3\_All, CL5013.Contig1\_All, Unigene14791\_All, Unigene18733\_All, CL4409.Contig1\_All, CL982.Contig1\_All, CL3381.Contig2\_All, CL6141.Contig10\_All, CL982.Contig4\_All, Unigene60259\_All, Unigene57944\_All, CL10693.Contig1\_All, Unigene23554\_All, CL2475.Contig2\_All, Unigene26792\_All, CL385.Contig1\_All, CL12823.Contig1\_All, CL10317.Contig2\_All, Unigene14060\_All, CL1913.Contig1\_All, Unigene19705\_All, Unigene33166\_All, CL4043.Contig3\_All, CL3160.Contig2\_All, CL12460.Contig1\_All, Unigene41339\_All, Unigene60714\_All, CL1962.Contig5\_All, CL3765.Contig1\_All, Unigene64775\_All, CL5446.Contig3\_All, Unigene20369\_All, CL1842.Contig1\_All, CL385.Contig9\_All, CL9662.Contig1\_All, Unigene21171\_All, CL633.Contig2\_All, Unigene29236\_All, CL2604.Contig2\_All, CL3155.Contig3\_All, Unigene3543\_All, Unigene61217\_All, CL219.Contig1\_All, Unigene20145\_All, CL4118.Contig1\_All, Unigene3022\_All, Unigene40353\_All, Unigene26161\_All, Unigene6944\_All, Unigene20392\_All, Unigene30112\_All, Unigene15775\_All, CL1913.Contig2\_All, Unigene46659\_All, CL5360.Contig2\_All, CL5325.Contig1\_All, Unigene24573\_All, Unigene54520\_All, CL982.Contig6\_All, Unigene6838\_All, CL312.Contig2\_All, Unigene59369\_All, CL9359.Contig2\_All, Unigene58912\_All, CL2475.Contig1\_All, Unigene42383\_All, CL9610.Contig2\_All, Unigene64838\_All, Unigene20634\_All, CL3868.Contig2\_All, Unigene27762\_All, Unigene12770\_All, Unigene13736\_All, CL385.Contig6\_All, Unigene60074\_All, CL982.Contig5\_All, Unigene19891\_All, CL3280.Contig1\_All, Unigene57755\_All, CL10796.Contig2\_All, CL6141.Contig7\_All, Unigene64242\_All, CL3155.Contig1\_All, Unigene139\_All, Unigene32356\_All, CL8551.Contig3\_All, CL7143.Contig5\_All, Unigene26617\_All, CL9423.Contig1\_All, Unigene34308\_All, CL6982.Contig3\_All, CL8395.Contig2\_All, CL13816.Contig1\_All, Unigene62888\_All, Unigene30453\_All, Unigene16561\_All, CL10634.Contig1\_All, CL982.Contig2\_All, Unigene57314\_All, Unigene61418\_All, Unigene55413\_All, CL2457.Contig1\_All, Unigene4803\_All, Unigene65913\_All, Unigene602\_All, CL5990.Contig2\_All, CL3395.Contig2\_All, Unigene60999\_All, Unigene30006\_All, CL9293.Contig2\_All, Unigene60005\_All, Unigene61065\_All, CL5360.Contig3\_All, Unigene11105\_All, Unigene10260\_All, Unigene56227\_All, CL6982.Contig2\_All, Unigene58374\_All, CL6141.Contig9\_All, CL257.Contig3\_All, CL2674.Contig6\_All, Unigene6108\_All, CL5990.Contig1\_All, CL3381.Contig3\_All, CL3421.Contig1\_All, Unigene61439\_All, Unigene26454\_All, Unigene48334\_All, Unigene36083\_All, Unigene62965\_All, CL3868.Contig1\_All, CL8594.Contig2\_All |
| aspartic-type peptidase activity | Unigene14110\_All, Unigene61253\_All, Unigene34621\_All, CL13776.Contig1\_All, CL6648.Contig2\_All, CL11287.Contig1\_All, CL1844.Contig1\_All, Unigene26822\_All, CL6683.Contig1\_All, CL5667.Contig1\_All, CL6648.Contig1\_All, Unigene63754\_All, CL1184.Contig5\_All, CL5001.Contig3\_All, CL3294.Contig2\_All, Unigene35204\_All, Unigene19964\_All, Unigene37614\_All, Unigene20846\_All, CL12617.Contig3\_All, Unigene63445\_All, Unigene58440\_All, Unigene3714\_All, CL13776.Contig2\_All, CL5001.Contig5\_All, Unigene14163\_All, CL11287.Contig2\_All, Unigene65415\_All, Unigene22918\_All, Unigene22861\_All |
| translation elongation factor activity | Unigene23394\_All, CL2907.Contig3\_All, Unigene33797\_All, Unigene16902\_All, CL13861.Contig5\_All, Unigene40080\_All, Unigene42887\_All, Unigene56323\_All, Unigene60069\_All, Unigene30945\_All, Unigene14583\_All, Unigene60740\_All, Unigene56196\_All, CL4186.Contig2\_All, CL13379.Contig2\_All, Unigene30740\_All, CL7737.Contig1\_All, Unigene42950\_All, CL7231.Contig1\_All, Unigene27024\_All, CL13378.Contig1\_All, Unigene24620\_All, CL9323.Contig2\_All, CL9286.Contig1\_All, CL2782.Contig1\_All, Unigene43054\_All, Unigene30448\_All, Unigene57248\_All, Unigene3973\_All, Unigene23393\_All, CL2782.Contig2\_All, Unigene6846\_All, CL12484.Contig2\_All, Unigene59157\_All, Unigene12371\_All, Unigene38355\_All, Unigene43514\_All, Unigene8835\_All, CL13641.Contig3\_All, Unigene61490\_All, Unigene29969\_All, CL13641.Contig1\_All, Unigene56324\_All, Unigene14807\_All, Unigene1316\_All, CL9286.Contig2\_All, Unigene14730\_All, CL8349.Contig1\_All, Unigene6711\_All, Unigene61511\_All, CL5000.Contig1\_All, CL7231.Contig2\_All, CL10354.Contig1\_All, CL12484.Contig1\_All, Unigene28581\_All, CL8349.Contig3\_All, Unigene19707\_All, CL13379.Contig1\_All, CL9286.Contig3\_All, CL13973.Contig1\_All, Unigene32107\_All, CL13378.Contig2\_All, Unigene41282\_All, CL2320.Contig2\_All |
| chitinase activity | CL219.Contig1\_All, CL7143.Contig5\_All, CL7143.Contig4\_All, Unigene4803\_All, Unigene2512\_All, CL7143.Contig2\_All, CL13816.Contig1\_All, CL13014.Contig3\_All, CL13014.Contig1\_All, CL13816.Contig2\_All |
| rRNA binding | CL7564.Contig1\_All, Unigene23149\_All, CL1339.Contig4\_All, Unigene54729\_All, Unigene22228\_All, CL2048.Contig4\_All, Unigene23043\_All, CL4701.Contig2\_All, CL3583.Contig2\_All, CL1339.Contig5\_All, Unigene42315\_All, Unigene8052\_All, Unigene4385\_All, CL10105.Contig1\_All, Unigene38556\_All, CL854.Contig4\_All, CL4661.Contig2\_All, CL9824.Contig2\_All, CL116.Contig3\_All, Unigene19923\_All, Unigene6077\_All, Unigene24742\_All, CL386.Contig3\_All, Unigene34402\_All, CL386.Contig1\_All, Unigene6078\_All, Unigene7670\_All, CL9824.Contig1\_All, Unigene16255\_All, Unigene10138\_All, Unigene24550\_All, Unigene15283\_All, Unigene60256\_All, Unigene13493\_All, CL5555.Contig2\_All, CL386.Contig2\_All, Unigene14921\_All, CL2586.Contig1\_All, Unigene43305\_All, Unigene28704\_All, CL6335.Contig1\_All, CL1438.Contig2\_All, CL2586.Contig2\_All, Unigene15519\_All, CL12599.Contig1\_All, Unigene42825\_All, CL854.Contig3\_All, CL4661.Contig3\_All, CL11803.Contig2\_All, CL3583.Contig1\_All, Unigene17239\_All, CL4701.Contig1\_All, CL8252.Contig1\_All, CL12178.Contig1\_All, Unigene37315\_All, CL2048.Contig3\_All, CL461.Contig3\_All, Unigene32598\_All, Unigene46996\_All, CL2048.Contig2\_All |
| oxygen transporter activity | CL4161.Contig2\_All, CL4161.Contig4\_All, CL4161.Contig1\_All, CL4161.Contig3\_All, Unigene22717\_All, CL13917.Contig2\_All |
| anion:cation symporter activity | CL9614.Contig1\_All, CL7188.Contig1\_All, CL10085.Contig3\_All, Unigene63073\_All, CL10085.Contig2\_All, Unigene55976\_All |
| secondary active transmembrane transporter activity | Unigene31375\_All, CL6859.Contig2\_All, Unigene3362\_All, CL11382.Contig2\_All, CL10864.Contig2\_All, CL3689.Contig6\_All, Unigene60373\_All, CL13880.Contig1\_All, CL2436.Contig2\_All, CL1205.Contig6\_All, CL3986.Contig1\_All, CL12679.Contig2\_All, Unigene34564\_All, CL2965.Contig2\_All, CL12418.Contig1\_All, Unigene29887\_All, Unigene55696\_All, CL1876.Contig1\_All, Unigene16336\_All, Unigene34172\_All, Unigene59478\_All, Unigene28965\_All, Unigene61494\_All, CL6796.Contig2\_All, CL920.Contig2\_All, CL10864.Contig1\_All, CL3281.Contig1\_All, Unigene40544\_All, Unigene26098\_All, CL5771.Contig3\_All, Unigene22297\_All, CL9444.Contig2\_All, Unigene56097\_All, CL10085.Contig3\_All, Unigene22762\_All, CL3074.Contig2\_All, CL8933.Contig1\_All, CL11788.Contig2\_All, CL12925.Contig3\_All, Unigene16763\_All, CL1946.Contig1\_All, Unigene56094\_All, CL7581.Contig3\_All, Unigene63073\_All, CL3689.Contig4\_All, CL7499.Contig9\_All, Unigene60257\_All, CL2702.Contig4\_All, CL12466.Contig4\_All, CL10764.Contig3\_All, Unigene55976\_All, CL1760.Contig3\_All, Unigene55233\_All, Unigene20755\_All, CL588.Contig4\_All, CL604.Contig1\_All, Unigene4175\_All, CL10352.Contig3\_All, Unigene15535\_All, Unigene57960\_All, Unigene56096\_All, Unigene45125\_All, CL12936.Contig3\_All, Unigene14619\_All, CL7499.Contig6\_All, Unigene59384\_All, CL7413.Contig1\_All, CL10085.Contig2\_All, CL8933.Contig2\_All, Unigene35631\_All, Unigene34157\_All, CL11788.Contig1\_All, Unigene3687\_All, Unigene4205\_All, CL2225.Contig2\_All, Unigene26932\_All, CL410.Contig3\_All, CL4472.Contig1\_All, CL9027.Contig14\_All, CL8254.Contig1\_All, CL604.Contig7\_All, CL9856.Contig2\_All, Unigene28694\_All, CL817.Contig4\_All, CL14006.Contig1\_All, Unigene30500\_All, CL3922.Contig1\_All, CL2035.Contig1\_All, Unigene65301\_All, CL3285.Contig20\_All, Unigene60014\_All, CL206.Contig1\_All, Unigene13591\_All, CL2288.Contig1\_All, CL7413.Contig2\_All, CL410.Contig1\_All, Unigene18751\_All, CL4725.Contig2\_All, CL4077.Contig2\_All, CL3986.Contig2\_All, Unigene60226\_All, CL7188.Contig1\_All, Unigene56095\_All, CL8464.Contig1\_All, CL8254.Contig2\_All, Unigene26229\_All, CL4077.Contig1\_All, Unigene18110\_All, CL12679.Contig4\_All, CL1946.Contig2\_All, Unigene16784\_All, Unigene41405\_All, CL8446.Contig2\_All, CL4937.Contig2\_All, Unigene18748\_All, Unigene932\_All, Unigene29618\_All, CL7581.Contig1\_All, Unigene43692\_All, Unigene20393\_All, Unigene30020\_All, CL9614.Contig1\_All, CL12548.Contig1\_All, CL2702.Contig2\_All, Unigene22965\_All, Unigene56928\_All, CL11361.Contig1\_All, CL920.Contig3\_All, Unigene18860\_All, CL12466.Contig2\_All, CL12548.Contig2\_All, CL9819.Contig2\_All, CL2561.Contig2\_All, CL6859.Contig1\_All, CL3986.Contig4\_All, CL2225.Contig1\_All, CL9027.Contig8\_All, CL410.Contig2\_All, Unigene59589\_All, CL11361.Contig2\_All |
| xyloglucan:xyloglucosyl transferase activity | Unigene14060\_All, CL10693.Contig3\_All, Unigene26143\_All, Unigene33166\_All, Unigene602\_All, CL3765.Contig1\_All, CL3381.Contig2\_All, Unigene15775\_All, Unigene60259\_All, CL10693.Contig1\_All, CL9662.Contig1\_All, CL3381.Contig3\_All, CL9662.Contig2\_All, CL10317.Contig1\_All, CL2604.Contig2\_All, Unigene58912\_All, CL10317.Contig2\_All |
| drug transporter activity | Unigene7976\_All, Unigene56095\_All, CL8254.Contig2\_All, CL11788.Contig2\_All, CL11788.Contig1\_All, Unigene56646\_All, CL4077.Contig1\_All, CL13880.Contig1\_All, CL12679.Contig4\_All, Unigene18110\_All, CL2225.Contig2\_All, CL1946.Contig2\_All, CL8910.Contig2\_All, CL12679.Contig2\_All, CL10296.Contig2\_All, CL1946.Contig1\_All, Unigene56094\_All, CL12418.Contig1\_All, CL7499.Contig9\_All, Unigene16770\_All, CL2129.Contig2\_All, CL9027.Contig14\_All, CL8254.Contig1\_All, Unigene55696\_All, Unigene35383\_All, Unigene56928\_All, Unigene63864\_All, CL10764.Contig3\_All, Unigene41679\_All, Unigene55203\_All, CL3975.Contig2\_All, CL920.Contig2\_All, CL3922.Contig1\_All, CL3281.Contig1\_All, Unigene65301\_All, CL920.Contig3\_All, Unigene20789\_All, CL10352.Contig3\_All, Unigene56096\_All, Unigene56097\_All, Unigene13591\_All, CL3622.Contig2\_All, Unigene18751\_All, CL7499.Contig6\_All, CL4077.Contig2\_All, CL9027.Contig8\_All, CL2225.Contig1\_All, Unigene17765\_All, Unigene60226\_All |
| oxidoreductase activity, acting on the CH-CH group of donors, NAD or NADP as acceptor | CL2228.Contig5\_All, CL9639.Contig2\_All, CL5167.Contig1\_All, CL649.Contig1\_All, CL8132.Contig1\_All, CL6201.Contig2\_All, Unigene15306\_All, Unigene63665\_All, Unigene7551\_All, Unigene20235\_All, CL6730.Contig3\_All, Unigene8722\_All, CL7971.Contig3\_All, Unigene14805\_All, CL1491.Contig3\_All, CL4609.Contig3\_All, CL479.Contig5\_All, CL5167.Contig5\_All, Unigene59478\_All, CL9395.Contig1\_All, CL6177.Contig2\_All, CL1195.Contig3\_All, CL1375.Contig1\_All, Unigene565\_All, Unigene41308\_All, CL7971.Contig2\_All, Unigene56097\_All, Unigene39526\_All, CL7447.Contig1\_All, CL4656.Contig1\_All, CL609.Contig49\_All, Unigene57674\_All, CL109.Contig7\_All, CL9152.Contig2\_All, Unigene10664\_All, CL109.Contig4\_All, Unigene15416\_All, CL10267.Contig1\_All, Unigene26344\_All, CL10396.Contig2\_All, CL3807.Contig3\_All, CL109.Contig6\_All, CL12729.Contig1\_All, CL8799.Contig2\_All, Unigene3321\_All, Unigene4846\_All, Unigene39790\_All, CL7061.Contig3\_All, CL6289.Contig2\_All, CL1491.Contig5\_All, CL8493.Contig4\_All, CL5309.Contig1\_All, Unigene56094\_All, Unigene12613\_All, CL4773.Contig2\_All, Unigene13422\_All, Unigene16775\_All, CL12376.Contig2\_All, Unigene13446\_All, CL7310.Contig1\_All, CL3405.Contig5\_All, Unigene54999\_All, Unigene61025\_All, CL4982.Contig2\_All, CL10427.Contig4\_All, CL12466.Contig4\_All, CL9639.Contig1\_All, Unigene4144\_All, CL8799.Contig1\_All, Unigene33156\_All, CL6136.Contig2\_All, CL6289.Contig1\_All, Unigene15535\_All, Unigene24554\_All, CL5146.Contig3\_All, Unigene56096\_All, CL5710.Contig2\_All, Unigene24435\_All, Unigene13776\_All, CL211.Contig3\_All, Unigene4259\_All, CL10396.Contig1\_All, CL6804.Contig2\_All, CL109.Contig5\_All, Unigene20850\_All, CL6201.Contig3\_All, CL609.Contig45\_All, CL11965.Contig3\_All, Unigene29846\_All, CL6117.Contig2\_All, CL12810.Contig1\_All, Unigene17130\_All, CL603.Contig5\_All, Unigene4205\_All, Unigene57139\_All, CL2225.Contig2\_All, Unigene12455\_All, CL1326.Contig2\_All, CL6201.Contig4\_All, CL6434.Contig4\_All, CL12376.Contig1\_All, Unigene16753\_All, Unigene55745\_All, CL2517.Contig9\_All, Unigene2390\_All, CL6730.Contig2\_All, CL379.Contig1\_All, CL1442.Contig5\_All, Unigene60007\_All, CL1195.Contig2\_All, CL109.Contig2\_All, CL479.Contig4\_All, Unigene13789\_All, CL3038.Contig1\_All, CL8258.Contig1\_All, CL1491.Contig2\_All, CL12942.Contig1\_All, CL603.Contig1\_All, Unigene28755\_All, Unigene30804\_All, CL2711.Contig1\_All, Unigene3737\_All, Unigene56997\_All, Unigene60014\_All, CL772.Contig2\_All, Unigene6066\_All, Unigene15289\_All, CL7818.Contig5\_All, Unigene62417\_All, CL4725.Contig2\_All, CL4773.Contig1\_All, Unigene57038\_All, Unigene60226\_All, CL1203.Contig4\_All, CL9494.Contig5\_All, Unigene58820\_All, Unigene56095\_All, CL3991.Contig2\_All, CL7061.Contig4\_All, CL8464.Contig1\_All, Unigene60254\_All, CL479.Contig2\_All, CL5146.Contig4\_All, Unigene41323\_All, CL7818.Contig6\_All, Unigene38732\_All, CL8446.Contig2\_All, Unigene20086\_All, Unigene38704\_All, Unigene13603\_All, CL2710.Contig2\_All, Unigene58150\_All, CL13433.Contig1\_All, CL9494.Contig2\_All, CL6117.Contig1\_All, CL6201.Contig1\_All, Unigene19285\_All, Unigene59421\_All, CL12964.Contig1\_All, CL1491.Contig1\_All, Unigene57352\_All, Unigene1034\_All, CL11965.Contig4\_All, CL2710.Contig1\_All, CL109.Contig1\_All, Unigene33749\_All, CL2200.Contig2\_All, CL8258.Contig2\_All, CL12466.Contig2\_All, Unigene105\_All, Unigene30105\_All, CL2225.Contig1\_All, Unigene17182\_All, Unigene17424\_All, CL11361.Contig2\_All, CL5823.Contig1\_All |
| galactosyltransferase activity | CL7693.Contig1\_All, CL5140.Contig2\_All, Unigene35304\_All, Unigene16399\_All, CL2618.Contig2\_All, Unigene42368\_All, CL7693.Contig3\_All, CL4521.Contig1\_All, Unigene27662\_All, Unigene22307\_All, Unigene25173\_All, CL271.Contig2\_All, Unigene7123\_All, CL2618.Contig3\_All, Unigene5078\_All, CL1842.Contig1\_All, Unigene19918\_All, CL271.Contig1\_All, Unigene42163\_All, CL8912.Contig1\_All, Unigene8864\_All, CL8395.Contig2\_All, Unigene27246\_All, CL13764.Contig1\_All |
| isocitrate dehydrogenase activity | CL3894.Contig1\_All, Unigene56231\_All, Unigene4039\_All, CL3894.Contig3\_All, CL7390.Contig1\_All, Unigene14349\_All, CL12769.Contig1\_All, CL12769.Contig2\_All, Unigene25365\_All, Unigene17142\_All, CL4781.Contig2\_All, Unigene14167\_All, Unigene51332\_All |
| NADPH:quinone reductase activity | CL7712.Contig1\_All, Unigene60317\_All, CL7712.Contig5\_All, Unigene62303\_All, CL2221.Contig4\_All, CL7072.Contig1\_All, CL10399.Contig2\_All, CL7072.Contig2\_All, CL1375.Contig1\_All, CL7712.Contig2\_All |
| oxidoreductase activity, acting on the CH-CH group of donors | CL2228.Contig5\_All, CL9639.Contig2\_All, CL5167.Contig1\_All, CL649.Contig1\_All, CL8132.Contig1\_All, CL6201.Contig2\_All, Unigene15306\_All, Unigene63665\_All, Unigene29585\_All, Unigene7551\_All, Unigene20235\_All, CL6730.Contig3\_All, Unigene8722\_All, CL7971.Contig3\_All, Unigene14805\_All, CL1491.Contig3\_All, CL4609.Contig3\_All, CL479.Contig5\_All, CL5167.Contig5\_All, CL7457.Contig2\_All, Unigene59478\_All, Unigene10156\_All, CL9395.Contig1\_All, CL6177.Contig2\_All, CL1195.Contig3\_All, CL11051.Contig2\_All, CL1375.Contig1\_All, Unigene565\_All, Unigene41308\_All, Unigene34388\_All, CL7971.Contig2\_All, Unigene56097\_All, Unigene39526\_All, CL7447.Contig1\_All, CL4656.Contig1\_All, CL609.Contig49\_All, Unigene57674\_All, CL109.Contig7\_All, CL9152.Contig2\_All, Unigene10664\_All, CL109.Contig4\_All, Unigene15416\_All, CL10267.Contig1\_All, Unigene26344\_All, CL10396.Contig2\_All, CL3807.Contig3\_All, CL109.Contig6\_All, CL12729.Contig1\_All, CL8799.Contig2\_All, CL5672.Contig3\_All, Unigene3321\_All, CL9751.Contig2\_All, Unigene4846\_All, Unigene39790\_All, CL11418.Contig2\_All, CL7061.Contig3\_All, Unigene40247\_All, CL6289.Contig2\_All, CL1491.Contig5\_All, CL8493.Contig4\_All, CL5309.Contig1\_All, Unigene56094\_All, Unigene12613\_All, CL4773.Contig2\_All, Unigene13422\_All, Unigene16775\_All, CL12376.Contig2\_All, Unigene13446\_All, CL7310.Contig1\_All, CL3405.Contig5\_All, Unigene54999\_All, Unigene61025\_All, CL4982.Contig2\_All, CL10427.Contig4\_All, Unigene12103\_All, CL12466.Contig4\_All, CL9639.Contig1\_All, Unigene4144\_All, CL8799.Contig1\_All, CL8389.Contig3\_All, Unigene33156\_All, CL288.Contig1\_All, CL6136.Contig2\_All, CL6289.Contig1\_All, Unigene36489\_All, Unigene15535\_All, Unigene24554\_All, CL5146.Contig3\_All, Unigene56096\_All, CL5710.Contig2\_All, Unigene24435\_All, Unigene13776\_All, CL211.Contig3\_All, Unigene4259\_All, CL10396.Contig1\_All, CL6804.Contig2\_All, CL109.Contig5\_All, Unigene20850\_All, CL6201.Contig3\_All, CL609.Contig45\_All, CL11965.Contig3\_All, CL609.Contig50\_All, Unigene29846\_All, CL6117.Contig2\_All, CL12810.Contig1\_All, Unigene17130\_All, CL603.Contig5\_All, Unigene4205\_All, Unigene57139\_All, CL2225.Contig2\_All, Unigene12455\_All, CL12376.Contig1\_All, CL6434.Contig4\_All, CL1326.Contig2\_All, CL6201.Contig4\_All, Unigene16753\_All, Unigene55745\_All, CL196.Contig1\_All, Unigene2390\_All, CL2517.Contig9\_All, CL6730.Contig2\_All, CL379.Contig1\_All, CL1442.Contig5\_All, Unigene60007\_All, CL1195.Contig2\_All, CL109.Contig2\_All, CL479.Contig4\_All, CL11418.Contig1\_All, Unigene13789\_All, Unigene40545\_All, CL3038.Contig1\_All, CL8258.Contig1\_All, CL1491.Contig2\_All, CL12942.Contig1\_All, CL603.Contig1\_All, Unigene28755\_All, Unigene30804\_All, CL2711.Contig1\_All, Unigene3737\_All, Unigene56997\_All, Unigene60014\_All, CL5672.Contig1\_All, CL772.Contig2\_All, Unigene6066\_All, Unigene15289\_All, CL7818.Contig5\_All, Unigene62417\_All, CL9572.Contig2\_All, CL4725.Contig2\_All, CL4773.Contig1\_All, Unigene57038\_All, Unigene60226\_All, CL1203.Contig4\_All, CL9494.Contig5\_All, Unigene58820\_All, CL11392.Contig2\_All, Unigene56095\_All, CL3991.Contig2\_All, CL7061.Contig4\_All, CL8464.Contig1\_All, Unigene60254\_All, CL479.Contig2\_All, CL5146.Contig4\_All, Unigene25097\_All, Unigene41323\_All, CL9572.Contig1\_All, CL7818.Contig6\_All, Unigene38732\_All, CL8446.Contig2\_All, Unigene20086\_All, CL923.Contig4\_All, CL8389.Contig1\_All, Unigene38704\_All, Unigene13603\_All, CL2710.Contig2\_All, Unigene58150\_All, CL2583.Contig1\_All, CL923.Contig3\_All, CL13433.Contig1\_All, CL9494.Contig2\_All, CL6117.Contig1\_All, CL5672.Contig2\_All, CL6201.Contig1\_All, Unigene19285\_All, Unigene59421\_All, CL11002.Contig2\_All, CL12964.Contig1\_All, CL1491.Contig1\_All, Unigene57352\_All, CL11002.Contig1\_All, Unigene1034\_All, CL11965.Contig4\_All, CL2710.Contig1\_All, CL109.Contig1\_All, Unigene35224\_All, CL11392.Contig1\_All, Unigene33749\_All, CL1016.Contig2\_All, CL2200.Contig2\_All, Unigene58406\_All, CL8258.Contig2\_All, CL12466.Contig2\_All, Unigene105\_All, Unigene30105\_All, CL2225.Contig1\_All, Unigene17182\_All, Unigene17424\_All, CL11361.Contig2\_All, CL5823.Contig1\_All |
| carboxylic acid binding | Unigene54840\_All, CL2588.Contig1\_All, CL12021.Contig2\_All, Unigene23332\_All, CL10607.Contig3\_All, CL1413.Contig1\_All, Unigene59749\_All, CL13868.Contig8\_All, Unigene59748\_All, CL12446.Contig7\_All, CL9639.Contig3\_All, CL171.Contig16\_All, Unigene40795\_All, CL201.Contig17\_All, CL2688.Contig1\_All, Unigene23030\_All, Unigene17682\_All, CL4175.Contig2\_All, CL2588.Contig2\_All, CL8770.Contig4\_All, Unigene17983\_All, Unigene35538\_All, Unigene5566\_All, CL2688.Contig2\_All, CL1895.Contig13\_All, CL12933.Contig1\_All, CL12972.Contig1\_All, CL12416.Contig1\_All, CL12021.Contig3\_All, Unigene23378\_All, Unigene54842\_All, CL171.Contig18\_All, CL9500.Contig2\_All, Unigene59750\_All, Unigene24898\_All, Unigene28693\_All, CL9717.Contig1\_All, CL12933.Contig2\_All, Unigene13760\_All, CL9967.Contig1\_All, CL171.Contig17\_All, CL201.Contig16\_All, CL13988.Contig2\_All, CL1895.Contig14\_All, CL171.Contig19\_All, Unigene29022\_All, Unigene54841\_All, Unigene36014\_All, CL2862.Contig1\_All, CL1895.Contig15\_All |
| oxidoreductase activity, acting on paired donors, with incorporation or reduction of molecular oxygen | CL2032.Contig1\_All, Unigene12838\_All, CL3930.Contig1\_All, Unigene13680\_All, CL3930.Contig4\_All, CL7484.Contig2\_All, CL9328.Contig1\_All, Unigene7551\_All, Unigene40795\_All, CL12184.Contig1\_All, Unigene38900\_All, CL13995.Contig1\_All, CL5008.Contig1\_All, CL2588.Contig2\_All, Unigene34671\_All, CL4994.Contig9\_All, CL9578.Contig2\_All, CL3333.Contig2\_All, CL2757.Contig3\_All, Unigene59858\_All, CL6502.Contig1\_All, Unigene25297\_All, CL7843.Contig3\_All, CL3333.Contig4\_All, Unigene25738\_All, CL238.Contig57\_All, Unigene23799\_All, CL6555.Contig7\_All, CL2233.Contig9\_All, Unigene44382\_All, CL7032.Contig1\_All, Unigene8100\_All, Unigene55841\_All, CL3593.Contig3\_All, CL8389.Contig2\_All, Unigene12972\_All, Unigene56577\_All, Unigene3061\_All, Unigene9422\_All, Unigene58425\_All, Unigene60002\_All, CL3930.Contig3\_All, CL13490.Contig1\_All, Unigene31739\_All, CL1776.Contig3\_All, CL5296.Contig1\_All, CL8234.Contig1\_All, Unigene41646\_All, CL10515.Contig2\_All, CL1045.Contig1\_All, CL12628.Contig1\_All, Unigene62552\_All, CL9711.Contig3\_All, Unigene41797\_All, CL1814.Contig3\_All, Unigene60207\_All, Unigene56805\_All, CL3875.Contig1\_All, CL5527.Contig4\_All, Unigene29884\_All, CL10515.Contig1\_All, CL2032.Contig4\_All, CL581.Contig3\_All, Unigene29847\_All, Unigene20331\_All, Unigene55459\_All, Unigene4339\_All, CL194.Contig3\_All, Unigene29872\_All, CL5747.Contig1\_All, CL10040.Contig2\_All, Unigene59047\_All, Unigene64694\_All, CL2032.Contig5\_All, CL8237.Contig2\_All, Unigene36019\_All, Unigene14151\_All, CL581.Contig1\_All, CL12628.Contig2\_All, CL11627.Contig2\_All, CL1895.Contig14\_All, CL5663.Contig2\_All, CL1726.Contig2\_All, Unigene15457\_All, CL7032.Contig2\_All, CL14008.Contig2\_All, CL1817.Contig1\_All, CL2176.Contig4\_All, Unigene39534\_All, Unigene57584\_All, CL238.Contig33\_All, CL1310.Contig2\_All, CL5527.Contig1\_All, Unigene56993\_All, CL2087.Contig1\_All, Unigene58783\_All, CL8195.Contig2\_All, CL6496.Contig1\_All, Unigene5483\_All, Unigene59048\_All, CL2032.Contig2\_All, Unigene42793\_All, CL10224.Contig1\_All, CL683.Contig2\_All, CL3507.Contig4\_All, Unigene9046\_All, CL2032.Contig7\_All, Unigene29920\_All, CL2330.Contig4\_All, Unigene18990\_All, CL9400.Contig2\_All, Unigene23512\_All, Unigene7124\_All, CL238.Contig39\_All, Unigene3685\_All, CL5008.Contig3\_All, Unigene17028\_All, CL11627.Contig3\_All, CL1776.Contig1\_All, CL2587.Contig1\_All, CL1690.Contig1\_All, Unigene65156\_All, CL1895.Contig15\_All, Unigene57585\_All, CL1817.Contig5\_All, CL2588.Contig1\_All, CL3333.Contig1\_All, CL6903.Contig2\_All, CL2330.Contig2\_All, Unigene37291\_All, CL3261.Contig4\_All, CL11417.Contig2\_All, Unigene33962\_All, Unigene26998\_All, Unigene3226\_All, CL13309.Contig1\_All, Unigene29581\_All, Unigene61438\_All, CL13893.Contig1\_All, CL1814.Contig4\_All, Unigene41430\_All, CL11742.Contig1\_All, CL8389.Contig1\_All, Unigene10514\_All, CL3593.Contig2\_All, CL683.Contig3\_All, Unigene30566\_All, Unigene62583\_All, CL238.Contig47\_All, CL1895.Contig13\_All, CL11002.Contig2\_All, Unigene41520\_All, CL238.Contig42\_All, CL2596.Contig2\_All, Unigene20151\_All, CL11002.Contig1\_All, CL10971.Contig2\_All, Unigene5926\_All, CL3930.Contig5\_All, CL8523.Contig1\_All, CL238.Contig40\_All, Unigene43661\_All, Unigene3643\_All, CL2233.Contig3\_All, Unigene13893\_All, CL3524.Contig1\_All, Unigene27302\_All |
| carbohydrate derivative binding | CL13014.Contig1\_All, CL13014.Contig3\_All, CL1600.Contig3\_All, CL219.Contig1\_All, CL4188.Contig2\_All, CL4188.Contig1\_All, CL13541.Contig1\_All |
| carboxylesterase activity | CL2161.Contig2\_All, Unigene58560\_All, CL5222.Contig3\_All, CL2012.Contig4\_All, CL11884.Contig2\_All, Unigene62245\_All, Unigene47014\_All, Unigene56581\_All, CL1405.Contig1\_All, Unigene17341\_All, Unigene56165\_All, CL4132.Contig2\_All, CL9188.Contig1\_All, Unigene29754\_All, CL11884.Contig1\_All, CL2012.Contig2\_All, CL1405.Contig3\_All, CL13215.Contig2\_All, Unigene32360\_All, CL4846.Contig2\_All, CL720.Contig2\_All, Unigene24441\_All, Unigene42939\_All, Unigene61105\_All, CL2012.Contig1\_All, Unigene28329\_All, CL4314.Contig6\_All, CL7491.Contig2\_All, CL12119.Contig2\_All, CL4132.Contig1\_All, CL9309.Contig2\_All, Unigene57638\_All, CL5745.Contig1\_All, Unigene31769\_All, CL3763.Contig1\_All |
| peroxiredoxin activity | Unigene10514\_All, CL3593.Contig3\_All, Unigene9046\_All, CL3593.Contig2\_All, CL9400.Contig2\_All, Unigene15457\_All, CL13309.Contig1\_All, Unigene23799\_All, Unigene64694\_All |
| carotenoid isomerase activity | Unigene18047\_All, Unigene61417\_All, Unigene56835\_All, Unigene58406\_All |
| L-leucine transaminase activity | Unigene19806\_All, CL944.Contig3\_All, CL944.Contig2\_All, CL944.Contig1\_All |
| L-valine transaminase activity | Unigene19806\_All, CL944.Contig3\_All, CL944.Contig2\_All, CL944.Contig1\_All |
| L-isoleucine transaminase activity | Unigene19806\_All, CL944.Contig3\_All, CL944.Contig2\_All, CL944.Contig1\_All |
| phosphoserine phosphatase activity | CL4857.Contig2\_All, CL12371.Contig1\_All, Unigene34935\_All, CL4798.Contig1\_All, Unigene36255\_All, CL4798.Contig2\_All |
| choline dehydrogenase activity | CL6022.Contig4\_All, CL6022.Contig2\_All, Unigene4398\_All, CL6526.Contig1\_All, CL6526.Contig2\_All, Unigene11358\_All |
| DNA polymerase processivity factor activity | Unigene45706\_All, Unigene10774\_All, CL3344.Contig3\_All, Unigene14415\_All, Unigene5467\_All, CL3344.Contig1\_All |
| aspartic-type endopeptidase activity | Unigene14110\_All, Unigene61253\_All, CL13776.Contig1\_All, CL6648.Contig2\_All, CL11287.Contig1\_All, CL1844.Contig1\_All, Unigene26822\_All, CL6683.Contig1\_All, CL5667.Contig1\_All, CL6648.Contig1\_All, Unigene63754\_All, CL1184.Contig5\_All, CL5001.Contig3\_All, CL3294.Contig2\_All, Unigene35204\_All, Unigene19964\_All, Unigene37614\_All, Unigene20846\_All, Unigene63445\_All, Unigene58440\_All, Unigene3714\_All, CL13776.Contig2\_All, CL5001.Contig5\_All, Unigene14163\_All, CL11287.Contig2\_All, Unigene65415\_All, Unigene22918\_All, Unigene22861\_All |
| NADPH dehydrogenase activity | Unigene58150\_All, Unigene55745\_All, CL9639.Contig2\_All, CL9639.Contig1\_All, Unigene57674\_All |
| ribose-5-phosphate isomerase activity | Unigene31529\_All, Unigene5838\_All, CL5077.Contig2\_All, CL879.Contig5\_All, CL879.Contig1\_All |
| NADP binding | CL9128.Contig2\_All, Unigene3242\_All, CL1053.Contig1\_All, Unigene42744\_All, Unigene42390\_All, Unigene912\_All, Unigene57584\_All, CL12720.Contig2\_All, Unigene32418\_All, CL3610.Contig7\_All, CL2260.Contig2\_All, Unigene60207\_All, Unigene14655\_All, Unigene62945\_All, Unigene3614\_All, Unigene42184\_All, Unigene42870\_All, CL3610.Contig2\_All, CL9128.Contig1\_All, Unigene22442\_All, CL12972.Contig1\_All, CL1986.Contig2\_All, CL12503.Contig2\_All, Unigene58117\_All, Unigene58317\_All, CL3283.Contig3\_All, CL7843.Contig3\_All, Unigene59694\_All, Unigene28560\_All, Unigene10549\_All, CL258.Contig2\_All, CL9973.Contig3\_All, CL1053.Contig2\_All, CL3610.Contig5\_All, Unigene15289\_All, Unigene29396\_All, CL2587.Contig1\_All, CL2777.Contig1\_All, Unigene57585\_All |
| galacturan 1,4-alpha-galacturonidase activity | CL4361.Contig3\_All, CL4361.Contig1\_All, Unigene4204\_All, Unigene10260\_All, Unigene13736\_All, Unigene20392\_All, CL4361.Contig2\_All |
| inorganic diphosphatase activity | CL11584.Contig1\_All, CL479.Contig6\_All, Unigene31329\_All, CL6201.Contig1\_All, Unigene6405\_All, CL479.Contig2\_All, CL479.Contig1\_All, CL6201.Contig2\_All, Unigene57194\_All, CL6201.Contig4\_All, Unigene15144\_All, CL479.Contig3\_All, CL1442.Contig5\_All, CL6201.Contig3\_All, CL479.Contig4\_All, CL479.Contig5\_All, Unigene37012\_All |
| cinnamyl-alcohol dehydrogenase activity | CL2814.Contig6\_All, CL2814.Contig4\_All, Unigene26365\_All, Unigene23051\_All, CL11925.Contig1\_All, CL2814.Contig3\_All, CL2814.Contig5\_All, Unigene44648\_All |
| minus-end-directed microtubule motor activity | Unigene3476\_All, Unigene14687\_All, CL5858.Contig7\_All, Unigene55733\_All, CL5858.Contig8\_All, Unigene46682\_All |
| mandelonitrile lyase activity | CL6022.Contig4\_All, CL6022.Contig2\_All, Unigene4398\_All, CL341.Contig1\_All, CL6526.Contig1\_All, Unigene11358\_All |
| carnitine racemase activity | CL9060.Contig1\_All, CL6726.Contig2\_All, CL6726.Contig1\_All |
| nicotinamidase activity | Unigene40987\_All, Unigene14927\_All, Unigene22882\_All |
| octanoyltransferase activity | Unigene1040\_All, Unigene64423\_All, Unigene10488\_All |
| lipoyltransferase activity | Unigene1040\_All, Unigene64423\_All, Unigene10488\_All |
| S-formylglutathione hydrolase activity | CL4192.Contig1\_All, Unigene42939\_All, Unigene47014\_All |
| sinapyl alcohol dehydrogenase activity | Unigene26365\_All, CL11925.Contig1\_All, Unigene44648\_All |
| monooxygenase activity | CL2032.Contig1\_All, CL2176.Contig4\_All, Unigene12838\_All, CL3930.Contig1\_All, Unigene13680\_All, Unigene57584\_All, CL3930.Contig4\_All, CL7484.Contig2\_All, CL238.Contig33\_All, CL9328.Contig1\_All, CL1310.Contig2\_All, CL5527.Contig1\_All, CL12184.Contig1\_All, Unigene38900\_All, Unigene5483\_All, Unigene59048\_All, CL2032.Contig2\_All, Unigene34671\_All, CL4994.Contig9\_All, Unigene42146\_All, CL10224.Contig1\_All, CL683.Contig2\_All, CL9578.Contig2\_All, CL3333.Contig2\_All, CL3507.Contig4\_All, CL2032.Contig7\_All, Unigene18990\_All, CL2330.Contig4\_All, Unigene25297\_All, Unigene23512\_All, CL7843.Contig3\_All, CL3333.Contig4\_All, CL238.Contig57\_All, CL6555.Contig7\_All, CL2233.Contig9\_All, CL238.Contig39\_All, Unigene44382\_All, Unigene3685\_All, CL7032.Contig1\_All, Unigene8100\_All, Unigene55841\_All, CL11627.Contig3\_All, Unigene12972\_All, CL2587.Contig1\_All, CL1690.Contig1\_All, Unigene3061\_All, Unigene65156\_All, Unigene58425\_All, Unigene60002\_All, CL3930.Contig3\_All, CL13490.Contig1\_All, Unigene57585\_All, Unigene31739\_All, CL1817.Contig5\_All, CL1288.Contig4\_All, CL3333.Contig1\_All, CL6903.Contig2\_All, CL2330.Contig2\_All, Unigene37291\_All, CL5296.Contig1\_All, CL3261.Contig4\_All, CL11417.Contig2\_All, Unigene41646\_All, Unigene33962\_All, CL10515.Contig2\_All, Unigene4846\_All, Unigene6988\_All, Unigene61438\_All, Unigene29581\_All, CL12628.Contig1\_All, Unigene62552\_All, CL13893.Contig1\_All, Unigene40378\_All, CL1288.Contig3\_All, CL9711.Contig3\_All, Unigene60207\_All, CL683.Contig3\_All, Unigene55021\_All, Unigene56805\_All, CL5527.Contig4\_All, Unigene29884\_All, CL10515.Contig1\_All, CL2032.Contig4\_All, Unigene30566\_All, CL238.Contig47\_All, Unigene29847\_All, Unigene62583\_All, Unigene55459\_All, Unigene4339\_All, Unigene11881\_All, CL3719.Contig12\_All, CL2596.Contig2\_All, CL238.Contig42\_All, Unigene20151\_All, Unigene29872\_All, CL10040.Contig2\_All, Unigene59047\_All, Unigene5926\_All, CL2032.Contig5\_All, CL3930.Contig5\_All, CL238.Contig40\_All, Unigene43661\_All, CL2233.Contig3\_All, Unigene36019\_All, CL11627.Contig2\_All, Unigene59816\_All, CL12628.Contig2\_All, Unigene13893\_All, CL5663.Contig2\_All, Unigene16517\_All, CL7032.Contig2\_All, Unigene27302\_All, CL14008.Contig2\_All, CL1817.Contig1\_All |
| RNA binding | CL7564.Contig1\_All, Unigene47188\_All, CL7395.Contig5\_All, Unigene6759\_All, CL2222.Contig1\_All, CL2048.Contig4\_All, CL12504.Contig2\_All, Unigene236\_All, CL5814.Contig2\_All, CL1339.Contig5\_All, CL4642.Contig3\_All, CL6620.Contig1\_All, CL10853.Contig2\_All, CL6108.Contig1\_All, CL2786.Contig6\_All, CL9286.Contig1\_All, Unigene59177\_All, CL6998.Contig1\_All, CL4661.Contig2\_All, CL2987.Contig1\_All, Unigene57719\_All, CL116.Contig3\_All, Unigene58013\_All, Unigene19750\_All, CL12348.Contig1\_All, CL3722.Contig3\_All, CL3286.Contig2\_All, Unigene3973\_All, Unigene6078\_All, CL1443.Contig1\_All, Unigene32496\_All, CL9960.Contig4\_All, Unigene55213\_All, Unigene63754\_All, Unigene15470\_All, CL386.Contig2\_All, CL10690.Contig2\_All, CL161.Contig1\_All, CL1430.Contig11\_All, CL26.Contig3\_All, Unigene43305\_All, CL11275.Contig2\_All, CL1351.Contig1\_All, Unigene35204\_All, Unigene34935\_All, CL2153.Contig1\_All, Unigene56324\_All, Unigene61423\_All, CL1071.Contig2\_All, CL8076.Contig2\_All, Unigene6711\_All, Unigene61511\_All, CL4661.Contig3\_All, CL812.Contig1\_All, CL10085.Contig3\_All, Unigene19707\_All, Unigene23489\_All, CL1308.Contig1\_All, Unigene28643\_All, CL8252.Contig1\_All, Unigene33589\_All, Unigene37315\_All, CL13973.Contig1\_All, Unigene56835\_All, CL1430.Contig6\_All, Unigene33641\_All, Unigene57190\_All, CL5814.Contig1\_All, Unigene7218\_All, Unigene15881\_All, CL2786.Contig21\_All, CL11478.Contig1\_All, Unigene40532\_All, CL12417.Contig11\_All, Unigene60740\_All, Unigene1036\_All, Unigene54418\_All, Unigene4385\_All, CL7059.Contig1\_All, CL1449.Contig2\_All, Unigene42961\_All, Unigene10129\_All, CL14023.Contig1\_All, Unigene54422\_All, Unigene54665\_All, Unigene24742\_All, CL386.Contig3\_All, Unigene22803\_All, CL544.Contig2\_All, CL2782.Contig2\_All, Unigene59157\_All, CL9503.Contig1\_All, CL10853.Contig1\_All, Unigene19727\_All, CL13964.Contig25\_All, Unigene38355\_All, CL10583.Contig2\_All, CL6465.Contig2\_All, CL2586.Contig1\_All, CL4354.Contig1\_All, CL13688.Contig2\_All, CL11026.Contig1\_All, Unigene41885\_All, CL13641.Contig3\_All, CL604.Contig1\_All, CL13832.Contig3\_All, Unigene29969\_All, CL1438.Contig2\_All, CL4796.Contig6\_All, CL4642.Contig4\_All, CL90.Contig3\_All, CL3993.Contig3\_All, Unigene42825\_All, Unigene25657\_All, CL854.Contig3\_All, Unigene36138\_All, CL11803.Contig2\_All, Unigene17239\_All, CL8349.Contig3\_All, CL1911.Contig7\_All, Unigene805\_All, CL10623.Contig1\_All, Unigene55957\_All, CL2071.Contig5\_All, CL10085.Contig2\_All, Unigene26301\_All, CL6733.Contig2\_All, Unigene25790\_All, Unigene63219\_All, CL2182.Contig1\_All, CL6318.Contig3\_All, CL6048.Contig1\_All, CL8248.Contig7\_All, Unigene32076\_All, Unigene54729\_All, Unigene14492\_All, CL12828.Contig2\_All, Unigene55760\_All, Unigene56196\_All, Unigene56646\_All, CL6620.Contig2\_All, CL8643.Contig2\_All, Unigene42315\_All, Unigene23065\_All, Unigene8052\_All, CL8848.Contig2\_All, CL10111.Contig1\_All, CL2642.Contig2\_All, CL4794.Contig1\_All, CL11726.Contig1\_All, Unigene16581\_All, Unigene20195\_All, Unigene54412\_All, Unigene25625\_All, CL3019.Contig1\_All, Unigene40509\_All, Unigene22270\_All, CL9824.Contig1\_All, CL90.Contig2\_All, Unigene31422\_All, CL8314.Contig2\_All, Unigene4202\_All, CL6318.Contig1\_All, CL10950.Contig2\_All, Unigene32999\_All, Unigene8835\_All, CL1184.Contig5\_All, CL2845.Contig2\_All, CL2340.Contig1\_All, Unigene42555\_All, CL2206.Contig1\_All, Unigene4820\_All, CL12762.Contig1\_All, CL12052.Contig1\_All, CL13641.Contig1\_All, Unigene45133\_All, CL1025.Contig1\_All, CL1249.Contig2\_All, CL10516.Contig3\_All, CL13326.Contig2\_All, Unigene31710\_All, CL4298.Contig1\_All, Unigene38433\_All, Unigene28581\_All, Unigene40435\_All, Unigene32107\_All, CL461.Contig3\_All, CL1849.Contig2\_All, CL2907.Contig3\_All, Unigene33797\_All, CL1911.Contig6\_All, CL5666.Contig1\_All, CL8643.Contig5\_All, Unigene20602\_All, Unigene30945\_All, Unigene42928\_All, CL1449.Contig1\_All, CL10618.Contig1\_All, CL10096.Contig1\_All, CL7704.Contig1\_All, Unigene42950\_All, Unigene27024\_All, CL3339.Contig2\_All, CL9323.Contig2\_All, Unigene57094\_All, CL2256.Contig4\_All, Unigene54623\_All, CL9824.Contig2\_All, CL11592.Contig2\_All, Unigene6077\_All, CL7658.Contig2\_All, Unigene30350\_All, CL2256.Contig2\_All, Unigene23393\_All, CL386.Contig1\_All, Unigene38848\_All, Unigene9264\_All, Unigene65415\_All, Unigene12371\_All, Unigene10138\_All, Unigene59092\_All, Unigene14110\_All, CL1430.Contig13\_All, Unigene33046\_All, Unigene61614\_All, Unigene60337\_All, CL1849.Contig1\_All, CL8052.Contig1\_All, Unigene36255\_All, Unigene47298\_All, CL10298.Contig1\_All, Unigene32702\_All, CL8076.Contig1\_All, Unigene15519\_All, CL12599.Contig1\_All, Unigene14730\_All, CL5809.Contig5\_All, CL10354.Contig1\_All, CL2200.Contig2\_All, CL5506.Contig1\_All, Unigene33591\_All, Unigene25614\_All, CL2320.Contig3\_All, Unigene42250\_All, CL10575.Contig1\_All, Unigene16902\_All, Unigene18179\_All, Unigene22228\_All, Unigene40215\_All, Unigene56323\_All, Unigene14583\_All, Unigene15951\_All, Unigene58798\_All, CL18.Contig4\_All, CL4701.Contig2\_All, Unigene60458\_All, CL1198.Contig3\_All, CL1586.Contig4\_All, Unigene37882\_All, CL3305.Contig1\_All, CL10253.Contig1\_All, CL7737.Contig1\_All, CL13352.Contig2\_All, CL7231.Contig1\_All, CL9204.Contig4\_All, Unigene11750\_All, CL2782.Contig1\_All, Unigene43054\_All, Unigene46682\_All, Unigene19923\_All, Unigene27997\_All, Unigene18047\_All, CL2222.Contig2\_All, CL8643.Contig1\_All, CL3295.Contig4\_All, CL1249.Contig1\_All, Unigene31764\_All, CL13832.Contig1\_All, CL1911.Contig9\_All, Unigene60958\_All, Unigene60256\_All, CL10083.Contig1\_All, CL6519.Contig1\_All, CL10583.Contig1\_All, Unigene63864\_All, Unigene54419\_All, Unigene29178\_All, Unigene12169\_All, CL3060.Contig4\_All, Unigene20080\_All, CL2586.Contig2\_All, Unigene8237\_All, Unigene25738\_All, Unigene8532\_All, CL12828.Contig1\_All, CL7395.Contig3\_All, CL150.Contig5\_All, Unigene23124\_All, CL3583.Contig1\_All, Unigene6773\_All, CL4701.Contig1\_All, CL13448.Contig1\_All, CL715.Contig1\_All, CL13326.Contig17\_All, Unigene6708\_All, CL1339.Contig4\_All, Unigene40080\_All, Unigene42887\_All, CL8720.Contig1\_All, CL1430.Contig4\_All, Unigene29507\_All, CL3019.Contig2\_All, Unigene18154\_All, CL3972.Contig3\_All, CL12446.Contig7\_All, CL10201.Contig2\_All, CL10105.Contig1\_All, Unigene38556\_All, CL2921.Contig2\_All, Unigene24620\_All, CL12408.Contig2\_All, CL1308.Contig2\_All, Unigene62007\_All, CL13496.Contig1\_All, Unigene34402\_All, CL5945.Contig3\_All, CL5949.Contig1\_All, CL6296.Contig2\_All, Unigene15283\_All, Unigene13493\_All, CL1911.Contig8\_All, Unigene41202\_All, CL4862.Contig1\_All, CL5555.Contig2\_All, Unigene28704\_All, CL5962.Contig1\_All, CL2256.Contig1\_All, Unigene24094\_All, Unigene2850\_All, Unigene1316\_All, Unigene55797\_All, CL9193.Contig1\_All, Unigene63445\_All, CL12538.Contig2\_All, CL1477.Contig2\_All, CL5176.Contig5\_All, CL12178.Contig1\_All, Unigene41843\_All, CL367.Contig1\_All, CL326.Contig2\_All, CL5540.Contig1\_All, CL3167.Contig1\_All, Unigene32598\_All, Unigene28880\_All, Unigene46996\_All, Unigene34854\_All, Unigene23394\_All, CL5949.Contig2\_All, Unigene23149\_All, CL10446.Contig1\_All, CL3060.Contig1\_All, CL13861.Contig5\_All, CL1911.Contig10\_All, Unigene29982\_All, CL12756.Contig2\_All, CL9960.Contig3\_All, CL3946.Contig1\_All, Unigene60069\_All, Unigene2295\_All, CL837.Contig2\_All, Unigene41232\_All, Unigene60826\_All, CL6818.Contig2\_All, CL4186.Contig2\_All, CL3583.Contig2\_All, CL2829.Contig1\_All, CL13379.Contig2\_All, CL6487.Contig3\_All, Unigene60978\_All, CL6465.Contig3\_All, CL7725.Contig1\_All, Unigene30740\_All, CL976.Contig2\_All, CL10623.Contig2\_All, CL2555.Contig3\_All, CL13378.Contig1\_All, CL10770.Contig1\_All, CL7737.Contig2\_All, Unigene61417\_All, Unigene51140\_All, Unigene32315\_All, CL2786.Contig14\_All, Unigene32059\_All, Unigene7670\_All, CL5176.Contig4\_All, CL6296.Contig1\_All, CL604.Contig7\_All, CL3603.Contig5\_All, CL805.Contig3\_All, Unigene43514\_All, Unigene14921\_All, Unigene39862\_All, CL3820.Contig3\_All, CL7997.Contig1\_All, Unigene61490\_All, CL2182.Contig3\_All, CL10560.Contig1\_All, Unigene37614\_All, CL9286.Contig2\_All, Unigene27330\_All, CL371.Contig1\_All, CL9204.Contig1\_All, CL6792.Contig1\_All, CL13326.Contig13\_All, Unigene10216\_All, CL5285.Contig1\_All, Unigene41632\_All, CL12751.Contig2\_All, CL36.Contig9\_All, CL2845.Contig3\_All, Unigene21939\_All, CL13378.Contig2\_All, CL1948.Contig3\_All, Unigene59302\_All, Unigene41282\_All, Unigene31261\_All, CL6328.Contig2\_All, CL7539.Contig2\_All, CL13326.Contig15\_All, Unigene61202\_All, CL7188.Contig1\_All, Unigene60274\_All, CL6376.Contig1\_All, Unigene23043\_All, CL2340.Contig2\_All, Unigene18804\_All, CL12272.Contig1\_All, Unigene61124\_All, CL905.Contig3\_All, Unigene47722\_All, Unigene39218\_All, CL7997.Contig2\_All, CL5683.Contig2\_All, CL8846.Contig1\_All, CL854.Contig4\_All, CL13326.Contig10\_All, CL8843.Contig3\_All, Unigene16516\_All, Unigene30448\_All, Unigene57248\_All, CL12408.Contig3\_All, CL604.Contig6\_All, Unigene55399\_All, CL12484.Contig2\_All, Unigene6846\_All, CL11239.Contig1\_All, CL8090.Contig3\_All, Unigene16255\_All, Unigene24550\_All, Unigene43808\_All, Unigene36263\_All, Unigene54413\_All, Unigene22234\_All, Unigene42069\_All, Unigene22400\_All, CL11514.Contig1\_All, Unigene24136\_All, CL6335.Contig1\_All, Unigene3774\_All, CL7725.Contig2\_All, Unigene14807\_All, Unigene39156\_All, Unigene5883\_All, CL7658.Contig1\_All, CL3339.Contig1\_All, CL4909.Contig2\_All, CL3295.Contig2\_All, CL8349.Contig1\_All, CL5000.Contig1\_All, CL7231.Contig2\_All, CL2555.Contig2\_All, CL12484.Contig1\_All, CL3286.Contig1\_All, CL2987.Contig2\_All, CL13379.Contig1\_All, Unigene58440\_All, CL9286.Contig3\_All, CL13326.Contig18\_All, CL4857.Contig2\_All, Unigene28108\_All, Unigene63946\_All, CL2048.Contig3\_All, CL812.Contig3\_All, CL5356.Contig2\_All, CL2320.Contig2\_All, CL2048.Contig2\_All |
| aryl-alcohol dehydrogenase (NAD+) activity | CL2814.Contig6\_All, CL2814.Contig4\_All, CL2814.Contig3\_All, CL2814.Contig5\_All |
| manganese ion binding | CL2901.Contig1\_All, CL7188.Contig1\_All, CL7100.Contig2\_All, Unigene22601\_All, Unigene27776\_All, CL2901.Contig2\_All, Unigene41651\_All, Unigene40910\_All, Unigene10286\_All, CL7100.Contig3\_All, Unigene26948\_All, CL9625.Contig2\_All, Unigene25662\_All, CL10085.Contig3\_All, Unigene21065\_All, Unigene32323\_All, Unigene61043\_All, CL8814.Contig4\_All, CL392.Contig4\_All, Unigene21419\_All, Unigene28979\_All, Unigene10624\_All, CL392.Contig3\_All, CL10085.Contig2\_All |
| carbon-sulfur lyase activity | CL9129.Contig2\_All, CL8994.Contig2\_All, Unigene27688\_All, CL2999.Contig1\_All, Unigene14568\_All, CL6364.Contig2\_All, Unigene26165\_All, Unigene16548\_All, CL4875.Contig2\_All, Unigene4967\_All, Unigene4846\_All, Unigene56757\_All, Unigene15533\_All, CL8994.Contig1\_All, CL9601.Contig1\_All, CL9850.Contig4\_All, Unigene56803\_All, Unigene56802\_All, CL6364.Contig1\_All, CL4875.Contig1\_All, Unigene38624\_All, CL9850.Contig3\_All, CL9146.Contig1\_All |
| sodium ion transmembrane transporter activity | Unigene22965\_All, CL817.Contig4\_All, CL7188.Contig1\_All, Unigene61494\_All, CL1760.Contig3\_All, Unigene22297\_All, Unigene16784\_All, Unigene18860\_All, CL10085.Contig3\_All, CL2965.Contig2\_All, CL7413.Contig2\_All, Unigene43692\_All, Unigene60257\_All, CL3986.Contig4\_All, Unigene59384\_All, CL10085.Contig2\_All, CL7413.Contig1\_All, Unigene59589\_All |
| procollagen-proline 4-dioxygenase activity | CL1895.Contig14\_All, CL2588.Contig1\_All, CL1895.Contig13\_All, Unigene40795\_All, CL2588.Contig2\_All, CL1895.Contig15\_All |
| chitin binding | CL13014.Contig1\_All, CL13014.Contig3\_All, CL1600.Contig3\_All, CL219.Contig1\_All, CL4188.Contig2\_All, CL4188.Contig1\_All |
| procollagen-proline dioxygenase activity | CL1895.Contig14\_All, CL2588.Contig1\_All, CL1895.Contig13\_All, Unigene40795\_All, CL2588.Contig2\_All, CL1895.Contig15\_All |
| peptidyl-proline dioxygenase activity | CL1895.Contig14\_All, CL2588.Contig1\_All, CL1895.Contig13\_All, Unigene40795\_All, CL2588.Contig2\_All, CL1895.Contig15\_All |
| peptidyl-proline 4-dioxygenase activity | CL1895.Contig14\_All, CL2588.Contig1\_All, CL1895.Contig13\_All, Unigene40795\_All, CL2588.Contig2\_All, CL1895.Contig15\_All |
| coenzyme binding | Unigene21226\_All, Unigene3242\_All, CL1239.Contig2\_All, Unigene11358\_All, CL8737.Contig1\_All, CL10257.Contig2\_All, Unigene10484\_All, CL5832.Contig1\_All, CL4812.Contig3\_All, CL3610.Contig7\_All, CL6062.Contig1\_All, CL7971.Contig3\_All, CL6119.Contig1\_All, CL12769.Contig2\_All, Unigene42184\_All, Unigene39960\_All, CL7457.Contig2\_All, CL9128.Contig1\_All, CL4994.Contig9\_All, CL1239.Contig1\_All, Unigene34737\_All, CL6005.Contig1\_All, Unigene38690\_All, CL12933.Contig1\_All, CL949.Contig1\_All, CL4812.Contig5\_All, CL949.Contig3\_All, Unigene54419\_All, Unigene17142\_All, CL12503.Contig2\_All, Unigene63349\_All, CL341.Contig1\_All, CL6526.Contig1\_All, CL7843.Contig3\_All, CL238.Contig57\_All, CL6119.Contig2\_All, Unigene56231\_All, CL258.Contig2\_All, Unigene34381\_All, CL7971.Contig2\_All, CL1053.Contig2\_All, CL3610.Contig5\_All, Unigene12122\_All, CL1232.Contig14\_All, CL1283.Contig4\_All, Unigene59986\_All, CL6022.Contig2\_All, CL6062.Contig3\_All, Unigene56835\_All, Unigene19567\_All, CL1053.Contig1\_All, CL6022.Contig4\_All, Unigene41207\_All, Unigene14349\_All, CL10257.Contig1\_All, Unigene30252\_All, CL9751.Contig2\_All, Unigene54418\_All, Unigene54422\_All, CL1288.Contig3\_All, Unigene60207\_All, CL6896.Contig2\_All, CL2120.Contig4\_All, Unigene29194\_All, CL1232.Contig8\_All, Unigene40707\_All, Unigene59793\_All, Unigene42278\_All, CL5273.Contig1\_All, CL2702.Contig4\_All, CL3610.Contig2\_All, CL8001.Contig1\_All, Unigene58324\_All, CL9354.Contig4\_All, CL1986.Contig2\_All, CL8389.Contig3\_All, CL4825.Contig2\_All, CL10040.Contig2\_All, Unigene55119\_All, Unigene36489\_All, Unigene59773\_All, CL12386.Contig1\_All, Unigene54321\_All, Unigene10549\_All, CL12933.Contig2\_All, CL3993.Contig3\_All, CL9973.Contig3\_All, Unigene9754\_All, CL1001.Contig3\_All, Unigene38128\_All, Unigene26632\_All, Unigene29396\_All, Unigene32479\_All, Unigene25365\_All, CL3574.Contig5\_All, CL2777.Contig1\_All, Unigene59471\_All, Unigene38927\_All, CL3795.Contig1\_All, Unigene42744\_All, CL1232.Contig10\_All, Unigene42390\_All, Unigene912\_All, Unigene57584\_All, CL12720.Contig2\_All, CL5185.Contig2\_All, Unigene7919\_All, CL238.Contig33\_All, CL6487.Contig3\_All, CL10875.Contig1\_All, Unigene32418\_All, Unigene34735\_All, CL7100.Contig3\_All, Unigene7472\_All, Unigene589\_All, CL6767.Contig1\_All, Unigene36907\_All, CL3894.Contig3\_All, CL98.Contig18\_All, Unigene3383\_All, Unigene54412\_All, Unigene14655\_All, Unigene31139\_All, Unigene3614\_All, Unigene59884\_All, Unigene42870\_All, CL4972.Contig3\_All, Unigene34736\_All, Unigene22442\_All, CL12972.Contig1\_All, CL1001.Contig2\_All, CL1425.Contig2\_All, CL5723.Contig3\_All, CL1207.Contig3\_All, Unigene10725\_All, Unigene7410\_All, Unigene58117\_All, Unigene58317\_All, Unigene4665\_All, Unigene28560\_All, Unigene59694\_All, CL238.Contig39\_All, CL3894.Contig1\_All, CL5672.Contig1\_All, CL4825.Contig1\_All, Unigene15289\_All, Unigene59572\_All, CL2587.Contig1\_All, Unigene14167\_All, CL1690.Contig1\_All, CL2670.Contig1\_All, Unigene57585\_All, CL9128.Contig2\_All, Unigene63707\_All, CL1525.Contig2\_All, CL1288.Contig4\_All, CL11392.Contig2\_All, CL9645.Contig1\_All, Unigene4398\_All, CL6526.Contig2\_All, Unigene7531\_All, CL6767.Contig2\_All, Unigene58323\_All, CL923.Contig4\_All, CL8389.Contig1\_All, CL1347.Contig3\_All, CL2260.Contig2\_All, CL1232.Contig6\_All, CL12769.Contig1\_All, CL1997.Contig2\_All, Unigene55021\_All, CL1283.Contig5\_All, Unigene62945\_All, CL923.Contig3\_All, CL2702.Contig2\_All, CL9892.Contig3\_All, CL283.Contig5\_All, CL5672.Contig2\_All, Unigene10799\_All, CL238.Contig47\_All, Unigene54413\_All, CL6062.Contig2\_All, CL7100.Contig2\_All, CL4972.Contig1\_All, CL238.Contig42\_All, CL3296.Contig1\_All, CL3283.Contig3\_All, Unigene55183\_All, CL11392.Contig1\_All, CL13390.Contig2\_All, CL238.Contig40\_All, CL3925.Contig3\_All, Unigene34380\_All, Unigene61369\_All, CL2003.Contig6\_All, Unigene3440\_All |
| oxidoreductase activity, acting on the CH-NH2 group of donors | Unigene9305\_All, CL6005.Contig1\_All, CL5818.Contig3\_All, Unigene7376\_All, Unigene19331\_All, Unigene54807\_All, Unigene29920\_All, CL14006.Contig1\_All, CL5167.Contig1\_All, Unigene25738\_All, CL5832.Contig1\_All, Unigene26932\_All, Unigene46513\_All, Unigene702\_All, Unigene23920\_All, Unigene7449\_All, CL13997.Contig1\_All, CL2702.Contig2\_All, CL5818.Contig2\_All, CL2702.Contig4\_All, Unigene41493\_All, CL13997.Contig3\_All, CL5167.Contig5\_All, Unigene39960\_All |
| auxin transmembrane transporter activity | Unigene59649\_All, CL13509.Contig2\_All, Unigene7976\_All, Unigene59760\_All, Unigene63864\_All, CL13885.Contig2\_All, Unigene39613\_All, Unigene41679\_All, CL3689.Contig6\_All, CL13885.Contig5\_All, Unigene56646\_All, CL3975.Contig2\_All, Unigene26098\_All, Unigene20789\_All, CL13885.Contig1\_All, CL10296.Contig2\_All, Unigene57960\_All, CL13885.Contig9\_All, Unigene36211\_All, Unigene932\_All, Unigene29618\_All, CL3689.Contig4\_All, CL3622.Contig2\_All, Unigene61641\_All, CL2129.Contig2\_All, Unigene35383\_All |
| IkappaB kinase activity | CL7075.Contig2\_All, Unigene59873\_All |
| 1-aminocyclopropane-1-carboxylate deaminase activity | Unigene4846\_All, Unigene16548\_All |
| DNA-3-methyladenine glycosylase activity | Unigene27762\_All, Unigene16758\_All |
| isochorismatase activity | Unigene40987\_All, Unigene22882\_All |
| UDP-galactose:N-glycan beta-1,3-galactosyltransferase activity | CL271.Contig1\_All, CL271.Contig2\_All |
| 15-hydroxyprostaglandin dehydrogenase (NAD+) activity | CL7072.Contig1\_All, CL7072.Contig2\_All |
| D-cysteine desulfhydrase activity | Unigene4846\_All, Unigene16548\_All |
| DNA-3-methylbase glycosylase activity | Unigene27762\_All, Unigene16758\_All |
| phosphosulfolactate synthase activity | CL8994.Contig1\_All, CL8994.Contig2\_All |
| p-coumarate 3-hydroxylase activity | CL11627.Contig2\_All, CL11627.Contig3\_All |
| histone methyltransferase activity (H3-K36 specific) | Unigene61417\_All, Unigene56835\_All |
| beta-pyrazolylalanine synthase activity | CL3624.Contig3\_All, CL3624.Contig4\_All |
| 9,9'-dicis-carotene:quinone oxidoreductase activity | CL11002.Contig1\_All, CL11002.Contig2\_All |
| 7,9,9'-tricis-neurosporene:quinone oxidoreductase activity | CL11002.Contig1\_All, CL11002.Contig2\_All |
| brassinosteroid sulfotransferase activity | CL4949.Contig2\_All, Unigene61954\_All |
| branched-chain-amino-acid transaminase activity | Unigene19806\_All, CL944.Contig3\_All, CL944.Contig2\_All, Unigene39106\_All, CL944.Contig1\_All, Unigene40623\_All, Unigene33277\_All |
| carboxylic ester hydrolase activity | CL2161.Contig2\_All, Unigene42963\_All, CL5222.Contig3\_All, Unigene62245\_All, CL4128.Contig2\_All, Unigene11251\_All, CL4512.Contig1\_All, Unigene56581\_All, CL1405.Contig1\_All, CL8660.Contig2\_All, Unigene29427\_All, Unigene17341\_All, CL2899.Contig8\_All, Unigene7322\_All, CL3136.Contig2\_All, Unigene56165\_All, Unigene19067\_All, CL3394.Contig2\_All, Unigene39903\_All, CL4132.Contig2\_All, Unigene30179\_All, CL8796.Contig2\_All, CL4324.Contig2\_All, Unigene29754\_All, Unigene20570\_All, CL8660.Contig1\_All, CL7661.Contig1\_All, CL2012.Contig2\_All, Unigene56753\_All, CL1405.Contig3\_All, CL13215.Contig2\_All, Unigene32360\_All, CL5058.Contig1\_All, CL913.Contig2\_All, CL8800.Contig2\_All, CL2390.Contig3\_All, Unigene61105\_All, Unigene28841\_All, CL2012.Contig1\_All, CL8686.Contig2\_All, CL9118.Contig1\_All, Unigene61904\_All, CL7491.Contig2\_All, CL4314.Contig6\_All, CL2899.Contig9\_All, CL7661.Contig2\_All, CL12119.Contig2\_All, Unigene57638\_All, Unigene31769\_All, CL5745.Contig1\_All, CL11990.Contig2\_All, CL3763.Contig1\_All, CL3277.Contig3\_All, Unigene58560\_All, CL9424.Contig1\_All, CL2012.Contig4\_All, Unigene20070\_All, CL11884.Contig2\_All, Unigene58302\_All, Unigene47014\_All, Unigene20069\_All, Unigene29116\_All, CL3442.Contig5\_All, Unigene36522\_All, CL2390.Contig1\_All, CL12241.Contig3\_All, CL4292.Contig1\_All, Unigene10181\_All, CL9188.Contig1\_All, Unigene35873\_All, CL4128.Contig1\_All, Unigene39246\_All, Unigene62708\_All, Unigene16215\_All, Unigene39814\_All, CL11884.Contig1\_All, CL2899.Contig3\_All, CL3277.Contig2\_All, CL3277.Contig1\_All, CL4846.Contig2\_All, CL720.Contig2\_All, Unigene22384\_All, Unigene56056\_All, Unigene5361\_All, Unigene24441\_All, Unigene42939\_All, CL12638.Contig2\_All, Unigene43539\_All, Unigene28329\_All, CL4292.Contig3\_All, CL4132.Contig1\_All, CL9309.Contig2\_All |
| auxin efflux transmembrane transporter activity | Unigene59649\_All, Unigene7976\_All, Unigene59760\_All, Unigene63864\_All, CL13885.Contig2\_All, Unigene39613\_All, Unigene41679\_All, CL13885.Contig5\_All, Unigene56646\_All, CL3975.Contig2\_All, Unigene20789\_All, CL13885.Contig1\_All, CL10296.Contig2\_All, CL13885.Contig9\_All, Unigene36211\_All, Unigene932\_All, CL3622.Contig2\_All, CL2129.Contig2\_All, Unigene61641\_All, Unigene35383\_All |
| 3-isopropylmalate dehydratase activity | CL3802.Contig3\_All, CL3496.Contig5\_All, CL3496.Contig3\_All, CL3496.Contig1\_All, Unigene34868\_All |
| UDP-galactosyltransferase activity | CL7693.Contig1\_All, Unigene16399\_All, CL2618.Contig2\_All, Unigene42368\_All, CL7693.Contig3\_All, CL4521.Contig1\_All, Unigene27662\_All, Unigene22307\_All, CL271.Contig2\_All, CL2618.Contig3\_All, Unigene5078\_All, Unigene19918\_All, CL271.Contig1\_All, Unigene42163\_All, Unigene8864\_All, CL13764.Contig1\_All |
| Ras guanyl-nucleotide exchange factor activity | CL5027.Contig2\_All, Unigene31946\_All, Unigene58629\_All, Unigene17670\_All, Unigene57182\_All, Unigene63628\_All, CL5027.Contig1\_All, Unigene58405\_All, Unigene41656\_All |
| Rho guanyl-nucleotide exchange factor activity | CL5027.Contig2\_All, Unigene31946\_All, Unigene58629\_All, Unigene17670\_All, Unigene57182\_All, Unigene63628\_All, CL5027.Contig1\_All, Unigene58405\_All, Unigene41656\_All |
| 2-alkenal reductase [NAD(P)] activity | CL5167.Contig1\_All, CL649.Contig1\_All, CL8132.Contig1\_All, CL6201.Contig2\_All, Unigene15306\_All, Unigene63665\_All, Unigene20235\_All, CL6730.Contig3\_All, Unigene14805\_All, CL4609.Contig3\_All, CL479.Contig5\_All, CL5167.Contig5\_All, Unigene59478\_All, CL9395.Contig1\_All, CL1195.Contig3\_All, Unigene41308\_All, Unigene56097\_All, Unigene39526\_All, CL7447.Contig1\_All, CL4656.Contig1\_All, CL609.Contig49\_All, CL109.Contig7\_All, CL9152.Contig2\_All, Unigene10664\_All, CL109.Contig4\_All, Unigene15416\_All, Unigene26344\_All, CL10396.Contig2\_All, CL3807.Contig3\_All, CL109.Contig6\_All, CL12729.Contig1\_All, CL8799.Contig2\_All, Unigene3321\_All, Unigene39790\_All, CL7061.Contig3\_All, CL6289.Contig2\_All, CL8493.Contig4\_All, CL5309.Contig1\_All, Unigene56094\_All, Unigene12613\_All, CL4773.Contig2\_All, Unigene13422\_All, Unigene16775\_All, Unigene13446\_All, CL7310.Contig1\_All, CL3405.Contig5\_All, Unigene54999\_All, Unigene61025\_All, CL4982.Contig2\_All, CL10427.Contig4\_All, CL12466.Contig4\_All, Unigene4144\_All, CL8799.Contig1\_All, CL6136.Contig2\_All, CL6289.Contig1\_All, Unigene15535\_All, Unigene24554\_All, CL5146.Contig3\_All, Unigene56096\_All, Unigene24435\_All, Unigene13776\_All, CL211.Contig3\_All, Unigene4259\_All, CL10396.Contig1\_All, CL6804.Contig2\_All, CL109.Contig5\_All, Unigene20850\_All, CL6201.Contig3\_All, CL609.Contig45\_All, CL11965.Contig3\_All, Unigene29846\_All, CL6117.Contig2\_All, Unigene17130\_All, CL603.Contig5\_All, Unigene4205\_All, Unigene57139\_All, CL2225.Contig2\_All, CL1326.Contig2\_All, CL6201.Contig4\_All, CL6434.Contig4\_All, Unigene16753\_All, CL2517.Contig9\_All, Unigene2390\_All, CL6730.Contig2\_All, CL379.Contig1\_All, CL1442.Contig5\_All, Unigene60007\_All, CL1195.Contig2\_All, CL109.Contig2\_All, CL479.Contig4\_All, Unigene13789\_All, CL3038.Contig1\_All, CL8258.Contig1\_All, Unigene28755\_All, CL603.Contig1\_All, Unigene30804\_All, CL2711.Contig1\_All, Unigene3737\_All, Unigene56997\_All, Unigene60014\_All, CL772.Contig2\_All, Unigene6066\_All, CL7818.Contig5\_All, Unigene62417\_All, CL4725.Contig2\_All, CL4773.Contig1\_All, Unigene57038\_All, CL1203.Contig4\_All, Unigene60226\_All, Unigene58820\_All, Unigene56095\_All, CL3991.Contig2\_All, CL7061.Contig4\_All, CL8464.Contig1\_All, Unigene60254\_All, CL479.Contig2\_All, CL5146.Contig4\_All, Unigene41323\_All, CL7818.Contig6\_All, Unigene38732\_All, CL8446.Contig2\_All, Unigene20086\_All, Unigene38704\_All, Unigene13603\_All, CL2710.Contig2\_All, CL13433.Contig1\_All, CL6117.Contig1\_All, CL6201.Contig1\_All, Unigene19285\_All, Unigene59421\_All, CL12964.Contig1\_All, Unigene57352\_All, Unigene1034\_All, CL11965.Contig4\_All, CL2710.Contig1\_All, CL109.Contig1\_All, Unigene33749\_All, CL2200.Contig2\_All, CL8258.Contig2\_All, CL12466.Contig2\_All, Unigene105\_All, Unigene30105\_All, CL2225.Contig1\_All, Unigene17182\_All, Unigene17424\_All, CL11361.Contig2\_All, CL5823.Contig1\_All |
| intramolecular oxidoreductase activity | CL9060.Contig1\_All, Unigene5838\_All, CL11370.Contig1\_All, CL5077.Contig2\_All, CL8920.Contig1\_All, CL4168.Contig1\_All, CL6646.Contig2\_All, CL8539.Contig1\_All, CL6487.Contig3\_All, Unigene59822\_All, Unigene40253\_All, CL8539.Contig2\_All, Unigene31529\_All, CL11285.Contig2\_All, CL11285.Contig1\_All, CL2859.Contig2\_All, Unigene17028\_All, Unigene58843\_All, Unigene7560\_All, CL11181.Contig2\_All, CL6726.Contig1\_All, CL6726.Contig2\_All, CL879.Contig5\_All, Unigene3614\_All, CL879.Contig1\_All |
| carbohydrate transmembrane transporter activity | Unigene31375\_All, CL2349.Contig3\_All, CL4843.Contig2\_All, Unigene34157\_All, CL8464.Contig1\_All, Unigene3687\_All, Unigene4205\_All, Unigene794\_All, CL1205.Contig6\_All, Unigene26932\_All, CL8446.Contig2\_All, CL4327.Contig1\_All, CL410.Contig3\_All, Unigene19104\_All, Unigene20393\_All, Unigene30020\_All, Unigene55899\_All, CL1799.Contig3\_All, CL2702.Contig2\_All, CL2702.Contig4\_All, CL1876.Contig1\_All, Unigene16336\_All, Unigene59478\_All, Unigene20676\_All, CL12032.Contig2\_All, CL12466.Contig4\_All, CL11361.Contig1\_All, Unigene35030\_All, Unigene30500\_All, Unigene40544\_All, Unigene18811\_All, CL3285.Contig20\_All, Unigene15535\_All, Unigene60014\_All, Unigene33588\_All, Unigene13545\_All, Unigene702\_All, Unigene23920\_All, CL12466.Contig2\_All, CL9819.Contig2\_All, CL410.Contig1\_All, CL2561.Contig2\_All, CL12936.Contig3\_All, CL4725.Contig2\_All, CL2262.Contig2\_All, CL1315.Contig2\_All, CL4333.Contig3\_All, CL410.Contig2\_All, CL11361.Contig2\_All |
| carbohydrate transporter activity | Unigene31375\_All, CL2349.Contig3\_All, CL4843.Contig2\_All, Unigene34157\_All, CL8464.Contig1\_All, Unigene3687\_All, Unigene4205\_All, Unigene794\_All, CL1205.Contig6\_All, Unigene26932\_All, CL8446.Contig2\_All, CL4327.Contig1\_All, CL410.Contig3\_All, Unigene19104\_All, Unigene20393\_All, Unigene30020\_All, Unigene55899\_All, CL1799.Contig3\_All, CL2702.Contig2\_All, CL2702.Contig4\_All, CL1876.Contig1\_All, Unigene16336\_All, Unigene59478\_All, Unigene20676\_All, CL12032.Contig2\_All, CL12466.Contig4\_All, CL11361.Contig1\_All, Unigene35030\_All, Unigene30500\_All, Unigene40544\_All, Unigene18811\_All, CL3285.Contig20\_All, Unigene15535\_All, Unigene60014\_All, Unigene33588\_All, Unigene13545\_All, Unigene702\_All, Unigene23920\_All, CL12466.Contig2\_All, CL9819.Contig2\_All, CL410.Contig1\_All, CL2561.Contig2\_All, CL12936.Contig3\_All, CL4725.Contig2\_All, CL2262.Contig2\_All, CL1315.Contig2\_All, CL4333.Contig3\_All, CL410.Contig2\_All, CL11361.Contig2\_All |
| aldehyde-lyase activity | CL6022.Contig4\_All, CL5711.Contig5\_All, Unigene14125\_All, CL4908.Contig1\_All, CL10097.Contig1\_All, CL3913.Contig2\_All, Unigene11358\_All, CL1237.Contig2\_All, CL10600.Contig3\_All, CL6022.Contig2\_All, CL5711.Contig4\_All, Unigene4398\_All, CL341.Contig1\_All, CL6526.Contig1\_All, CL587.Contig5\_All |
| 1-phosphatidylinositol binding | CL7818.Contig2\_All, CL7818.Contig6\_All, CL7818.Contig5\_All, Unigene60254\_All, Unigene39526\_All, CL6434.Contig4\_All |
| amino acid binding | Unigene54840\_All, CL12021.Contig2\_All, Unigene23332\_All, CL10607.Contig3\_All, Unigene59749\_All, CL13868.Contig8\_All, Unigene59748\_All, CL171.Contig16\_All, CL201.Contig17\_All, CL2688.Contig1\_All, CL4175.Contig2\_All, CL8770.Contig4\_All, Unigene17983\_All, Unigene35538\_All, CL2688.Contig2\_All, Unigene5566\_All, CL12933.Contig1\_All, CL12972.Contig1\_All, CL12416.Contig1\_All, CL12021.Contig3\_All, Unigene54842\_All, Unigene23378\_All, CL171.Contig18\_All, Unigene59750\_All, Unigene28693\_All, Unigene24898\_All, CL9717.Contig1\_All, CL12933.Contig2\_All, Unigene13760\_All, CL171.Contig17\_All, CL201.Contig16\_All, CL13988.Contig2\_All, CL171.Contig19\_All, Unigene54841\_All, Unigene36014\_All, CL2862.Contig1\_All |
| oxidoreductase activity, acting on the CH-NH group of donors, NAD or NADP as acceptor | Unigene27257\_All, CL8472.Contig3\_All, CL911.Contig1\_All, Unigene29003\_All, CL7072.Contig1\_All, CL7072.Contig2\_All, CL5500.Contig2\_All, Unigene10055\_All, Unigene4069\_All, CL4084.Contig11\_All, Unigene26129\_All, Unigene15719\_All |
| NAD(P)H dehydrogenase (quinone) activity | Unigene27257\_All, Unigene29003\_All, Unigene4069\_All |
| fatty acid binding | Unigene29022\_All, CL9967.Contig1\_All, Unigene23030\_All |
| sodium:dicarboxylate symporter activity | CL7188.Contig1\_All, CL10085.Contig3\_All, CL10085.Contig2\_All |
| allene-oxide cyclase activity | CL11285.Contig2\_All, CL11285.Contig1\_All, Unigene7560\_All |
| 4,4-dimethyl-9beta,19-cyclopropylsterol-4alpha-methyl oxidase activity | CL683.Contig3\_All, CL683.Contig2\_All, Unigene3061\_All |
| antiporter activity | CL8933.Contig2\_All, Unigene35631\_All, CL6859.Contig2\_All, Unigene3362\_All, CL8933.Contig1\_All, CL8254.Contig2\_All, CL11788.Contig2\_All, CL11788.Contig1\_All, Unigene60373\_All, CL12925.Contig3\_All, CL4077.Contig1\_All, CL13880.Contig1\_All, CL12679.Contig4\_All, Unigene18110\_All, Unigene16784\_All, CL1946.Contig2\_All, CL3986.Contig1\_All, Unigene16763\_All, CL12679.Contig2\_All, CL1946.Contig1\_All, CL4937.Contig2\_All, Unigene18748\_All, Unigene34564\_All, CL2965.Contig2\_All, CL4472.Contig1\_All, CL12418.Contig1\_All, Unigene20393\_All, CL7499.Contig9\_All, CL9027.Contig14\_All, CL8254.Contig1\_All, Unigene55696\_All, CL9856.Contig2\_All, Unigene28965\_All, Unigene22965\_All, Unigene56928\_All, CL10764.Contig3\_All, CL14006.Contig1\_All, CL6796.Contig2\_All, CL920.Contig2\_All, CL3922.Contig1\_All, CL3281.Contig1\_All, Unigene4175\_All, CL5771.Contig3\_All, Unigene65301\_All, CL2035.Contig1\_All, CL920.Contig3\_All, Unigene22297\_All, CL10352.Contig3\_All, CL9444.Contig2\_All, CL2288.Contig1\_All, Unigene13591\_All, CL7413.Contig2\_All, CL6859.Contig1\_All, Unigene18751\_All, Unigene22762\_All, CL3986.Contig4\_All, Unigene59384\_All, CL7499.Contig6\_All, CL4077.Contig2\_All, CL3986.Contig2\_All, CL9027.Contig8\_All, CL7413.Contig1\_All |
| squalene monooxygenase activity | CL238.Contig39\_All, CL4994.Contig9\_All, CL238.Contig42\_All, CL238.Contig47\_All, CL238.Contig33\_All, CL238.Contig40\_All, CL238.Contig57\_All |
| acetate-CoA ligase activity | Unigene35795\_All, CL8940.Contig1\_All, CL5590.Contig4\_All, CL1292.Contig2\_All, CL5590.Contig3\_All, CL8940.Contig4\_All, CL5590.Contig5\_All, CL8940.Contig5\_All |
| citrate (Si)-synthase activity | Unigene34123\_All, Unigene34122\_All, CL2983.Contig3\_All, CL10159.Contig2\_All, CL2108.Contig3\_All, Unigene63714\_All, Unigene38630\_All, CL2983.Contig4\_All, Unigene34124\_All |
| galactosylxylosylprotein 3-beta-galactosyltransferase activity | CL7693.Contig1\_All, Unigene16399\_All, CL7693.Contig3\_All, CL13764.Contig1\_All, CL4521.Contig1\_All, Unigene19918\_All, CL271.Contig1\_All, CL271.Contig2\_All, Unigene42163\_All |
| mannosyltransferase activity | CL1693.Contig2\_All, Unigene9786\_All, CL1693.Contig1\_All, CL850.Contig2\_All, CL850.Contig1\_All, Unigene23382\_All, CL11911.Contig2\_All, CL4539.Contig1\_All, CL6020.Contig5\_All, CL7513.Contig2\_All, CL1693.Contig3\_All, Unigene58118\_All, CL7513.Contig1\_All, CL6020.Contig6\_All |
| cofactor binding | Unigene21226\_All, CL8737.Contig1\_All, CL5167.Contig1\_All, CL3536.Contig2\_All, CL10257.Contig2\_All, CL4805.Contig3\_All, CL8738.Contig2\_All, Unigene10484\_All, Unigene14785\_All, Unigene60701\_All, CL3610.Contig7\_All, CL6062.Contig1\_All, CL4908.Contig1\_All, CL9850.Contig4\_All, CL6119.Contig1\_All, Unigene42184\_All, Unigene39960\_All, CL514.Contig3\_All, CL1239.Contig1\_All, Unigene38690\_All, CL949.Contig1\_All, CL949.Contig3\_All, CL7843.Contig3\_All, CL238.Contig57\_All, CL2912.Contig1\_All, CL6119.Contig2\_All, CL258.Contig2\_All, Unigene34381\_All, CL7971.Contig2\_All, CL1283.Contig4\_All, CL6022.Contig2\_All, Unigene16162\_All, Unigene56835\_All, Unigene19567\_All, CL6022.Contig4\_All, CL2999.Contig1\_All, Unigene10105\_All, CL10257.Contig1\_All, Unigene54825\_All, CL9751.Contig2\_All, Unigene56757\_All, Unigene54418\_All, CL4148.Contig2\_All, Unigene47896\_All, Unigene54422\_All, CL6896.Contig2\_All, CL2120.Contig4\_All, CL1232.Contig8\_All, Unigene40707\_All, Unigene59793\_All, Unigene42278\_All, CL10289.Contig1\_All, CL3610.Contig2\_All, CL13562.Contig2\_All, CL8001.Contig1\_All, Unigene58324\_All, CL9354.Contig4\_All, CL1986.Contig2\_All, CL8389.Contig3\_All, CL4825.Contig2\_All, Unigene55119\_All, Unigene59773\_All, Unigene36489\_All, Unigene23445\_All, Unigene54321\_All, CL3993.Contig3\_All, CL2655.Contig3\_All, Unigene25365\_All, CL3574.Contig5\_All, Unigene36014\_All, CL10823.Contig2\_All, CL2777.Contig1\_All, Unigene59471\_All, Unigene38927\_All, CL3795.Contig1\_All, CL12061.Contig2\_All, Unigene912\_All, Unigene57584\_All, Unigene32589\_All, Unigene7919\_All, CL238.Contig33\_All, Unigene32418\_All, CL7100.Contig3\_All, Unigene7472\_All, CL6767.Contig1\_All, CL10195.Contig1\_All, CL98.Contig18\_All, Unigene54412\_All, Unigene3383\_All, Unigene14655\_All, Unigene31139\_All, Unigene3614\_All, Unigene42870\_All, CL12061.Contig3\_All, CL4972.Contig3\_All, CL142.Contig2\_All, CL12972.Contig1\_All, CL1425.Contig2\_All, CL5723.Contig3\_All, CL1207.Contig3\_All, CL14006.Contig1\_All, Unigene58317\_All, Unigene58117\_All, Unigene4665\_All, Unigene28560\_All, CL238.Contig39\_All, CL7896.Contig2\_All, Unigene59572\_All, CL2587.Contig1\_All, Unigene14167\_All, CL1690.Contig1\_All, CL2670.Contig1\_All, CL9850.Contig3\_All, Unigene57585\_All, CL11392.Contig2\_All, CL889.Contig2\_All, Unigene26165\_All, Unigene4398\_All, CL6526.Contig2\_All, CL923.Contig4\_All, CL8389.Contig1\_All, CL1347.Contig3\_All, CL12769.Contig1\_All, CL10234.Contig2\_All, Unigene62945\_All, CL2702.Contig2\_All, CL283.Contig5\_All, CL9892.Contig3\_All, CL5672.Contig2\_All, Unigene10799\_All, CL6062.Contig2\_All, CL8052.Contig1\_All, CL11761.Contig2\_All, CL4972.Contig1\_All, CL13390.Contig2\_All, CL4044.Contig1\_All, Unigene34380\_All, CL238.Contig40\_All, CL3925.Contig3\_All, Unigene1798\_All, Unigene3242\_All, CL5818.Contig3\_All, CL1239.Contig2\_All, Unigene11358\_All, CL10234.Contig1\_All, CL5832.Contig1\_All, CL4812.Contig3\_All, CL7971.Contig3\_All, CL12769.Contig2\_All, CL5167.Contig5\_All, CL4994.Contig9\_All, CL9128.Contig1\_All, CL7457.Contig2\_All, Unigene34737\_All, CL6005.Contig1\_All, CL12933.Contig1\_All, CL4812.Contig5\_All, Unigene3462\_All, Unigene54419\_All, Unigene60613\_All, Unigene17142\_All, CL12503.Contig2\_All, CL341.Contig1\_All, Unigene63349\_All, CL6526.Contig1\_All, Unigene56231\_All, CL1053.Contig2\_All, CL1232.Contig14\_All, Unigene12122\_All, CL3610.Contig5\_All, Unigene59986\_All, CL6062.Contig3\_All, CL6717.Contig2\_All, CL7298.Contig2\_All, CL1053.Contig1\_All, CL8738.Contig3\_All, Unigene41207\_All, CL3913.Contig2\_All, Unigene14349\_All, Unigene30252\_All, CL1288.Contig3\_All, Unigene60207\_All, Unigene29194\_All, CL2702.Contig4\_All, CL5273.Contig1\_All, CL142.Contig1\_All, CL2877.Contig1\_All, CL7896.Contig1\_All, CL6717.Contig3\_All, CL10040.Contig2\_All, CL12386.Contig1\_All, Unigene10549\_All, CL12933.Contig2\_All, CL9973.Contig3\_All, Unigene9754\_All, CL1001.Contig3\_All, Unigene26632\_All, Unigene38128\_All, Unigene32479\_All, Unigene29396\_All, Unigene57130\_All, Unigene9305\_All, Unigene7376\_All, CL1232.Contig10\_All, Unigene42744\_All, Unigene42390\_All, CL4718.Contig3\_All, CL12720.Contig2\_All, Unigene54807\_All, CL5185.Contig2\_All, CL6487.Contig3\_All, Unigene22816\_All, CL10875.Contig1\_All, Unigene34735\_All, Unigene589\_All, Unigene36907\_All, CL3894.Contig3\_All, Unigene59884\_All, Unigene17759\_All, Unigene34736\_All, Unigene22442\_All, CL1001.Contig2\_All, Unigene10725\_All, Unigene7410\_All, Unigene59694\_All, Unigene20163\_All, CL3894.Contig1\_All, CL5672.Contig1\_All, CL5285.Contig1\_All, CL4825.Contig1\_All, Unigene15289\_All, Unigene7449\_All, Unigene31261\_All, CL9128.Contig2\_All, CL1525.Contig2\_All, Unigene63707\_All, CL1288.Contig4\_All, CL6717.Contig4\_All, Unigene38488\_All, CL9645.Contig1\_All, Unigene7531\_All, CL6767.Contig2\_All, Unigene58323\_All, CL2260.Contig2\_All, CL1997.Contig2\_All, CL1232.Contig6\_All, CL1283.Contig5\_All, Unigene55021\_All, CL2688.Contig1\_All, CL923.Contig3\_All, Unigene41493\_All, CL5818.Contig2\_All, CL2688.Contig2\_All, CL238.Contig47\_All, Unigene54413\_All, CL7100.Contig2\_All, CL238.Contig42\_All, CL3296.Contig1\_All, CL3283.Contig3\_All, Unigene55183\_All, CL11392.Contig1\_All, Unigene61369\_All, CL2003.Contig6\_All, Unigene23386\_All, Unigene3440\_All |
| amide transmembrane transporter activity | Unigene30346\_All, CL3548.Contig2\_All, Unigene56095\_All, CL73.Contig3\_All, Unigene31136\_All, Unigene56646\_All, CL73.Contig2\_All, CL73.Contig1\_All, CL2225.Contig2\_All, CL8910.Contig2\_All, CL11060.Contig1\_All, Unigene56096\_All, Unigene56094\_All, Unigene56097\_All, Unigene16770\_All, Unigene23271\_All, CL2225.Contig1\_All, Unigene60226\_All |
| microtubule binding | CL10915.Contig4\_All, Unigene26386\_All, CL2730.Contig1\_All, CL13689.Contig3\_All, CL1928.Contig3\_All, Unigene19987\_All, CL9531.Contig2\_All, CL6487.Contig3\_All, Unigene57324\_All, CL1338.Contig1\_All, CL1808.Contig1\_All, CL3377.Contig3\_All, CL7597.Contig1\_All, Unigene44308\_All, CL9815.Contig1\_All, CL1808.Contig2\_All, Unigene24849\_All, Unigene56817\_All, Unigene3798\_All, Unigene61100\_All, Unigene29424\_All, CL3377.Contig2\_All, CL8239.Contig2\_All, Unigene14454\_All, CL1390.Contig1\_All, Unigene33314\_All, CL1390.Contig2\_All, Unigene19051\_All, Unigene1478\_All, Unigene57323\_All, Unigene10820\_All, Unigene58546\_All, CL13437.Contig3\_All, Unigene16768\_All, CL187.Contig2\_All, CL187.Contig1\_All, CL11491.Contig1\_All, Unigene18038\_All, CL12452.Contig2\_All, CL3377.Contig1\_All, Unigene2577\_All, Unigene11483\_All, Unigene57107\_All, Unigene58305\_All |
| poly-pyrimidine tract binding | Unigene47188\_All, CL1430.Contig13\_All, Unigene54413\_All, CL8052.Contig1\_All, CL1430.Contig4\_All, CL1430.Contig11\_All, Unigene54419\_All, CL10298.Contig1\_All, Unigene22400\_All, CL12272.Contig1\_All, Unigene54418\_All, CL6792.Contig1\_All, Unigene54422\_All, CL5285.Contig1\_All, Unigene23124\_All, Unigene54412\_All, Unigene33591\_All, Unigene33589\_All, Unigene22270\_All, CL1430.Contig6\_All, Unigene34854\_All, Unigene31261\_All |
| poly(U) RNA binding | Unigene47188\_All, CL1430.Contig13\_All, Unigene54413\_All, CL8052.Contig1\_All, CL1430.Contig4\_All, CL1430.Contig11\_All, Unigene54419\_All, CL10298.Contig1\_All, Unigene22400\_All, CL12272.Contig1\_All, Unigene54418\_All, CL6792.Contig1\_All, Unigene54422\_All, CL5285.Contig1\_All, Unigene23124\_All, Unigene54412\_All, Unigene33591\_All, Unigene33589\_All, Unigene22270\_All, CL1430.Contig6\_All, Unigene34854\_All, Unigene31261\_All |
| anthranilate synthase activity | CL187.Contig12\_All, CL187.Contig11\_All, CL5595.Contig1\_All, CL7638.Contig3\_All, CL879.Contig3\_All |
| UDP-N-acetylmuramate dehydrogenase activity | Unigene21226\_All, Unigene58324\_All, Unigene58323\_All, CL6062.Contig1\_All, CL6062.Contig2\_All, CL8737.Contig1\_All, CL1207.Contig3\_All, CL3574.Contig5\_All, CL9645.Contig1\_All, Unigene30252\_All, Unigene63349\_All, CL6062.Contig3\_All, CL5273.Contig1\_All, Unigene4665\_All |
| cadmium ion transmembrane transporter activity | CL807.Contig4\_All, CL807.Contig5\_All, Unigene30755\_All, CL11162.Contig3\_All, CL13951.Contig1\_All, CL2989.Contig3\_All, Unigene12030\_All, CL12157.Contig12\_All, Unigene60246\_All, Unigene38910\_All |
| isomerase activity | CL10109.Contig2\_All, Unigene58609\_All, Unigene12475\_All, CL12949.Contig25\_All, Unigene20690\_All, CL12261.Contig1\_All, CL5134.Contig9\_All, CL7411.Contig2\_All, Unigene37882\_All, CL7505.Contig3\_All, Unigene15863\_All, CL8539.Contig1\_All, CL13317.Contig2\_All, Unigene14785\_All, Unigene36062\_All, Unigene57719\_All, CL11181.Contig2\_All, Unigene60476\_All, CL2115.Contig4\_All, Unigene19067\_All, Unigene18047\_All, CL11800.Contig3\_All, Unigene34428\_All, Unigene12275\_All, CL2992.Contig16\_All, CL949.Contig1\_All, Unigene7134\_All, CL949.Contig3\_All, CL4168.Contig1\_All, Unigene27776\_All, CL12043.Contig1\_All, Unigene61476\_All, Unigene34381\_All, Unigene11907\_All, Unigene58843\_All, CL9326.Contig2\_All, Unigene59986\_All, Unigene16162\_All, Unigene56835\_All, Unigene19567\_All, Unigene61059\_All, Unigene40910\_All, CL645.Contig2\_All, CL7059.Contig1\_All, Unigene47896\_All, Unigene32386\_All, Unigene4935\_All, CL2434.Contig1\_All, Unigene25149\_All, Unigene40707\_All, Unigene42278\_All, CL6726.Contig2\_All, CL879.Contig5\_All, CL4508.Contig1\_All, CL4128.Contig1\_All, CL13562.Contig2\_All, CL8001.Contig1\_All, CL2992.Contig12\_All, CL4214.Contig2\_All, CL13951.Contig1\_All, CL9354.Contig4\_All, CL1986.Contig2\_All, CL1752.Contig1\_All, Unigene54594\_All, Unigene33138\_All, CL3496.Contig5\_All, CL9967.Contig1\_All, Unigene39843\_All, Unigene63708\_All, CL6726.Contig1\_All, Unigene33013\_All, Unigene2709\_All, Unigene14456\_All, CL2777.Contig1\_All, CL5047.Contig1\_All, Unigene47262\_All, Unigene35230\_All, CL5134.Contig8\_All, Unigene2026\_All, CL4128.Contig2\_All, CL6646.Contig2\_All, CL6487.Contig3\_All, Unigene29621\_All, Unigene42029\_All, Unigene34977\_All, Unigene59822\_All, Unigene61185\_All, Unigene9161\_All, CL4798.Contig2\_All, Unigene42541\_All, Unigene22166\_All, CL8539.Contig2\_All, Unigene31529\_All, Unigene61165\_All, CL6767.Contig1\_All, Unigene61417\_All, CL1027.Contig1\_All, Unigene32323\_All, Unigene14655\_All, Unigene3614\_All, CL4972.Contig3\_All, CL9060.Contig1\_All, Unigene39542\_All, CL13373.Contig1\_All, CL8474.Contig2\_All, CL599.Contig2\_All, Unigene40241\_All, Unigene17028\_All, CL807.Contig5\_All, Unigene7560\_All, Unigene23273\_All, Unigene29022\_All, Unigene61044\_All, CL879.Contig1\_All, Unigene5838\_All, Unigene42013\_All, Unigene13828\_All, Unigene5950\_All, CL8920.Contig1\_All, CL4798.Contig1\_All, CL13837.Contig2\_All, Unigene40253\_All, CL6767.Contig2\_All, CL3339.Contig2\_All, CL11285.Contig1\_All, CL2260.Contig2\_All, CL12805.Contig2\_All, Unigene63943\_All, Unigene11024\_All, CL13317.Contig3\_All, Unigene27985\_All, Unigene37471\_All, CL11370.Contig1\_All, CL5077.Contig2\_All, CL145.Contig1\_All, CL4972.Contig1\_All, Unigene62270\_All, CL3496.Contig3\_All, Unigene11900\_All, CL3496.Contig1\_All, CL3339.Contig1\_All, Unigene32188\_All, CL11285.Contig2\_All, CL2859.Contig2\_All, Unigene34380\_All, CL4299.Contig2\_All, Unigene38874\_All, Unigene58406\_All, CL9913.Contig1\_All, CL13851.Contig1\_All, CL8418.Contig1\_All, Unigene9027\_All, Unigene36422\_All, Unigene39158\_All, Unigene3440\_All |
| peptidase inhibitor activity | CL8559.Contig1\_All, Unigene41946\_All, Unigene36095\_All, CL1005.Contig2\_All, Unigene12387\_All, CL1005.Contig1\_All, CL8559.Contig2\_All, CL12131.Contig2\_All, CL2022.Contig1\_All |
| peptidase regulator activity | CL8559.Contig1\_All, Unigene41946\_All, Unigene36095\_All, CL1005.Contig2\_All, Unigene12387\_All, CL1005.Contig1\_All, CL8559.Contig2\_All, CL12131.Contig2\_All, CL2022.Contig1\_All |
| alcohol dehydrogenase (NAD) activity | CL5178.Contig6\_All, CL5178.Contig4\_All, CL1283.Contig5\_All, CL5178.Contig2\_All, CL5178.Contig5\_All, CL5178.Contig3\_All, CL1283.Contig4\_All |
| sodium:hydrogen antiporter activity | CL3986.Contig4\_All, Unigene59384\_All, Unigene22965\_All, CL7413.Contig1\_All, Unigene22297\_All, Unigene16784\_All, CL7413.Contig2\_All |
| ATP:ADP antiporter activity | Unigene28965\_All, CL2288.Contig1\_All, Unigene3362\_All, Unigene60373\_All, CL14006.Contig1\_All, CL6796.Contig2\_All, Unigene4175\_All, CL2035.Contig1\_All |
| sulfotransferase activity | CL4949.Contig2\_All, CL1525.Contig2\_All, CL4867.Contig1\_All, CL4949.Contig1\_All, Unigene61954\_All, CL7781.Contig1\_All, Unigene1566\_All, CL269.Contig2\_All |
| urea transmembrane transporter activity | CL11060.Contig1\_All, Unigene30346\_All, CL3548.Contig2\_All, CL73.Contig3\_All, Unigene31136\_All, Unigene23271\_All, CL73.Contig2\_All, CL73.Contig1\_All |
| transferase activity, transferring hexosyl groups | Unigene58478\_All, CL9785.Contig1\_All, Unigene46080\_All, CL10861.Contig5\_All, Unigene40045\_All, CL6020.Contig5\_All, CL1926.Contig4\_All, Unigene20159\_All, CL3233.Contig4\_All, CL2059.Contig3\_All, CL8038.Contig3\_All, CL4521.Contig1\_All, CL13317.Contig2\_All, Unigene25173\_All, CL4649.Contig6\_All, Unigene56167\_All, Unigene5078\_All, Unigene61193\_All, CL3782.Contig5\_All, Unigene6187\_All, Unigene6872\_All, Unigene58066\_All, CL3077.Contig1\_All, CL7937.Contig1\_All, CL10317.Contig1\_All, CL11441.Contig1\_All, CL1360.Contig7\_All, CL13575.Contig6\_All, CL4017.Contig1\_All, Unigene12860\_All, CL7664.Contig3\_All, CL5518.Contig2\_All, CL10693.Contig3\_All, Unigene34428\_All, CL5140.Contig2\_All, CL203.Contig26\_All, Unigene9784\_All, CL3301.Contig2\_All, CL4539.Contig1\_All, Unigene60613\_All, CL12109.Contig2\_All, CL2468.Contig2\_All, Unigene21952\_All, CL4168.Contig1\_All, CL325.Contig1\_All, CL2912.Contig1\_All, Unigene43365\_All, Unigene60528\_All, CL850.Contig1\_All, Unigene59961\_All, CL8912.Contig1\_All, Unigene58373\_All, Unigene2958\_All, CL13764.Contig1\_All, Unigene28526\_All, CL2468.Contig1\_All, CL4252.Contig3\_All, Unigene17463\_All, Unigene9786\_All, CL203.Contig23\_All, CL7693.Contig1\_All, CL3301.Contig1\_All, CL413.Contig3\_All, Unigene23382\_All, CL11911.Contig2\_All, Unigene6103\_All, Unigene23183\_All, CL388.Contig1\_All, Unigene57682\_All, CL3660.Contig9\_All, Unigene27662\_All, Unigene27669\_All, CL271.Contig2\_All, Unigene7123\_All, CL1693.Contig1\_All, CL438.Contig3\_All, CL12780.Contig1\_All, CL203.Contig6\_All, Unigene19918\_All, CL5355.Contig1\_All, Unigene60059\_All, Unigene57259\_All, CL203.Contig24\_All, CL7513.Contig2\_All, CL9662.Contig2\_All, Unigene23145\_All, CL203.Contig28\_All, CL4508.Contig1\_All, Unigene61130\_All, CL1151.Contig3\_All, Unigene429\_All, Unigene26143\_All, Unigene35304\_All, Unigene10100\_All, Unigene61081\_All, CL7664.Contig1\_All, CL3077.Contig2\_All, CL1743.Contig3\_All, Unigene19340\_All, Unigene58118\_All, CL3233.Contig5\_All, CL3381.Contig2\_All, Unigene54594\_All, Unigene60259\_All, Unigene4765\_All, CL413.Contig1\_All, Unigene59737\_All, CL10693.Contig1\_All, CL3300.Contig2\_All, CL13575.Contig5\_All, Unigene5803\_All, CL3300.Contig1\_All, Unigene57130\_All, Unigene36014\_All, Unigene15989\_All, CL2746.Contig1\_All, CL2029.Contig7\_All, CL10317.Contig2\_All, Unigene32598\_All, Unigene14060\_All, Unigene33166\_All, CL4649.Contig13\_All, Unigene4614\_All, Unigene35230\_All, Unigene8706\_All, Unigene18469\_All, Unigene29621\_All, CL4274.Contig1\_All, Unigene22307\_All, CL3765.Contig1\_All, CL1693.Contig2\_All, Unigene27367\_All, Unigene1016\_All, CL1842.Contig1\_All, CL203.Contig15\_All, CL271.Contig1\_All, CL9662.Contig1\_All, CL8038.Contig2\_All, Unigene8864\_All, CL3883.Contig4\_All, Unigene24631\_All, CL12109.Contig1\_All, CL7258.Contig3\_All, CL9785.Contig2\_All, CL2746.Contig5\_All, Unigene33783\_All, CL2604.Contig2\_All, Unigene569\_All, Unigene60247\_All, CL10962.Contig1\_All, CL1743.Contig4\_All, CL12476.Contig2\_All, Unigene42368\_All, CL11228.Contig1\_All, Unigene7605\_All, Unigene37487\_All, CL10962.Contig2\_All, Unigene15775\_All, CL2618.Contig3\_All, Unigene58479\_All, Unigene45197\_All, CL3898.Contig2\_All, CL7664.Contig2\_All, CL3233.Contig1\_All, Unigene27246\_All, CL2574.Contig2\_All, CL2574.Contig1\_All, Unigene40018\_All, Unigene60855\_All, Unigene58912\_All, CL3301.Contig4\_All, CL3217.Contig4\_All, CL1872.Contig1\_All, Unigene32595\_All, CL10931.Contig2\_All, CL7693.Contig3\_All, CL1693.Contig3\_All, Unigene24864\_All, CL7513.Contig1\_All, Unigene59129\_All, CL3883.Contig2\_All, Unigene33741\_All, CL4649.Contig1\_All, CL3355.Contig37\_All, CL10800.Contig3\_All, Unigene34308\_All, Unigene23131\_All, CL11330.Contig2\_All, CL8395.Contig2\_All, CL4649.Contig10\_All, Unigene39364\_All, CL11228.Contig2\_All, CL3233.Contig6\_All, CL3301.Contig3\_All, CL220.Contig2\_All, CL13317.Contig3\_All, CL4708.Contig2\_All, CL6433.Contig2\_All, CL7600.Contig2\_All, Unigene62466\_All, CL850.Contig2\_All, CL7258.Contig1\_All, Unigene602\_All, Unigene16399\_All, CL2618.Contig2\_All, CL10931.Contig1\_All, CL3660.Contig7\_All, CL9132.Contig34\_All, Unigene30672\_All, CL1360.Contig4\_All, CL7391.Contig2\_All, Unigene57875\_All, CL2029.Contig11\_All, CL4274.Contig2\_All, CL1360.Contig8\_All, CL3301.Contig5\_All, Unigene31503\_All, CL8143.Contig1\_All, Unigene31185\_All, Unigene42163\_All, Unigene1798\_All, CL3381.Contig3\_All, Unigene6871\_All, CL10366.Contig1\_All, CL12968.Contig5\_All, CL6020.Contig6\_All, CL8418.Contig1\_All |
| phosphoribosylformylglycinamidine cyclo-ligase activity | CL8458.Contig1\_All, CL7902.Contig1\_All, CL4011.Contig2\_All, CL4011.Contig1\_All |
| G-protein coupled receptor kinase activity | CL379.Contig1\_All, Unigene10688\_All, CL6263.Contig2\_All, Unigene58820\_All |
| phospholipase activator activity | CL967.Contig7\_All, CL967.Contig8\_All, CL967.Contig6\_All, Unigene17052\_All |
| cinnamoyl-CoA reductase activity | Unigene18047\_All, Unigene61417\_All, Unigene56835\_All, Unigene4557\_All |
| aspartate-prephenate aminotransferase activity | CL12061.Contig2\_All, CL4718.Contig3\_All, CL10823.Contig2\_All, CL12061.Contig3\_All |
| glutamate-prephenate aminotransferase activity | CL12061.Contig2\_All, CL4718.Contig3\_All, CL10823.Contig2\_All, CL12061.Contig3\_All |
| omega-3 fatty acid desaturase activity | CL8195.Contig2\_All, CL8523.Contig1\_All, Unigene9422\_All, Unigene7124\_All |
| lipase activator activity | CL967.Contig7\_All, CL967.Contig8\_All, CL967.Contig6\_All, Unigene17052\_All |
| 3R-hydroxyacyl-CoA dehydratase activity | Unigene29888\_All, CL4440.Contig1\_All, CL10010.Contig1\_All, CL4440.Contig2\_All |
| polygalacturonase activity | CL4361.Contig3\_All, Unigene20634\_All, Unigene4204\_All, Unigene20145\_All, Unigene54925\_All, Unigene10260\_All, Unigene6838\_All, Unigene13736\_All, CL4361.Contig2\_All, CL7842.Contig2\_All, CL4361.Contig1\_All, Unigene139\_All, Unigene6944\_All, Unigene20392\_All |
| organic acid:sodium symporter activity | Unigene18860\_All, CL817.Contig4\_All, CL7188.Contig1\_All, CL10085.Contig3\_All, Unigene61494\_All, CL1760.Contig3\_All, Unigene43692\_All, Unigene60257\_All, CL10085.Contig2\_All, Unigene59589\_All |
| active transmembrane transporter activity | CL5593.Contig4\_All, Unigene47188\_All, CL6859.Contig2\_All, CL10864.Contig2\_All, CL3689.Contig6\_All, CL479.Contig1\_All, CL13880.Contig1\_All, CL2436.Contig2\_All, CL649.Contig1\_All, CL6201.Contig2\_All, CL1205.Contig6\_All, CL3986.Contig1\_All, CL12679.Contig2\_All, Unigene26745\_All, Unigene51017\_All, Unigene60321\_All, Unigene16770\_All, Unigene17982\_All, Unigene58417\_All, Unigene23271\_All, Unigene29887\_All, Unigene24785\_All, CL2750.Contig3\_All, CL1876.Contig1\_All, Unigene384\_All, CL12683.Contig2\_All, Unigene34172\_All, Unigene59478\_All, Unigene28965\_All, CL1430.Contig11\_All, CL6796.Contig2\_All, CL10864.Contig1\_All, Unigene60246\_All, CL3281.Contig1\_All, Unigene40544\_All, Unigene26098\_All, Unigene22297\_All, Unigene56097\_All, CL10085.Contig3\_All, Unigene22762\_All, Unigene33589\_All, CL1430.Contig6\_All, CL3074.Contig2\_All, CL10994.Contig1\_All, Unigene39613\_All, CL8933.Contig1\_All, CL12925.Contig3\_All, Unigene6405\_All, CL2967.Contig2\_All, Unigene16763\_All, Unigene62513\_All, CL5309.Contig1\_All, Unigene56094\_All, CL7581.Contig3\_All, CL3689.Contig4\_All, Unigene9943\_All, CL4773.Contig2\_All, CL7499.Contig9\_All, CL2129.Contig2\_All, Unigene13422\_All, CL10910.Contig1\_All, CL5375.Contig3\_All, CL4360.Contig1\_All, CL10764.Contig3\_All, CL1760.Contig3\_All, Unigene55233\_All, Unigene20755\_All, CL588.Contig4\_All, CL604.Contig1\_All, CL8287.Contig1\_All, Unigene4175\_All, Unigene38112\_All, Unigene15535\_All, CL12439.Contig1\_All, Unigene57960\_All, Unigene56096\_All, CL1699.Contig1\_All, Unigene45125\_All, CL2967.Contig3\_All, CL12936.Contig3\_All, Unigene14619\_All, CL10085.Contig2\_All, CL4684.Contig2\_All, CL8933.Contig2\_All, Unigene35631\_All, CL5220.Contig13\_All, Unigene34157\_All, Unigene31329\_All, Unigene46152\_All, CL73.Contig3\_All, CL11788.Contig1\_All, Unigene56646\_All, Unigene4205\_All, CL6201.Contig4\_All, Unigene26932\_All, Unigene42059\_All, CL10296.Contig2\_All, Unigene8561\_All, CL4687.Contig2\_All, Unigene376\_All, CL1442.Contig5\_All, CL8254.Contig1\_All, CL5220.Contig6\_All, Unigene59649\_All, Unigene55203\_All, CL11278.Contig1\_All, CL2989.Contig3\_All, CL8130.Contig1\_All, CL14006.Contig1\_All, Unigene30500\_All, CL73.Contig1\_All, CL6352.Contig6\_All, CL10557.Contig1\_All, CL5736.Contig1\_All, CL206.Contig1\_All, Unigene36211\_All, CL807.Contig5\_All, Unigene11481\_All, CL2288.Contig1\_All, Unigene13591\_All, CL7413.Contig2\_All, CL5220.Contig9\_All, CL4725.Contig2\_All, Unigene59930\_All, Unigene63929\_All, CL3986.Contig2\_All, CL8444.Contig1\_All, Unigene56095\_All, CL8464.Contig1\_All, CL73.Contig2\_All, CL4247.Contig6\_All, Unigene41405\_All, Unigene17859\_All, CL8446.Contig2\_All, Unigene24061\_All, CL4937.Contig2\_All, Unigene29618\_All, CL12683.Contig1\_All, CL2702.Contig2\_All, Unigene31237\_All, CL4481.Contig2\_All, CL2960.Contig1\_All, CL1430.Contig13\_All, Unigene54678\_All, Unigene56928\_All, Unigene22965\_All, Unigene60803\_All, CL10298.Contig1\_All, Unigene40408\_All, CL920.Contig3\_All, Unigene18860\_All, Unigene60\_All, CL9819.Contig2\_All, CL6859.Contig1\_All, CL479.Contig3\_All, Unigene33591\_All, CL1113.Contig2\_All, CL2225.Contig1\_All, CL9027.Contig8\_All, CL11361.Contig2\_All, CL8444.Contig2\_All, Unigene31375\_All, CL479.Contig6\_All, CL2533.Contig4\_All, Unigene59760\_All, Unigene3362\_All, CL11382.Contig2\_All, Unigene56878\_All, Unigene35294\_All, Unigene60373\_All, Unigene31402\_All, Unigene29861\_All, CL2521.Contig16\_All, CL2521.Contig9\_All, CL5220.Contig5\_All, Unigene34564\_All, CL2965.Contig2\_All, Unigene23529\_All, CL12418.Contig1\_All, Unigene59521\_All, Unigene55696\_All, CL10307.Contig1\_All, CL479.Contig5\_All, Unigene25741\_All, Unigene16336\_All, Unigene55727\_All, Unigene63864\_All, Unigene61494\_All, CL3975.Contig2\_All, CL920.Contig2\_All, Unigene150\_All, CL5771.Contig3\_All, Unigene58549\_All, CL9444.Contig2\_All, Unigene57189\_All, CL1103.Contig4\_All, Unigene38910\_All, Unigene3794\_All, Unigene20143\_All, CL6921.Contig1\_All, Unigene7976\_All, CL1430.Contig4\_All, CL7625.Contig1\_All, CL11788.Contig2\_All, CL9706.Contig7\_All, CL361.Contig5\_All, Unigene35836\_All, CL1946.Contig1\_All, CL308.Contig6\_All, Unigene29863\_All, Unigene12613\_All, CL6352.Contig5\_All, Unigene63073\_All, CL13986.Contig5\_All, Unigene60257\_All, CL3222.Contig1\_All, CL3405.Contig5\_All, CL2702.Contig4\_All, Unigene55451\_All, Unigene35383\_All, Unigene37012\_All, Unigene30755\_All, CL13951.Contig1\_All, CL12466.Contig4\_All, Unigene55976\_All, CL10352.Contig3\_All, CL3793.Contig1\_All, CL4336.Contig3\_All, CL807.Contig4\_All, CL3222.Contig2\_All, Unigene57701\_All, CL6201.Contig3\_All, Unigene59384\_All, CL7499.Contig6\_All, CL7413.Contig1\_All, CL3742.Contig5\_All, CL4789.Contig1\_All, Unigene55085\_All, Unigene47893\_All, CL5220.Contig1\_All, Unigene57402\_All, Unigene3687\_All, CL2225.Contig2\_All, CL9399.Contig1\_All, Unigene253\_All, CL410.Contig3\_All, CL4472.Contig1\_All, CL259.Contig4\_All, CL10845.Contig18\_All, CL4789.Contig2\_All, CL9027.Contig14\_All, CL479.Contig4\_All, CL604.Contig7\_All, CL12365.Contig6\_All, CL9856.Contig2\_All, Unigene28694\_All, CL5678.Contig2\_All, CL817.Contig4\_All, CL6812.Contig1\_All, Unigene41679\_All, Unigene58923\_All, CL3922.Contig1\_All, CL259.Contig2\_All, Unigene1952\_All, CL2035.Contig1\_All, Unigene20789\_All, Unigene65301\_All, CL3285.Contig20\_All, CL2533.Contig3\_All, Unigene60014\_All, CL9399.Contig2\_All, CL3622.Contig2\_All, CL410.Contig1\_All, Unigene18751\_All, CL4773.Contig1\_All, CL4077.Contig2\_All, Unigene60226\_All, Unigene17765\_All, CL11162.Contig3\_All, CL7188.Contig1\_All, CL1024.Contig1\_All, CL9948.Contig1\_All, CL8254.Contig2\_All, Unigene32233\_All, CL10122.Contig1\_All, CL12272.Contig1\_All, Unigene9829\_All, Unigene26229\_All, CL479.Contig2\_All, CL4077.Contig1\_All, CL12679.Contig4\_All, Unigene18110\_All, Unigene16784\_All, CL8910.Contig2\_All, CL1946.Contig2\_All, Unigene18929\_All, CL270.Contig13\_All, Unigene18748\_All, Unigene2876\_All, Unigene932\_All, CL7581.Contig1\_All, Unigene43692\_All, Unigene20393\_All, Unigene30020\_All, CL9614.Contig1\_All, CL12548.Contig1\_All, Unigene2656\_All, CL11513.Contig3\_All, CL12607.Contig2\_All, CL6201.Contig1\_All, Unigene43841\_All, CL10557.Contig2\_All, Unigene22400\_All, CL11361.Contig1\_All, Unigene62270\_All, Unigene30524\_All, CL649.Contig3\_All, Unigene58511\_All, CL12548.Contig2\_All, CL12466.Contig2\_All, CL2561.Contig2\_All, CL13989.Contig2\_All, CL3986.Contig4\_All, Unigene34504\_All, CL410.Contig2\_All, Unigene59589\_All |
| oxo-acid-lyase activity | CL187.Contig12\_All, CL7912.Contig3\_All, CL187.Contig11\_All, CL7638.Contig3\_All, CL10529.Contig1\_All, CL879.Contig3\_All, CL727.Contig1\_All, CL2115.Contig4\_All, Unigene1173\_All, CL5595.Contig1\_All, CL10900.Contig2\_All, Unigene35667\_All, CL6884.Contig1\_All, CL187.Contig8\_All, Unigene35668\_All |
| UDP-glucose 4-epimerase activity | Unigene34381\_All, Unigene34380\_All, CL9354.Contig4\_All, Unigene59986\_All, Unigene40707\_All, Unigene42278\_All, Unigene19567\_All, Unigene39158\_All, Unigene3440\_All |
| protochlorophyllide reductase activity | Unigene55745\_All, CL9639.Contig2\_All, CL5710.Contig2\_All, CL9639.Contig1\_All, Unigene58150\_All, Unigene4846\_All, Unigene57674\_All, Unigene12455\_All, Unigene565\_All |
| carbamoyl-phosphate synthase (glutamine-hydrolyzing) activity | Unigene10263\_All, CL7504.Contig4\_All, CL3790.Contig2\_All, CL7504.Contig1\_All, CL3790.Contig4\_All |
| naringenin 3-dioxygenase activity | CL194.Contig3\_All, Unigene42793\_All, CL11742.Contig1\_All, CL6502.Contig1\_All, CL2757.Contig3\_All |
| solute:cation symporter activity | Unigene31375\_All, Unigene34157\_All, CL7188.Contig1\_All, CL8464.Contig1\_All, CL3689.Contig6\_All, Unigene3687\_All, Unigene4205\_All, CL1205.Contig6\_All, Unigene26932\_All, CL8446.Contig2\_All, Unigene932\_All, CL7581.Contig3\_All, CL410.Contig3\_All, Unigene63073\_All, CL7581.Contig1\_All, CL3689.Contig4\_All, Unigene43692\_All, Unigene60257\_All, Unigene30020\_All, CL9614.Contig1\_All, CL2702.Contig2\_All, CL2702.Contig4\_All, CL1876.Contig1\_All, Unigene16336\_All, Unigene34172\_All, Unigene59478\_All, CL817.Contig4\_All, CL12466.Contig4\_All, Unigene61494\_All, Unigene55976\_All, CL1760.Contig3\_All, CL11361.Contig1\_All, Unigene30500\_All, Unigene40544\_All, CL3285.Contig20\_All, Unigene15535\_All, Unigene60014\_All, Unigene57960\_All, Unigene18860\_All, CL10085.Contig3\_All, CL12466.Contig2\_All, CL9819.Contig2\_All, CL410.Contig1\_All, CL12936.Contig3\_All, CL2561.Contig2\_All, CL4725.Contig2\_All, CL10085.Contig2\_All, CL410.Contig2\_All, Unigene59589\_All, CL11361.Contig2\_All |
| methyl indole-3-acetate esterase activity | CL13215.Contig2\_All, CL12119.Contig2\_All, CL9309.Contig2\_All, Unigene32360\_All, Unigene22384\_All, CL9188.Contig1\_All, CL3442.Contig5\_All, CL9118.Contig1\_All |
| protease binding | CL603.Contig7\_All, Unigene20143\_All, CL11965.Contig3\_All, CL3480.Contig3\_All, Unigene35278\_All, CL12607.Contig2\_All |
| catalase activity | CL173.Contig17\_All, CL7181.Contig4\_All, CL5511.Contig4\_All, Unigene40276\_All, Unigene29002\_All, CL5138.Contig1\_All, CL173.Contig11\_All, CL173.Contig14\_All, CL173.Contig44\_All, Unigene55746\_All, CL173.Contig24\_All, CL173.Contig35\_All, CL11167.Contig1\_All, CL7181.Contig2\_All, CL7181.Contig5\_All, CL5138.Contig4\_All |
| chlorophyll binding | CL1384.Contig4\_All, CL3429.Contig2\_All, CL8347.Contig2\_All, CL8347.Contig1\_All, CL213.Contig7\_All, CL3429.Contig1\_All, CL10025.Contig1\_All, CL1384.Contig1\_All, CL213.Contig8\_All, Unigene32218\_All, CL213.Contig2\_All |
| 3-dehydroquinate dehydratase activity | CL1053.Contig1\_All, CL1053.Contig2\_All, Unigene912\_All |
| adenylylsulfate kinase activity | CL7447.Contig1\_All, Unigene61560\_All, CL1326.Contig2\_All |
| fumarate hydratase activity | CL8838.Contig2\_All, CL783.Contig4\_All, CL783.Contig2\_All |
| isocitrate lyase activity | Unigene1173\_All, CL7912.Contig3\_All, CL6884.Contig1\_All |
| eukaryotic translation initiation factor 2alpha kinase activity | Unigene55957\_All, CL3286.Contig2\_All, CL3286.Contig1\_All |
| shikimate 3-dehydrogenase (NADP+) activity | CL1053.Contig1\_All, CL1053.Contig2\_All, Unigene912\_All |
| palmitoyl-(protein) hydrolase activity | CL1260.Contig3\_All, CL1260.Contig2\_All, CL1260.Contig1\_All |
| auxin influx transmembrane transporter activity | CL13509.Contig2\_All, Unigene29618\_All, Unigene26098\_All |
| lupeol synthase activity | CL807.Contig5\_All, CL13951.Contig1\_All, Unigene61185\_All |
| pyruvate, phosphate dikinase activity | Unigene43846\_All, CL12103.Contig2\_All, CL12103.Contig1\_All |
| tubulin binding | CL10915.Contig4\_All, Unigene26386\_All, CL2730.Contig1\_All, CL13689.Contig3\_All, CL1928.Contig3\_All, Unigene19987\_All, CL9531.Contig2\_All, CL6487.Contig3\_All, Unigene57324\_All, CL1338.Contig1\_All, CL1808.Contig1\_All, CL3377.Contig3\_All, CL7597.Contig1\_All, Unigene44308\_All, CL9815.Contig1\_All, Unigene23555\_All, CL1808.Contig2\_All, Unigene24849\_All, Unigene56817\_All, Unigene3798\_All, Unigene61100\_All, Unigene29424\_All, CL10118.Contig1\_All, CL3377.Contig2\_All, CL8239.Contig2\_All, Unigene14454\_All, CL1390.Contig1\_All, Unigene33314\_All, CL1390.Contig2\_All, Unigene19051\_All, Unigene1478\_All, Unigene10820\_All, Unigene57323\_All, Unigene58546\_All, CL13437.Contig3\_All, Unigene16768\_All, CL187.Contig2\_All, CL187.Contig1\_All, CL11491.Contig1\_All, Unigene18038\_All, CL12452.Contig2\_All, CL3377.Contig1\_All, Unigene2577\_All, Unigene11483\_All, CL10118.Contig2\_All, Unigene58305\_All, Unigene57107\_All |
| solute:sodium symporter activity | Unigene18860\_All, CL817.Contig4\_All, CL7188.Contig1\_All, CL10085.Contig3\_All, Unigene61494\_All, CL1760.Contig3\_All, Unigene43692\_All, Unigene60257\_All, CL10085.Contig2\_All, Unigene59589\_All |
| symporter activity | Unigene31375\_All, Unigene34157\_All, CL7188.Contig1\_All, CL8464.Contig1\_All, CL3689.Contig6\_All, Unigene3687\_All, Unigene4205\_All, CL1205.Contig6\_All, Unigene26932\_All, CL8446.Contig2\_All, Unigene932\_All, Unigene29618\_All, CL7581.Contig3\_All, CL410.Contig3\_All, Unigene63073\_All, CL7581.Contig1\_All, CL3689.Contig4\_All, Unigene43692\_All, Unigene60257\_All, Unigene30020\_All, CL9614.Contig1\_All, CL2702.Contig2\_All, CL2702.Contig4\_All, CL1876.Contig1\_All, Unigene16336\_All, Unigene34172\_All, Unigene59478\_All, CL817.Contig4\_All, CL12466.Contig4\_All, Unigene61494\_All, Unigene55976\_All, CL1760.Contig3\_All, CL588.Contig4\_All, CL11361.Contig1\_All, Unigene30500\_All, Unigene26098\_All, Unigene40544\_All, CL3285.Contig20\_All, Unigene15535\_All, Unigene60014\_All, Unigene57960\_All, Unigene18860\_All, CL10085.Contig3\_All, CL12466.Contig2\_All, CL9819.Contig2\_All, CL410.Contig1\_All, CL12936.Contig3\_All, CL2561.Contig2\_All, CL4725.Contig2\_All, CL10085.Contig2\_All, CL410.Contig2\_All, Unigene59589\_All, CL11361.Contig2\_All |
| oxidoreductase activity, acting on the CH-NH group of donors | CL3014.Contig2\_All, Unigene58324\_All, Unigene27257\_All, CL8472.Contig3\_All, Unigene29003\_All, CL7072.Contig1\_All, CL7072.Contig2\_All, CL4084.Contig11\_All, Unigene15719\_All, Unigene58323\_All, CL911.Contig1\_All, Unigene56098\_All, CL13997.Contig1\_All, CL5500.Contig2\_All, Unigene10055\_All, CL13997.Contig3\_All, Unigene4069\_All, Unigene26129\_All |
| ADP binding | Unigene58600\_All, Unigene56594\_All, CL1084.Contig3\_All, Unigene24238\_All, Unigene33541\_All, CL1084.Contig4\_All, CL97.Contig5\_All, Unigene291\_All, Unigene39865\_All, Unigene58791\_All, Unigene27556\_All, Unigene41209\_All, Unigene61047\_All, CL1706.Contig1\_All, CL3414.Contig3\_All, Unigene58223\_All, Unigene58464\_All, CL105.Contig47\_All |
| intramolecular oxidoreductase activity, interconverting aldoses and ketoses | Unigene40253\_All, Unigene31529\_All, Unigene5838\_All, CL11370.Contig1\_All, CL5077.Contig2\_All, CL11181.Contig2\_All, CL8920.Contig1\_All, CL6646.Contig2\_All, CL879.Contig5\_All, Unigene59822\_All, Unigene3614\_All, CL879.Contig1\_All |
| nucleoside diphosphate kinase activity | CL8151.Contig2\_All, Unigene12506\_All, Unigene2048\_All, Unigene42090\_All, Unigene40096\_All, CL8151.Contig1\_All, CL8905.Contig1\_All, CL9942.Contig1\_All, Unigene23221\_All |
| adenyl-nucleotide exchange factor activity | CL10654.Contig2\_All, CL10654.Contig1\_All |
| DNA (6-4) photolyase activity | CL8914.Contig1\_All, CL10128.Contig2\_All |
| N4-(beta-N-acetylglucosaminyl)-L-asparaginase activity | CL12027.Contig2\_All, CL12027.Contig3\_All |
| aspartate-semialdehyde dehydrogenase activity | Unigene3242\_All, Unigene58317\_All |
| cystathionine beta-lyase activity | CL9601.Contig1\_All, Unigene38624\_All |
| cytidine deaminase activity | CL11579.Contig2\_All, Unigene10417\_All |
| 4-alpha-glucanotransferase activity | Unigene32595\_All, Unigene24631\_All |
| diphosphomevalonate decarboxylase activity | CL6222.Contig2\_All, CL6222.Contig1\_All |
| saccharopine dehydrogenase activity | CL4084.Contig11\_All, Unigene26129\_All |
| phosphatidylinositol-3,4,5-trisphosphate binding | Unigene3798\_All, CL9531.Contig2\_All |
| tRNA guanylyltransferase activity | CL3612.Contig4\_All, CL3612.Contig1\_All |
| red or far-red light photoreceptor activity | Unigene3569\_All, Unigene27060\_All |
| carotene 7,8-desaturase activity | CL11002.Contig1\_All, CL11002.Contig2\_All |
| ubiquitin conjugating enzyme binding | Unigene33925\_All, Unigene41355\_All |
| aldehyde dehydrogenase (NADP+) activity | Unigene4590\_All, CL10120.Contig1\_All |
| ribonuclease T2 activity | Unigene19750\_All, CL5540.Contig1\_All |
| annealing helicase activity | CL11321.Contig2\_All, CL11321.Contig1\_All |
| histone kinase activity (H3-S28 specific) | CL9134.Contig1\_All, Unigene57733\_All |
| protein binding involved in protein folding | Unigene29769\_All, Unigene5511\_All |
| small protein conjugating enzyme binding | Unigene33925\_All, Unigene41355\_All |
| acyl-[acyl-carrier-protein] desaturase activity | CL5008.Contig3\_All, CL5008.Contig1\_All |
| delta3,5-delta2,4-dienoyl-CoA isomerase activity | CL8539.Contig2\_All, CL8539.Contig1\_All |
| mitochondrial light strand promoter anti-sense binding | Unigene38848\_All, Unigene39862\_All |
| lactoylglutathione lyase activity | Unigene56803\_All, Unigene56802\_All, CL6364.Contig2\_All, CL4875.Contig2\_All, CL6364.Contig1\_All, CL4875.Contig1\_All, Unigene15533\_All, CL9146.Contig1\_All |
| structural constituent of cell wall | CL4943.Contig2\_All, Unigene33649\_All, Unigene22940\_All, Unigene41308\_All, CL639.Contig2\_All, CL8213.Contig3\_All, CL3949.Contig1\_All, CL6563.Contig1\_All |
| oxidoreductase activity, acting on the CH-CH group of donors, quinone or related compound as acceptor | CL7457.Contig2\_All, CL5672.Contig1\_All, CL5672.Contig2\_All, Unigene12103\_All, CL11002.Contig2\_All, CL12942.Contig1\_All, CL11002.Contig1\_All, Unigene36489\_All |
| monocarboxylic acid binding | CL12446.Contig7\_All, CL9639.Contig3\_All, CL9967.Contig1\_All, Unigene29022\_All, CL9500.Contig2\_All, CL1413.Contig1\_All, Unigene17682\_All, Unigene23030\_All |
| organic phosphonate transmembrane-transporting ATPase activity | CL6921.Contig1\_All, CL13951.Contig1\_All, Unigene46152\_All, Unigene150\_All, Unigene35836\_All, CL1699.Contig1\_All, Unigene253\_All, CL9399.Contig2\_All, CL807.Contig4\_All, Unigene26745\_All, Unigene8561\_All, Unigene57189\_All, Unigene60\_All, CL1103.Contig4\_All, Unigene63929\_All, CL5375.Contig3\_All, CL10994.Contig1\_All |
| organic phosphonate transmembrane transporter activity | CL6921.Contig1\_All, CL13951.Contig1\_All, Unigene46152\_All, Unigene150\_All, Unigene35836\_All, CL1699.Contig1\_All, Unigene253\_All, CL9399.Contig2\_All, CL807.Contig4\_All, Unigene26745\_All, Unigene8561\_All, Unigene57189\_All, Unigene60\_All, CL1103.Contig4\_All, Unigene63929\_All, CL5375.Contig3\_All, CL10994.Contig1\_All |
| organophosphate ester transmembrane transporter activity | CL6921.Contig1\_All, CL13951.Contig1\_All, Unigene46152\_All, Unigene150\_All, Unigene35836\_All, CL1699.Contig1\_All, Unigene253\_All, CL9399.Contig2\_All, CL807.Contig4\_All, Unigene26745\_All, Unigene8561\_All, Unigene57189\_All, Unigene60\_All, CL1103.Contig4\_All, Unigene63929\_All, CL5375.Contig3\_All, CL10994.Contig1\_All |
| cysteine-type endopeptidase inhibitor activity | Unigene41946\_All, CL1005.Contig2\_All, CL1005.Contig1\_All, CL2022.Contig1\_All |
| RNA guanylyltransferase activity | CL2654.Contig1\_All, CL2654.Contig2\_All, CL3612.Contig4\_All, CL3612.Contig1\_All |
| histone kinase activity | CL12982.Contig1\_All, Unigene35757\_All, CL9134.Contig1\_All, Unigene57733\_All |
| endopeptidase inhibitor activity | CL8559.Contig1\_All, Unigene41946\_All, Unigene36095\_All, CL1005.Contig2\_All, CL1005.Contig1\_All, CL8559.Contig2\_All, CL2022.Contig1\_All |
| endopeptidase regulator activity | CL8559.Contig1\_All, Unigene41946\_All, Unigene36095\_All, CL1005.Contig2\_All, CL1005.Contig1\_All, CL8559.Contig2\_All, CL2022.Contig1\_All |
| oxidoreductase activity, oxidizing metal ions | CL10466.Contig1\_All, CL1779.Contig5\_All, CL7377.Contig2\_All, CL10998.Contig1\_All, CL1779.Contig2\_All, Unigene21981\_All |
| desacetoxyvindoline 4-hydroxylase activity | CL581.Contig1\_All, Unigene41430\_All, Unigene59858\_All, Unigene41797\_All, Unigene41520\_All, CL1814.Contig3\_All |
| beta-galactosidase activity | CL3155.Contig3\_All, Unigene61217\_All, CL973.Contig3\_All, CL3160.Contig2\_All, CL3155.Contig1\_All, CL5013.Contig1\_All, Unigene57944\_All, Unigene26617\_All, CL2475.Contig2\_All, CL257.Contig3\_All, CL5013.Contig2\_All, Unigene59369\_All, CL10634.Contig1\_All, CL2475.Contig1\_All, Unigene26454\_All, Unigene62369\_All |
| alpha-L-fucosidase activity | Unigene30112\_All, CL1405.Contig3\_All, Unigene19705\_All, Unigene60999\_All, CL1405.Contig1\_All |
| fucosidase activity | Unigene30112\_All, CL1405.Contig3\_All, Unigene19705\_All, Unigene60999\_All, CL1405.Contig1\_All |
| single-stranded RNA binding | Unigene47188\_All, Unigene32496\_All, CL1430.Contig13\_All, Unigene54413\_All, CL8052.Contig1\_All, Unigene39862\_All, CL1430.Contig4\_All, CL1430.Contig11\_All, CL837.Contig2\_All, Unigene54419\_All, CL10298.Contig1\_All, Unigene22400\_All, CL12272.Contig1\_All, Unigene54418\_All, CL6792.Contig1\_All, Unigene54422\_All, CL5285.Contig1\_All, Unigene23124\_All, Unigene54412\_All, Unigene33591\_All, Unigene28108\_All, Unigene33589\_All, Unigene38848\_All, Unigene22270\_All, CL1430.Contig6\_All, Unigene31261\_All, Unigene34854\_All |
| fatty acid synthase activity | CL2228.Contig5\_All, Unigene60021\_All, Unigene41803\_All, CL1019.Contig6\_All, Unigene13363\_All, CL4483.Contig2\_All, CL8123.Contig1\_All, CL9651.Contig1\_All, Unigene9821\_All, CL13362.Contig1\_All, CL12434.Contig3\_All, CL9651.Contig3\_All, Unigene56272\_All, CL4483.Contig3\_All, CL6499.Contig1\_All, CL4483.Contig1\_All, CL12434.Contig2\_All |
| carbohydrate binding | CL10110.Contig2\_All, CL12460.Contig1\_All, CL6646.Contig2\_All, Unigene15863\_All, Unigene25173\_All, Unigene64775\_All, CL2008.Contig2\_All, Unigene41793\_All, CL982.Contig3\_All, CL11926.Contig1\_All, Unigene40795\_All, CL271.Contig1\_All, CL2937.Contig6\_All, Unigene62369\_All, CL2588.Contig2\_All, CL3155.Contig3\_All, Unigene61217\_All, CL13926.Contig2\_All, CL2992.Contig16\_All, CL2008.Contig1\_All, CL8086.Contig1\_All, CL11228.Contig1\_All, CL601.Contig3\_All, Unigene11131\_All, CL5360.Contig4\_All, Unigene24573\_All, CL12950.Contig1\_All, CL982.Contig6\_All, Unigene23273\_All, Unigene59369\_All, CL5013.Contig2\_All, CL13764.Contig1\_All, CL2475.Contig1\_All, CL1895.Contig15\_All, CL2588.Contig1\_All, CL982.Contig5\_All, Unigene5950\_All, Unigene19891\_All, CL3155.Contig1\_All, CL271.Contig2\_All, Unigene32356\_All, CL13926.Contig1\_All, Unigene26617\_All, CL3346.Contig1\_All, CL11228.Contig2\_All, CL10634.Contig1\_All, CL982.Contig2\_All, CL10110.Contig1\_All, Unigene239\_All, CL2992.Contig12\_All, CL1895.Contig13\_All, Unigene55955\_All, CL1105.Contig7\_All, CL7479.Contig3\_All, CL973.Contig3\_All, CL9922.Contig1\_All, CL2937.Contig3\_All, Unigene14791\_All, CL5013.Contig1\_All, Unigene58480\_All, CL982.Contig1\_All, CL982.Contig4\_All, CL3993.Contig3\_All, CL5360.Contig3\_All, Unigene57944\_All, Unigene42163\_All, Unigene58374\_All, CL2475.Contig2\_All, CL257.Contig3\_All, CL1895.Contig14\_All, Unigene6108\_All, CL3346.Contig2\_All, Unigene26454\_All, Unigene58305\_All |
| amine transmembrane transporter activity | Unigene57402\_All, CL4687.Contig2\_All, CL73.Contig3\_All, Unigene376\_All, Unigene23271\_All, CL4247.Contig6\_All, CL73.Contig2\_All, CL73.Contig1\_All, CL12439.Contig1\_All |
| oxidoreductase activity, acting on NAD(P)H, quinone or similar compound as acceptor | Unigene60317\_All, CL23.Contig1\_All, CL1239.Contig2\_All, Unigene29003\_All, CL11074.Contig2\_All, CL815.Contig2\_All, CL7712.Contig1\_All, CL4812.Contig3\_All, Unigene589\_All, Unigene32386\_All, CL1347.Contig3\_All, Unigene62303\_All, CL2221.Contig4\_All, Unigene7288\_All, CL10399.Contig2\_All, Unigene34452\_All, CL4405.Contig4\_All, CL4209.Contig1\_All, CL1239.Contig1\_All, Unigene27257\_All, CL3764.Contig1\_All, CL4812.Contig5\_All, CL1425.Contig2\_All, CL7072.Contig1\_All, CL4782.Contig2\_All, CL7072.Contig2\_All, CL1375.Contig1\_All, Unigene15519\_All, CL11074.Contig1\_All, CL6810.Contig4\_All, CL2825.Contig1\_All, CL4044.Contig1\_All, CL7712.Contig5\_All, CL3764.Contig2\_All, CL2003.Contig6\_All, Unigene4069\_All, CL7712.Contig2\_All |
| sucrose synthase activity | Unigene62466\_All, CL1872.Contig1\_All, CL9785.Contig1\_All, Unigene20159\_All, CL9785.Contig2\_All, CL2746.Contig5\_All, CL2746.Contig1\_All, CL7600.Contig2\_All |
| galactosidase activity | CL3155.Contig3\_All, Unigene61217\_All, CL973.Contig3\_All, CL3160.Contig2\_All, CL3155.Contig1\_All, CL5013.Contig1\_All, Unigene57944\_All, Unigene26617\_All, CL2475.Contig2\_All, CL8395.Contig2\_All, CL257.Contig3\_All, CL5013.Contig2\_All, Unigene59369\_All, CL10634.Contig1\_All, CL2475.Contig1\_All, Unigene26454\_All, Unigene62369\_All |
| steroid dehydrogenase activity | CL2228.Contig5\_All, CL9128.Contig2\_All, CL9128.Contig1\_All, CL3610.Contig2\_All, CL3443.Contig2\_All, CL3610.Contig7\_All, Unigene30214\_All, Unigene10603\_All, CL2372.Contig1\_All, CL3610.Contig5\_All, Unigene29396\_All, Unigene59012\_All, CL12503.Contig2\_All, Unigene60039\_All, Unigene61476\_All |
| tRNA (guanine-N7-)-methyltransferase activity | Unigene59092\_All, CL6122.Contig1\_All, CL6122.Contig2\_All |
| xylose isomerase activity | Unigene40253\_All, CL11370.Contig1\_All, Unigene59822\_All |
| L-alanine transmembrane transporter activity | Unigene376\_All, CL5086.Contig2\_All, CL5086.Contig1\_All |
| alanine transmembrane transporter activity | Unigene376\_All, CL5086.Contig2\_All, CL5086.Contig1\_All |
| L-tryptophan aminotransferase activity | Unigene4967\_All, Unigene56757\_All, Unigene14568\_All |
| carbon-oxygen lyase activity, acting on polysaccharides | CL13810.Contig1\_All, CL27.Contig2\_All, CL27.Contig3\_All, Unigene23148\_All, Unigene27818\_All, CL13810.Contig2\_All, Unigene23727\_All, CL11268.Contig2\_All, CL11268.Contig1\_All, Unigene30689\_All, Unigene14059\_All |
| pectate lyase activity | CL13810.Contig1\_All, CL27.Contig2\_All, CL27.Contig3\_All, Unigene23148\_All, Unigene27818\_All, CL13810.Contig2\_All, Unigene23727\_All, CL11268.Contig2\_All, CL11268.Contig1\_All, Unigene30689\_All, Unigene14059\_All |
| glycerate dehydrogenase activity | Unigene54412\_All, Unigene54422\_All, Unigene54418\_All, Unigene54413\_All, Unigene54419\_All |
| abscisic acid binding | CL12446.Contig7\_All, CL9639.Contig3\_All, CL1413.Contig1\_All, CL9500.Contig2\_All, Unigene17682\_All |
| methylammonium transmembrane transporter activity | Unigene23271\_All, CL73.Contig2\_All, CL4247.Contig6\_All, CL73.Contig1\_All, CL73.Contig3\_All |
| isoprenoid binding | CL12446.Contig7\_All, CL9639.Contig3\_All, CL1413.Contig1\_All, CL9500.Contig2\_All, Unigene17682\_All |
| ammonia transmembrane transporter activity | Unigene23271\_All, CL73.Contig2\_All, CL4247.Contig6\_All, CL73.Contig1\_All, CL73.Contig3\_All |
| pyrimidine nucleotide-sugar transmembrane transporter activity | Unigene28694\_All, CL410.Contig3\_All, CL410.Contig2\_All, CL410.Contig1\_All |
| cadmium-transporting ATPase activity | CL2989.Contig3\_All, Unigene60246\_All, Unigene30755\_All, CL11162.Contig3\_All |
| racemase and epimerase activity, acting on hydroxy acids and derivatives | CL9060.Contig1\_All, CL6487.Contig3\_All, CL6726.Contig2\_All, CL6726.Contig1\_All |
| NADPH binding | CL1986.Contig2\_All, Unigene14655\_All, CL2260.Contig2\_All, Unigene15289\_All |
| CoA-ligase activity | Unigene42146\_All, CL5890.Contig3\_All, CL5590.Contig3\_All, CL1531.Contig5\_All, Unigene32589\_All, Unigene35795\_All, Unigene54825\_All, CL3536.Contig2\_All, CL6301.Contig3\_All, CL5890.Contig1\_All, CL5590.Contig4\_All, Unigene20287\_All, Unigene24359\_All, CL5590.Contig5\_All, Unigene140\_All, CL8940.Contig1\_All, Unigene30813\_All, Unigene376\_All, CL1292.Contig2\_All, Unigene20061\_All, CL8940.Contig4\_All, CL6301.Contig1\_All, CL8940.Contig5\_All |
| xylosyltransferase activity | CL203.Contig23\_All, Unigene61104\_All, CL203.Contig26\_All, CL413.Contig3\_All, Unigene61193\_All, CL2727.Contig1\_All, CL413.Contig1\_All, Unigene59961\_All, CL203.Contig28\_All, CL203.Contig24\_All |
| actin filament binding | CL4399.Contig2\_All, CL9632.Contig1\_All, CL13731.Contig7\_All, CL7140.Contig10\_All, CL13731.Contig1\_All, CL1708.Contig2\_All, CL11637.Contig1\_All, CL9948.Contig1\_All, CL6394.Contig4\_All, CL10535.Contig11\_All, CL9084.Contig2\_All, Unigene9135\_All, Unigene56318\_All, CL13731.Contig5\_All, Unigene59050\_All, CL9084.Contig1\_All, CL13731.Contig2\_All, Unigene61589\_All, CL13731.Contig3\_All, Unigene59423\_All, Unigene19646\_All, Unigene58233\_All, Unigene30574\_All, CL7140.Contig8\_All, Unigene24785\_All |
| translation factor activity, nucleic acid binding | CL6733.Contig2\_All, Unigene23394\_All, CL7395.Contig5\_All, CL8248.Contig7\_All, Unigene16902\_All, CL13861.Contig5\_All, Unigene56323\_All, Unigene60069\_All, Unigene14583\_All, Unigene56196\_All, Unigene236\_All, Unigene58798\_All, CL6818.Contig2\_All, CL4186.Contig2\_All, CL1198.Contig3\_All, Unigene60458\_All, CL13379.Contig2\_All, Unigene30740\_All, CL7737.Contig1\_All, CL10623.Contig2\_All, CL7231.Contig1\_All, CL13378.Contig1\_All, CL2786.Contig6\_All, CL7737.Contig2\_All, CL9286.Contig1\_All, CL2782.Contig1\_All, CL10770.Contig1\_All, CL6998.Contig1\_All, Unigene43054\_All, CL2987.Contig1\_All, Unigene20195\_All, Unigene27997\_All, CL2786.Contig14\_All, Unigene32059\_All, Unigene3973\_All, CL5176.Contig4\_All, CL1443.Contig1\_All, Unigene43514\_All, Unigene8835\_All, CL161.Contig1\_All, CL2340.Contig1\_All, Unigene12169\_All, Unigene61490\_All, Unigene20080\_All, CL13641.Contig1\_All, CL2153.Contig1\_All, Unigene56324\_All, Unigene27330\_All, CL9286.Contig2\_All, CL8076.Contig2\_All, Unigene6711\_All, CL7395.Contig3\_All, CL4298.Contig1\_All, Unigene61511\_All, Unigene28581\_All, Unigene19707\_All, Unigene6773\_All, CL13973.Contig1\_All, Unigene32107\_All, CL13378.Contig2\_All, Unigene41282\_All, Unigene33797\_All, CL2907.Contig3\_All, Unigene57190\_All, CL7539.Contig2\_All, CL2786.Contig21\_All, Unigene40080\_All, Unigene42887\_All, Unigene40532\_All, Unigene29507\_All, Unigene30945\_All, Unigene60740\_All, CL12417.Contig11\_All, CL2340.Contig2\_All, Unigene18154\_All, CL3972.Contig3\_All, Unigene42950\_All, CL10201.Contig2\_All, Unigene27024\_All, Unigene24620\_All, CL9323.Contig2\_All, CL2256.Contig4\_All, Unigene54665\_All, CL12408.Contig2\_All, CL8843.Contig3\_All, Unigene30448\_All, Unigene57248\_All, CL13496.Contig1\_All, CL2256.Contig2\_All, CL12408.Contig3\_All, Unigene23393\_All, CL2782.Contig2\_All, CL12484.Contig2\_All, Unigene6846\_All, Unigene59157\_All, Unigene12371\_All, Unigene38355\_All, Unigene41202\_All, Unigene36263\_All, CL4862.Contig1\_All, CL13641.Contig3\_All, CL2256.Contig1\_All, CL11514.Contig1\_All, Unigene29969\_All, Unigene24094\_All, CL8076.Contig1\_All, Unigene14807\_All, Unigene1316\_All, Unigene5883\_All, CL4796.Contig6\_All, Unigene14730\_All, CL8349.Contig1\_All, CL5809.Contig5\_All, CL5000.Contig1\_All, CL7231.Contig2\_All, CL10354.Contig1\_All, CL12484.Contig1\_All, CL2987.Contig2\_All, CL8349.Contig3\_All, CL13379.Contig1\_All, CL9286.Contig3\_All, CL10623.Contig1\_All, Unigene63946\_All, CL5176.Contig5\_All, CL2320.Contig3\_All, CL2071.Contig5\_All, CL2320.Contig2\_All |
| efflux transmembrane transporter activity | Unigene59649\_All, Unigene35631\_All, Unigene7976\_All, Unigene59760\_All, Unigene63864\_All, CL13885.Contig2\_All, Unigene39613\_All, Unigene41679\_All, CL13885.Contig5\_All, Unigene56646\_All, CL3975.Contig2\_All, Unigene20789\_All, CL13885.Contig1\_All, CL10296.Contig2\_All, CL13885.Contig9\_All, Unigene36211\_All, Unigene932\_All, CL3622.Contig2\_All, CL2129.Contig2\_All, Unigene61641\_All, Unigene35383\_All |
| N,N-dimethylaniline monooxygenase activity | Unigene57584\_All, CL2587.Contig1\_All, CL1690.Contig1\_All, CL10040.Contig2\_All, CL7843.Contig3\_All, Unigene60207\_All, Unigene57585\_All |
| methyl jasmonate esterase activity | CL13215.Contig2\_All, CL12119.Contig2\_All, CL3394.Contig2\_All, CL9309.Contig2\_All, Unigene32360\_All, CL9188.Contig1\_All, CL3442.Contig5\_All |
| L-aspartate:2-oxoglutarate aminotransferase activity | CL514.Contig3\_All, CL6493.Contig1\_All, CL6717.Contig4\_All, CL12061.Contig2\_All, CL4718.Contig3\_All, CL889.Contig2\_All, Unigene38488\_All, CL6717.Contig3\_All, CL6717.Contig2\_All, CL10823.Contig2\_All, CL12061.Contig3\_All |
| zinc ion transmembrane transporter activity | Unigene6374\_All, CL10427.Contig4\_All, CL10427.Contig1\_All, Unigene54678\_All, CL11162.Contig3\_All, Unigene15416\_All, CL2989.Contig3\_All, Unigene54999\_All, CL10427.Contig3\_All |
| basic amino acid transmembrane transporter activity | Unigene55085\_All, Unigene376\_All, CL5086.Contig2\_All, CL8130.Contig1\_All, CL5086.Contig1\_All, Unigene21269\_All |
| acid-thiol ligase activity | Unigene42146\_All, CL5890.Contig3\_All, CL5590.Contig3\_All, CL1531.Contig5\_All, Unigene32589\_All, Unigene35795\_All, Unigene54825\_All, CL3536.Contig2\_All, CL6301.Contig3\_All, CL5890.Contig1\_All, CL5590.Contig4\_All, Unigene20287\_All, Unigene24359\_All, CL5590.Contig5\_All, Unigene140\_All, CL8940.Contig1\_All, Unigene30813\_All, Unigene376\_All, CL1292.Contig2\_All, Unigene20061\_All, CL8940.Contig4\_All, CL6301.Contig1\_All, CL8940.Contig5\_All |
| alpha-1,6-mannosyltransferase activity | CL850.Contig2\_All, CL850.Contig1\_All |
| gamma-glutamyltransferase activity | CL2340.Contig1\_All, CL2340.Contig2\_All |
| cholinesterase activity | CL1405.Contig3\_All, CL1405.Contig1\_All |
| leukotriene-A4 hydrolase activity | Unigene40987\_All, Unigene22882\_All |
| Rab geranylgeranyltransferase activity | CL7660.Contig3\_All, CL7660.Contig1\_All |
| sialyltransferase activity | CL3710.Contig1\_All, CL3710.Contig2\_All |
| nitrate:hydrogen symporter activity | CL9614.Contig1\_All, Unigene55976\_All |
| 16:0 monogalactosyldiacylglycerol desaturase activity | CL8523.Contig1\_All, Unigene7124\_All |
| acireductone dioxygenase [iron(II)-requiring] activity | CL4791.Contig2\_All, CL4791.Contig1\_All |
| L-galactose-1-phosphate phosphatase activity | CL9234.Contig2\_All, Unigene60305\_All |
| gamma-aminobutyric acid transmembrane transporter activity | Unigene376\_All, CL4687.Contig2\_All |
| lysophosphatidic acid acyltransferase activity | CL8660.Contig1\_All, CL8660.Contig2\_All |
| galactinol-raffinose galactosyltransferase activity | CL1842.Contig1\_All, CL8912.Contig1\_All |
| GDP-mannose 3,5-epimerase activity | CL4972.Contig3\_All, CL4972.Contig1\_All |
| galactose-1-phosphate phosphatase activity | CL9234.Contig2\_All, Unigene60305\_All |
| 5S rDNA binding | Unigene37315\_All, Unigene43305\_All |
| cysteine synthase activity | CL7896.Contig2\_All, CL3624.Contig3\_All, CL10469.Contig3\_All, CL7896.Contig1\_All, CL3624.Contig4\_All |
| pyruvate decarboxylase activity | Unigene34735\_All, Unigene34737\_All, CL4825.Contig2\_All, Unigene34736\_All, CL4825.Contig1\_All |
| succinate-CoA ligase (GDP-forming) activity | Unigene32589\_All, Unigene20287\_All, Unigene54825\_All, CL1531.Contig5\_All, Unigene140\_All |
| cationic amino acid transmembrane transporter activity | CL384.Contig2\_All, CL2661.Contig3\_All, CL2661.Contig2\_All, CL384.Contig5\_All, CL384.Contig3\_All |
| xenobiotic-transporting ATPase activity | Unigene7976\_All, Unigene63864\_All, Unigene56095\_All, Unigene41679\_All, Unigene55203\_All, Unigene56646\_All, CL3975.Contig2\_All, Unigene20789\_All, CL2225.Contig2\_All, CL8910.Contig2\_All, CL10296.Contig2\_All, Unigene56096\_All, Unigene56094\_All, Unigene56097\_All, CL3622.Contig2\_All, CL2129.Contig2\_All, Unigene16770\_All, CL2225.Contig1\_All, Unigene35383\_All, Unigene17765\_All, Unigene60226\_All |
| xenobiotic transporter activity | Unigene7976\_All, Unigene63864\_All, Unigene56095\_All, Unigene41679\_All, Unigene55203\_All, Unigene56646\_All, CL3975.Contig2\_All, Unigene20789\_All, CL2225.Contig2\_All, CL8910.Contig2\_All, CL10296.Contig2\_All, Unigene56096\_All, Unigene56094\_All, Unigene56097\_All, CL3622.Contig2\_All, CL2129.Contig2\_All, Unigene16770\_All, CL2225.Contig1\_All, Unigene35383\_All, Unigene17765\_All, Unigene60226\_All |
[truncated: 1,446,128 more chars]
