# Supplementary figures and images for: Transcriptomic Analysis Based on RNA-Seq Technology Reveals the Molecular Mechanisms of Sunflower (Helianthus annuus L.) Response to Salt Stress
Source: Genes (Basel). 2026 May 30;17(6):629. doi: 10.3390/genes17060629 (PMC13298483; doi:10.3390/genes17060629)

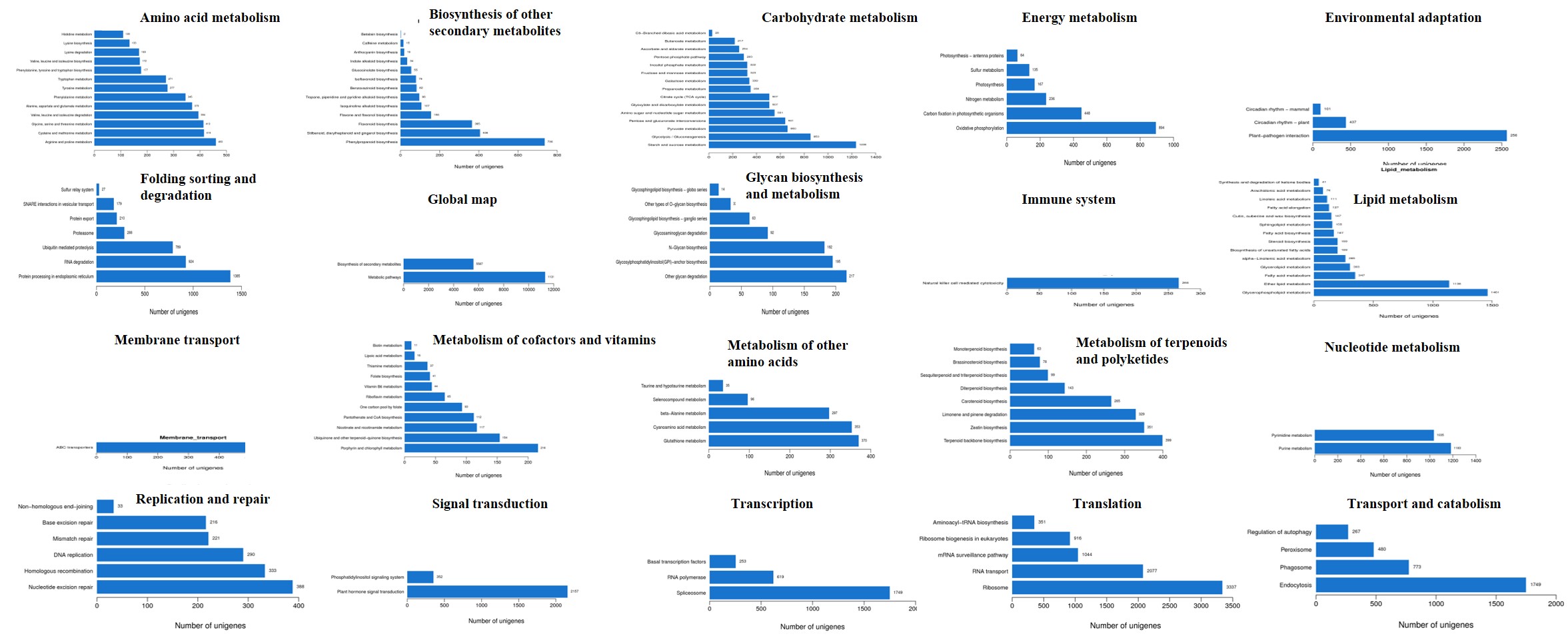

Supplement: Supplementary file 1 [file genes-17-00629-s001.zip › Figure S1.jpg]

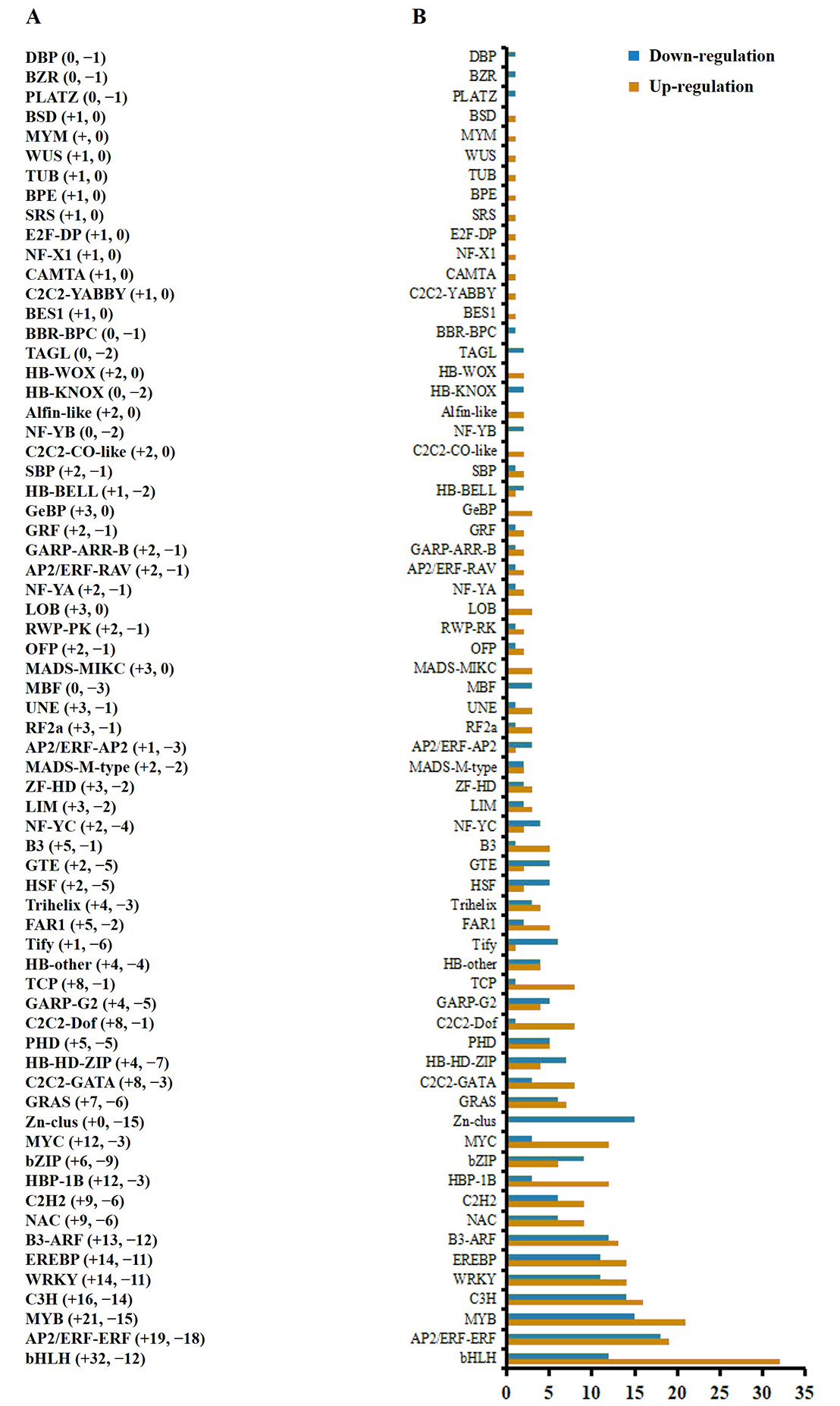

Supplement: Supplementary file 1 [file genes-17-00629-s001.zip › Figure S2.jpg]
